# Integrative DNA Methylation and Gene Expression Analyses Identify DNA Packaging and Epigenetic Regulatory Genes Associated with Low Motility Sperm

**DOI:** 10.1371/journal.pone.0020280

**Published:** 2011-06-02

**Authors:** Sara E. Pacheco, E. Andres Houseman, Brock C. Christensen, Carmen J. Marsit, Karl T. Kelsey, Mark Sigman, Kim Boekelheide

**Affiliations:** 1 Department of Pathology and Laboratory Medicine, Brown University, Providence, Rhode Island, United States of America; 2 Department of Community Health, Brown University, Providence, Rhode Island, United States of America; 3 Division of Urology, Brown University, Providence, Rhode Island, United States of America; Florida State University, United States of America

## Abstract

**Background:**

In previous studies using candidate gene approaches, low sperm count (oligospermia) has been associated with altered sperm mRNA content and DNA methylation in both imprinted and non-imprinted genes. We performed a genome-wide analysis of sperm DNA methylation and mRNA content to test for associations with sperm function.

**Methods and Results:**

Sperm DNA and mRNA were isolated from 21 men with a range of semen parameters presenting to a tertiary male reproductive health clinic. DNA methylation was measured with the Illumina Infinium array at 27,578 CpG loci. Unsupervised clustering of methylation data differentiated the 21 sperm samples by their motility values. Recursively partitioned mixture modeling (RPMM) of methylation data resulted in four distinct methylation profiles that were significantly associated with sperm motility (*P* = 0.01). Linear models of microarray analysis (LIMMA) was performed based on motility and identified 9,189 CpG loci with significantly altered methylation (*Q*<0.05) in the low motility samples. In addition, the majority of these disrupted CpG loci (80%) were hypomethylated. Of the aberrantly methylated CpGs, 194 were associated with imprinted genes and were almost equally distributed into hypermethylated (predominantly paternally expressed) and hypomethylated (predominantly maternally expressed) groups. Sperm mRNA was measured with the Human Gene 1.0 ST Affymetrix GeneChip Array. LIMMA analysis identified 20 candidate transcripts as differentially present in low motility sperm, including *HDAC1* (NCBI 3065), *SIRT3* (NCBI 23410), and *DNMT3A* (NCBI 1788). There was a trend among altered expression of these epigenetic regulatory genes and RPMM DNA methylation class.

**Conclusions:**

Using integrative genome-wide approaches we identified CpG methylation profiles and mRNA alterations associated with low sperm motility.

## Introduction

Traditional semen analysis measures sperm concentration, motility, morphology, and semen volume, and is acknowledged to be a poor predictor of fertility, demonstrating remarkable intra- and inter-individual variability [1], [2]. Because of these limitations, effort has been devoted to developing sperm molecular biomarkers that may better and more stably reflect sperm function.

DNA methylation is the stable, covalent addition of a methyl group to cytosine that can represent response to environmental cues or exposures that may modify gene expression. Both human and animal studies indicate that abnormal sperm DNA methylation patterns are associated with subfertility, including aberrant methylation of both imprinted [3]–[11] and non-imprinted genes [4], [12], [13] in oligospermic men.

In addition to DNA methylation, significant effort is being devoted to developing human sperm mRNAs as biomarkers of infertility [14]–[30]. The discovery of mRNAs in mature sperm shook the long-held belief that the sole purpose of sperm was to deliver its DNA to the egg [14]. Recent evidence indicates that some of these transcripts may be intentionally transported to the oocyte to aid embryogenesis, since some sperm mRNAs are found to persist in the zygote and are functionally important [14], [27], [28]. In addition, remnant sperm mRNAs provide a record of the spermatogenic environment and may have clinical applications as novel biomarkers of fertility status [15]–[26].

In the present study, we utilized high-density array techniques to investigate the hypothesis that alterations to the pattern of sperm DNA methylation or mRNA content are associated with sperm function.

## Materials and Methods

### Ethics Statement

The Committee on the Protection of Human Subjects: Rhode Island Hospital Institutional Review Board 2 (Committee #403908) approved the study and written informed consent was obtained from all participants. Clinical investigation was conducted according to the principles expressed in the Declaration of Helsinki.

### Microarray DataSets

The microarray data discussed in this publication is MAIME compliant and the raw data has been deposited in NCBI's Gene Expression Omnibus (Edgar *et al.*, 2002) as detailed in the MGED Society website http://www.mged.org/Workgroups/MAIME/maime.html. This data is accessible through GEO Series accession number GSE26982 (http://www.ncbi.nlm.nih.gov/geo/query/acc.cgi?acc=GSE26982).

### Patient Population, Semen Analysis, and Sperm Isolation

Study subjects presented for semen evaluation at Rhode Island Hospital's tertiary male reproductive health clinic. Samples were collected from 21 men with unknown fertility status and a range of semen characteristics (Table 1). During the semen analysis, morphology was scored using Kruger strict criteria and total motility was calculated as described in the WHO laboratory manual (2010) [31].

**Table 1:** Semen Parameters of Subjects Examined.

Subject ID	Motility (%)	Morphology (%)	Count (×10^6^/ml)
16	77	11	132
8	74	4.5	18
26	70	6	80
5	64	8	44
22	64	5.5	67
29	63	7	174
35	62	8	103
13*	60	8	26
28	60	10	72
10	57	10	58
31*	57	4	30
11	52	8	49
36	51	3	44
37	49	1	9
32	46	8.5	23
23	45	1	67
33*	41	6	1.6
34	39	4	20
40	34	4	72
9	30	3.5	7
41	21	0	15

Note: Patients with * were excluded from the mRNA analysis due to low yield.

After clinical analysis the samples were divided into one quarter and three quarter aliquots for DNA and RNA isolations, respectively. Each group was processed through an optimized Percoll (GE Healthcare, Uppsala, Sweden) gradient to eliminate debris, non-sperm cells, and dead sperm [32]. Briefly, 1 ml of the fresh semen was applied to a monolayer of 50% Percoll. After centrifugation, the upper and interface layers containing the dead sperm and other somatic contaminants were aspirated off, leaving the sperm enriched fraction. The sperm fraction was washed with phosphate buffered saline and the purified sperm samples were processed immediately for mRNA and DNA isolation.

Prior to processing the 21 samples, sperm purity was confirmed by the absence of somatic cell contaminants using bright phase microscopy and by the absence of 18/28S ribosomal RNA peaks by RNA gel electrophoresis (data not shown) [19], [21].

### DNA Isolation, Bisulfite Modification, and Illumina Infinium HumanMethylation27 BeadChip Array

DNA was isolated from the sperm of the 21 men using a modified protocol in which sperm pellets were lysed for 16 hours in a solution containing Tris (Fisher Scientific, Pittsburgh, PA, USA), DTT (Promega Corporation, Madison, WI, USA), NaCl (EMD Chemicals, Inc., the North American Affiliate of Merck KGaA, Darmstadt, Germany), EDTA (Fisher Scientific, Pittsburgh, PA, USA), SDS (Fisher Scientific, Pittsburgh, PA, USA), Proteinase K (Promega Corporation, Madison, WI, USA), and beta-mercaptoethanol (Sigma-Aldrich, St. Louis, MO, USA) [33]. The DNA was then extracted using phenol/chloroform (Sigma-Aldrich, St. Louis, MO, USA), ethanol precipitated, and bisulfite modified using the EZ DNA Methylation kit (Zymo Research Corporation, Orange, CA, USA). Genome-wide scanning for DNA methylation was performed using the Illumina Infinium HumanMethylation27 BeadChip assay (Illumina, Inc., San Diego, CA, USA) to determine the methylation state at 27,578 CpG sites spanning more than 14,000 genes; and on this array, there are 616 CpGs associated with 187 imprinted genes identified using the array's annotation file (HumanMethylation27_270596_v.1.2, www.Illumina.com). Multiple groups including ours have previously demonstrated the validity of Illumina methylation array data using several different approaches [34]–[39].

### Imprinted Genes

A list of 187 imprinted genes in the human genome was compiled based on information from three sources: (1) experimentally determined imprinted genes listed in two databases (http://www.geneimprint.com/databases/ and http://igc.otago.ac.nz/home.html) (n = 62); (2) imprinted genes identified using the ChIP-SNP method (n = 27) [40]; and (3) protein-coding genes from the 156 putatively imprinted sequences that correspond to known genes listed by NCBI (n = 106) [41]. Taken together, a final list of 187 imprinted genes is identified from these three sources (Table S1).

### mRNA Isolation and Affymetrix GeneChip Human Gene 1.0 ST Array

Sperm mRNA was extracted from 18 of the 21 men using a modified Stat 60 (IsoTex Diagnostics, Inc., Friendswood TX, USA) protocol in addition to components of Qiagen's RNeasy kit (Qiagen Sciences, Germantown, MD, USA). Using the Brown Genomics Core Facility, the isolated sperm mRNA was processed and hybridized to Affymetrix GeneChip Human Gene 1.0 ST Arrays (Affymetrix, Santa Clara, CA, USA), providing whole-transcript coverage of 28,869 genes by ∼26 probes spread across the length of each gene. The probe cell intensity data from the Affymetrix GeneChips was normalized and annotated using Affymetrix Expression Console as recommended by the manufacturer. The application uses the RMA-Sketch workflow analysis as the default to create CHP files. The CHP log2 expression files were then merged in Expression Console with the annotation file and the annotated log2 results were exported as a text file for third-party downstream analysis.

### Statistical Analyses

Aside from array normalization procedures, the R software environment (R Foundation for Statistical Computing, Vienna, Austria) was used for all statistical analysis.

### Recursively Partitioned Mixture Modeling

Recursively partitioned mixture modeling (RPMM) profiles were fit to the entire Infinium array using previously described methods [42]. This method builds classes of samples based upon the similarity of methylation profiles by recursively splitting samples into parsimoniously differentiated classes. The classes are identified by pattern of branching into right (R) or left (L) arms. Permutation tests (5,000 permutations run with the Kruskal-Wallis [KW] test statistic) were used to test associations between RPMM class and the 3 clinical fertility variables: count, motility and morphology, using the values in Table 1. Our test statistic was the maximum of the KW test statistic, and the null distribution for this test statistic was obtained by the permutation. Semen parameters were considered significantly associated with RPMM profiles when P<0.02, after Bonferroni correction for multiple comparisons.

### Quantitative Analysis of the DNA Methylation Status of All CpGs

The LIMMA procedure [43] (R package limma) utilized a matrix design containing the 21 samples and their corresponding percent motility values listed in Table 1 to fit a simple linear regression model for each CpG dinucleotide. This univariately tests each CpG for association between methylation and sperm motility. LIMMA results provided estimates of strength and direction of association between CpG methylation and sperm motility and were adjusted for multiple comparisons with the qvalue package in R [44]. CpGs with positive slopes were interpreted as hypomethylated in low motility sperm and CpGs with negative slopes were interpreted as hypermethylated in low motility sperm.

### mRNA Content Analysis of Candidate Transcripts

The transcript presence of the 276 candidate genes was tested using the same statistical strategy as the CpG analysis except here the design matrix was limited to the 18 samples with array data and the slopes were transformed into fold change values. The Affymetrix platform yielded a dataset with ∼28,000 transcripts to assess. However, sperm contain a limited transcriptome (∼5000 transcripts) with few (∼400) consistently expressed in sperm [22]. Therefore, we assessed 276 genes where an *a priori* hypothesis for association with subfertility existed based on previous reports. The analysis included 177 imprinted genes (10 of the 187 potential imprinted genes were not present on the Affymetrix array) as well as 99 candidate genes with biallelic expression (Table S1 and Table S2) [10], [11], [13], [24], [26], [29], [45]–[49].

### Statistical Analysis Comparing Associations Among RPMM Classes and Candidate Genes

Associations among the RPMM classes and the normalized gene expression values for candidate transcripts were calculated with the KW test statistic utilizing the strategy employed previously. Messenger RNAs were considered significantly associated with RPMM class when P<0.02, after adjusting for multiple comparisons using the Bonferroni correction.

## Results

### Sperm DNA Methylation Profiles Cluster by Motility

**Figure 1:**
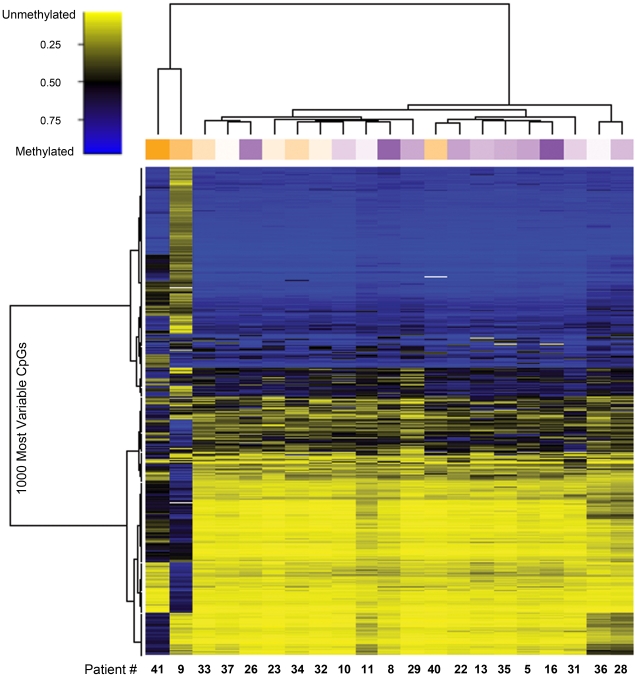
Unsupervised clustering of the 1,000 most variable CpG loci average beta values. The dendrograms above the heatmap show unsupervised clustering based on the methylation data alone, using a Euclidean metric with Ward's method of hierarchical clustering. Patients are represented by column (n = 21) and CpG loci (n = 1000) by row. Each cell represents the CpG level of methylation for one site in one sample. The methylation scale indicates the level of methylation: yellow = sample predominantly unmethylated (0–49% methylated); black = 50% of the sample is methylated; blue = sample predominantly methylated (51–100%). The column annotation track shows motility: orange = low, purple = high.

**Figure 2:**
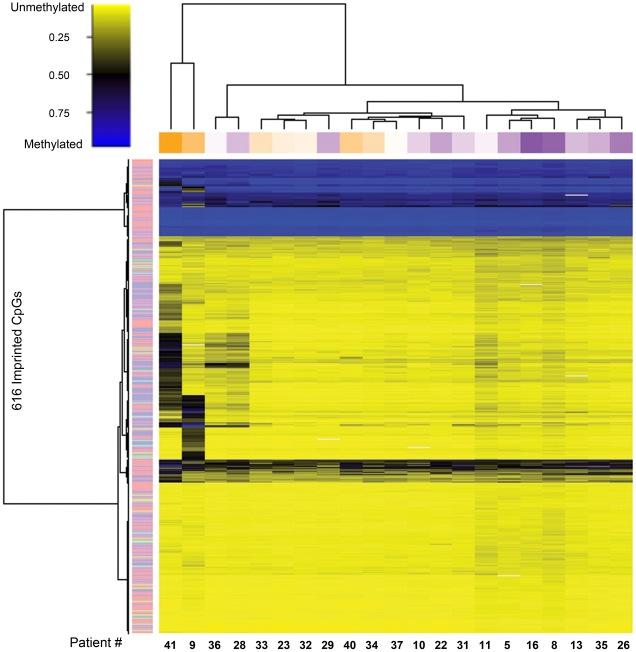
Heatmap displaying the methylation status of CpG loci related to known and predicted imprinted genes. The dendrograms above the heatmap show unsupervised clustering based on the methylation data alone, using a Euclidean metric with Ward's method of hierarchical clustering. Patients are represented by column (n = 21) and CpG loci by row (n = 616). Each cell represents the CpG level of methylation for one site in one sample. The methylation scale indicates the level of methylation: yellow = sample predominantly unmethylated (0–49% methylated); black = 50% of the sample is methylated; blue = sample predominantly methylated (51–100%). The column annotation track shows motility: orange = low, purple = high. The row annotation track shows type of imprinting: pink = maternally expressed, light blue = paternally expressed, light green = not determined.

Unsupervised clustering of sperm DNA methylation data for the 1,000 most variable CpG loci on the array highlights the methylation differences among the 21 individual men (Figure 1). As shown in the column annotation track, the clustering differentiated men based upon the motility of their sperm, with high motility samples (dark purple) clustering together and low motility samples (dark orange) clustering together, with intermediate shades between. The DNA methylation of CpGs within imprinted genes is established during spermatogenesis and maintained in mature spermatozoa. In addition, several laboratories have shown alterations at imprinted loci to occur more frequently in men with sperm abnormalities [3]–[8], [10], [11]. Thus, we hypothesized that imprinted loci may be specifically targeted for aberrant methylation in low motility sperm and separately clustered the 616 CpG loci associated with the 187 imprinted genes present on the array. We observed the same overall trend, with high motility samples clustering together and low motility samples clustering together (Figure 2).

### Sperm DNA Methylation Profiles are Significantly Associated with Motility

**Figure 3:**
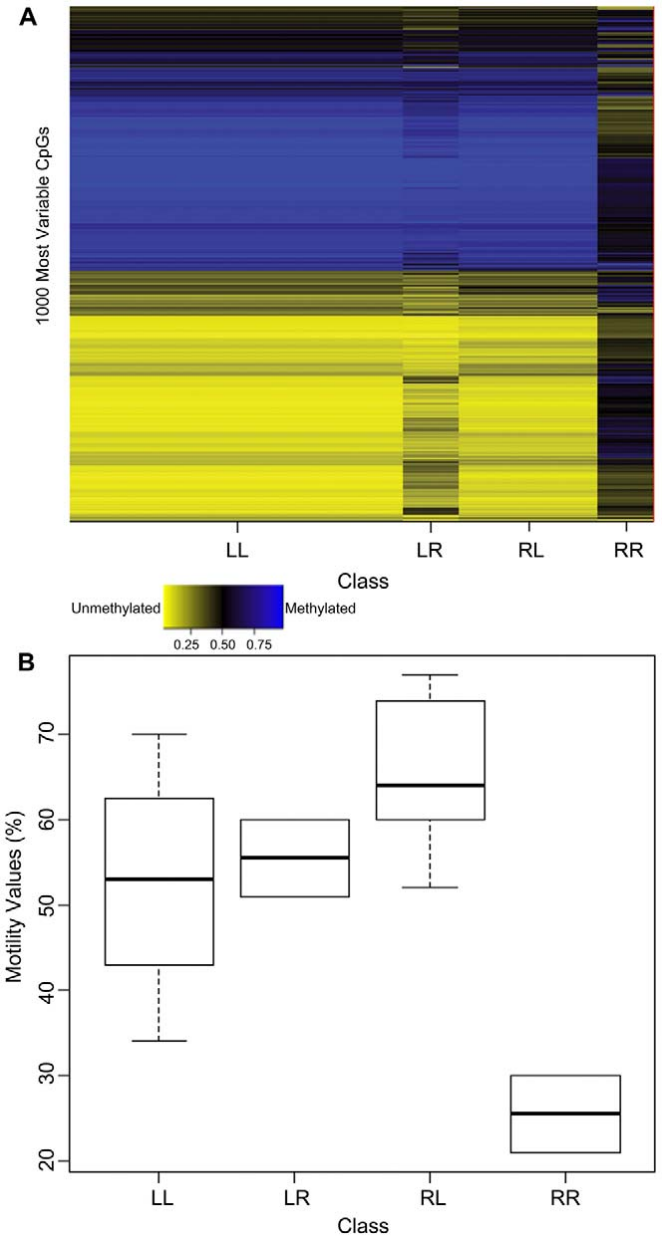
RPMM classes. (A) RPMM displaying 1,000 most variable CpGs by class. Each column represents a class generated by RPMM (left left (LL), left right (LR), right left (RL), and right right (RR)). The width of the column represents the number of patients distributed in that class. The rows represent the average beta values for each CpG within the class. The methylation scale indicates the level of methylation: yellow = sample predominantly unmethylated (0–49% methylated); black = 50% of the sample is methylated; blue = sample predominantly methylated (51–100%).(B) Boxplot comparing the motility values for each class.

Recursively partitioned mixture modeling (RPMM) was performed on raw methylation data to organize the sperm samples into methylation classes based on similarity. The algorithm first separated the 21 sperm profiles into two different branches left (L) and right (R) and then further subdivided each branch into right and left branches resulting in 4 total classes: left left (LL), left right (LR), right left (RL) and right right (RR) (Figure 3, A). In Figure 3 (B) we plotted methylation class-specific sperm motility values: samples in methylation class RR had the lowest median motility, and methylation class was significantly associated with motility after adjusting for multiple comparisons (*P* = 0.01). The association between RPMM methylation class and sperm morphology approached statistical significance (*P* = 0.09), though methylation class was not associated with sperm count (*P* = 0.29).

### Thousands of CpG Loci are Significantly Altered in Low Motility Sperm

Linear models of microarray analysis (LIMMA) was used to univariately test each CpG for association with motility. 9,189 of 27,578 CpGs (34%) had significantly altered methylation associated with motility after adjusting for multiple comparisons (*Q*<0.05) (Table S3). Of these, 1,827 CpGs (20%) were hypermethylated in the low motility samples, whereas 7,362 CpGs (80%) were hypomethylated.

Because establishing proper methylation marks within imprinted genes during spermatogenesis is critical, we next restricted our analysis to CpGs associated with imprinted genes. Of the 616 CpGs associated with imprinted genes, 194 CpGs (31.5%) had significant associations with motility, similar to the distribution of the array overall. Amongst these loci, 47% (n = 92) were hypermethylated in the low motility samples, whereas 53% (n = 102) were hypomethylated. The majority of hypomethylated CpGs were on maternally expressed genes (45%), followed by paternally expressed (33%) and those with undetermined parent of expression (22%). Conversely, the majority of hypermethylated CpGs were associated with paternally expressed genes (70%), with the remainder maternally expressed (26%), and of undetermined parental expression (4%). The 194 loci corresponded to 92 genes, with 11 genes showing both hyper- and hypomethylated loci (Table 2).

**Table 2:** Imprinted Genes with Aberrant DNA Methylation.

Known Imprinted								Predicted Imprinted							
Gene	E	#	MS	Gene	E	#	MS	Gene	E	#	MS	Gene	E	#	MS
*BMPR2*	N	1	−	*MAPK12*	N	2	−	*ALDH1L1*	M	1	−	*LMX1B*	M	1	−
*CCNE1*	N	1	−	MEG3*	M	3	+	*ANKRD11*	M	2	−	*LY6D*	P	2	+
*CD44*	N	2	−	MEST*	P	3	−/+	*APBA1*	P	2	−	*MYEOV2*	P	1	−
*CDKN1C*	M	3	−	*MKRN3*	P	1	+	*BRP44L*	P	1	−	*OTX1*	M	2	−
*COPG2*	P	2	−	*NDN*	P	3	+	*COL9A3*	M	2	−/+	*PEX10*	M	2	−/+
*CTAG2*	N	2	+	*NEDD9*	N	1	−	*DVL1*	M	1	−	*PHPT1*	M	1	−
*CTNND2*	N	1	−	*NGFB*	N	1	−	*EGFL7*	P	2	−	*PPAP2C*	M	2	+
*CYR61*	N	1	−	*NNAT*	P	5	+	*FAM59A*	P	2	−	*PRDM16*	P	1	−
DIRAS3*	P	11	+	*PCNA*	N	2	−	*FAM70B*	M	1	−	*PTPN14*	M	1	−
*DLK1*	P	1	+	*PEG10*	P	8	−/+	*FASTK*	M	1	−	*PURG*	P	2	−
*DLX5*	M	3	−/+	*PHLDA2*	M	4	−	*FOXF1*	M	1	−	*PYY2*	P	2	+
*GABRA5*	P	1	−	PLAGL1*	P	5	+	*GATA3*	P	1	−	*RPL22*	P	1	−
*GFI1*	P	1	−	*SDHD*	P	1	+	*HES1*	P	1	−	*SALL1*	M	1	−
*GRB10*	N	5	−/+	*SGCE*	P	2	+	*HIST3H2BB*	M	1	−	*SLC4A2*	P	2	−
H19*	M	4	−/+	*SHANK2*	M	1	−	*HOXA11*	M	1	−	*SOX8*	P	1	−
*HYMAI*	P	1	−	*SLC22A18*	M	7	−/+	*HOXA5*	M	1	+	*TIGD1*	P	1	−
IGF2*	P	1	−	SNRPN*	P	6	+	*HOXB2*	M	1	−	*TMEM60*	P	2	−
*IGF2AS*	P	4	−	*TCEB3C*	M	3	+	*HOXB3*	M	1	+	*VAX2*	M	1	−
*IL1B*	N	2	−	*TP73*	M	2	−/+	*HOXC4*	M	1	−	*WDR8*	M	1	−
*ILK*	N	2	−	*UBE3A*	M	1	−	*HSPA6*	M	1	+	*ZFP36L2*	M	1	−
*KCNQ1DN*	M	2	+	*WT1*	P	9	−/+	*IFITM1*	M	1	−	*ZNF550*	P	1	−
*L3MBTL*	P	2	+	*ZIM2*	P	5	+	*LDB1*	M	1	−				
*LASS4*	N	2	−	*ZNF264*	M	3	−/+								
*LMO1*	N	1	−	*ZNF331*	P	1	−								
*MAGEL2*	P	3	+												

Note: E = parent with expressed allele; # = number of significantly altered loci in low motility samples; MS = methylation status of the loci; M = maternally expressed; P = paternally expressed; N = parent of origin not determined; − = loci hypomethylated in low motility samples; + = loci hypermethylated in low motility samples; −/+ = more than two loci were altered and some of the loci were hypomethylated and some of were hypermethylated. Genes with * have been previously reported differentially methylated in sperm.

Aberrant promoter methylation in genes related to spermatogenesis and epigenetic regulation have recently been identified in sperm from men with poor semen quality [3]–[13] Thus, we next performed an analysis restricted to array CpGs associated with genes related to spermatogenesis and epigenetic regulation. Of the 147 CpGs on the array associated with genes involved in spermatogenesis, 39% (n = 58) were significantly altered in low motility sperm (similar to the 34% of CpGs associated with low motility in array-wide tests, Table 2). Among these 58 CpG loci, 71% (n = 41) were hypomethylated and 29% (n = 17) were hypermethylated in low motility samples. There were 50 CpG loci associated with epigenetic regulatory genes identified on the array, and only 26% (n = 13) had significantly altered methylation in low motility sperm samples. Of these, 61.5% (n = 8) were hypomethylated and 38.5% (n = 5) were hypermethylated (Table 3).

**Table 3:** Genes Associated with Spermatogenesis and Epigenetic Regulation with Aberrant DNA Methylation.

Gene	#	MS	Gene	#	MS	Gene	#	MS	Gene	#	MS
*ADAM2*	1	+	*CSF1*	2	−	*KIT*	1	−	*SIRT5*	1	−
*ADAMTS2*	1	−	*CTCF*	1	−	*LIMK2*	1	−	*SIRT7*	2	−
*AR*	4	−/+	*CTCFL*	1	+	*MLH1*	2	+	*SLC12A2*	1	−
*ATM*	5	−/+	DAZL*	1	+	*MORC1*	2	+	*SPO11*	2	+
*BCL2*	2	−/+	*DDX4*	1	+	*MSH5*	1	−	*STRBP*	1	−
*BCL2L2*	1	−	*DHH*	1	−	MTHFR*	1	+	*STYX*	1	−
*BCL6*	2	−	*DMC1*	1	−	*PCSK4*	1	−	*SYCP3*	2	+
*BRDT*	1	+	*DNMT3A*	1	−	*PIK3CG*	1	−	*TBPL1*	1	−
*BMP8B*	1	+	*DNMT3B*	1	+	*PMS2*	2	−	*TERT*	1	−
*BSG*	1	−	*EGR4*	2	−/+	*RXRB*	1	−	*TUSC2*	2	−
*CCNA1*	1	−	*HCLS1*	1	−	*SIAH1*	2	−	*VDAC3*	1	+
*CDKN2C*	1	−	*HDAC2*	1	−	*SIRT1*	2	−/+			
*CREM*	2	−	*INPP5B*	1	−	*SIRT2*	1	−			

Note: # = Number of significantly altered loci in low motility samples; MS = methylation status of the loci; − = loci hypomethylated in low motility samples; + = loci hypermethylated in low motility samples; −/+ = more than two loci were altered and some of the loci were hypomethylated and some of were hypermethylated. Genes with * have been previously reported differentially methylated in sperm.

### mRNA Content is Altered in Low Motility Sperm

Focusing on imprinted mRNAs and candidate biallelic mRNAs, LIMMA analysis was performed to identify differentially expressed transcripts, conditioning on motility. Twenty genes were identified as significant after adjusting for false discovery rate (*Q*<0.05) (Table S4). These included 11 imprinted genes (*GLI3* (NCBI 2737), *APAB1* (NCBI 320), *CTNND2* (NCBI 1501), *FERMT2* (NCBI 10979), *PHPT1* (NCBI 29085), *SNRPN* (NCBI 6638), *PPP1R9A* (NCBI 55607), *CDH18* (NCBI 603019), *ALDH1L1* (NCBI 10840), *LDB1* (NCBI 8861), and *PEX10* (NCBI 5192)), six genes associated with spermatogenesis (*SERPINA5* (NCBI 5104), *ACE* (NCBI 1636), *FANCC* (NCBI 2176), *PCSK4* (NCBI 57460), *CYP19A1* (NCBI 1588), and *FAS* (NCBI 355)), and three epigenetic regulatory genes (*HDAC1*, *DNMT3A*, and *SIRT3*). *HDAC1*, *DNMT3A*, *LDB1* and *FAS* showed increased mRNA content in the low motility samples, whereas the remaining 16 showed decreased mRNA content.

### Integration of Epigenetic and Transcript Data

**Figure 4:**
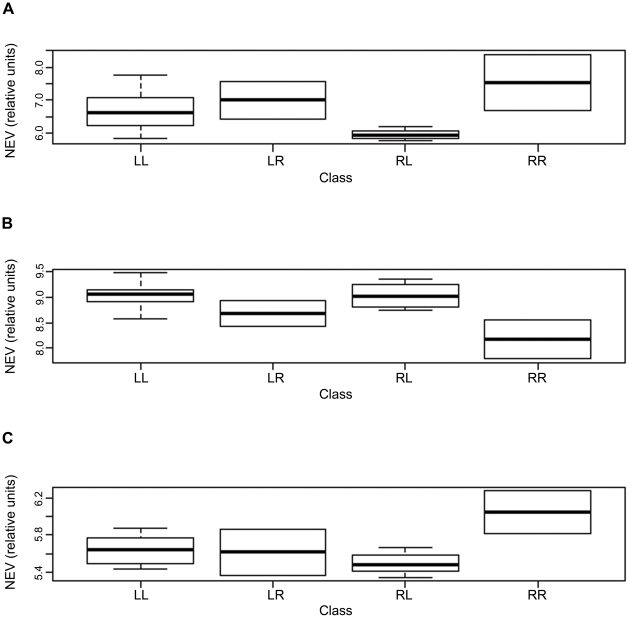
Boxplots comparing DNA methylation profiles and gene expression values for the 3 epigenetic regulators. Each panel of boxplots (A–C) compares expression data for a particular gene: (A) *HDAC1*; (B) *SIRT3*; and (C) *DNMT3A*. The x-axis represents RPMM classes. The y-axis represents normalized gene expression values (NEV).

It is known that major modifications in chromatin organization occur in spermatid nuclei during spermatogenesis, leading to the high degree of packaging in the sperm head. Chromatin compaction ensues when the histones surrounding the DNA are replaced by protamines, and this occurs in parallel with transcriptional arrest [45]. Therefore, nuclear packaging and transcript content are interrelated. To determine whether altered expression of epigenetic regulatory genes was associated with methylation profiles we plotted the methylation class-specific gene expression values for the three epigenetic regulatory genes (*HDAC1*, *SIRT3*, and *DNMT3A*) with significantly altered expression in low motility sperm (Figure 4). Among methylation classes, expression values for *HDAC1*, *SIRT3*, and *DNMT3A* were most altered in class RR, the class with lowest motility sperm (increased expression for *HDAC1* and *DNMT3A*, and decreased expression for *SIRT3*). For all three genes, the association between mRNA expression level and methylation class membership approached significance after adjusting for multiple comparisons (*HDAC1*, *P* = 0.03; *SIRT3*, *P* = 0.06; and *DNMT3A*, *P* = 0.07).

## Discussion

Currently, the evaluation of male infertility relies upon physical exam and semen and hormone analyses; although quick and relatively inexpensive, these physiologic measurements often do not explain the underlying cause of infertility nor predict the usefulness of various therapeutic interventions. Therefore, new approaches are needed to identify the etiologies of male infertility. Recent data suggest that sperm DNA methylation abnormalities and alterations in sperm mRNA content are found in infertile men [3]–[8], [10], [11], [13]–[30], [50]. Here we extend these studies by performing integrative analysis of sperm DNA methylation and mRNA content using genome-wide approaches to identify significant associations among these profiles and semen parameters.

Due to the unreliable nature of classifying men into abnormal and normal groups during a semen analysis, we used a data driven approach to first qualitatively assess associations among sperm DNA methylation and our patient population. Unsupervised clustering indicated that there was an association between DNA methylation and motility status. This was true both for all of the CpGs on the array and the imprint-only subset.

RPMM separated the 21 men into four classes based on similarity of DNA methylation array data. The median motility values were calculated for each class and the results suggested that the methylation profiles were associated with motility. Comparing the DNA methylation heatmap to the class versus motility boxplot indicates that the low motility class has the most aberrantly methylated CpGs. Overall, these data suggest that low motility sperm have increased hypomethylation relative to high motility sperm. We used LIMMA to identify the significantly altered CpGs conditioned on changes in motility for all CpGs on the array: over one-third of the CpGs (and almost half of the genes represented on the array) were significantly differentially methylated in the low motility samples and the majority of these were hypomethylated. The high prevalence of aberrantly methylated CpGs suggests a genome-wide DNA methylation defect in the low motility sperm. It has been previously hypothesized that the aberrant sperm DNA methylation could be due to abnormal chromatin compaction, inefficient DNA methyltransferases, and/or failure to maintain or acquire the correct methylation marks during spermatogenesis and our results are consistent with this literature [11]–[13], [29].

We initially focused on CpGs mapping to imprinted genes because of their plasticity during spermatogenesis, biological relevance following conception and development, and because previous studies have identified imprinted loci as aberrantly methylated in abnormal sperm [3]–[8]. In our data, the distribution of significantly hyper- and hypomethylated imprinted loci was nearly equal. Expanding the imprinting analysis to the gene level identified 92 genes with altered CpG methylation, seven of which (*DIRAS3* (NCBI 9077), *H19* (NCBI 283120), *IGF2* (NCBI 3481), *MEST/PEG1* (NCBI 4232), *PLAGL1/ZAC* (NCBI 5325), *MEG3/GTL2* (NCBI 55384), and *SNRPN*) have already been noted as aberrantly methylated in abnormal sperm [3]–[8], [10], [11]. The methylation status of two genes, (*PEG3* (NCBI 5178) and *LIT1/KCNQ1OT1* (NCBI 10984)) has been inconsistently reported in the literature [3], [8], [10]. We observed no statistical differences for these genes between the low and high motility sperm, which is consistent with the results published by Sato, et al. [10]. In fact, our study confirmed all of the DNA methylation results reported in the aforementioned study.

To further clarify the potential functional alterations to imprinted genes and critical epigenetic regulatory genes, we evaluated sperm mRNA content of 177 imprinted genes and 99 other transcripts where an *a priori* hypothesis for association with male subfertility or epigenetic regulation exists. Twenty genes were identified as demonstrating significantly altered transcript levels in low motility sperm. All of the mRNAs except *HDAC1*, *DNMT3A*, *LBD1*, and *FAS* were present in decreased amounts in low motility sperm, and we did not observe altered mRNA content for *BRDT*, which was previously reported to have increased expression in subfertile patients [29].

Integration of epigenetic and expression data revealed a relationship between transcript content of three epigenetic regulatory genes (*HDAC1*, *SIRT3*, and *DNMT3A*) and methylation class. *HDAC1* is the predominant histone deacetylase (HDAC) during spermatogenesis. Histone hyperacetlyation is required for the histone to protamine exchange and is facilitated by the degradation of HDAC1 in elongated spermatids [51]. If HDAC1 is in excess, one could hypothesize that the histones are not being replaced by protamines, leading to an “immature” sperm chromatin structure, with less compact DNA. Therefore, incomplete or incorrect nuclear compaction may influence overall sperm maturation and be reflected in the physiological endpoint of motility.

*SIRT3* is a class III histone deacetylase and this HDAC family is similar to the yeast Sir2 protein which has been associated with chromatin silencing and also plays roles in cellular metabolism and aging [46]. In mammals, however, SIRT3 is targeted to the mitochondria and functions to induce the expression of the antioxidant MnSOD to eliminate reactive oxygen species (ROS) generated during oxidative phosphorylation [52]. Recent studies have found that increased ROS in sperm have deleterious effects on sperm motility parameters which ultimately have adverse effects on fertility [53]. Therefore, the decrease in *SIRT3* mRNA in the low motility sperm may reflect reduced MnSOD and increased intracellular ROS during spermatogenesis, leading to a diminished fertility potential.

The literature also suggests that oxidative stress itself can impede the process of DNA methylation, resulting in a hypomethylated phenotype [54]. Interestingly, we observed global hypomethylation in the low motility sperm even though we saw increased DNMT3A transcript presence in the low motility sperm. Because DNMT3A is the DNA methyltransferase responsible for *de novo* methylation, our data suggests a failure of the low motility sperm to acquire the proper methylation patterns.

Although we were limited by sample size, we used a powerful integrative approach to simultaneously examine sperm DNA methylation and mRNA content utilizing two high density array techniques. We found that: (1) low motility sperm have genome-wide DNA hypomethylation that may be due to a failure of the sperm to complete chromatin compaction properly because of increased *HDAC1* presence; (2) low motility sperm have reduced *SIRT3* mRNA content which might be related to increased subcellular ROS during spermatogenesis leading to the abnormal motility phenotype; and (3) this oxidative stress may be impeding the ability of DNMT3A to set the correct methylation marks which would also contribute to the hypomethylated phenotype. Our results suggest that additional integrative studies including larger sample sizes as well as prospective studies of fertility following these integrated molecular assessments have great potential to advance our understanding of the molecular features of sperm associated with fertility status.

## Supporting Information

Table S1 **Imprinted Genes.**
(DOC)

Table S2 **Genes Associated with Spermatogenesis and Epigenetic Regulation.**
(DOC)

Table S3 **Aberrant CpGs in Low Motility Sperm.**
(DOC)

Table S4 **Aberrant mRNA Transcripts in Low Motility Sperm.**
(DOC)
