# Supplementary material for: Integrative DNA Methylation and Gene Expression Analyses Identify DNA Packaging and Epigenetic Regulatory Genes Associated with Low Motility Sperm
Source: PLoS One. 2011 Jun 2;6(6):e20280. doi: 10.1371/journal.pone.0020280 (PMC3107223; doi:10.1371/journal.pone.0020280)
Supplement: Table S1 — Imprinted Genes. (DOC) [file pone.0020280.s001.doc]

| **Table S1: Imprinted Genes** | | | | | | | | | | | |
| --- | --- | --- | --- | --- | --- | --- | --- | --- | --- | --- | --- |
| **Experimentally Tested** | | | | | | **Computationally Predicted** | | | | | |
| **Gene** | **Exp** | **NCBI** | **Gene** | **Exp** | **NCBI** | **Gene** | **Exp** | **NCBI** | **Gene** | **Exp** | **NCBI** |
| *ATP10A* | M | 57194 | *LASS4* | ND | 79603 | *ABCC9* | M | 10060 | *HOXC9* | M | 3225 |
| *BMPR2* | ND | 659 | *LMO1* | ND | 4004 | *ABCG8* | M | 64241 | *HSPA6* | M | 3310 |
| *CCDC86* | ND | 79080 | *MAGEL2* | P | 54551 | *ACD* | M | 65057 | *IFITM1* | M | 8519 |
| *CCNE1* | ND | 898 | *MAGI2* | M | 9863 | *ADAMTS16* | M | 170690 | *KBTBD3* | P | 143879 |
| *CD44* | ND | 960 | *MAPK12* | ND | 6300 | *ALDH1L1* | M | 10840 | *LDB1* | M | 8861 |
| *CDKAL1* | ND | 54901 | *MEG3* | M | 55384 | *ANKRD11* | M | 29123 | *LILRB4* | M | 11006 |
| *CDKN1C* | M | 1028 | *MEST* | P | 4232 | *APBA1* | P | 320 | *LMX1B* | M | 4010 |
| *COPG2* | P | 26958 | *MESTIT1** | P | 317751 | *B4GALNT4* | M | 338707 | *LY6D* | P | 8581 |
| *COPG2IT1** | P | 53844 | *MKRN3* | P | 7681 | *BMP8* | P | 656 | *MYEOV2* | P | 150678 |
| *CPA4* | M | 51200 | *NDN* | P | 4692 | *BRP44L* | P | 51660 | *MZF1* | M | 7593 |
| *CTAG2* | ND | 30848 | *NEDD9* | ND | 4739 | *BRUNOL4* | M | 56853 | *NDUFA4* | P | 4697 |
| *CTNNA3* | M | 29119 | *NGFB* | ND | 4803 | *BTNL2* | M | 56244 | *NKAIN3* | P | 286183 |
| *CTNND2* | ND | 1501 | *NLRP2* | ND | 55655 | *CCBL2* | M | 56267 | *NKX6-2* | M | 84504 |
| *CYR61* | ND | 3491 | *NNAT* | P | 4826 | *CCDC85A* | P | 114800 | *OBSCN* | P | 84033 |
| *DHCR24* | ND | 1718 | *OR11L1* | P | 391189 | *CDH18* | P | 1016 | *OSBPL1A* | ND | 114876 |
| *DIRAS3* | P | 9077 | *OSBPL5* | M | 114879 | *CDK4* | M | 1019 | *OTX1* | M | 5013 |
| *DLGAP2* | P | 9228 | *PCNA* | ND | 5111 | *CHMP2A* | M | 27243 | *PAOX* | M | 196743 |
| *DLK1* | P | 8788 | *PEG3* | P | 5178 | *CHST8* | M | 64377 | *PEX10* | M | 5192 |
| *DLX5* | M | 1749 | *PEG10* | P | 23089 | *COL9A3* | M | 1299 | *PHPT1* | M | 29085 |
| *DOK7* | ND | 285489 | *PHLDA2* | M | 7262 | *CSF2* | M | 1437 | *PKP3* | M | 11187 |
| *E2F7* | M | 144455 | *PLAGL1* | P | 5325 | *CYP1B1* | P | 1545 | *PPAP2C* | M | 8612 |
| *EPS15* | ND | 2060 | *PPP1R9A* | M | 55607 | *DGCR6* | P | 8214 | *PRDM16* | P | 63976 |
| *GABRA5* | P | 2558 | *SDHD* | P | 6392 | *DUX2** | P | 26583 | *PTPN14* | M | 5784 |
| *GABRB3* | P | 2562 | *SGCE* | P | 8910 | *DVL1* | M | 1855 | *PURG* | P | 29942 |
| *GABRG3* | P | 2567 | *SHANK2* | M | 22941 | *EGFL7* | P | 51162 | *PYY2* | P | 23615 |
| *GDNF* | P | 2668 | *SLC22A18* | M | 5002 | *EVX1* | P | 2128 | *RAB1B* | M | 81876 |
| *GFI1* | P | 2672 | *SLC22A18AS* | M | 5003 | *FAM50B* | M | 26240 | *RBP5* | P | 83758 |
| *GNAS* | M | 2778 | *SLC22A3* | P | 6581 | *FAM59A* | P | 64762 | *RPL22* | P | 6146 |
| *GRB10* | ND | 2887 | *SNORD64* | P | 347686 | *FAM70B* | M | 348013 | *RTL1* | M | 388015 |
| *GRIA1* | ND | 2890 | *SNORD107** | P | 91380 | *FAM132A* | M | 388581 | *SALL1* | M | 6299 |
| *H19* | M | 283120 | *SNORD108** | P | 338427 | *FAM174A* | P | 345757 | *SIM2* | P | 6493 |
| *HTR2A* | M | 3356 | *SNORD109B** | P | 338429 | *FASTK* | M | 10922 | *SLC22A2* | P | 6582 |
| *HYMAI** | P | 57061 | *SNORD116@** | P | 692236 | *FERMT2* | P | 10979 | *SLC26A10* | M | 65012 |
| *IGF2* | P | 3481 | *SNRPN* | P | 6638 | *FGFRL1* | M | 53834 | *SLC4A2* | M | 6522 |
| *IGF2AS* | P | 51214 | *SNURF* | P | 8926 | *FOXF1* | M | 2294 | *SOX8* | P | 30812 |
| *IL1B* | ND | 3553 | *TCEB3C* | M | 162699 | *FOXG1* | P | 2290 | *SPON2* | P | 10417 |
| *ILK* | ND | 3611 | *TFPI2* | M | 7980 | *FUCA1* | P | 2517 | *TIGD1* | P | 200765 |
| *INS** | P | 3630 | *TP73* | M | 7161 | *GATA3* | P | 2625 | *TMEM52* | P | 339456 |
| *KCNK9* | M | 51305 | *UBE3A* | M | 7337 | *GLI3* | M | 2737 | *TMEM60* | P | 85025 |
| *KCNQ1* | M | 3784 | *WT1* | P | 7490 | *GPT* | M | 2875 | *TMEM88* | M | 92162 |
| *KCNQ1DN* | M | 55539 | *ZIM2* | P | 23619 | *HES1* | P | 3280 | *TSHZ3* | P | 57616 |
| *KCNQ1OT1** | P | 10984 | *ZNF264* | M | 9422 | *HIST3H2BB* | M | 128312 | *VAX2* | M | 25806 |
| *KLF14* | M | 136259 | *ZNF331* | P | 55422 | *HOXA2* | M | 3199 | *VENTX2* | M | 27287 |
| *L3MBTL* | P | 26013 |  |  |  | *HOXA3* | M | 3200 | *WDR8* | M | 49856 |
|  |  |  |  |  |  | *HOXA4* | M | 3201 | *ZFP36L2* | M | 678 |
|  |  |  |  |  |  | *HOXA5* | M | 3202 | *ZIC1* | M | 7545 |
|  |  |  |  |  |  | *HOXA11* | M | 3207 | *ZNF225* | P | 7768 |
|  |  |  |  |  |  | *HOXB2* | M | 3212 | *ZNF229* | M | 7772 |
|  |  |  |  |  |  | *HOXB3* | M | 3213 | *ZNF550* | M | 162972 |
|  |  |  |  |  |  | *HOXC4* | M | 3221 | *ZNF738* | P | 148203 |
| Note: Exp = parent with expressed allele; P = paternally expressed; M = maternally expressed; ND = parent of origin not determined; Genes with * were excluded from the LIMMA mRNA analysis because they were not on the Affymetrix array. | | | | | | | | | | | |
