# Supplementary material for: Integrative DNA Methylation and Gene Expression Analyses Identify DNA Packaging and Epigenetic Regulatory Genes Associated with Low Motility Sperm
Source: PLoS One. 2011 Jun 2;6(6):e20280. doi: 10.1371/journal.pone.0020280 (PMC3107223; doi:10.1371/journal.pone.0020280)
Supplement: Table S2 — Genes Associated with Spermatogenesis and Epigenetic Regulation. (DOC) [file pone.0020280.s002.doc]

| **Table S2: Genes Associated with Spermatogenesis and Epigenetic Regulation** | | | | | | | |
| --- | --- | --- | --- | --- | --- | --- | --- |
| **Gene** | **NCBI** | **Gene** | **NCBI** | **Gene** | **NCBI** | **Gene** | **NCBI** |
| *ACE* | 1636 | *CTCF* | 10664 | *HDAC8* | 55869 | *RXRB* | 6257 |
| *ADAM2* | 2515 | *CTCFL* | 14069 | *HMGB2* | 3148 | *SERPINA5* | 5104 |
| *ADAM3A** | 1587 | *CYP19A1* | 1588 | *HSPA1B* | 3304 | *SIAH1* | 6477 |
| *ADAMTS2* | 9509 | *DAZL* | 1618 | *INHA* | 3623 | *SIRT1* | 23411 |
| *AGFG1** | 3267 | *DDX4* | 54514 | *INPP5B* | 3633 | *SIRT2* | 22933 |
| *APAF1* | 317 | *DHH* | 50846 | *KIT* | 3815 | *SIRT3* | 23410 |
| *APOB* | 338 | *DMC1* | 11144 | *KITLG* | 4254 | *SIRT4* | 23409 |
| *AR* | 367 | *DMRT1* | 1761 | *LHCGR* | 3973 | *SIRT5* | 23408 |
| *ATM* | 472 | *DNAH1** | 25981 | *LIMK2* | 3985 | *SIRT6* | 51548 |
| *BAX* | 581 | *DNMT1* | 1786 | *MAN2A2* | 4122 | *SIRT7* | 51547 |
| *BCL2* | 596 | *DNMT3A* | 1788 | *MLH1* | 4292 | *SLC12A2* | 6558 |
| *BCL2L2* | 599 | *DNMT3B* | 1789 | *MORC1* | 27136 | *SPO11* | 23626 |
| *BCL6* | 604 | *DNMT3L* | 29947 | *MSH5* | 4439 | *STRBP* | 55342 |
| *BMP8B* | 656 | *EGR4* | 1961 | *MTHFR* | 4524 | *STYX* | 6815 |
| *BDRT* | 676 | *EMHT1** | 79813 | *MYBL1* | 4603 | *SYCP3* | 50511 |
| *BSG* | 682 | *FANCA** | 2157 | *NCOA1* | 8648 | *TBPL1* | 9519 |
| *CAMK4* | 814 | *FANCC** | 2176 | *NCOA3* | 8202 | *TERT* | 7015 |
| *CCNA1* | 8900 | *FANCG** | 2189 | *PCSK4* | 54760 | *THEG* | 51298 |
| *CDKN2C* | 1031 | *FAS* | 355 | *PIK3CG* | 5294 | *TNP1* | 7141 |
| *CLDN11* | 5010 | *GJA1* | 2697 | *PMS2* | 5395 | *TNP2* | 7142 |
| *CLGN* | 1047 | *HAT1* | 8520 | *PPP1CC* | 5510 | *TRDMT1** | 1787 |
| *CPEB1** | 64506 | *HCLS1* | 3059 | *PRM1* | 5620 | *TUSC2* | 11334 |
| *CREM* | 1380 | *HDAC1* | 3065 | *PRM2** | 5619 | *UBE2B* | 7320 |
| *CSF1* | 1435 | *HDAC2* | 3066 | *PRM3** | 58531 | *VDAC3* | 7419 |
| *CSNK2A2* | 1459 | *HDAC3* | 8841 | *ROS1* | 6098 |  |  |
| Note: Genes with * were not on the Illumina Infinium Array. | | | | | | | |
