# Supplementary material for: Integrative DNA Methylation and Gene Expression Analyses Identify DNA Packaging and Epigenetic Regulatory Genes Associated with Low Motility Sperm
Source: PLoS One. 2011 Jun 2;6(6):e20280. doi: 10.1371/journal.pone.0020280 (PMC3107223; doi:10.1371/journal.pone.0020280)
Supplement: Table S3 — Aberrant CpGs in Low Motility Sperm. (DOC) [file pone.0020280.s003.doc]

| **Table S3: Aberrant CpGs in Low Motility Sperm** | | | | |
| --- | --- | --- | --- | --- |
| **Illumina CpG ID** | **Gene** | **NCBI** | **Slope (log_2_)** | **Q-value** |
| cg00006414 | *ZNF398* | 57541 | 0.18 | 0.03633 |
| cg00008713 | *IMPA2* | 3613 | 0.11 | 0.03633 |
| cg00025138 | *MAP3K9* | 4293 | 0.17 | 0.03633 |
| cg00042156 | *MGC16291* | 84856 | -0.70 | 0.03633 |
| cg00072216 | *MXD4* | 10608 | 0.13 | 0.03633 |
| cg00078194 | *MDK* | 4192 | 0.13 | 0.03633 |
| cg00107632 | *PGM2* | 55276 | 0.11 | 0.03633 |
| cg00117172 | *RUNX3* | 864 | 0.15 | 0.03633 |
| cg00128197 | *HADH2* | 3028 | 0.17 | 0.03633 |
| cg00141162 | *HCLS1* | 3059 | 0.82 | 0.03633 |
| cg00143376 | *NMUR1* | 10316 | -0.68 | 0.03633 |
| cg00155485 | *THRAP2* | 23389 | 0.17 | 0.03633 |
| cg00168942 | *CX40.1* | 219770 | 0.36 | 0.03633 |
| cg00184893 | *ALOXE3* | 59344 | 0.12 | 0.03633 |
| cg00223950 | *RBAF600* | 23352 | 0.10 | 0.03633 |
| cg00228799 | *ENDOG* | 2021 | 0.12 | 0.03633 |
| cg00261552 | *CREBL2* | 1389 | 0.15 | 0.03633 |
| cg00269115 | *LOC148898* | 148898 | 0.10 | 0.03633 |
| cg00273124 | *NOLA2* | 55651 | -0.74 | 0.03633 |
| cg00282683 | *UBE3C* | 9690 | 0.12 | 0.03633 |
| cg00294382 | *IL23A* | 51561 | 0.57 | 0.03633 |
| cg00302793 | *PPP3CA* | 5530 | 0.12 | 0.03633 |
| cg00311768 | *TSTA3* | 7264 | -0.42 | 0.03633 |
| cg00320243 | *RAB3A* | 5864 | 0.10 | 0.03633 |
| cg00321065 | *RLF* | 6018 | 0.14 | 0.03633 |
| cg00338893 | *MRPS33* | 51650 | 0.16 | 0.03633 |
| cg00405070 | *C12orf8* | 10961 | 0.15 | 0.03633 |
| cg00459975 | *HMBOX1* | 79618 | 0.11 | 0.03633 |
| cg00462311 | *NFATC4* | 4776 | 0.22 | 0.03633 |
| cg00463577 | *C6orf150* | 115004 | 0.15 | 0.03633 |
| cg00465846 | *RNF139* | 11236 | 0.12 | 0.03633 |
| cg00480209 | *C17orf48* | 56985 | 0.09 | 0.03633 |
| cg00480356 | *HYPK* | 25764 | 0.12 | 0.03633 |
| cg00485296 | *NME3* | 4832 | 0.11 | 0.03633 |
| cg00487159 | *SOCS1* | 8651 | 0.08 | 0.03633 |
| cg00491839 | *PPP1R15A* | 23645 | 0.10 | 0.03633 |
| cg00537910 | *ABCB6* | 10058 | -0.85 | 0.03633 |
| cg00545573 | *TAOK3* | 51347 | 0.18 | 0.03633 |
| cg00585790 | *LIMS1* | 3987 | 0.42 | 0.03633 |
| cg00601486 | *H1T2* | 341567 | -0.57 | 0.03633 |
| cg00605270 | *SMPDL3B* | 27293 | 0.76 | 0.03633 |
| cg00629585 | *GFI1B* | 8328 | 0.24 | 0.03633 |
| cg00634577 | *HIST1H4K* | 8362 | 0.13 | 0.03633 |
| cg00648883 | *LOC196549* | 196549 | 0.51 | 0.03633 |
| cg00661485 | *FOXI1* | 2299 | 0.16 | 0.03633 |
| cg00669856 | *SPRY1* | 10252 | 0.16 | 0.03633 |
| cg00686623 | *CAPN1* | 823 | 0.12 | 0.03633 |
| cg00714377 | *SLA2* | 84174 | 0.60 | 0.03633 |
| cg00717862 | *DNAJB11* | 51726 | 0.18 | 0.03633 |
| cg00720723 | *NOP5/NOP58* | 51602 | 0.12 | 0.03633 |
| cg00725635 | *B3GALT2* | 8707 | 0.46 | 0.03633 |
| cg00751288 | *LOC90624* | 90624 | 0.12 | 0.03633 |
| cg00769520 | *ST8SIA1* | 6489 | 0.12 | 0.03633 |
| cg00778920 | *PRPF19* | 27339 | 0.15 | 0.03633 |
| cg00783759 | *LARP2* | 55132 | 0.14 | 0.03633 |
| cg00804048 | *EIF5A* | 1984 | 0.16 | 0.03633 |
| cg00815440 | *UQCRFS1* | 7386 | 0.12 | 0.03633 |
| cg00832517 | *C14orf126* | 112487 | 0.24 | 0.03633 |
| cg00840403 | *CHST4* | 10164 | 0.47 | 0.03633 |
| cg00840516 | *HYAL2* | 8692 | -0.71 | 0.03633 |
| cg00877887 | *LOC405753* | 405753 | 0.10 | 0.03633 |
| cg00884221 | *IGFBP7* | 3490 | 0.20 | 0.03633 |
| cg00893987 | *RPL7L1* | 285855 | 0.13 | 0.03633 |
| cg00899086 | *SEPT11* | 55752 | 0.15 | 0.03633 |
| cg00905170 | *RRS1* | 23212 | 0.11 | 0.03633 |
| cg00908551 | *FANCF* | 2188 | 0.08 | 0.03633 |
| cg00916199 | *PRO1580* | 55374 | 0.14 | 0.03633 |
| cg00917893 | *PDHB* | 5162 | 0.12 | 0.03633 |
| cg00918794 | *PPIB* | 5479 | 0.16 | 0.03633 |
| cg00954003 | *TMSB10* | 9168 | 0.14 | 0.03633 |
| cg00962459 | *PROKR1* | 10887 | -0.49 | 0.03633 |
| cg00964109 | *UBL7* | 84993 | 0.12 | 0.03633 |
| cg00997424 | *PI4KII* | 55361 | 0.19 | 0.03633 |
| cg01001286 | *IL16* | 3603 | 0.43 | 0.03633 |
| cg01015879 | *RDX* | 5962 | 0.15 | 0.03633 |
| cg01029592 | *SOX15* | 6665 | -0.59 | 0.03633 |
| cg01036173 | *C9orf46* | 55848 | 0.12 | 0.03633 |
| cg01048931 | *ECOP* | 81552 | 0.11 | 0.03633 |
| cg01069256 | *C8orf70* | 51101 | 0.10 | 0.03633 |
| cg01078276 | *PLAU* | 5328 | 0.11 | 0.03633 |
| cg01107031 | *TGFB1* | 7040 | 0.11 | 0.03633 |
| cg01107741 | *CANT1* | 124583 | 0.13 | 0.03633 |
| cg01120898 | *IL1RAP* | 3556 | 0.12 | 0.03633 |
| cg01139966 | *GOLPH3L* | 55204 | 0.11 | 0.03633 |
| cg01140782 | *FAM58A* | 92002 | 0.16 | 0.03633 |
| cg01176271 | *SNX1* | 6642 | 0.16 | 0.03633 |
| cg01244871 | *FAM82B* | 51115 | 0.11 | 0.03633 |
| cg01254505 | *BST2* | 684 | 0.82 | 0.03633 |
| cg01269795 | *BTN3A3* | 10384 | 0.60 | 0.03633 |
| cg01272601 | *AAK1* | 22848 | 0.15 | 0.03633 |
| cg01278291 | *DOK3* | 79930 | 0.74 | 0.03633 |
| cg01306510 | *CDKN1A* | 1026 | 0.15 | 0.03633 |
| cg01340005 | *SOX2* | 6657 | 0.11 | 0.03633 |
| cg01346589 | *RCC2* | 55920 | 0.12 | 0.03633 |
| cg01357263 | *BCL7A* | 605 | 0.11 | 0.03633 |
| cg01371258 | *ZNF184* | 7738 | 0.12 | 0.03633 |
| cg01372605 | *C12orf62* | 84987 | 0.16 | 0.03633 |
| cg01374047 | *HCFC1R1* | 54985 | 0.13 | 0.03633 |
| cg01399317 | *FLJ46072* | 286077 | -0.71 | 0.03633 |
| cg01401337 | *PHF2* | 5253 | 0.11 | 0.03633 |
| cg01413314 | *IFRD2* | 7866 | 0.12 | 0.03633 |
| cg01420388 | *FBXO2* | 26232 | -0.79 | 0.03633 |
| cg01422136 | *ZCCHC10* | 54819 | 0.12 | 0.03633 |
| cg01422337 | *CARD8* | 22900 | 0.14 | 0.03633 |
| cg01428361 | *RSAD1* | 55316 | 0.15 | 0.03633 |
| cg01429181 | *WDR42A* | 50717 | 0.15 | 0.03633 |
| cg01447817 | *AQP12A* | 375318 | 0.71 | 0.03633 |
| cg01454004 | *SLC17A5* | 26503 | 0.14 | 0.03633 |
| cg01485645 | *MLLT6* | 4302 | 0.19 | 0.03633 |
| cg01493303 | *GRWD1* | 83743 | 0.11 | 0.03633 |
| cg01497501 | *ZFAND2A* | 90637 | 0.13 | 0.03633 |
| cg01533387 | *IFI30* | 10437 | 0.18 | 0.03633 |
| cg01555431 | *AKAP12* | 9590 | 0.13 | 0.03633 |
| cg01557297 | *SLC22A17* | 51310 | 0.10 | 0.03633 |
| cg01557989 | *SLC24A3* | 57419 | 0.12 | 0.03633 |
| cg01565918 | *GNAS* | 2778 | -0.78 | 0.03633 |
| cg01578324 | *CLIC1* | 1192 | 0.83 | 0.03633 |
| cg01614759 | *ZNF22* | 7570 | -0.55 | 0.03633 |
| cg01620569 | *AK2* | 204 | 0.12 | 0.03633 |
| cg01636591 | *CCL8* | 6355 | 0.37 | 0.03633 |
| cg01653110 | *VAMP4* | 8674 | 0.18 | 0.03633 |
| cg01668465 | *SCG2* | 7857 | 0.14 | 0.03633 |
| cg01685739 | *SLC4A2* | 6522 | 0.13 | 0.03633 |
| cg01712016 | *HDAC11* | 79885 | 0.12 | 0.03633 |
| cg01738944 | *SLC35E2* | 9906 | 0.14 | 0.03633 |
| cg01775265 | *RP11-49G10.8* | 317716 | 0.22 | 0.03633 |
| cg01780482 | *EPAS1* | 2034 | 0.15 | 0.03633 |
| cg01782320 | *YME1L1* | 10730 | 0.15 | 0.03633 |
| cg01782487 | *ERMAP* | 114625 | 0.10 | 0.03633 |
| cg01797043 | *RPL3L* | 6123 | 0.20 | 0.03633 |
| cg01798443 | *RPSA* | 3921 | 0.13 | 0.03633 |
| cg01802635 | *TXNDC5* | 81567 | 0.13 | 0.03633 |
| cg01802653 | *HERC3* | 8916 | 0.12 | 0.03633 |
| cg01803059 | *SMAD1* | 4086 | 0.20 | 0.03633 |
| cg01808090 | *NUBP1* | 4682 | 0.14 | 0.03633 |
| cg01813965 | *C16orf50* | 84229 | 0.72 | 0.03633 |
| cg01837689 | *P15RS* | 55197 | 0.13 | 0.03633 |
| cg01838317 | *GCH1* | 2643 | 0.14 | 0.03633 |
| cg01839464 | *DCC* | 1630 | 0.12 | 0.03633 |
| cg01846065 | *STAM2* | 10254 | 0.14 | 0.03633 |
| cg01848594 | *C16orf61* | 56942 | 0.20 | 0.03633 |
| cg01858173 | *ROM1* | 6094 | 0.13 | 0.03633 |
| cg01885202 | *CDKL5* | 6792 | 0.12 | 0.03633 |
| cg01909921 | *GFI1B* | 8328 | 0.29 | 0.03633 |
| cg01914242 | *ATP2A3* | 489 | 0.11 | 0.03633 |
| cg01924619 | *B4GALT5* | 9334 | 0.13 | 0.03633 |
| cg01932459 | *CD164* | 8763 | 0.10 | 0.03633 |
| cg01940867 | *TMEM8* | 58986 | 0.16 | 0.03633 |
| cg01951308 | *KIF3B* | 9371 | 0.14 | 0.03633 |
| cg01965939 | *SH3TC2* | 79628 | 0.77 | 0.03633 |
| cg01968530 | *C18orf45* | 85019 | 0.17 | 0.03633 |
| cg01980222 | *TREM2* | 54209 | 0.42 | 0.03633 |
| cg01994513 | *BLM* | 641 | 0.13 | 0.03633 |
| cg02004156 | *TFE3* | 7030 | 0.14 | 0.03633 |
| cg02008154 | *TBX20* | 57057 | 0.14 | 0.03633 |
| cg02017155 | *FFAR3* | 2865 | 0.68 | 0.03633 |
| cg02029926 | *CXCL1* | 2919 | 0.15 | 0.03633 |
| cg02040734 | *C10orf95* | 79946 | 0.20 | 0.03633 |
| cg02058918 | *GABBR2* | 9568 | 0.13 | 0.03633 |
| cg02090283 | *CNTNAP3* | 79937 | 0.39 | 0.03633 |
| cg02097636 | *PAFAH1B3* | 5050 | 0.12 | 0.03633 |
| cg02102644 | *AP1GBP1* | 11276 | 0.13 | 0.03633 |
| cg02157083 | *APOA5* | 116519 | -0.25 | 0.03633 |
| cg02163937 | *FAM53B* | 9679 | 0.17 | 0.03633 |
| cg02169446 | *MYO9A* | 4649 | 0.15 | 0.03633 |
| cg02181506 | *SERPINA1* | 5265 | 0.64 | 0.03633 |
| cg02218260 | *GRM2* | 2912 | -0.31 | 0.03633 |
| cg02226871 | *VPS28* | 51160 | 0.13 | 0.03633 |
| cg02238747 | *KIAA0690* | 23223 | 0.14 | 0.03633 |
| cg02238826 | *B3GNT5* | 84002 | 0.09 | 0.03633 |
| cg02255004 | *GDEP* | 118425 | 0.28 | 0.03633 |
| cg02273078 | *USP14* | 9097 | 0.09 | 0.03633 |
| cg02273392 | *SERPINE1* | 5054 | 0.72 | 0.03633 |
| cg02326006 | *ARHGEF9* | 23229 | 0.15 | 0.03633 |
| cg02332073 | *TSGA13* | 114960 | 0.52 | 0.03633 |
| cg02332537 | *RTP3* | 83597 | 0.20 | 0.03633 |
| cg02337166 | *NR1D1* | 9572 | 0.12 | 0.03633 |
| cg02342494 | *MGC50559* | 254013 | 0.09 | 0.03633 |
| cg02357714 | *DOK3* | 79930 | 0.70 | 0.03633 |
| cg02360616 | *C1orf24* | 116496 | 0.15 | 0.03633 |
| cg02360776 | *ZBTB3* | 79842 | 0.14 | 0.03633 |
| cg02364038 | *PTBP1* | 5725 | 0.15 | 0.03633 |
| cg02372712 | *ZNF467* | 168544 | 0.26 | 0.03633 |
| cg02376703 | *EPHA1* | 2041 | 0.16 | 0.03633 |
| cg02387854 | *ANAPC11* | 51529 | 0.09 | 0.03633 |
| cg02391387 | *IMMP2L* | 83943 | 0.17 | 0.03633 |
| cg02399449 | *CLDN12* | 9069 | 0.15 | 0.03633 |
| cg02420102 | *MGC33948* | 160140 | 0.17 | 0.03633 |
| cg02421543 | *DR1* | 1810 | 0.16 | 0.03633 |
| cg02426410 | *AASDH* | 132949 | 0.09 | 0.03633 |
| cg02428100 | *KEAP1* | 9817 | 0.15 | 0.03633 |
| cg02432101 | *TCEB3C* | 162699 | -0.49 | 0.03633 |
| cg02441831 | *SYVN1* | 84447 | 0.22 | 0.03633 |
| cg02447314 | *POLI* | 11201 | 0.11 | 0.03633 |
| cg02449978 | *CDC25A* | 993 | 0.11 | 0.03633 |
| cg02460349 | *RRBP1* | 6238 | 0.12 | 0.03633 |
| cg02491878 | *ATP6V0A4* | 50617 | 0.39 | 0.03633 |
| cg02497175 | *NLK* | 51701 | 0.15 | 0.03633 |
| cg02505409 | *ANGPTL4* | 51129 | 0.15 | 0.03633 |
| cg02505689 | *DBF4B* | 80174 | 0.11 | 0.03633 |
| cg02506728 | *PPP1R3D* | 5509 | 0.11 | 0.03633 |
| cg02507175 | *PKNOX1* | 5316 | 0.09 | 0.03633 |
| cg02513045 | *C21orf2* | 755 | 0.12 | 0.03633 |
| cg02539714 | *RHOF* | 54509 | 0.13 | 0.03633 |
| cg02545192 | *TERT* | 7015 | 0.14 | 0.03633 |
| cg02546521 | *ULK1* | 8408 | 0.12 | 0.03633 |
| cg02547439 | *ELMO2* | 63916 | 0.18 | 0.03633 |
| cg02554810 | *SNX15* | 29907 | 0.13 | 0.03633 |
| cg02558133 | *PKN2* | 5586 | 0.15 | 0.03633 |
| cg02564061 | *C12orf25* | 84070 | 0.62 | 0.03633 |
| cg02599225 | *C10orf57* | 80195 | 0.08 | 0.03633 |
| cg02600515 | *ZBTB3* | 79842 | 0.18 | 0.03633 |
| cg02620470 | *EXT2* | 2132 | 0.11 | 0.03633 |
| cg02624129 | *SNX19* | 399979 | 0.31 | 0.03633 |
| cg02630122 | *HTF9C* | 27037 | 0.12 | 0.03633 |
| cg02681442 | *FOXG1B* | 2290 | 0.18 | 0.03633 |
| cg02690554 | *CITED1* | 4435 | 0.13 | 0.03633 |
| cg02724472 | *LRRC17* | 10234 | 0.41 | 0.03633 |
| cg02740128 | *DLG4* | 1742 | 0.16 | 0.03633 |
| cg02740947 | *RAD51L3* | 5892 | 0.09 | 0.03633 |
| cg02755525 | *NETO2* | 81831 | 0.11 | 0.03633 |
| cg02764611 | *MGC19604* | 112812 | 0.13 | 0.03633 |
| cg02774486 | *H2-ALPHA* | 113457 | -0.72 | 0.03633 |
| cg02809263 | *LRRC40* | 55631 | 0.13 | 0.03633 |
| cg02813021 | *ZMYND11* | 10771 | 0.12 | 0.03633 |
| cg02838492 | *KIF12* | 113220 | -0.71 | 0.03633 |
| cg02847216 | *CTAGE1* | 64693 | -0.79 | 0.03633 |
| cg02863237 | *RBM17* | 84991 | 0.09 | 0.03633 |
| cg02868338 | *EXOSC4* | 54512 | 0.11 | 0.03633 |
| cg02887841 | *TBX1* | 6899 | 0.14 | 0.03633 |
| cg02890926 | *TMEM53* | 79639 | 0.13 | 0.03633 |
| cg02901159 | *CLNS1A* | 1207 | 0.12 | 0.03633 |
| cg02921068 | *LSM8* | 51691 | 0.10 | 0.03633 |
| cg02930996 | *B4GALT6* | 9331 | 0.15 | 0.03633 |
| cg02936468 | *C9orf41* | 138199 | 0.12 | 0.03633 |
| cg02946754 | *NHLRC2* | 374354 | 0.13 | 0.03633 |
| cg02959669 | *ZNF256* | 10172 | 0.11 | 0.03633 |
| cg02973883 | *WDR21A* | 26094 | -0.45 | 0.03633 |
| cg02975142 | *FLCN* | 201163 | 0.13 | 0.03633 |
| cg02975259 | *NCK1* | 4690 | 0.10 | 0.03633 |
| cg02995130 | *RSN* | 6249 | 0.10 | 0.03633 |
| cg03005055 | *HSPA2* | 3306 | 0.10 | 0.03633 |
| cg03026462 | *FOXA1* | 3169 | 0.11 | 0.03633 |
| cg03050522 | *ARHGEF1* | 9138 | 0.18 | 0.03633 |
| cg03055492 | *UGP2* | 7360 | 0.13 | 0.03633 |
| cg03064241 | *RNF34* | 80196 | 0.14 | 0.03633 |
| cg03079549 | *PLDN* | 26258 | 0.11 | 0.03633 |
| cg03096975 | *EML2* | 24139 | 0.14 | 0.03633 |
| cg03098361 | *RFC2* | 5982 | 0.12 | 0.03633 |
| cg03108701 | *C6orf167* | 253714 | 0.13 | 0.03633 |
| cg03109316 | *ZNF80* | 7634 | -0.61 | 0.03633 |
| cg03115649 | *TOR1AIP1* | 26092 | 0.12 | 0.03633 |
| cg03116238 | *GNG8* | 94235 | 0.14 | 0.03633 |
| cg03122511 | *NKAP* | 79576 | 0.17 | 0.03633 |
| cg03126490 | *SPATA13* | 221178 | 0.14 | 0.03633 |
| cg03135598 | *SEPW1* | 6415 | 0.11 | 0.03633 |
| cg03136712 | *DND1* | 373863 | -0.80 | 0.03633 |
| cg03152785 | *KCNC4* | 3749 | 0.12 | 0.03633 |
| cg03159785 | *SKIL* | 6498 | 0.10 | 0.03633 |
| cg03176585 | *MRPS35* | 60488 | 0.11 | 0.03633 |
| cg03192551 | *CGI-69* | 51629 | 0.11 | 0.03633 |
| cg03197524 | *TNIP1* | 10318 | 0.19 | 0.03633 |
| cg03224418 | *SAMD10* | 140700 | -0.48 | 0.03633 |
| cg03231024 | *EFNA1* | 1942 | 0.13 | 0.03633 |
| cg03238797 | *ADAMTS18* | 170692 | 0.11 | 0.03633 |
| cg03243895 | *SPTAN1* | 6709 | 0.13 | 0.03633 |
| cg03245962 | *XRN1* | 54464 | 0.12 | 0.03633 |
| cg03274876 | *FLJ20534* | 54969 | 0.19 | 0.03633 |
| cg03302287 | *PKP2* | 5318 | 0.15 | 0.03633 |
| cg03305230 | *RAI1* | 10743 | 0.16 | 0.03633 |
| cg03310469 | *SIX2* | 10736 | 0.10 | 0.03633 |
| cg03328750 | *ARHGAP26* | 23092 | 0.08 | 0.03633 |
| cg03332970 | *ESAM* | 90952 | 0.14 | 0.03633 |
| cg03347966 | *FBXL6* | 26233 | 0.17 | 0.03633 |
| cg03359508 | *ANXA11* | 311 | 0.11 | 0.03633 |
| cg03402876 | *KCNF1* | 3754 | 0.14 | 0.03633 |
| cg03418289 | *LIX1L* | 128077 | 0.12 | 0.03633 |
| cg03442064 | *FOXP4* | 116113 | 0.13 | 0.03633 |
| cg03461495 | *ATG9A* | 79065 | 0.13 | 0.03633 |
| cg03513163 | *PCDHB1* | 29930 | 0.17 | 0.03633 |
| cg03522910 | *B4GALT3* | 8703 | 0.11 | 0.03633 |
| cg03533858 | *MORN1* | 79906 | -0.52 | 0.03633 |
| cg03538345 | *MTMR3* | 8897 | 0.17 | 0.03633 |
| cg03569637 | *FAM113B* | 91523 | 0.83 | 0.03633 |
| cg03580876 | *TRMU* | 55687 | 0.09 | 0.03633 |
| cg03640148 | *WDR6* | 11180 | 0.16 | 0.03633 |
| cg03640568 | *DDX17* | 10521 | 0.11 | 0.03633 |
| cg03648823 | *ZNF643* | 65243 | 0.16 | 0.03633 |
| cg03664992 | *BMP8A* | 353500 | 0.11 | 0.03633 |
| cg03698393 | *HAPLN3* | 145864 | 0.08 | 0.03633 |
| cg03761560 | *DNAJB12* | 54788 | 0.08 | 0.03633 |
| cg03764506 | *ESRRA* | 2101 | 0.11 | 0.03633 |
| cg03770147 | *KLF7* | 8609 | 0.11 | 0.03633 |
| cg03791917 | *BTK* | 695 | 0.83 | 0.03633 |
| cg03794550 | *FANCF* | 2188 | 0.09 | 0.03633 |
| cg03804568 | *CBX7* | 23492 | 0.15 | 0.03633 |
| cg03848145 | *C1orf52* | 148423 | 0.11 | 0.03633 |
| cg03848555 | *C9orf40* | 55071 | 0.11 | 0.03633 |
| cg03852570 | *C10orf33* | 84795 | 0.42 | 0.03633 |
| cg03861428 | *SDHB* | 6390 | 0.09 | 0.03633 |
| cg03870261 | *TIMM13* | 26517 | -0.75 | 0.03633 |
| cg03870862 | *ZC3H3* | 23144 | -0.53 | 0.03633 |
| cg03882305 | *TRIM50C* | 378108 | -0.57 | 0.03633 |
| cg03900542 | *DEDD2* | 162989 | 0.15 | 0.03633 |
| cg03914397 | *NMUR2* | 56923 | -0.40 | 0.03633 |
| cg03924115 | *P518* | 347148 | 0.66 | 0.03633 |
| cg03939485 | *RGL2* | 5863 | 0.13 | 0.03633 |
| cg03955296 | *ETV3* | 2117 | 0.13 | 0.03633 |
| cg03977782 | *SIDT1* | 54847 | 0.10 | 0.03633 |
| cg03998173 | *RHEB* | 6009 | 0.16 | 0.03633 |
| cg04006554 | *ENPP5* | 59084 | 0.14 | 0.03633 |
| cg04008913 | *SNRK* | 54861 | -0.62 | 0.03633 |
| cg04041960 | *RGS10* | 6001 | 0.13 | 0.03633 |
| cg04057053 | *WNT2B* | 7482 | 0.14 | 0.03633 |
| cg04076481 | *FLJ12949* | 65095 | 0.22 | 0.03633 |
| cg04099673 | *WDR7* | 23335 | 0.14 | 0.03633 |
| cg04133652 | *MLLT6* | 4302 | 0.47 | 0.03633 |
| cg04136480 | *BAT1* | 7919 | 0.10 | 0.03633 |
| cg04172348 | *SYN2* | 6854 | 0.15 | 0.03633 |
| cg04187545 | *RPS20* | 6224 | 0.16 | 0.03633 |
| cg04209631 | *C11orf54* | 28970 | 0.13 | 0.03633 |
| cg04223442 | *NCOR1* | 9611 | 0.09 | 0.03633 |
| cg04228553 | *CAPN7* | 23473 | 0.13 | 0.03633 |
| cg04228573 | *MAP2K6* | 5608 | 0.10 | 0.03633 |
| cg04230869 | *RRM2B* | 50484 | 0.14 | 0.03633 |
| cg04237663 | *KCNV1* | 27012 | 0.13 | 0.03633 |
| cg04276232 | *UGT2B4* | 7363 | -0.20 | 0.03633 |
| cg04282422 | *PAGE1* | 8712 | -0.56 | 0.03633 |
| cg04284814 | *C5orf16* | 285613 | 0.11 | 0.03633 |
| cg04286043 | *C6orf69* | 222658 | 0.10 | 0.03633 |
| cg04297329 | *NAP1L2* | 4674 | 0.18 | 0.03633 |
| cg04311606 | *ZNF136* | 7695 | 0.13 | 0.03633 |
| cg04317940 | *PMS2* | 5395 | 0.14 | 0.03633 |
| cg04329069 | *AP1B1* | 162 | 0.11 | 0.03633 |
| cg04340502 | *GSTA3* | 2940 | 0.90 | 0.03633 |
| cg04349311 | *TXNDC12* | 51060 | 0.13 | 0.03633 |
| cg04353769 | *MS4A6A* | 64231 | 0.87 | 0.03633 |
| cg04376617 | *ATP5A1* | 498 | -0.25 | 0.03633 |
| cg04380513 | *ST8SIA4* | 7903 | 0.16 | 0.03633 |
| cg04383274 | *C21orf55* | 54943 | 0.14 | 0.03633 |
| cg04385519 | *ADCY9* | 115 | -0.74 | 0.03633 |
| cg04391540 | *ZNF541* | 84215 | -0.52 | 0.03633 |
| cg04400030 | *SNX1* | 6642 | 0.14 | 0.03633 |
| cg04409945 | *PSMB3* | 5691 | 0.15 | 0.03633 |
| cg04416752 | *ST6GALNAC4* | 27090 | 0.22 | 0.03633 |
| cg04422896 | *C12orf43* | 64897 | 0.13 | 0.03633 |
| cg04437590 | *NDUFA5* | 4698 | 0.13 | 0.03633 |
| cg04466253 | *ACBD5* | 91452 | 0.13 | 0.03633 |
| cg04542415 | *ZFP64* | 55734 | 0.11 | 0.03633 |
| cg04542938 | *ATXN1* | 6310 | 0.12 | 0.03633 |
| cg04544154 | *ELF4* | 2000 | -0.50 | 0.03633 |
| cg04564646 | *MOV10* | 4343 | 0.11 | 0.03633 |
| cg04587829 | *FN3K* | 64122 | -0.70 | 0.03633 |
| cg04603184 | *RNF182* | 221687 | 0.19 | 0.03633 |
| cg04614380 | *SEMA3F* | 6405 | 0.12 | 0.03633 |
| cg04655481 | *GPR21* | 2844 | 0.73 | 0.03633 |
| cg04689061 | *PKIA* | 5569 | 0.16 | 0.03633 |
| cg04718342 | *CLPX* | 10845 | 0.15 | 0.03633 |
| cg04721883 | *ESX1* | 80712 | 0.16 | 0.03633 |
| cg04731091 | *WDR37* | 22884 | 0.12 | 0.03633 |
| cg04743063 | *RTCD1* | 8634 | 0.12 | 0.03633 |
| cg04754011 | *GPR44* | 11251 | 0.68 | 0.03633 |
| cg04756629 | *LOC400696* | 400696 | 0.13 | 0.03633 |
| cg04770504 | *DFNA5* | 1687 | 0.12 | 0.03633 |
| cg04810997 | *TAS2R60* | 338398 | 0.47 | 0.03633 |
| cg04816348 | *CLEC4G* | 339390 | -0.90 | 0.03633 |
| cg04824341 | *CHCHD8* | 51287 | 0.10 | 0.03633 |
| cg04849025 | *YIPF2* | 78992 | 0.11 | 0.03633 |
| cg04853383 | *C12orf49* | 79794 | 0.08 | 0.03633 |
| cg04875162 | *PABPC5* | 140886 | 0.13 | 0.03633 |
| cg04881903 | *CAPG* | 822 | 0.73 | 0.03633 |
| cg04884122 | *PSMB5* | 5693 | 0.14 | 0.03633 |
| cg04929736 | *CLU* | 1191 | 0.13 | 0.03633 |
| cg04946916 | *HUS1B* | 135458 | -0.97 | 0.03633 |
| cg04950789 | *E2F5* | 1875 | 0.15 | 0.03633 |
| cg04984513 | *BRI3BP* | 140707 | 0.15 | 0.03633 |
| cg05038121 | *FAH* | 2184 | 0.21 | 0.03633 |
| cg05103623 | *TAGAP* | 117289 | 0.45 | 0.03633 |
| cg05111036 | *HIGD1A* | 25994 | 0.19 | 0.03633 |
| cg05112986 | *SUPT3H* | 8464 | 0.14 | 0.03633 |
| cg05127380 | *STIP1* | 10963 | 0.25 | 0.03633 |
| cg05139924 | *LZTR1* | 8216 | 0.12 | 0.03633 |
| cg05149586 | *SDSL* | 113675 | 0.77 | 0.03633 |
| cg05155595 | *ANXA4* | 307 | 0.31 | 0.03633 |
| cg05205664 | *UCP2* | 7351 | 0.14 | 0.03633 |
| cg05208878 | *PRAME* | 23532 | -0.61 | 0.03633 |
| cg05245861 | *C18orf34* | 374864 | 0.10 | 0.03633 |
| cg05262335 | *TAGAP* | 117289 | 0.41 | 0.03633 |
| cg05288803 | *TIMP3* | 7078 | 0.13 | 0.03633 |
| cg05310071 | *PIGL* | 9487 | 0.16 | 0.03633 |
| cg05321960 | *KIAA0907* | 22889 | 0.17 | 0.03633 |
| cg05337966 | *C13orf1* | 57213 | 0.12 | 0.03633 |
| cg05338167 | *CALML4* | 91860 | 0.55 | 0.03633 |
| cg05341670 | *C1orf83* | 127428 | 0.12 | 0.03633 |
| cg05346899 | *LASS4* | 79603 | 0.13 | 0.03633 |
| cg05360220 | *TNFRSF14* | 8764 | 0.61 | 0.03633 |
| cg05367858 | *RNF141* | 50862 | 0.13 | 0.03633 |
| cg05388361 | *STAT2* | 6773 | 0.14 | 0.03633 |
| cg05390437 | *WDR45L* | 56270 | 0.16 | 0.03633 |
| cg05395995 | *LOC221143* | 221143 | 0.13 | 0.03633 |
| cg05399707 | *TRIP10* | 9322 | 0.13 | 0.03633 |
| cg05402265 | *ICK* | 22858 | 0.10 | 0.03633 |
| cg05406101 | *C21orf6* | 10069 | -0.71 | 0.03633 |
| cg05411032 | *CLPTM1* | 1209 | 0.17 | 0.03633 |
| cg05418487 | *KCNQ1* | 3784 | 0.12 | 0.03633 |
| cg05434957 | *ICA1* | 3382 | 0.10 | 0.03633 |
| cg05446471 | *HDAC11* | 79885 | -0.83 | 0.03633 |
| cg05451974 | *C20orf35* | 55861 | 0.10 | 0.03633 |
| cg05467458 | *SLC7A9* | 11136 | 0.37 | 0.03633 |
| cg05498681 | *SRC* | 6714 | -0.19 | 0.03633 |
| cg05499853 | *C10orf97* | 80013 | 0.15 | 0.03633 |
| cg05516832 | *SGTA* | 6449 | 0.13 | 0.03633 |
| cg05520656 | *ZNF681* | 148213 | 0.13 | 0.03633 |
| cg05538387 | *RP11-529I10.4* | 25911 | 0.12 | 0.03633 |
| cg05552534 | *ABCB10* | 23456 | 0.22 | 0.03633 |
| cg05575043 | *ZNF394* | 84124 | 0.11 | 0.03633 |
| cg05587736 | *NUP93* | 9688 | -0.23 | 0.03633 |
| cg05596756 | *FAM113B* | 91523 | 0.83 | 0.03633 |
| cg05603623 | *TIMM44* | 10469 | 0.16 | 0.03633 |
| cg05650565 | *UBXD2* | 23190 | 0.12 | 0.03633 |
| cg05669100 | *FAM103A1* | 83640 | 0.13 | 0.03633 |
| cg05670596 | *CCRL2* | 9034 | 0.77 | 0.03633 |
| cg05681757 | *FGD4* | 121512 | 0.34 | 0.03633 |
| cg05684015 | *DR1* | 1810 | 0.11 | 0.03633 |
| cg05717160 | *USMG5* | 84833 | 0.11 | 0.03633 |
| cg05718625 | *TMEM85* | 51234 | 0.15 | 0.03633 |
| cg05722906 | *CYP4F12* | 66002 | 0.64 | 0.03633 |
| cg05745457 | *TKTL2* | 84076 | -0.71 | 0.03633 |
| cg05764607 | *PHF22* | 57117 | 0.14 | 0.03633 |
| cg05766425 | *CROP* | 51747 | 0.11 | 0.03633 |
| cg05782975 | *NGFRAP1* | 27018 | 0.16 | 0.03633 |
| cg05795313 | *ZNF641* | 121274 | 0.12 | 0.03633 |
| cg05812599 | *CLPB* | 81570 | -0.52 | 0.03633 |
| cg05815906 | *RAB33B* | 83452 | 0.12 | 0.03633 |
| cg05832051 | *MYADM* | 91663 | 0.14 | 0.03633 |
| cg05845357 | *PGEA1* | 25776 | 0.14 | 0.03633 |
| cg05845503 | *GRHPR* | 9380 | 0.11 | 0.03633 |
| cg05851042 | *ATP9A* | 10079 | -0.46 | 0.03633 |
| cg05857825 | *MAPRE1* | 22919 | 0.15 | 0.03633 |
| cg05890578 | *PRKCE* | 5581 | 0.16 | 0.03633 |
| cg05909475 | *ETV3* | 2117 | 0.14 | 0.03633 |
| cg05983247 | *SLTM* | 79811 | 0.12 | 0.03633 |
| cg06005396 | *HCN2* | 610 | 0.17 | 0.03633 |
| cg06043114 | *TSPAN2* | 10100 | 0.13 | 0.03633 |
| cg06057707 | *XAGE2* | 9502 | -0.84 | 0.03633 |
| cg06063714 | *GABRD* | 2563 | -0.61 | 0.03633 |
| cg06090421 | *PPM1D* | 8493 | 0.14 | 0.03633 |
| cg06110365 | *TTLL12* | 23170 | 0.22 | 0.03633 |
| cg06110728 | *NELL2* | 4753 | 0.17 | 0.03633 |
| cg06117855 | *CLEC3B* | 7123 | 0.85 | 0.03633 |
| cg06118312 | *GZMA* | 3001 | 0.63 | 0.03633 |
| cg06132694 | *NDST3* | 9348 | 0.13 | 0.03633 |
| cg06187947 | *GULP1* | 51454 | 0.10 | 0.03633 |
| cg06192619 | *SLC1A6* | 6511 | -0.93 | 0.03633 |
| cg06200339 | *SOX30* | 11063 | -0.69 | 0.03633 |
| cg06213287 | *LIX1* | 167410 | 0.43 | 0.03633 |
| cg06244417 | *FCN1* | 2219 | 0.34 | 0.03633 |
| cg06244906 | *ZIM2* | 23619 | -0.59 | 0.03633 |
| cg06255524 | *DNAJC3* | 5611 | 0.17 | 0.03633 |
| cg06292277 | *CLCN3* | 1182 | 0.14 | 0.03633 |
| cg06331674 | *KIAA0241* | 23080 | 0.10 | 0.03633 |
| cg06334093 | *CCDC28A* | 25901 | 0.13 | 0.03633 |
| cg06336792 | *CREM* | 1390 | 0.10 | 0.03633 |
| cg06394229 | *LGALS4* | 3960 | 0.40 | 0.03633 |
| cg06403126 | *PPP2R2A* | 5520 | 0.13 | 0.03633 |
| cg06416685 | *TSPAN6* | 7105 | 0.18 | 0.03633 |
| cg06417705 | *ETAA16* | 54465 | 0.11 | 0.03633 |
| cg06417962 | *RNH1* | 6050 | -0.41 | 0.03633 |
| cg06428195 | *GOT1* | 2805 | 0.13 | 0.03633 |
| cg06433658 | *FAM63A* | 55793 | 0.68 | 0.03633 |
| cg06456031 | *FLJ11000* | 55281 | 0.42 | 0.03633 |
| cg06462703 | *CHFR* | 55743 | -0.12 | 0.03633 |
| cg06463913 | *BET1L* | 51272 | 0.11 | 0.03633 |
| cg06465021 | *SLC12A2* | 6558 | 0.11 | 0.03633 |
| cg06470552 | *CSPG6* | 9126 | 0.13 | 0.03633 |
| cg06490162 | *C16orf63* | 123811 | 0.10 | 0.03633 |
| cg06497795 | *ZC3H10* | 84872 | 0.15 | 0.03633 |
| cg06530118 | *CLN3* | 1201 | 0.10 | 0.03633 |
| cg06588247 | *TRAF3* | 7187 | 0.11 | 0.03633 |
| cg06630241 | *SOCS2* | 8835 | 0.19 | 0.03633 |
| cg06640279 | *FAIM3* | 9214 | 0.73 | 0.03633 |
| cg06661765 | *NFIX* | 4784 | 0.16 | 0.03633 |
| cg06679221 | *THUMPD3* | 25917 | 0.14 | 0.03633 |
| cg06700462 | *PHKA2* | 5256 | 0.13 | 0.03633 |
| cg06734828 | *KIAA0251* | 23042 | 0.12 | 0.03633 |
| cg06782849 | *NPHP3* | 27031 | 0.13 | 0.03633 |
| cg06800962 | *TM4SF1* | 4071 | 0.60 | 0.03633 |
| cg06806080 | *CD47* | 961 | -0.73 | 0.03633 |
| cg06807379 | *DAPK3* | 1613 | 0.16 | 0.03633 |
| cg06815817 | *ZNF212* | 7988 | 0.14 | 0.03633 |
| cg06833562 | *C1orf181* | 54680 | 0.11 | 0.03633 |
| cg06839953 | *C1orf160* | 84065 | 0.14 | 0.03633 |
| cg06840042 | *MKRN2* | 23609 | 0.11 | 0.03633 |
| cg06878726 | *RBM19* | 9904 | 0.10 | 0.03633 |
| cg06887170 | *FAM14A* | 83982 | 0.18 | 0.03633 |
| cg06887663 | *SNAPC1* | 6617 | 0.10 | 0.03633 |
| cg06963844 | *FBXO6* | 26270 | 0.14 | 0.03633 |
| cg06975499 | *EZH1* | 2145 | 0.16 | 0.03633 |
| cg06976977 | *FLJ38991* | 285521 | 0.11 | 0.03633 |
| cg06983551 | *BUB1* | 699 | 0.28 | 0.03633 |
| cg07000551 | *BMF* | 90427 | 0.11 | 0.03633 |
| cg07006526 | *SMTN* | 6525 | 0.12 | 0.03633 |
| cg07009002 | *MB* | 4151 | 0.60 | 0.03633 |
| cg07017374 | *FLT3* | 2322 | 0.17 | 0.03633 |
| cg07034269 | *UGCGL1* | 56886 | 0.10 | 0.03633 |
| cg07038617 | *ABCF2* | 10061 | 0.12 | 0.03633 |
| cg07038887 | *BLMH* | 642 | 0.12 | 0.03633 |
| cg07048066 | *C5orf5* | 51306 | 0.18 | 0.03633 |
| cg07052087 | *TPI1* | 7167 | 0.11 | 0.03633 |
| cg07059052 | *TCEB2* | 6923 | 0.13 | 0.03633 |
| cg07060572 | *MAML2* | 84441 | 0.12 | 0.03633 |
| cg07061913 | *DNAJC9* | 23234 | 0.15 | 0.03633 |
| cg07067241 | *TRPC1* | 7220 | 0.13 | 0.03633 |
| cg07070940 | *HDHD1A* | 8226 | 0.13 | 0.03633 |
| cg07086898 | *USMG5* | 84833 | 0.14 | 0.03633 |
| cg07115558 | *TLE2* | 7089 | 0.21 | 0.03633 |
| cg07125009 | *ZNF689* | 115509 | 0.12 | 0.03633 |
| cg07128330 | *MDS028* | 55846 | 0.13 | 0.03633 |
| cg07186138 | *APOBEC3C* | 27350 | 0.36 | 0.03633 |
| cg07191600 | *TRERF1* | 55809 | 0.11 | 0.03633 |
| cg07200280 | *RAFTLIN* | 23180 | 0.11 | 0.03633 |
| cg07236358 | *POP5* | 51367 | 0.10 | 0.03633 |
| cg07237830 | *BSCL2* | 26580 | -0.56 | 0.03633 |
| cg07251857 | *ALPK3* | 57538 | -0.84 | 0.03633 |
| cg07278207 | *DCDC1* | 341019 | 0.11 | 0.03633 |
| cg07281688 | *PTGES3* | 10728 | 0.11 | 0.03633 |
| cg07291563 | *GRWD1* | 83743 | 0.14 | 0.03633 |
| cg07314337 | *ARL6IP6* | 151188 | 0.12 | 0.03633 |
| cg07330718 | *HYOU1* | 10525 | 0.12 | 0.03633 |
| cg07342545 | *FOSL2* | 2355 | 0.12 | 0.03633 |
| cg07346310 | *RBM24* | 221662 | 0.19 | 0.03633 |
| cg07347725 | *C9orf18* | 254956 | 0.13 | 0.03633 |
| cg07398350 | *C16orf57* | 79650 | 0.17 | 0.03633 |
| cg07405796 | *TRIM40* | 135644 | 0.53 | 0.03633 |
| cg07408456 | *PGLYRP2* | 114770 | 0.69 | 0.03633 |
| cg07409200 | *FLJ40919* | 144809 | 0.75 | 0.03633 |
| cg07411229 | *LOC128153* | 128153 | 0.17 | 0.03633 |
| cg07446846 | *SLC6A8* | 6535 | -0.48 | 0.03633 |
| cg07453618 | *FAM79A* | 127262 | 0.10 | 0.03633 |
| cg07454215 | *DCP1A* | 55802 | 0.11 | 0.03633 |
| cg07479491 | *BMP6* | 654 | 0.16 | 0.03633 |
| cg07498879 | *OSM* | 5008 | 0.91 | 0.03633 |
| cg07525077 | *RNASE3* | 6037 | 0.56 | 0.03633 |
| cg07526021 | *DHFR* | 1719 | 0.11 | 0.03633 |
| cg07550278 | *C10orf4* | 118924 | 0.14 | 0.03633 |
| cg07557424 | *C9orf23* | 138716 | 0.16 | 0.03633 |
| cg07584959 | *THRAP5* | 10025 | 0.18 | 0.03633 |
| cg07589355 | *RBMS2* | 5939 | 0.26 | 0.03633 |
| cg07595943 | *LOC161931* | 161931 | -0.99 | 0.03633 |
| cg07602200 | *P2RX7* | 5027 | 0.72 | 0.03633 |
| cg07621042 | *CXorf55* | 139804 | -0.76 | 0.03633 |
| cg07637239 | *KCNK18* | 338567 | 0.11 | 0.03633 |
| cg07652553 | *ZCCHC17* | 51538 | 0.12 | 0.03633 |
| cg07657776 | *UBE3B* | 89910 | -0.59 | 0.03633 |
| cg07671603 | *C7orf13* | 129790 | 0.12 | 0.03633 |
| cg07685786 | *S100B* | 6285 | 0.30 | 0.03633 |
| cg07695590 | *CEBPZ* | 10153 | 0.12 | 0.03633 |
| cg07697078 | *PFDN1* | 5201 | 0.15 | 0.03633 |
| cg07719512 | *SLC11A1* | 6556 | 0.70 | 0.03633 |
| cg07748017 | *PEX5* | 5830 | 0.14 | 0.03633 |
| cg07761912 | *TOR1AIP2* | 163590 | 0.13 | 0.03633 |
| cg07766315 | *LOC400451* | 400451 | 0.17 | 0.03633 |
| cg07789552 | *STK40* | 83931 | 0.11 | 0.03633 |
| cg07808960 | *RBM6* | 10180 | 0.18 | 0.03633 |
| cg07817698 | *DHODH* | 1723 | 0.12 | 0.03633 |
| cg07842593 | *SEPHS2* | 22928 | 0.12 | 0.03633 |
| cg07847428 | *MARCH2* | 51257 | 0.12 | 0.03633 |
| cg07874520 | *THEM5* | 284486 | -0.25 | 0.03633 |
| cg07894162 | *TUG1* | 55000 | 0.11 | 0.03633 |
| cg07903918 | *GABBR2* | 9568 | 0.13 | 0.03633 |
| cg07909265 | *ITGAV* | 3685 | 0.14 | 0.03633 |
| cg07914959 | *BACH2* | 60468 | 0.11 | 0.03633 |
| cg07925687 | *RABL3* | 285282 | 0.16 | 0.03633 |
| cg07933199 | *C3orf59* | 151963 | 0.11 | 0.03633 |
| cg07973967 | *CD79B* | 974 | 0.78 | 0.03633 |
| cg07981910 | *DAB2IP* | 153090 | 0.12 | 0.03633 |
| cg07989514 | *C14orf4* | 64207 | 0.09 | 0.03633 |
| cg08012454 | *KIF5B* | 3799 | 0.14 | 0.03633 |
| cg08020051 | *GPAA1* | 8733 | 0.12 | 0.03633 |
| cg08044694 | *BRD4* | 23476 | 0.73 | 0.03633 |
| cg08047907 | *C1orf114* | 57821 | 0.13 | 0.03633 |
| cg08063013 | *COX8A* | 1351 | 0.10 | 0.03633 |
| cg08072716 | *GPR62* | 118442 | -0.70 | 0.03633 |
| cg08079693 | *CHD6* | 84181 | 0.14 | 0.03633 |
| cg08108311 | *WNK4* | 65266 | 0.16 | 0.03633 |
| cg08120190 | *C7orf36* | 57002 | 0.16 | 0.03633 |
| cg08122545 | *HES4* | 57801 | 0.18 | 0.03633 |
| cg08151470 | *UBE4B* | 10277 | -0.23 | 0.03633 |
| cg08156349 | *MEF2D* | 4209 | 0.13 | 0.03633 |
| cg08164315 | *PTGER4* | 5734 | 0.16 | 0.03633 |
| cg08186124 | *LZTFL1* | 54585 | 0.12 | 0.03633 |
| cg08198102 | *PTPLA* | 9200 | 0.13 | 0.03633 |
| cg08222514 | *SENP6* | 26054 | 0.18 | 0.03633 |
| cg08229627 | *NPHP1* | 4867 | 0.12 | 0.03633 |
| cg08235271 | *LPHN2* | 23266 | 0.15 | 0.03633 |
| cg08242020 | *RTN3* | 10313 | 0.22 | 0.03633 |
| cg08250444 | *EFNA1* | 1942 | 0.12 | 0.03633 |
| cg08260959 | *HIST1H4F* | 8361 | 0.14 | 0.03633 |
| cg08261666 | *VAMP2* | 6844 | 0.12 | 0.03633 |
| cg08274234 | *SEMA3E* | 9723 | 0.13 | 0.03633 |
| cg08284598 | *SLC4A11* | 83959 | -0.39 | 0.03633 |
| cg08300860 | *LDB3* | 11155 | -0.10 | 0.03633 |
| cg08305742 | *LOC51315* | 51315 | 0.12 | 0.03633 |
| cg08312191 | *SNRPG* | 6637 | 0.15 | 0.03633 |
| cg08341874 | *VMO1* | 284013 | 0.16 | 0.03633 |
| cg08348496 | *HAPLN3* | 145864 | 0.16 | 0.03633 |
| cg08351105 | *FOSL2* | 2355 | 0.14 | 0.03633 |
| cg08374799 | *ITGB7* | 3695 | 0.49 | 0.03633 |
| cg08383063 | *RB1* | 5925 | 0.09 | 0.03633 |
| cg08390021 | *C18orf54* | 162681 | 0.11 | 0.03633 |
| cg08392591 | *ANKRD11* | 29123 | 0.20 | 0.03633 |
| cg08418332 | *CCL19* | 6363 | 0.30 | 0.03633 |
| cg08418978 | *CLDN10* | 9071 | -0.55 | 0.03633 |
| cg08446111 | *ABCC11* | 85320 | -0.46 | 0.03633 |
| cg08450168 | *RAB42* | 115273 | -0.44 | 0.03633 |
| cg08458170 | *ZNF537* | 57616 | -0.11 | 0.03633 |
| cg08461397 | *SNTB2* | 6645 | 0.11 | 0.03633 |
| cg08463061 | *RND3* | 390 | 0.18 | 0.03633 |
| cg08469834 | *CASP4* | 837 | 0.23 | 0.03633 |
| cg08492707 | *IFNAR1* | 3454 | 0.12 | 0.03633 |
| cg08494812 | *TAF1B* | 9014 | 0.21 | 0.03633 |
| cg08496601 | *EBP* | 10682 | 0.11 | 0.03633 |
| cg08504583 | *FLJ30046* | 122060 | 0.65 | 0.03633 |
| cg08525207 | *RPS7* | 6201 | 0.13 | 0.03633 |
| cg08553339 | *CRSP7* | 9441 | 0.11 | 0.03633 |
| cg08573893 | *C8orf32* | 55093 | 0.12 | 0.03633 |
| cg08576197 | *CCDC57* | 284001 | 0.13 | 0.03633 |
| cg08578023 | *CTSS* | 1520 | 0.32 | 0.03633 |
| cg08610361 | *E2F7* | 144455 | 0.12 | 0.03633 |
| cg08612037 | *BTBD11* | 121551 | 0.10 | 0.03633 |
| cg08634539 | *DHRS7* | 51635 | 0.13 | 0.03633 |
| cg08661290 | *PHF13* | 148479 | 0.14 | 0.03633 |
| cg08693206 | *AP1B1* | 162 | 0.11 | 0.03633 |
| cg08707078 | *MGC72075* | 340277 | 0.16 | 0.03633 |
| cg08715837 | *TUBA8* | 51807 | 0.12 | 0.03633 |
| cg08724474 | *SLC36A1* | 206358 | 0.12 | 0.03633 |
| cg08737421 | *MAX* | 4149 | 0.13 | 0.03633 |
| cg08753553 | *DEFB106A* | 245909 | 0.21 | 0.03633 |
| cg08771731 | *BASP1* | 10409 | 0.12 | 0.03633 |
| cg08779777 | *PIK3CG* | 5294 | 0.37 | 0.03633 |
| cg08790491 | *ARID4A* | 5926 | 0.15 | 0.03633 |
| cg08790550 | *LOC112714* | 112714 | -0.70 | 0.03633 |
| cg08800033 | *CETN2* | 1069 | 0.13 | 0.03633 |
| cg08804892 | *TRAK1* | 22906 | 0.34 | 0.03633 |
| cg08818385 | *FAHD2A* | 51011 | -0.39 | 0.03633 |
| cg08843314 | *CXCR3* | 2833 | -0.50 | 0.03633 |
| cg08861115 | *IL1F9* | 56300 | -0.11 | 0.03633 |
| cg08914623 | *ALX4* | 60529 | 0.11 | 0.03633 |
| cg08928646 | *UBE1L2* | 55236 | 0.16 | 0.03633 |
| cg08929103 | *CRHR1* | 1394 | -0.64 | 0.03633 |
| cg08983975 | *TCOF1* | 6949 | 0.15 | 0.03633 |
| cg08987989 | *PGGT1B* | 5229 | -0.59 | 0.03633 |
| cg08995424 | *ATP5G2* | 517 | 0.10 | 0.03633 |
| cg09018824 | *DKFZP686A01247* | 22998 | -0.24 | 0.03633 |
| cg09054876 | *PECI* | 10455 | 0.17 | 0.03633 |
| cg09059945 | *MEST* | 4232 | 0.13 | 0.03633 |
| cg09082287 | *DNAJC6* | 9829 | 0.17 | 0.03633 |
| cg09094355 | *PRKX* | 5613 | 0.16 | 0.03633 |
| cg09103232 | *SLC2A4* | 6517 | 0.11 | 0.03633 |
| cg09130402 | *TXNL1* | 9352 | 0.13 | 0.03633 |
| cg09146695 | *LEF1* | 51176 | 0.17 | 0.03633 |
| cg09156233 | *BMPR1B* | 658 | 0.10 | 0.03633 |
| cg09220361 | *GABRG2* | 2566 | 0.48 | 0.03633 |
| cg09238677 | *C3AR1* | 719 | 0.36 | 0.03633 |
| cg09239756 | *CKAP4* | 10970 | 0.13 | 0.03633 |
| cg09245378 | *KIAA0157* | 23172 | 0.12 | 0.03633 |
| cg09252204 | *CNKSR2* | 22866 | 0.11 | 0.03633 |
| cg09260830 | *GNA14* | 9630 | 0.12 | 0.03633 |
| cg09266433 | *OSBPL6* | 114880 | 0.13 | 0.03633 |
| cg09280976 | *MEG3* | 55384 | -0.52 | 0.03633 |
| cg09283635 | *LOC168850* | 168850 | 0.14 | 0.03633 |
| cg09296044 | *CCNDBP1* | 23582 | 0.11 | 0.03633 |
| cg09300114 | *SLC16A5* | 9121 | 0.10 | 0.03633 |
| cg09304040 | *CDK2* | 1017 | 0.10 | 0.03633 |
| cg09307264 | *INCA1* | 388324 | -0.65 | 0.03633 |
| cg09327628 | *MARCKSL1* | 65108 | 0.14 | 0.03633 |
| cg09338170 | *SLC35A3* | 23443 | 0.17 | 0.03633 |
| cg09339301 | *QKI* | 9444 | 0.08 | 0.03633 |
| cg09358725 | *LMO2* | 4005 | 0.66 | 0.03633 |
| cg09377486 | *BIRC8* | 112401 | -0.33 | 0.03633 |
| cg09381701 | *SASH1* | 23328 | 0.10 | 0.03633 |
| cg09399047 | *DALRD3* | 55152 | 0.15 | 0.03633 |
| cg09405083 | *C10orf26* | 54838 | 0.64 | 0.03633 |
| cg09406238 | *MTMR8* | 55613 | 0.24 | 0.03633 |
| cg09409398 | *SF3B4* | 10262 | 0.16 | 0.03633 |
| cg09410845 | *BMPR2* | 659 | 0.13 | 0.03633 |
| cg09424308 | *FLJ90575* | 257236 | -0.56 | 0.03633 |
| cg09424925 | *RAD51L1* | 5890 | 0.13 | 0.03633 |
| cg09430689 | *C14orf1* | 11161 | 0.10 | 0.03633 |
| cg09450238 | *BTBD6* | 90135 | -0.63 | 0.03633 |
| cg09461185 | *PTGS2* | 5743 | 0.10 | 0.03633 |
| cg09475324 | *HYPE* | 11153 | 0.13 | 0.03633 |
| cg09478597 | *SPFH2* | 11160 | 0.11 | 0.03633 |
| cg09499698 | *CENPC1* | 1060 | 0.12 | 0.03633 |
| cg09499849 | *ACVR1* | 90 | 0.86 | 0.03633 |
| cg09500672 | *MGC52057* | 130574 | 0.11 | 0.03633 |
| cg09516965 | *PTGDR* | 5729 | 0.16 | 0.03633 |
| cg09535168 | *SF4* | 57794 | 0.12 | 0.03633 |
| cg09543792 | *SLC16A13* | 201232 | 0.14 | 0.03633 |
| cg09562455 | *NR0B1* | 190 | 0.14 | 0.03633 |
| cg09572106 | *PDLIM7* | 9260 | 0.12 | 0.03633 |
| cg09610963 | *LRRC37A* | 9884 | -0.27 | 0.03633 |
| cg09615982 | *E4F1* | 1877 | 0.14 | 0.03633 |
| cg09619786 | *RPP25* | 54913 | 0.10 | 0.03633 |
| cg09628199 | *TSNARE1* | 203062 | 0.12 | 0.03633 |
| cg09658438 | *CS* | 1431 | 0.09 | 0.03633 |
| cg09663323 | *LOC112714* | 112714 | -0.73 | 0.03633 |
| cg09669099 | *TERF2* | 7014 | 0.15 | 0.03633 |
| cg09685104 | *C18orf19* | 125228 | 0.11 | 0.03633 |
| cg09694403 | *DHRS7* | 51635 | 0.14 | 0.03633 |
| cg09712527 | *KBTBD7* | 84078 | 0.11 | 0.03633 |
| cg09750385 | *GUCY1A3* | 2982 | 0.14 | 0.03633 |
| cg09809242 | *MKI67IP* | 84365 | 0.13 | 0.03633 |
| cg09813840 | *SELPLG* | 6404 | 0.76 | 0.03633 |
| cg09822907 | *EME1* | 146956 | 0.17 | 0.03633 |
| cg09831311 | *COX4NB* | 10328 | 0.14 | 0.03633 |
| cg09888137 | *ACTR1A* | 10121 | 0.13 | 0.03633 |
| cg09889850 | *C14orf11* | 55837 | 0.14 | 0.03633 |
| cg09892203 | *CACNG4* | 27092 | 0.12 | 0.03633 |
| cg09906309 | *C11orf46* | 120534 | 0.10 | 0.03633 |
| cg09906488 | *CAMK1D* | 57118 | 0.13 | 0.03633 |
| cg09917461 | *CSAG1* | 158511 | -0.52 | 0.03633 |
| cg09922115 | *GIT2* | 9815 | 0.11 | 0.03633 |
| cg09948350 | *FLJ25084* | 151516 | 0.31 | 0.03633 |
| cg09954385 | *ARHGAP8* | 23779 | 0.12 | 0.03633 |
| cg09972884 | *RRN3* | 54700 | 0.14 | 0.03633 |
| cg09979256 | *RSPO3* | 84870 | 0.14 | 0.03633 |
| cg10001720 | *LAPTM5* | 7805 | 0.72 | 0.03633 |
| cg10002561 | *CCNA2* | 890 | 0.14 | 0.03633 |
| cg10045881 | *CHI3L2* | 1117 | 0.21 | 0.03633 |
| cg10049968 | *C9orf25* | 203259 | 0.11 | 0.03633 |
| cg10061138 | *STAB1* | 23166 | 0.32 | 0.03633 |
| cg10065416 | *MPP3* | 4356 | 0.11 | 0.03633 |
| cg10066332 | *PPP1R13B* | 23368 | 0.16 | 0.03633 |
| cg10078415 | *ACSM3* | 6296 | 0.17 | 0.03633 |
| cg10107671 | *FLJ45684* | 400666 | -0.11 | 0.03633 |
| cg10139614 | *APRT* | 353 | 0.14 | 0.03633 |
| cg10174687 | *FAM98B* | 283742 | 0.15 | 0.03633 |
| cg10200408 | *ASB8* | 140461 | 0.14 | 0.03633 |
| cg10214058 | *CNOT8* | 9337 | 0.16 | 0.03633 |
| cg10237911 | *ANKRA2* | 57763 | -0.43 | 0.03633 |
| cg10265786 | *DOM3Z* | 1797 | 0.13 | 0.03633 |
| cg10299667 | *SLC25A10* | 1468 | 0.13 | 0.03633 |
| cg10342590 | *MRPL49* | 740 | -0.18 | 0.03633 |
| cg10343901 | *RAB6A* | 5870 | 0.12 | 0.03633 |
| cg10401803 | *TAF1* | 6872 | 0.14 | 0.03633 |
| cg10464775 | *LAMP1* | 3916 | -0.60 | 0.03633 |
| cg10479325 | *MGC33302* | 256471 | 0.11 | 0.03633 |
| cg10480741 | *PPP5C* | 5536 | 0.14 | 0.03633 |
| cg10488637 | *ZNF180* | 7733 | 0.18 | 0.03633 |
| cg10490196 | *TPCN2* | 219931 | 0.12 | 0.03633 |
| cg10503232 | *KCNQ1DN* | 55539 | -0.92 | 0.03633 |
| cg10521267 | *ZNF684* | 127396 | 0.18 | 0.03633 |
| cg10530281 | *TBX3* | 6926 | 0.12 | 0.03633 |
| cg10541755 | *EIF5A2* | 56648 | 0.13 | 0.03633 |
| cg10545682 | *TUFT1* | 7286 | 0.12 | 0.03633 |
| cg10570177 | *C9orf7* | 11094 | 0.12 | 0.03633 |
| cg10575414 | *RTDR1* | 27156 | 0.11 | 0.03633 |
| cg10575735 | *SSX4* | 6759 | -0.57 | 0.03633 |
| cg10575841 | *TMED7* | 51014 | 0.19 | 0.03633 |
| cg10586756 | *NUP93* | 9688 | -0.11 | 0.03633 |
| cg10594105 | *C19orf37* | 126299 | 0.12 | 0.03633 |
| cg10601168 | *CNKSR3* | 154043 | 0.12 | 0.03633 |
| cg10609615 | *TRAP1* | 10131 | 0.13 | 0.03633 |
| cg10628098 | *SCYL1* | 57410 | 0.11 | 0.03633 |
| cg10636246 | *AIM2* | 9447 | 0.58 | 0.03633 |
| cg10640379 | *LARP2* | 55132 | 0.18 | 0.03633 |
| cg10671066 | *SLAMF6* | 114836 | 0.36 | 0.03633 |
| cg10681065 | *TFR2* | 7036 | 0.78 | 0.03633 |
| cg10682057 | *ASAHL* | 27163 | 0.13 | 0.03633 |
| cg10688941 | *ASCC3L1* | 23020 | 0.13 | 0.03633 |
| cg10729531 | *TCFL5* | 10732 | 0.13 | 0.03633 |
| cg10730174 | *PRRX1* | 5396 | 0.16 | 0.03633 |
| cg10737521 | *KIAA0676* | 23061 | 0.13 | 0.03633 |
| cg10737625 | *THAP6* | 152815 | 0.08 | 0.03633 |
| cg10773309 | *BAI2* | 576 | 0.16 | 0.03633 |
| cg10783042 | *HDAC6* | 10013 | 0.22 | 0.03633 |
| cg10786880 | *PRKAA1* | 5562 | 0.12 | 0.03633 |
| cg10805667 | *FLJ20625* | 55004 | 0.11 | 0.03633 |
| cg10810921 | *CRLF1* | 9244 | 0.11 | 0.03633 |
| cg10833576 | *ANKRA2* | 57763 | 0.12 | 0.03633 |
| cg10839723 | *TBL1X* | 6907 | 0.23 | 0.03633 |
| cg10861017 | *ZNF420* | 147923 | 0.12 | 0.03633 |
| cg10861751 | *RGS1* | 5996 | 0.42 | 0.03633 |
| cg10882227 | *APLP2* | 334 | 0.19 | 0.03633 |
| cg10883352 |  | 5088 | -0.80 | 0.03633 |
| cg10887392 | *CTDSP1* | 58190 | 0.08 | 0.03633 |
| cg10906552 | *IL1RAP* | 3556 | 0.12 | 0.03633 |
| cg10909324 | *EHMT2* | 10919 | 0.16 | 0.03633 |
| cg10911660 | *ZNF585A* | 199704 | 0.15 | 0.03633 |
| cg10919204 | *CDH6* | 1004 | -0.32 | 0.03633 |
| cg10966500 | *TCHP* | 84260 | 0.14 | 0.03633 |
| cg10989517 | *SERGEF* | 26297 | -0.61 | 0.03633 |
| cg10996143 | *FKBP1A* | 2280 | 0.19 | 0.03633 |
| cg11007423 | *SPPL2A* | 84888 | 0.18 | 0.03633 |
| cg11016745 | *FLJ25444* | 254158 | 0.15 | 0.03633 |
| cg11025793 | *STX10* | 8677 | 0.17 | 0.03633 |
| cg11033833 | *GPR101* | 83550 | 0.18 | 0.03633 |
| cg11037148 | *HIST1H4J* | 8363 | 0.13 | 0.03633 |
| cg11049305 | *KIAA1166* | 55906 | 0.15 | 0.03633 |
| cg11053574 | *NT5C3L* | 115024 | 0.14 | 0.03633 |
| cg11058696 | *SNRPB2* | 6629 | 0.11 | 0.03633 |
| cg11064558 | *ZNF295* | 49854 | 0.15 | 0.03633 |
| cg11065518 | *MDH1B* | 130752 | 0.10 | 0.03633 |
| cg11081833 | *LGALS2* | 3957 | 0.56 | 0.03633 |
| cg11084035 | *GANAB* | 23193 | 0.11 | 0.03633 |
| cg11098259 | *AQP9* | 366 | 0.45 | 0.03633 |
| cg11099291 | *MGC14376* | 84981 | 0.17 | 0.03633 |
| cg11102794 | *LMO1* | 4004 | 0.18 | 0.03633 |
| cg11160820 | *CD58* | 965 | 0.17 | 0.03633 |
| cg11164347 | *TRAPPC3* | 27095 | 0.19 | 0.03633 |
| cg11166823 | *AGPAT3* | 56894 | 0.09 | 0.03633 |
| cg11171719 | *CTDSPL* | 10217 | 0.11 | 0.03633 |
| cg11187508 | *CWF19L1* | 55280 | 0.12 | 0.03633 |
| cg11208880 | *LRRC8D* | 55144 | 0.10 | 0.03633 |
| cg11233153 | *SLC10A3* | 8273 | 0.15 | 0.03633 |
| cg11251858 | *ESR1* | 2099 | 0.12 | 0.03633 |
| cg11264817 | *KIF1C* | 10749 | 0.15 | 0.03633 |
| cg11274211 | *C9orf42* | 116224 | 0.12 | 0.03633 |
| cg11276280 | *TBCC* | 6903 | 0.13 | 0.03633 |
| cg11286163 | *PSMA5* | 5686 | 0.11 | 0.03633 |
| cg11301598 | *OASL* | 8638 | 0.79 | 0.03633 |
| cg11311499 | *MSH2* | 4436 | 0.12 | 0.03633 |
| cg11321895 | *PLAG1* | 5324 | 0.08 | 0.03633 |
| cg11332950 | *AHSA2* | 130872 | 0.12 | 0.03633 |
| cg11353032 | *U2AF1L2* | 8233 | 0.15 | 0.03633 |
| cg11366015 | *C20orf27* | 54976 | 0.13 | 0.03633 |
| cg11368946 | *GCAT* | 23464 | 0.11 | 0.03633 |
| cg11375102 | *C16orf30* | 79652 | 0.35 | 0.03633 |
| cg11385473 | *IMMP1L* | 196294 | 0.11 | 0.03633 |
| cg11388238 | *KCTD18* | 130535 | 0.14 | 0.03633 |
| cg11399891 | *ZNF444* | 55311 | 0.12 | 0.03633 |
| cg11406695 | *C6orf125* | 84300 | 0.13 | 0.03633 |
| cg11414046 | *BRAF* | 673 | 0.16 | 0.03633 |
| cg11429658 | *ABCC13* | 150000 | -0.67 | 0.03633 |
| cg11480873 | *RAB7L1* | 8934 | 0.12 | 0.03633 |
| cg11481720 | *FLJ90396* | 163049 | 0.13 | 0.03633 |
| cg11481793 | *FLJ25476* | 149076 | 0.23 | 0.03633 |
| cg11492403 | *AARSD1* | 80755 | -0.76 | 0.03633 |
| cg11503011 | *EBF* | 1879 | 0.23 | 0.03633 |
| cg11526405 | *SH3GLB2* | 56904 | 0.16 | 0.03633 |
| cg11540997 | *DUOX2* | 50506 | -0.24 | 0.03633 |
| cg11542231 | *RPA2* | 6118 | 0.12 | 0.03633 |
| cg11572744 | *DPYSL3* | 1809 | 0.16 | 0.03633 |
| cg11609366 | *RPL8* | 6132 | 0.13 | 0.03633 |
| cg11625178 | *TMPO* | 7112 | 0.09 | 0.03633 |
| cg11625451 | *GAN* | 8139 | 0.15 | 0.03633 |
| cg11630242 | *AKAP10* | 11216 | 0.08 | 0.03633 |
| cg11632617 | *PPCDC* | 60490 | 0.17 | 0.03633 |
| cg11653271 | *POLR3K* | 51728 | 0.11 | 0.03633 |
| cg11662098 | *GOPC* | 57120 | 0.12 | 0.03633 |
| cg11675413 | *RNF113B* | 140432 | -0.83 | 0.03633 |
| cg11692477 | *SLC40A1* | 30061 | 0.13 | 0.03633 |
| cg11694641 | *SLC25A3* | 5250 | 0.09 | 0.03633 |
| cg11743795 | *SLMAP* | 7871 | 0.12 | 0.03633 |
| cg11752275 | *GNLY* | 10578 | 0.54 | 0.03633 |
| cg11765205 | *ARL11* | 115761 | 0.51 | 0.03633 |
| cg11766698 | *UQCRC2* | 7385 | 0.14 | 0.03633 |
| cg11768886 | *STK32B* | 55351 | 0.13 | 0.03633 |
| cg11808544 | *FKBP9L* | 360132 | 0.47 | 0.03633 |
| cg11822932 | *LMO2* | 4005 | 0.74 | 0.03633 |
| cg11832722 | *DSC3* | 1825 | 0.13 | 0.03633 |
| cg11835197 | *TFAP2E* | 339488 | -0.58 | 0.03633 |
| cg11854007 | *RP11-49G10.8* | 317716 | 0.37 | 0.03633 |
| cg11857445 | *AATF* | 26574 | 0.08 | 0.03633 |
| cg11871549 | *ZCCHC12* | 170261 | 0.12 | 0.03633 |
| cg11884546 | *ITGAX* | 3687 | 0.79 | 0.03633 |
| cg11890956 | *DSCR2* | 8624 | 0.18 | 0.03633 |
| cg11893323 | *C9orf74* | 81605 | 0.12 | 0.03633 |
| cg11916609 | *IL1RL1* | 9173 | 0.46 | 0.03633 |
| cg11917694 | *STAMBPL1* | 57559 | 0.11 | 0.03633 |
| cg11939496 | *CD244* | 51744 | 0.59 | 0.03633 |
| cg11970301 | *PPP4C* | 5531 | 0.10 | 0.03633 |
| cg11980188 | *CYP2R1* | 120227 | 0.13 | 0.03633 |
| cg11991627 | *C20orf96* | 140680 | 0.13 | 0.03633 |
| cg12002314 | *CHRAC1* | 54108 | 0.11 | 0.03633 |
| cg12014417 | *GPR109A* | 338442 | 0.42 | 0.03633 |
| cg12020639 | *AHCYL1* | 10768 | 0.11 | 0.03633 |
| cg12032116 | *DMXL1* | 1657 | 0.15 | 0.03633 |
| cg12034229 | *HUS1* | 3364 | 0.13 | 0.03633 |
| cg12051359 | *DEPDC6* | 64798 | 0.10 | 0.03633 |
| cg12052258 | *S100A11* | 6282 | 0.12 | 0.03633 |
| cg12073779 | *CRYGD* | 1421 | 0.15 | 0.03633 |
| cg12079362 | *ATP6V0B* | 533 | 0.13 | 0.03633 |
| cg12080812 | *FAM89B* | 23625 | -0.11 | 0.03633 |
| cg12091944 | *BARD1* | 580 | 0.19 | 0.03633 |
| cg12105450 | *CASP10* | 843 | 0.50 | 0.03633 |
| cg12120741 | *EDNRB* | 1910 | -0.53 | 0.03633 |
| cg12128839 | *HOXA5* | 3202 | -0.39 | 0.03633 |
| cg12133444 | *KCTD3* | 51133 | 0.15 | 0.03633 |
| cg12177677 | *PSCDBP* | 9595 | 0.67 | 0.03633 |
| cg12180123 | *PAPOLG* | 64895 | 0.11 | 0.03633 |
| cg12194493 | *PIGG* | 54872 | 0.13 | 0.03633 |
| cg12204727 | *COMMD4* | 54939 | 0.22 | 0.03633 |
| cg12206093 | *FKSG44* | 83786 | 0.10 | 0.03633 |
| cg12229172 | *MGST2* | 4258 | 0.10 | 0.03633 |
| cg12230010 | *EIF4EBP2* | 1979 | 0.16 | 0.03633 |
| cg12270520 | *ZNF517* | 340385 | 0.13 | 0.03633 |
| cg12304441 | *USP15* | 9958 | 0.16 | 0.03633 |
| cg12316147 | *GBA2* | 57704 | 0.14 | 0.03633 |
| cg12318316 | *NDUFB2* | 4708 | 0.09 | 0.03633 |
| cg12324831 | *VBP1* | 7411 | 0.13 | 0.03633 |
| cg12336877 | *MAN1B1* | 11253 | 0.11 | 0.03633 |
| cg12374577 | *UGT2A3* | 79799 | 0.64 | 0.03633 |
| cg12385425 | *TMEM17* | 200728 | 0.16 | 0.03633 |
| cg12393697 | *KIAA1946* | 165215 | 0.14 | 0.03633 |
| cg12398332 | *GPRC5B* | 51704 | 0.12 | 0.03633 |
| cg12400041 | *DST* | 667 | -0.57 | 0.03633 |
| cg12400881 | *PPL* | 5493 | 0.10 | 0.03633 |
| cg12405833 | *RUNDC2A* | 84127 | 0.16 | 0.03633 |
| cg12406460 | *ATP5L* | 10632 | 0.12 | 0.03633 |
| cg12417299 | *NEK4* | 6787 | 0.12 | 0.03633 |
| cg12424817 | *LAPTM4B* | 55353 | 0.12 | 0.03633 |
| cg12428416 | *GJA3* | 2700 | -0.74 | 0.03633 |
| cg12431699 | *CDK5RAP1* | 51654 | 0.10 | 0.03633 |
| cg12434779 | *ARHGAP6* | 395 | 0.12 | 0.03633 |
| cg12438666 | *C2orf15* | 150590 | 0.17 | 0.03633 |
| cg12467864 | *PRC1* | 9055 | 0.12 | 0.03633 |
| cg12515371 | *PPP1R16A* | 84988 | -0.11 | 0.03633 |
| cg12534466 | *FLJ20551* | 54977 | 0.14 | 0.03633 |
| cg12539975 | *PRDM12* | 59335 | 0.21 | 0.03633 |
| cg12554819 | *REV1L* | 51455 | 0.14 | 0.03633 |
| cg12558957 | *TOMM20* | 9804 | 0.12 | 0.03633 |
| cg12564962 | *DSCR6* | 53820 | -0.70 | 0.03633 |
| cg12570787 | *LOC133957* | 133957 | 0.18 | 0.03633 |
| cg12573766 | *RPS3* | 6188 | 0.15 | 0.03633 |
| cg12588301 | *C1orf42* | 54544 | -0.63 | 0.03633 |
| cg12594641 | *MGC52057* | 130574 | 0.11 | 0.03633 |
| cg12598178 | *UTS2R* | 2837 | -0.62 | 0.03633 |
| cg12628956 | *LOC317671* | 317671 | 0.16 | 0.03633 |
| cg12647643 | *PTPN13* | 5783 | 0.13 | 0.03633 |
| cg12680609 | *ZFP41* | 286128 | 0.13 | 0.03633 |
| cg12688043 | *MAP4* | 4134 | 0.15 | 0.03633 |
| cg12701101 | *DKFZp434N062* | 151313 | 0.15 | 0.03633 |
| cg12709412 | *C18orf8* | 29919 | 0.13 | 0.03633 |
| cg12722998 | *FLJ11305* | 55795 | 0.17 | 0.03633 |
| cg12723809 | *MGC26744* | 132989 | 0.15 | 0.03633 |
| cg12733079 | *ZNF277* | 11179 | 0.14 | 0.03633 |
| cg12757684 | *PLAGL1* | 5325 | -1.07 | 0.03633 |
| cg12781218 | *C7orf31* | 136895 | 0.09 | 0.03633 |
| cg12781568 | *WT1* | 7490 | -0.42 | 0.03633 |
| cg12784172 | *ZNF690* | 146050 | -0.79 | 0.03633 |
| cg12785689 | *ITGB4BP* | 3692 | 0.15 | 0.03633 |
| cg12787624 | *LIMA1* | 51474 | 0.10 | 0.03633 |
| cg12789833 | *FBXO22* | 26263 | 0.25 | 0.03633 |
| cg12792366 | *LRRFIP2* | 9209 | 0.12 | 0.03633 |
| cg12803724 | *C8orf70* | 51101 | 0.13 | 0.03633 |
| cg12823748 | *USP15* | 9958 | 0.11 | 0.03633 |
| cg12830829 | *TXNDC* | 81542 | 0.10 | 0.03633 |
| cg12839593 | *SIX1* | 6495 | 0.15 | 0.03633 |
| cg12858514 | *PADI3* | 51702 | -0.78 | 0.03633 |
| cg12914657 | *GIMAP1* | 170575 | 0.79 | 0.03633 |
| cg12916723 | *NKG7* | 4818 | 0.23 | 0.03633 |
| cg12973651 | *CNFN* | 84518 | 0.65 | 0.03633 |
| cg12997958 | *FLII* | 2314 | 0.19 | 0.03633 |
| cg12998491 | *FAM78A* | 286336 | 0.14 | 0.03633 |
| cg13021384 | *SLC27A2* | 11001 | 0.12 | 0.03633 |
| cg13031679 | *FLJ10560* | 55171 | 0.11 | 0.03633 |
| cg13038560 | *FLJ22555* | 79568 | -0.62 | 0.03633 |
| cg13043509 | *GIMAP5* | 55340 | 0.68 | 0.03633 |
| cg13052755 | *PVT1* | 5820 | -0.62 | 0.03633 |
| cg13059782 | *CNTNAP3* | 79937 | 0.25 | 0.03633 |
| cg13060997 | *CCNA1* | 8900 | 0.12 | 0.03633 |
| cg13064881 | *CDC16* | 8881 | 0.12 | 0.03633 |
| cg13084429 | *VWA2* | 340706 | 0.11 | 0.03633 |
| cg13120814 | *FLJ11000* | 55281 | 0.43 | 0.03633 |
| cg13128531 | *ZBTB33* | 10009 | 0.16 | 0.03633 |
| cg13149307 | *PLXDC2* | 84898 | 0.12 | 0.03633 |
| cg13151102 | *KCNJ4* | 3761 | 0.12 | 0.03633 |
| cg13173909 | *UGCGL1* | 56886 | 0.11 | 0.03633 |
| cg13174197 | *GNL3* | 26354 | 0.18 | 0.03633 |
| cg13221796 | *RB1* | 5925 | -0.52 | 0.03633 |
| cg13224710 | *TGIF* | 7050 | -0.68 | 0.03633 |
| cg13226591 | *MAGEB18* | 286514 | -0.64 | 0.03633 |
| cg13232900 | *ADAT1* | 23536 | 0.14 | 0.03633 |
| cg13236854 | *NAPA* | 8775 | 0.11 | 0.03633 |
| cg13283751 | *GPX5* | 2880 | 0.42 | 0.03633 |
| cg13311440 | *CD48* | 962 | 0.75 | 0.03633 |
| cg13332088 | *C14orf80* | 283643 | 0.13 | 0.03633 |
| cg13332130 | *RAB4B* | 53916 | 0.12 | 0.03633 |
| cg13351698 | *CBX3* | 11335 | 0.13 | 0.03633 |
| cg13362403 | *UBE2Z* | 65264 | 0.15 | 0.03633 |
| cg13378388 | *VEGFC* | 7424 | 0.12 | 0.03633 |
| cg13397365 | *MEA1* | 4201 | 0.14 | 0.03633 |
| cg13406950 | *GBP1* | 2633 | 0.53 | 0.03633 |
| cg13455369 | *FAM44A* | 259282 | 0.10 | 0.03633 |
| cg13467649 | *CCDC25* | 55246 | 0.10 | 0.03633 |
| cg13509147 | *CREB3L3* | 84699 | 0.41 | 0.03633 |
| cg13510327 | *NEU1* | 4758 | 0.11 | 0.03633 |
| cg13516796 | *ALG1* | 56052 | 0.10 | 0.03633 |
| cg13517305 | *CHCHD6* | 84303 | 0.16 | 0.03633 |
| cg13539030 | *ARL4C* | 10123 | 0.13 | 0.03633 |
| cg13543096 | *GDPD3* | 79153 | -0.13 | 0.03633 |
| cg13557178 | *EFHA1* | 221154 | 0.12 | 0.03633 |
| cg13563405 | *ASAH1* | 427 | 0.10 | 0.03633 |
| cg13564595 | *GRPEL1* | 80273 | 0.12 | 0.03633 |
| cg13576739 | *PFDN6* | 10471 | 0.14 | 0.03633 |
| cg13593287 | *SPTB* | 6710 | 0.67 | 0.03633 |
| cg13608094 | *CCND1* | 595 | -0.14 | 0.03633 |
| cg13615396 | *RACGAP1* | 29127 | 0.15 | 0.03633 |
| cg13652556 | *SEPX1* | 51734 | 0.15 | 0.03633 |
| cg13658777 | *PDHA1* | 5160 | 0.15 | 0.03633 |
| cg13668397 | *LIG1* | 3978 | 0.16 | 0.03633 |
| cg13670595 | *CDR2L* | 30850 | 0.11 | 0.03633 |
| cg13688966 | *TM4SF4* | 7104 | -0.38 | 0.03633 |
| cg13727946 | *MBD3L1* | 85509 | 0.51 | 0.03633 |
| cg13756879 | *IGF2* | 3481 | 0.17 | 0.03633 |
| cg13759143 | *EXPH5* | 23086 | 0.13 | 0.03633 |
| cg13763232 | *SLC6A6* | 6533 | -0.56 | 0.03633 |
| cg13794642 | *ZNF157* | 7712 | -0.13 | 0.03633 |
| cg13797031 | *NIPSNAP1* | 8508 | -0.49 | 0.03633 |
| cg13802364 | *RCOR2* | 283248 | 0.09 | 0.03633 |
| cg13828758 | *NDN* | 4692 | -0.51 | 0.03633 |
| cg13837202 | *PAGE5* | 90737 | -0.39 | 0.03633 |
| cg13842258 | *APBB2* | 323 | 0.12 | 0.03633 |
| cg13856728 | *CREBBP* | 1387 | 0.12 | 0.03633 |
| cg13878093 | *TBPL1* | 9519 | 0.13 | 0.03633 |
| cg13878641 | *GNAI3* | 2773 | 0.16 | 0.03633 |
| cg13899108 | *PDE4C* | 5143 | -0.43 | 0.03633 |
| cg13908523 | *PRKCD* | 5580 | 0.09 | 0.03633 |
| cg13910855 | *PBX2* | 5089 | 0.15 | 0.03633 |
| cg13912204 | *SLC19A2* | 10560 | 0.13 | 0.03633 |
| cg13927251 | *FLT4* | 2324 | 0.15 | 0.03633 |
| cg13958676 | *LGI3* | 203190 | 0.12 | 0.03633 |
| cg13982661 | *SC5DL* | 6309 | 0.17 | 0.03633 |
| cg13983578 | *DUSP4* | 1846 | 0.13 | 0.03633 |
| cg13992856 | *FLOT2* | 2319 | 0.13 | 0.03633 |
| cg14043602 | *OR1E1* | 8387 | 0.46 | 0.03633 |
| cg14056306 | *CDK3* | 1018 | -0.10 | 0.03633 |
| cg14060060 | *KIAA0251* | 23042 | 0.10 | 0.03633 |
| cg14072120 | *RAC2* | 5880 | 0.39 | 0.03633 |
| cg14074431 | *MAP3K10* | 4294 | 0.16 | 0.03633 |
| cg14101501 | *EHBP1* | 23301 | 0.13 | 0.03633 |
| cg14106263 | *RBMX2* | 51634 | 0.30 | 0.03633 |
| cg14106308 | *VEPH1* | 79674 | 0.18 | 0.03633 |
| cg14127336 | *TCL1A* | 8115 | -0.83 | 0.03633 |
| cg14127958 | *DIP13B* | 55198 | 0.10 | 0.03633 |
| cg14142521 | *TAF11* | 6882 | 0.10 | 0.03633 |
| cg14145762 | *FLJ10726* | 55216 | 0.21 | 0.03633 |
| cg14167017 | *UBAP1* | 51271 | 0.10 | 0.03633 |
| cg14172532 | *STK25* | 10494 | 0.11 | 0.03633 |
| cg14195915 | *SFRS4* | 6429 | 0.12 | 0.03633 |
| cg14205126 | *TMEM9B* | 56674 | 0.14 | 0.03633 |
| cg14213620 | *DFFB* | 1677 | 0.13 | 0.03633 |
| cg14243026 | *HMP19* | 51617 | 0.46 | 0.03633 |
| cg14252569 | *DACT1* | 51339 | 0.11 | 0.03633 |
| cg14271400 | *EPHB2* | 2048 | 0.12 | 0.03633 |
| cg14272175 | *PXK* | 54899 | 0.12 | 0.03633 |
| cg14284171 | *SSX4* | 6759 | -0.73 | 0.03633 |
| cg14290291 | *FLJ14346* | 80097 | -0.66 | 0.03633 |
| cg14296374 | *HSBP1* | 3281 | -0.64 | 0.03633 |
| cg14299177 | *MTFR1* | 9650 | 0.11 | 0.03633 |
| cg14303330 | *RPL22* | 6146 | 0.26 | 0.03633 |
| cg14318370 | *CHCHD5* | 84269 | 0.14 | 0.03633 |
| cg14325649 | *RBMS1* | 5937 | 0.13 | 0.03633 |
| cg14343062 | *ERCC6* | 2074 | 0.15 | 0.03633 |
| cg14346035 | *PPM2C* | 54704 | 0.11 | 0.03633 |
| cg14364739 | *NOSIP* | 51070 | 0.11 | 0.03633 |
| cg14375111 | *TMEM43* | 79188 | -0.81 | 0.03633 |
| cg14384940 | *C21orf81* | 114035 | -0.46 | 0.03633 |
| cg14452140 | *SEC14L2* | 23541 | 0.14 | 0.03633 |
| cg14458834 | *HOXB4* | 3214 | 0.15 | 0.03633 |
| cg14472778 | *MTHFR* | 4524 | -0.42 | 0.03633 |
| cg14474880 | *MTF2* | 22823 | 0.16 | 0.03633 |
| cg14490250 | *FAM96A* | 84191 | 0.14 | 0.03633 |
| cg14501253 | *FLJ36980* | 286032 | 0.12 | 0.03633 |
| cg14509125 | *FLJ20512* | 54958 | 0.14 | 0.03633 |
| cg14511156 | *OSCAR* | 126014 | 0.66 | 0.03633 |
| cg14517922 |  | 6922 | -0.08 | 0.03633 |
| cg14528319 | *GIPC1* | 10755 | -0.82 | 0.03633 |
| cg14541950 | *HIST3H2BB* | 128312 | 0.11 | 0.03633 |
| cg14556618 | *IGF2R* | 3482 | 0.12 | 0.03633 |
| cg14576628 | *PRMT1* | 3276 | 0.11 | 0.03633 |
| cg14611112 | *LCN6* | 158062 | 0.65 | 0.03633 |
| cg14665389 | *TCF7L2* | 6934 | 0.12 | 0.03633 |
| cg14689355 | *ACVR2A* | 92 | 0.15 | 0.03633 |
| cg14700707 | *NOTCH4* | 4855 | 0.74 | 0.03633 |
| cg14741666 | *ZNHIT3* | 9326 | 0.09 | 0.03633 |
| cg14776962 | *HIST1H2BH* | 8345 | 0.18 | 0.03633 |
| cg14777972 | *SEC10L1* | 10640 | 0.12 | 0.03633 |
| cg14789590 | *BPIL2* | 254240 | -0.13 | 0.03633 |
| cg14789804 | *OS9* | 10956 | 0.12 | 0.03633 |
| cg14817542 | *HIF1AN* | 55662 | 0.11 | 0.03633 |
| cg14831838 | *CDK5R2* | 8941 | 0.09 | 0.03633 |
| cg14838256 | *SRD5A2L* | 79644 | 0.12 | 0.03633 |
| cg14844130 | *VPREB1* | 7441 | 0.61 | 0.03633 |
| cg14849559 | *TMBIM1* | 64114 | 0.18 | 0.03633 |
| cg14859749 | *SF3A1* | 10291 | 0.10 | 0.03633 |
| cg14869028 | *TINAGL1* | 64129 | 0.56 | 0.03633 |
| cg14886269 | *TNFRSF18* | 8784 | 0.83 | 0.03633 |
| cg14896516 | *CRHR2* | 1395 | 0.11 | 0.03633 |
| cg14898779 | *STK31* | 56164 | -0.40 | 0.03633 |
| cg14916288 | *CCL23* | 6368 | 0.51 | 0.03633 |
| cg14920334 | *GALNT8* | 26290 | 0.42 | 0.03633 |
| cg14931645 | *ZNF672* | 79894 | 0.18 | 0.03633 |
| cg14944362 | *PDYN* | 5173 | 0.51 | 0.03633 |
| cg14949649 | *KCTD10* | 83892 | 0.10 | 0.03633 |
| cg14964658 | *PEX19* | 5824 | 0.12 | 0.03633 |
| cg14993293 | *CETN3* | 1070 | 0.12 | 0.03633 |
| cg15002187 | *SLC37A3* | 84255 | 0.18 | 0.03633 |
| cg15009813 | *TNFSF12* | 8742 | 0.16 | 0.03633 |
| cg15016234 | *TRIM62* | 55223 | 0.13 | 0.03633 |
| cg15039399 | *CDK2AP2* | 10263 | 0.13 | 0.03633 |
| cg15043975 | *RASSF1* | 11186 | 0.09 | 0.03633 |
| cg15054260 | *RIPK2* | 8767 | 0.15 | 0.03633 |
| cg15060177 | *SACM1L* | 22908 | 0.09 | 0.03633 |
| cg15091337 | *POLE4* | 56655 | 0.17 | 0.03633 |
| cg15103675 | *UAP1* | 6675 | 0.16 | 0.03633 |
| cg15119375 | *MGC3020* | 79014 | 0.12 | 0.03633 |
| cg15125424 | *SOCS2* | 8835 | 0.14 | 0.03633 |
| cg15134628 | *MAPK12* | 6300 | 0.12 | 0.03633 |
| cg15142488 | *LYPD4* | 147719 | -0.81 | 0.03633 |
| cg15143643 | *FBLN1* | 2192 | -0.51 | 0.03633 |
| cg15173196 | *UBE2T* | 29089 | 0.14 | 0.03633 |
| cg15185001 | *LSR* | 51599 | 0.11 | 0.03633 |
| cg15195276 | *EDG2* | 1902 | 0.12 | 0.03633 |
| cg15205507 | *ZNF331* | 55422 | 0.19 | 0.03633 |
| cg15209169 | *RABGAP1* | 23637 | -0.28 | 0.03633 |
| cg15213605 | *SF3B3* | 23450 | 0.11 | 0.03633 |
| cg15253243 | *CHPF* | 79586 | 0.10 | 0.03633 |
| cg15262516 | *COL4A2* | 1284 | 0.24 | 0.03633 |
| cg15271594 | *RPL29* | 6159 | 0.12 | 0.03633 |
| cg15299721 | *LOC133308* | 133308 | 0.14 | 0.03633 |
| cg15301525 | *ATP6V0D1* | 9114 | 0.10 | 0.03633 |
| cg15302379 | *KAZALD1* | 81621 | -0.81 | 0.03633 |
| cg15309236 | *BHLHB9* | 80823 | 0.13 | 0.03633 |
| cg15312298 | *FAM84B* | 157638 | 0.35 | 0.03633 |
| cg15321195 | *AGPAT3* | 56894 | 0.14 | 0.03633 |
| cg15337055 | *FOS* | 2353 | 0.12 | 0.03633 |
| cg15342384 | *HNRPH3* | 3189 | 0.11 | 0.03633 |
| cg15352829 | *PLD4* | 122618 | 0.76 | 0.03633 |
| cg15371509 | *C1orf60* | 65123 | 0.13 | 0.03633 |
| cg15383120 | *DUSP22* | 56940 | -0.59 | 0.03633 |
| cg15408454 | *MAGEA6* | 4105 | -0.85 | 0.03633 |
| cg15417900 | *TMEM4* | 10330 | 0.13 | 0.03633 |
| cg15431576 | *C10orf83* | 118812 | 0.17 | 0.03633 |
| cg15443822 | *ATP2A3* | 489 | 0.11 | 0.03633 |
| cg15444978 | *MARK3* | 4140 | 0.16 | 0.03633 |
| cg15460516 | *TNFRSF12A* | 51330 | 0.12 | 0.03633 |
| cg15484375 | *SAA1* | 6288 | 0.74 | 0.03633 |
| cg15502446 | *HSPC138* | 51501 | 0.11 | 0.03633 |
| cg15517965 | *MGC3123* | 79089 | 0.12 | 0.03633 |
| cg15541315 | *REXO2* | 25996 | 0.11 | 0.03633 |
| cg15547662 | *PAFAH2* | 5051 | 0.15 | 0.03633 |
| cg15552238 | *EMR3* | 84658 | 0.66 | 0.03633 |
| cg15558658 | *KIAA1279* | 26128 | 0.09 | 0.03633 |
| cg15560112 | *MYBPC2* | 4606 | 0.14 | 0.03633 |
| cg15602735 | *MAGEC2* | 51438 | -0.58 | 0.03633 |
| cg15604467 | *POU4F1* | 5457 | 0.15 | 0.03633 |
| cg15641998 | *DHRS8* | 51170 | 0.10 | 0.03633 |
| cg15645309 | *BATF* | 10538 | 0.40 | 0.03633 |
| cg15645784 | *FLJ22386* | 79641 | 0.16 | 0.03633 |
| cg15652797 | *TP53AP1* | 11257 | 0.11 | 0.03633 |
| cg15677294 | *TUBB4* | 10382 | -0.68 | 0.03633 |
| cg15690721 | *ZNF85* | 7639 | 0.19 | 0.03633 |
| cg15691199 | *CEBPE* | 1053 | 0.46 | 0.03633 |
| cg15700739 | *HOXC5* | 3222 | 0.15 | 0.03633 |
| cg15701111 | *KRTHB1* | 3887 | -0.66 | 0.03633 |
| cg15701622 | *MCRS1* | 10445 | 0.12 | 0.03633 |
| cg15705469 | *ZNF71* | 58491 | 0.14 | 0.03633 |
| cg15736338 | *FAM19A2* | 338811 | 0.13 | 0.03633 |
| cg15739581 | *GALNT3* | 2591 | 0.43 | 0.03633 |
| cg15746719 | *DAPK1* | 1612 | 0.14 | 0.03633 |
| cg15772393 | *SAP30* | 8819 | 0.13 | 0.03633 |
| cg15781316 | *SHFM1* | 7979 | 0.17 | 0.03633 |
| cg15781625 | *UCK2* | 7371 | 0.21 | 0.03633 |
| cg15797414 | *HIST1H3B* | 8358 | 0.11 | 0.03633 |
| cg15799959 | *ZNF248* | 57209 | 0.13 | 0.03633 |
| cg15821095 | *RBM15B* | 29890 | -0.74 | 0.03633 |
| cg15822411 | *MGC34732* | 220047 | -0.77 | 0.03633 |
| cg15846718 | *COX7A2* | 1347 | 0.15 | 0.03633 |
| cg15851800 | *LOC133619* | 133619 | 0.10 | 0.03633 |
| cg15852891 | *OTP* | 23440 | 0.11 | 0.03633 |
| cg15877314 | *GNAI2* | 2771 | 0.15 | 0.03633 |
| cg15895197 | *EMILIN1* | 11117 | 0.62 | 0.03633 |
| cg15898840 | *IGFBP3* | 3486 | 0.10 | 0.03633 |
| cg15945417 | *LLGL1* | 3996 | 0.10 | 0.03633 |
| cg15955341 | *GSTM4* | 2948 | 0.12 | 0.03633 |
| cg15965055 | *C1orf32* | 387597 | 0.10 | 0.03633 |
| cg16000888 | *C11orf35* | 256329 | 0.16 | 0.03633 |
| cg16005443 | *LILRB3* | 11025 | 0.78 | 0.03633 |
| cg16013246 | *RCC1* | 1104 | 0.13 | 0.03633 |
| cg16026550 | *SORL1* | 6653 | 0.15 | 0.03633 |
| cg16034046 | *KIAA0182* | 23199 | 0.14 | 0.03633 |
| cg16077929 | *CDKL1* | 8814 | -0.55 | 0.03633 |
| cg16094954 | *BCL3* | 602 | 0.28 | 0.03633 |
| cg16108132 | *ANXA7* | 310 | 0.22 | 0.03633 |
| cg16112050 | *ENOSF1* | 55556 | -0.79 | 0.03633 |
| cg16120811 | *WEE1* | 7465 | 0.14 | 0.03633 |
| cg16124337 | *UBE2J1* | 51465 | 0.12 | 0.03633 |
| cg16128363 | *DLGAP1* | 9229 | -0.61 | 0.03633 |
| cg16182127 | *SASS6* | 163786 | 0.12 | 0.03633 |
| cg16191009 | *CPNE9* | 151835 | -0.60 | 0.03633 |
| cg16205058 | *OSGEP* | 55644 | 0.15 | 0.03633 |
| cg16243646 | *PLA2G12A* | 81579 | 0.12 | 0.03633 |
| cg16253168 | *NOL7* | 51406 | 0.14 | 0.03633 |
| cg16253412 | *TPST1* | 8460 | 0.16 | 0.03633 |
| cg16264526 | *EDNRA* | 1909 | -0.30 | 0.03633 |
| cg16267491 | *H2AFZ* | 3015 | 0.13 | 0.03633 |
| cg16276070 | *ZNF550* | 162972 | 0.10 | 0.03633 |
| cg16280313 | *PEX16* | 9409 | 0.12 | 0.03633 |
| cg16293105 | *SIGLEC6* | 946 | 0.11 | 0.03633 |
| cg16298264 | *LOC153364* | 153364 | 0.13 | 0.03633 |
| cg16299341 | *OSBPL9* | 114883 | 0.10 | 0.03633 |
| cg16313587 | *BRF2* | 55290 | 0.15 | 0.03633 |
| cg16335762 | *CMTM3* | 123920 | 0.16 | 0.03633 |
| cg16357921 | *CCRK* | 23552 | 0.13 | 0.03633 |
| cg16359129 | *C1orf111* | 284680 | -0.14 | 0.03633 |
| cg16361890 | *LTC4S* | 4056 | 0.35 | 0.03633 |
| cg16372520 | *NRXN3* | 9369 | 0.21 | 0.03633 |
| cg16381752 | *TOR1AIP2* | 163590 | 0.15 | 0.03633 |
| cg16405575 | *TSPAN9* | 10867 | 0.14 | 0.03633 |
| cg16409869 | *NDUFC2* | 4718 | 0.15 | 0.03633 |
| cg16413535 | *TKTL2* | 84076 | -0.53 | 0.03633 |
| cg16414852 | *SULT1B1* | 27284 | 0.17 | 0.03633 |
| cg16438432 | *HNRPD* | 3184 | 0.18 | 0.03633 |
| cg16468729 | *IL8* | 3576 | 0.26 | 0.03633 |
| cg16481024 | *KLF10* | 7071 | 0.11 | 0.03633 |
| cg16515820 | *ZBTB20* | 26137 | 0.15 | 0.03633 |
| cg16517394 | *TNFSF4* | 7292 | 0.53 | 0.03633 |
| cg16519742 | *ZIM2* | 23619 | -0.61 | 0.03633 |
| cg16521836 | *KIAA1407* | 57577 | 0.10 | 0.03633 |
| cg16534233 | *FAM80A* | 284716 | 0.12 | 0.03633 |
| cg16543294 | *ASB6* | 140459 | 0.11 | 0.03633 |
| cg16547450 | *TTC10* | 8100 | 0.14 | 0.03633 |
| cg16574065 | *RAD52B* | 201299 | 0.10 | 0.03633 |
| cg16588061 | *CTSC* | 1075 | 0.13 | 0.03633 |
| cg16616325 | *TSNAX* | 7257 | 0.10 | 0.03633 |
| cg16625125 | *NUDT8* | 254552 | 0.12 | 0.03633 |
| cg16625216 | *GAK* | 2580 | 0.15 | 0.03633 |
| cg16636110 | *ZNF264* | 9422 | -0.60 | 0.03633 |
| cg16641915 | *CAPN5* | 726 | 0.12 | 0.03633 |
| cg16652639 | *TM2D2* | 83877 | 0.14 | 0.03633 |
| cg16682903 | *ACVR1* | 90 | 0.35 | 0.03633 |
| cg16692277 | *GUCY1B2* | 2974 | 0.60 | 0.03633 |
| cg16701105 | *DENND4A* | 10260 | 0.13 | 0.03633 |
| cg16705481 | *TRAPPC3* | 27095 | 0.19 | 0.03633 |
| cg16711185 | *SHC4* | 399694 | 0.13 | 0.03633 |
| cg16718891 | *ITGA5* | 3678 | 0.09 | 0.03633 |
| cg16719404 | *CD2* | 914 | 0.39 | 0.03633 |
| cg16720880 | *CHMP5* | 51510 | 0.17 | 0.03633 |
| cg16722118 | *KIAA0738* | 9747 | -0.47 | 0.03633 |
| cg16732901 | *FLJ35784* | 374877 | 0.47 | 0.03633 |
| cg16738336 | *SKIV2L2* | 23517 | 0.14 | 0.03633 |
| cg16739396 | *ATG4C* | 84938 | 0.15 | 0.03633 |
| cg16740543 | *PHF10* | 55274 | 0.11 | 0.03633 |
| cg16778809 | *ADAM23* | 8745 | 0.19 | 0.03633 |
| cg16786117 | *VSIG4* | 11326 | -0.40 | 0.03633 |
| cg16866038 | *ARCN1* | 372 | 0.15 | 0.03633 |
| cg16870320 | *GALNT4* | 8693 | 0.10 | 0.03633 |
| cg16886188 | *ADRA2A* | 150 | 0.12 | 0.03633 |
| cg16893574 | *CALB2* | 794 | -0.64 | 0.03633 |
| cg16907024 | *TCEB3C* | 162699 | -0.44 | 0.03633 |
| cg16907167 | *TGOLN2* | 10618 | 0.16 | 0.03633 |
| cg16913124 | *HYAL3* | 8372 | -0.13 | 0.03633 |
| cg16927136 | *RPL35A* | 6165 | 0.16 | 0.03633 |
| cg16948369 | *KLHL4* | 56062 | 0.30 | 0.03633 |
| cg16957313 | *DUSP1* | 1843 | 0.13 | 0.03633 |
| cg16961092 | *KIAA1737* | 85457 | 0.14 | 0.03633 |
| cg16967583 | *AGXT* | 189 | 0.52 | 0.03633 |
| cg16970232 | *APC* | 324 | 0.12 | 0.03633 |
| cg16979445 | *NRM* | 11270 | -0.58 | 0.03633 |
| cg16983159 | *LOC340061* | 340061 | 0.45 | 0.03633 |
| cg16993794 | *SLC2A11* | 66035 | 0.15 | 0.03633 |
| cg17004373 | *GNL2* | 29889 | 0.14 | 0.03633 |
| cg17017278 | *TRUB1* | 142940 | 0.14 | 0.03633 |
| cg17022914 | *BATF2* | 116071 | 0.84 | 0.03633 |
| cg17026542 | *OR2A4* | 79541 | -0.63 | 0.03633 |
| cg17051623 | *DDEFL1* | 55616 | 0.17 | 0.03633 |
| cg17055734 | *CRYGA* | 1418 | -0.13 | 0.03633 |
| cg17066382 | *LMBR1L* | 55716 | 0.15 | 0.03633 |
| cg17083925 | *TGM2* | 7052 | -0.62 | 0.03633 |
| cg17127769 | *LCP2* | 3937 | 0.54 | 0.03633 |
| cg17155596 | *STAMBPL1* | 57559 | 0.14 | 0.03633 |
| cg17162271 | *GPSN2* | 9524 | 0.10 | 0.03633 |
| cg17165760 | *ANKRD26* | 22852 | 0.13 | 0.03633 |
| cg17169998 | *MLC1* | 23209 | 0.89 | 0.03633 |
| cg17176732 | *OXR1* | 55074 | 0.16 | 0.03633 |
| cg17178888 | *FLJ14166* | 79616 | 0.13 | 0.03633 |
| cg17189058 | *FLJ20558* | 54980 | 0.15 | 0.03633 |
| cg17241310 | *BARHL2* | 343472 | 0.41 | 0.03633 |
| cg17280624 | *PGRMC2* | 10424 | 0.16 | 0.03633 |
| cg17333479 | *ZNF571* | 51276 | 0.12 | 0.03633 |
| cg17349199 | *C10orf82* | 143379 | -0.67 | 0.03633 |
| cg17354052 | *UBPH* | 56061 | 0.16 | 0.03633 |
| cg17367215 | *SUPT5H* | 6829 | 0.15 | 0.03633 |
| cg17398003 | *FLJ44216* | 375484 | 0.18 | 0.03633 |
| cg17408647 | *FLJ10803* | 55744 | 0.10 | 0.03633 |
| cg17425224 | *PARK7* | 11315 | 0.17 | 0.03633 |
| cg17439480 | *LOC203427* | 203427 | 0.13 | 0.03633 |
| cg17454897 | *ADAMTS10* | 81794 | 0.12 | 0.03633 |
| cg17465219 | *LOC124491* | 124491 | 0.11 | 0.03633 |
| cg17470637 | *MARCH2* | 51257 | 0.20 | 0.03633 |
| cg17482740 | *DNMT3B* | 1789 | -0.63 | 0.03633 |
| cg17497271 | *GPR176* | 11245 | 0.14 | 0.03633 |
| cg17498321 | *NOTCH3* | 4854 | 0.12 | 0.03633 |
| cg17505757 | *NUP88* | 4927 | 0.20 | 0.03633 |
| cg17552093 | *SIRT7* | 51547 | 0.13 | 0.03633 |
| cg17565490 | *CENPB* | 1059 | 0.15 | 0.03633 |
| cg17567317 | *POLR2F* | 5435 | 0.11 | 0.03633 |
| cg17582250 | *RCD-8* | 23644 | 0.15 | 0.03633 |
| cg17606683 | *HSPC138* | 51501 | 0.10 | 0.03633 |
| cg17607231 | *SP140* | 11262 | 0.86 | 0.03633 |
| cg17610929 | *ACCN4* | 55515 | 0.75 | 0.03633 |
| cg17614750 | *P4HB* | 5034 | 0.10 | 0.03633 |
| cg17638137 | *TCERG1* | 10915 | 0.12 | 0.03633 |
| cg17638468 | *CD200R1* | 131450 | 0.62 | 0.03633 |
| cg17647273 | *UPB1* | 51733 | -0.79 | 0.03633 |
| cg17661881 | *PARP1* | 142 | 0.13 | 0.03633 |
| cg17662177 | *PFC* | 5199 | 0.67 | 0.03633 |
| cg17693957 | *TCN2* | 6948 | 0.41 | 0.03633 |
| cg17694877 | *DIAPH2* | 1730 | 0.10 | 0.03633 |
| cg17732044 | *PEX11A* | 8800 | 0.14 | 0.03633 |
| cg17745122 | *IRX3* | 79191 | 0.10 | 0.03633 |
| cg17747332 | *SMARCA4* | 6597 | 0.15 | 0.03633 |
| cg17758721 | *SNRPB* | 6628 | 0.12 | 0.03633 |
| cg17775235 | *NPTX1* | 4884 | 0.14 | 0.03633 |
| cg17775283 | *UBE1* | 7317 | 0.18 | 0.03633 |
| cg17775713 | *TF* | 7018 | -0.55 | 0.03633 |
| cg17791766 | *HSPBP1* | 23640 | 0.14 | 0.03633 |
| cg17804987 | *MAPKAPK3* | 7867 | 0.12 | 0.03633 |
| cg17808849 | *HERPUD1* | 9709 | 0.12 | 0.03633 |
| cg17820591 | *ENO3* | 2027 | 0.12 | 0.03633 |
| cg17836145 | *VNN2* | 8875 | 0.75 | 0.03633 |
| cg17838765 | *ALKBH3* | 221120 | 0.14 | 0.03633 |
| cg17843759 | *ELL* | 8178 | 0.12 | 0.03633 |
| cg17851105 | *UNKL* | 64718 | 0.16 | 0.03633 |
| cg17853587 | *NDST3* | 9348 | 0.10 | 0.03633 |
| cg17858663 | *C16orf60* | 55839 | 0.13 | 0.03633 |
| cg17863168 | *APRT* | 353 | 0.14 | 0.03633 |
| cg17870792 | *LPHN2* | 23266 | 0.11 | 0.03633 |
| cg17875156 | *NTN1* | 9423 | 0.12 | 0.03633 |
| cg17877494 | *ACAA1* | 30 | 0.11 | 0.03633 |
| cg17886204 | *DKFZp434I1020* | 196968 | 0.11 | 0.03633 |
| cg17889912 | *TSPYL2* | 64061 | 0.18 | 0.03633 |
| cg17896249 | *E2F4* | 1874 | 0.16 | 0.03633 |
| cg17918201 | *ABI1* | 10006 | 0.11 | 0.03633 |
| cg17966192 | *SULT1C2* | 27233 | 0.52 | 0.03633 |
| cg17977409 | *SH3GL2* | 6456 | 0.14 | 0.03633 |
| cg17980508 | *IFI44L* | 10964 | 0.48 | 0.03633 |
| cg17991347 | *MAGED1* | 9500 | 0.14 | 0.03633 |
| cg18001427 | *C21orf6* | 10069 | 0.14 | 0.03633 |
| cg18009798 | *FEN1* | 2237 | 0.12 | 0.03633 |
| cg18015677 | *FLJ12571* | 79989 | 0.12 | 0.03633 |
| cg18020749 | *LRRC37A* | 9884 | -0.23 | 0.03633 |
| cg18022193 | *C19orf22* | 91300 | 0.16 | 0.03633 |
| cg18036707 | *TBCE* | 6905 | 0.17 | 0.03633 |
| cg18055007 | *DDAH2* | 23564 | 0.14 | 0.03633 |
| cg18058833 | *FAM86A* | 196483 | 0.57 | 0.03633 |
| cg18071588 | *DIDO1* | 11083 | 0.15 | 0.03633 |
| cg18089145 | *SEC23A* | 10484 | 0.12 | 0.03633 |
| cg18112005 | *C14orf80* | 283643 | 0.13 | 0.03633 |
| cg18112163 | *STAT3* | 6774 | 0.16 | 0.03633 |
| cg18119529 | *ZNF114* | 163071 | 0.13 | 0.03633 |
| cg18130076 | *RBM14* | 10432 | 0.10 | 0.03633 |
| cg18135555 | *PIWIL2* | 55124 | -0.68 | 0.03633 |
| cg18146152 | *GNPTG* | 84572 | -0.36 | 0.03633 |
| cg18152712 | *E2F3* | 1871 | 0.14 | 0.03633 |
| cg18159180 | *CUL7* | 9820 | -0.51 | 0.03633 |
| cg18174542 | *CSDC2* | 27254 | 0.70 | 0.03633 |
| cg18178963 | *NDUFB6* | 4712 | 0.12 | 0.03633 |
| cg18181070 | *HTRA2* | 27429 | 0.14 | 0.03633 |
| cg18182038 | *BSDC1* | 55108 | 0.19 | 0.03633 |
| cg18201077 | *RSAD2* | 91543 | 0.55 | 0.03633 |
| cg18202456 | *KLF17* | 128209 | -0.89 | 0.03633 |
| cg18206040 | *DNAJA2* | 10294 | 0.12 | 0.03633 |
| cg18208268 | *RPL13A* | 23521 | 0.12 | 0.03633 |
| cg18217172 | *REPS1* | 85021 | 0.12 | 0.03633 |
| cg18219418 | *PARP6* | 56965 | -0.71 | 0.03633 |
| cg18222235 | *FBXW8* | 26259 | 0.13 | 0.03633 |
| cg18239702 | *NHS* | 4810 | 0.11 | 0.03633 |
| cg18247054 | *RNASET2* | 8635 | 0.13 | 0.03633 |
| cg18263030 | *MARK3* | 4140 | 0.13 | 0.03633 |
| cg18273566 | *LOC148898* | 148898 | 0.10 | 0.03633 |
| cg18277508 | *SLC39A8* | 64116 | 0.23 | 0.03633 |
| cg18289259 | *TIMM8A* | 1678 | 0.15 | 0.03633 |
| cg18302652 | *IL8* | 3576 | 0.35 | 0.03633 |
| cg18328334 | *TNS1* | 7145 | 0.37 | 0.03633 |
| cg18335933 | *WRNIP1* | 56897 | 0.11 | 0.03633 |
| cg18342900 | *LOC388272* | 388272 | 0.18 | 0.03633 |
| cg18343292 | *MS4A7* | 58475 | 0.41 | 0.03633 |
| cg18350391 | *IL32* | 9235 | -0.15 | 0.03633 |
| cg18350458 | *EIF4EBP1* | 1978 | 0.13 | 0.03633 |
| cg18359921 | *MCCC2* | 64087 | 0.11 | 0.03633 |
| cg18387216 | *HDAC2* | 3066 | 0.15 | 0.03633 |
| cg18394496 | *LOC374395* | 374395 | 0.11 | 0.03633 |
| cg18396865 | *SNX6* | 58533 | 0.22 | 0.03633 |
| cg18399183 | *METTL7A* | 25840 | 0.29 | 0.03633 |
| cg18403361 | *CLEC14A* | 161198 | 0.14 | 0.03633 |
| cg18414741 | *ZNF77* | 58492 | 0.09 | 0.03633 |
| cg18428217 | *C7orf26* | 79034 | 0.10 | 0.03633 |
| cg18428373 | *FRMPD1* | 22844 | 0.17 | 0.03633 |
| cg18452600 | *MSRA* | 4482 | 0.16 | 0.03633 |
| cg18459342 | *TPD52* | 7163 | 0.21 | 0.03633 |
| cg18474135 | *CDC26* | 246184 | 0.14 | 0.03633 |
| cg18485154 | *TTLL4* | 9654 | 0.13 | 0.03633 |
| cg18487515 | *ZNF342* | 162979 | 0.12 | 0.03633 |
| cg18489440 | *KIAA0746* | 23231 | 0.16 | 0.03633 |
| cg18504532 | *RABGAP1L* | 9910 | 0.13 | 0.03633 |
| cg18507125 | *RPL36A* | 6173 | 0.14 | 0.03633 |
| cg18579447 | *MAP2K1IP1* | 8649 | 0.14 | 0.03633 |
| cg18601426 | *PTPRN2* | 5799 | -0.50 | 0.03633 |
| cg18606310 | *PYGB* | 5834 | 0.12 | 0.03633 |
| cg18638253 | *ACVR1C* | 130399 | 0.20 | 0.03633 |
| cg18641050 | *BLOC1S2* | 282991 | 0.19 | 0.03633 |
| cg18675600 | *PTP4A3* | 11156 | 0.18 | 0.03633 |
| cg18676679 | *BANP* | 54971 | 0.14 | 0.03633 |
| cg18679190 | *MFAP2* | 4237 | -0.15 | 0.03633 |
| cg18680021 | *OTUB2* | 78990 | 0.12 | 0.03633 |
| cg18690395 | *CSTF2T* | 23283 | 0.11 | 0.03633 |
| cg18700967 | *SSH2* | 85464 | 0.13 | 0.03633 |
| cg18710985 | *C20orf23* | 55614 | 0.14 | 0.03633 |
| cg18712119 | *TNFAIP1* | 7126 | 0.13 | 0.03633 |
| cg18717447 | *LOC159090* | 159090 | 0.22 | 0.03633 |
| cg18722841 | *PHOX2A* | 401 | 0.11 | 0.03633 |
| cg18731789 | *FKBP2* | 2286 | 0.16 | 0.03633 |
| cg18741908 | *GPR160* | 26996 | 0.13 | 0.03633 |
| cg18750756 | *MGC29671* | 201305 | 0.73 | 0.03633 |
| cg18755204 | *FAM45A* | 404636 | 0.20 | 0.03633 |
| cg18776764 | *BCL6* | 604 | 0.12 | 0.03633 |
| cg18780401 | *OTUD5* | 55593 | 0.13 | 0.03633 |
| cg18794577 | *GRIN3A* | 116443 | 0.11 | 0.03633 |
| cg18795395 | *GNG12* | 55970 | 0.17 | 0.03633 |
| cg18801292 | *SUPT16H* | 11198 | 0.14 | 0.03633 |
| cg18851831 | *DYRK1B* | 9149 | 0.11 | 0.03633 |
| cg18877506 | *PDPN* | 10630 | 0.09 | 0.03633 |
| cg18887228 | *PHF3* | 23469 | -0.13 | 0.03633 |
| cg18895155 | *ZNF498* | 221785 | 0.13 | 0.03633 |
| cg18896687 | *MAFK* | 7975 | 0.12 | 0.03633 |
| cg18902203 | *TFDP1* | 7027 | -0.95 | 0.03633 |
| cg18905952 | *NRBP1* | 29959 | 0.13 | 0.03633 |
| cg18907029 | *SYNGR2* | 9144 | 0.10 | 0.03633 |
| cg18908499 | *C1orf150* | 148823 | 0.36 | 0.03633 |
| cg18909638 | *MIPOL1* | 145282 | 0.17 | 0.03633 |
| cg18915437 | *USP15* | 9958 | 0.12 | 0.03633 |
| cg18938150 | *LSM12* | 124801 | 0.09 | 0.03633 |
| cg18960218 | *SLC7A7* | 9056 | 0.47 | 0.03633 |
| cg18986273 | *FAM53C* | 51307 | 0.24 | 0.03633 |
| cg18987220 | *RAB32* | 10981 | -0.56 | 0.03633 |
| cg19028369 | *C3orf19* | 51244 | 0.09 | 0.03633 |
| cg19042062 | *KCNJ2* | 3759 | 0.14 | 0.03633 |
| cg19055639 | *ARMCX2* | 9823 | 0.18 | 0.03633 |
| cg19070338 | *ASNSD1* | 54529 | 0.10 | 0.03633 |
| cg19104072 | *B3GNT6* | 11041 | 0.11 | 0.03633 |
| cg19118077 | *AKR1C3* | 8644 | 0.38 | 0.03633 |
| cg19125323 | *SFRS2* | 6427 | 0.11 | 0.03633 |
| cg19133618 | *CDKN2B* | 1030 | 0.16 | 0.03633 |
| cg19168631 | *DAAM1* | 23002 | 0.09 | 0.03633 |
| cg19190714 | *GMPR* | 2766 | 0.14 | 0.03633 |
| cg19202384 | *PYCR1* | 5831 | -0.46 | 0.03633 |
| cg19207856 | *DSCR5* | 51227 | 0.13 | 0.03633 |
| cg19208681 | *RAD51C* | 5889 | -0.56 | 0.03633 |
| cg19224164 | *C4orf9* | 8602 | 0.13 | 0.03633 |
| cg19233472 | *FOXI1* | 2299 | -0.55 | 0.03633 |
| cg19262019 | *HNRPM* | 4670 | 0.10 | 0.03633 |
| cg19278165 | *DSCR1* | 1827 | 0.16 | 0.03633 |
| cg19318511 | *C9orf30* | 91283 | 0.15 | 0.03633 |
| cg19324791 | *CEECAM1* | 51148 | 0.13 | 0.03633 |
| cg19353006 | *TUSC3* | 7991 | -0.68 | 0.03633 |
| cg19357918 | *STIP1* | 10963 | 0.12 | 0.03633 |
| cg19383689 | *WSB1* | 26118 | 0.15 | 0.03633 |
| cg19398446 | *NUDT2* | 318 | 0.15 | 0.03633 |
| cg19400725 | *RILP* | 83547 | 0.14 | 0.03633 |
| cg19407266 | *FLJ39827* | 139285 | 0.13 | 0.03633 |
| cg19432434 | *SIKE* | 80143 | 0.12 | 0.03633 |
| cg19439331 | *CXXC6* | 80312 | 0.14 | 0.03633 |
| cg19464016 | *PRDM1* | 639 | 0.12 | 0.03633 |
| cg19478743 | *CXCL16* | 58191 | 0.12 | 0.03633 |
| cg19486673 | *LILRA2* | 11027 | 0.77 | 0.03633 |
| cg19491035 | *MANSC1* | 54682 | 0.10 | 0.03633 |
| cg19517291 | *NMB* | 4828 | -0.70 | 0.03633 |
| cg19522294 | *PVRL3* | 25945 | 0.16 | 0.03633 |
| cg19552482 | *ZNF691* | 51058 | 0.17 | 0.03633 |
| cg19572242 | *IRAK1* | 3654 | 0.17 | 0.03633 |
| cg19574623 | *SSPN* | 8082 | 0.13 | 0.03633 |
| cg19596204 | *ISYNA1* | 51477 | 0.11 | 0.03633 |
| cg19601328 | *MAP3K14* | 9020 | 0.38 | 0.03633 |
| cg19604470 | *CD81* | 975 | 0.12 | 0.03633 |
| cg19626078 | *KNDC1* | 85442 | 0.12 | 0.03633 |
| cg19636861 | *ZNF556* | 80032 | -0.76 | 0.03633 |
| cg19655070 | *HEXIM2* | 124790 | 0.15 | 0.03633 |
| cg19685066 | *ACTL8* | 81569 | -0.70 | 0.03633 |
| cg19690101 | *DHX57* | 90957 | -0.56 | 0.03633 |
| cg19690214 | *PIP3-E* | 26034 | 0.63 | 0.03633 |
| cg19704039 | *GNPDA1* | 10007 | 0.08 | 0.03633 |
| cg19714241 | *STCH* | 6782 | 0.15 | 0.03633 |
| cg19777783 | *SLC15A4* | 121260 | -0.57 | 0.03633 |
| cg19779211 | *KCNQ1* | 3784 | 0.28 | 0.03633 |
| cg19781053 | *TJP1* | 7082 | 0.13 | 0.03633 |
| cg19781251 | *PEG10* | 23089 | -0.80 | 0.03633 |
| cg19791253 | *ATP2B4* | 493 | 0.12 | 0.03633 |
| cg19791277 | *KHDRBS3* | 10656 | 0.12 | 0.03633 |
| cg19799744 | *ENPP5* | 59084 | 0.10 | 0.03633 |
| cg19824362 | *NR2C1* | 7181 | 0.12 | 0.03633 |
| cg19830147 | *LSS* | 4047 | 0.15 | 0.03633 |
| cg19843036 | *FLJ11155* | 55314 | 0.09 | 0.03633 |
| cg19856594 | *SAGE1* | 55511 | -0.59 | 0.03633 |
| cg19857541 | *MORC1* | 27136 | -0.76 | 0.03633 |
| cg19875047 | *ANKRD42* | 338699 | 0.10 | 0.03633 |
| cg19906550 | *SLC22A18* | 5002 | 0.73 | 0.03633 |
| cg19906926 | *KLHL5* | 51088 | 0.14 | 0.03633 |
| cg19911362 | *USP8* | 9101 | 0.14 | 0.03633 |
| cg19941758 | *MDS025* | 60492 | 0.09 | 0.03633 |
| cg19963522 | *PIP3-E* | 26034 | 0.73 | 0.03633 |
| cg19972859 | *PURA* | 5813 | 0.16 | 0.03633 |
| cg19979773 | *FCN3* | 8547 | -0.08 | 0.03633 |
| cg19981839 | *CD2BP2* | 10421 | 0.19 | 0.03633 |
| cg19998328 | *LOC90580* | 90580 | -0.48 | 0.03633 |
| cg20011794 | *KCTD17* | 79734 | 0.17 | 0.03633 |
| cg20029201 | *BCL9L* | 283149 | -0.56 | 0.03633 |
| cg20047732 | *TTC14* | 151613 | 0.12 | 0.03633 |
| cg20052411 | *RCE1* | 9986 | 0.10 | 0.03633 |
| cg20059312 | *NGEF* | 25791 | -0.11 | 0.03633 |
| cg20082641 | *PAQR5* | 54852 | -0.54 | 0.03633 |
| cg20092036 | *UBE3B* | 89910 | -0.54 | 0.03633 |
| cg20098118 | *UXT* | 8409 | 0.17 | 0.03633 |
| cg20098478 | *WIF1* | 11197 | -0.70 | 0.03633 |
| cg20122491 | *ZFP95* | 23660 | 0.16 | 0.03633 |
| cg20135306 | *SAFB* | 6294 | 0.12 | 0.03633 |
| cg20139214 | *ACACA* | 31 | 0.11 | 0.03633 |
| cg20150565 | *ZNFX1* | 57169 | 0.21 | 0.03633 |
| cg20162159 | *MON1A* | 84315 | 0.13 | 0.03633 |
| cg20175449 | *LOC196264* | 196264 | 0.18 | 0.03633 |
| cg20176648 | *AQP1* | 358 | 0.25 | 0.03633 |
| cg20185083 | *SERPINB9* | 5272 | -0.59 | 0.03633 |
| cg20189937 | *L2HGDH* | 79944 | -0.52 | 0.03633 |
| cg20191453 | *AMT* | 275 | 0.72 | 0.03633 |
| cg20209009 | *TBX21* | 30009 | 0.10 | 0.03633 |
| cg20225889 | *ZNF297* | 9278 | 0.18 | 0.03633 |
| cg20225915 | *LRDD* | 55367 | 0.21 | 0.03633 |
| cg20228512 | *PCGF2* | 7703 | 0.12 | 0.03633 |
| cg20241335 | *NUPL1* | 9818 | 0.11 | 0.03633 |
| cg20259398 | *CDCA7L* | 55536 | 0.09 | 0.03633 |
| cg20281815 | *PSCA* | 8000 | -0.10 | 0.03633 |
| cg20284673 | *DOM3Z* | 1797 | 0.28 | 0.03633 |
| cg20305610 | *PDLIM5* | 10611 | 0.11 | 0.03633 |
| cg20326853 | *CPSF1* | 29894 | 0.10 | 0.03633 |
| cg20340242 | *IL1R2* | 7850 | 0.78 | 0.03633 |
| cg20346122 | *IFT122* | 55764 | 0.14 | 0.03633 |
| cg20366832 | *LLGL2* | 3993 | -0.21 | 0.03633 |
| cg20372689 | *RCN2* | 5955 | 0.20 | 0.03633 |
| cg20380069 | *MSI1* | 4440 | 0.17 | 0.03633 |
| cg20395892 | *IRAK3* | 11213 | 0.12 | 0.03633 |
| cg20411654 | *CLPB* | 81570 | 0.13 | 0.03633 |
| cg20426860 | *FLJ20422* | 54929 | 0.15 | 0.03633 |
| cg20430816 | *GENX-3414* | 8987 | 0.13 | 0.03633 |
| cg20482364 | *NACA* | 4666 | 0.10 | 0.03633 |
| cg20484306 | *MAP2K1* | 5604 | 0.12 | 0.03633 |
| cg20492869 | *TAF1A* | 9015 | 0.13 | 0.03633 |
| cg20492912 | *UBE4A* | 9354 | -0.69 | 0.03633 |
| cg20495040 | *RAG1AP1* | 55974 | 0.21 | 0.03633 |
| cg20514061 | *MID2* | 11043 | 0.14 | 0.03633 |
| cg20543571 | *C15orf43* | 145645 | -0.63 | 0.03633 |
| cg20544605 | *SORBS2* | 8470 | -0.67 | 0.03633 |
| cg20571908 | *MPHOSPH1* | 9585 | 0.14 | 0.03633 |
| cg20573242 | *CCNA2* | 890 | 0.16 | 0.03633 |
| cg20579480 | *APOBEC4* | 403314 | 0.56 | 0.03633 |
| cg20595300 | *UHRF2* | 115426 | 0.10 | 0.03633 |
| cg20603888 | *MYEF2* | 50804 | -0.46 | 0.03633 |
| cg20623943 | *CMPK* | 51727 | 0.12 | 0.03633 |
| cg20624391 | *BVES* | 11149 | 0.19 | 0.03633 |
| cg20633959 | *NRF1* | 4899 | 0.09 | 0.03633 |
| cg20638426 | *ZNF14* | 7561 | 0.12 | 0.03633 |
| cg20642765 | *MLF2* | 8079 | 0.15 | 0.03633 |
| cg20664247 | *PEX10* | 5192 | -0.71 | 0.03633 |
| cg20673611 | *TM7SF3* | 51768 | 0.12 | 0.03633 |
| cg20681975 | *TNRC5* | 10695 | 0.13 | 0.03633 |
| cg20688340 | *MMP21* | 118856 | -0.73 | 0.03633 |
| cg20697204 | *FLJ40172* | 285051 | -0.61 | 0.03633 |
| cg20699736 | *VAX2* | 25806 | 0.13 | 0.03633 |
| cg20700904 | *HNRPH3* | 3189 | 0.11 | 0.03633 |
| cg20702327 | *CSNK2A1* | 1457 | 0.09 | 0.03633 |
| cg20707333 | *C20orf177* | 63939 | -0.59 | 0.03633 |
| cg20713333 | *RG9MTD2* | 93587 | 0.14 | 0.03633 |
| cg20748065 | *POR* | 5447 | 0.79 | 0.03633 |
| cg20752438 | *C9orf127* | 51754 | 0.13 | 0.03633 |
| cg20757196 | *TRAF7* | 84231 | 0.11 | 0.03633 |
| cg20767936 | *ASNSD1* | 54529 | 0.09 | 0.03633 |
| cg20775840 | *G3BP* | 10146 | 0.08 | 0.03633 |
| cg20804555 | *GSC* | 145258 | 0.10 | 0.03633 |
| cg20822579 | *RIPK3* | 11035 | 0.10 | 0.03633 |
| cg20852286 | *DHFRL1* | 200895 | 0.12 | 0.03633 |
| cg20854748 | *PIP5K1A* | 8394 | 0.11 | 0.03633 |
| cg20855303 | *REPS2* | 9185 | 0.28 | 0.03633 |
| cg20857455 | *CPNE8* | 144402 | 0.17 | 0.03633 |
| cg20902737 | *EFTUD2* | 9343 | 0.14 | 0.03633 |
| cg20922422 | *PCYT1B* | 9468 | 0.15 | 0.03633 |
| cg20940661 | *KCNS3* | 3790 | 0.10 | 0.03633 |
| cg20970875 | *ANKRD47* | 256949 | 0.12 | 0.03633 |
| cg20979799 | *RFXDC1* | 222546 | 0.14 | 0.03633 |
| cg21008709 | *SS18L2* | 51188 | 0.11 | 0.03633 |
| cg21030483 | *FAM11A* | 84548 | 0.12 | 0.03633 |
| cg21035142 | *CRYGN* | 155051 | 0.21 | 0.03633 |
| cg21039822 | *PCYT2* | 5833 | 0.14 | 0.03633 |
| cg21048501 | *MRPS24* | 64951 | 0.14 | 0.03633 |
| cg21053015 | *SYNGR1* | 9145 | 0.15 | 0.03633 |
| cg21069922 | *ERAL1* | 26284 | 0.13 | 0.03633 |
| cg21076470 | *MRPS14* | 63931 | 0.17 | 0.03633 |
| cg21085768 | *PRSS21* | 10942 | -0.69 | 0.03633 |
| cg21092324 | *MMRN1* | 22915 | 0.46 | 0.03633 |
| cg21094669 | *GLRA3* | 8001 | 0.13 | 0.03633 |
| cg21115990 | *LRP5* | 4041 | -0.64 | 0.03633 |
| cg21116410 | *PEX12* | 5193 | 0.12 | 0.03633 |
| cg21117584 | *SNIP1* | 79753 | 0.15 | 0.03633 |
| cg21129531 | *LRRC4* | 64101 | -0.70 | 0.03633 |
| cg21209091 | *DKK4* | 27121 | 0.38 | 0.03633 |
| cg21211748 | *E2F2* | 1870 | 0.13 | 0.03633 |
| cg21223368 | *IDH3A* | 3419 | 0.16 | 0.03633 |
| cg21238457 | *WNT11* | 7481 | 0.14 | 0.03633 |
| cg21250577 | *ETFA* | 2108 | 0.15 | 0.03633 |
| cg21255626 | *UQCRFS1* | 7386 | 0.11 | 0.03633 |
| cg21257635 | *SYF2* | 25949 | 0.10 | 0.03633 |
| cg21273125 | *ABHD2* | 11057 | 0.12 | 0.03633 |
| cg21277505 | *LOC284361* | 284361 | -0.16 | 0.03633 |
| cg21283680 | *SH3BP5* | 9467 | 0.43 | 0.03633 |
| cg21288099 | *RRAS2* | 22800 | 0.13 | 0.03633 |
| cg21295467 | *LOH11CR2A* | 4013 | 0.12 | 0.03633 |
| cg21309049 | *STK17A* | 9263 | 0.12 | 0.03633 |
| cg21311175 | *HIRA* | 7290 | 0.13 | 0.03633 |
| cg21322149 | *SENP7* | 57337 | 0.13 | 0.03633 |
| cg21336547 | *AKAP8* | 10270 | 0.13 | 0.03633 |
| cg21350115 | *CALCRL* | 10203 | 0.40 | 0.03633 |
| cg21363943 | *SF3B4* | 10262 | 0.11 | 0.03633 |
| cg21372397 | *HIC2* | 23119 | 0.12 | 0.03633 |
| cg21379309 | *C14orf147* | 171546 | 0.15 | 0.03633 |
| cg21380842 | *KCNJ6* | 3763 | 0.12 | 0.03633 |
| cg21388029 | *CREG2* | 200407 | 0.13 | 0.03633 |
| cg21389884 | *PVRL1* | 5818 | 0.12 | 0.03633 |
| cg21391660 | *LLGL2* | 3993 | 0.10 | 0.03633 |
| cg21404906 | *XRCC5* | 7520 | 0.14 | 0.03633 |
| cg21405195 | *PEG10* | 23089 | -0.41 | 0.03633 |
| cg21414251 | *OR12D2* | 26529 | -0.51 | 0.03633 |
| cg21415698 | *EXPH5* | 23086 | 0.15 | 0.03633 |
| cg21416022 | *TTC23* | 64927 | 0.17 | 0.03633 |
| cg21416237 | *FKBP10* | 60681 | -0.29 | 0.03633 |
| cg21432842 | *CSF3* | 1440 | 0.73 | 0.03633 |
| cg21459921 | *ADRM1* | 11047 | 0.11 | 0.03633 |
| cg21472099 | *SYAP1* | 94056 | 0.14 | 0.03633 |
| cg21476940 | *ITLN1* | 55600 | 0.45 | 0.03633 |
| cg21508214 | *IFT80* | 57560 | 0.11 | 0.03633 |
| cg21509023 | *HBA2* | 3040 | 0.11 | 0.03633 |
| cg21515243 | *RIOK3* | 8780 | 0.18 | 0.03633 |
| cg21523528 | *CBFA2T2* | 9139 | 0.13 | 0.03633 |
| cg21530453 | *GNAO1* | 2775 | 0.12 | 0.03633 |
| cg21530890 | *SOX8* | 30812 | 0.14 | 0.03633 |
| cg21548414 | *CLIC5* | 53405 | 0.15 | 0.03633 |
| cg21587861 | *BICD1* | 636 | 0.11 | 0.03633 |
| cg21588305 | *NNAT* | 4826 | -0.58 | 0.03633 |
| cg21608489 | *POU2F2* | 5452 | 0.12 | 0.03633 |
| cg21614231 | *BAG1* | 573 | -0.16 | 0.03633 |
| cg21636748 | *PLEKHA8* | 84725 | 0.14 | 0.03633 |
| cg21638219 | *PHYH2* | 26061 | 0.12 | 0.03633 |
| cg21655790 | *LEPR* | 3953 | 0.17 | 0.03633 |
| cg21663580 | *PTK7* | 5754 | 0.11 | 0.03633 |
| cg21666217 | *TRIM33* | 51592 | 0.15 | 0.03633 |
| cg21673646 | *SHF* | 90525 | 0.11 | 0.03633 |
| cg21693321 | *MED12* | 9968 | 0.16 | 0.03633 |
| cg21694349 | *FBXO24* | 26261 | 0.09 | 0.03633 |
| cg21712685 | *UNQ3045* | 389383 | 0.12 | 0.03633 |
| cg21722680 | *TBK1* | 29110 | 0.12 | 0.03633 |
| cg21730067 | *ZNF326* | 284695 | 0.10 | 0.03633 |
| cg21740204 | *SMC5L1* | 23137 | 0.19 | 0.03633 |
| cg21784498 | *TMEM97* | 27346 | 0.11 | 0.03633 |
| cg21796234 | *C3orf23* | 285343 | 0.11 | 0.03633 |
| cg21801378 | *BRUNOL6* | 60677 | 0.12 | 0.03633 |
| cg21825364 | *VCY* | 9084 | -0.80 | 0.03633 |
| cg21832243 | *TTC3* | 7267 | -0.44 | 0.03633 |
| cg21837443 | *FLJ20647* | 55013 | 0.10 | 0.03633 |
| cg21846903 | *VTN* | 7448 | 0.63 | 0.03633 |
| cg21861211 | *ZFYVE26* | 23503 | 0.16 | 0.03633 |
| cg21885995 | *SRP68* | 6730 | -0.55 | 0.03633 |
| cg21902119 | *VAT1* | 10493 | 0.15 | 0.03633 |
| cg21921474 | *C1orf172* | 126695 | -0.46 | 0.03633 |
| cg21925493 | *C9orf89* | 84270 | 0.17 | 0.03633 |
| cg21932814 | *CSTA* | 1475 | 0.56 | 0.03633 |
| cg21948169 | *CXXC6* | 80312 | 0.11 | 0.03633 |
| cg21949781 | *PSTPIP2* | 9050 | 0.15 | 0.03633 |
| cg21973517 | *VGLL4* | 9686 | 0.14 | 0.03633 |
| cg21974239 | *MAPK12* | 6300 | 0.11 | 0.03633 |
| cg21985356 | *ZBED3* | 84327 | 0.11 | 0.03633 |
| cg21989500 | *NDUFAB1* | 4706 | 0.09 | 0.03633 |
| cg21991830 | *RB1CC1* | 9821 | 0.12 | 0.03633 |
| cg22016818 | *TULP3* | 7289 | 0.14 | 0.03633 |
| cg22029275 | *FLJ25477* | 219287 | 0.18 | 0.03633 |
| cg22030890 | *RAB32* | 10981 | 0.08 | 0.03633 |
| cg22031736 | *LOC129138* | 129138 | 0.12 | 0.03633 |
| cg22034555 | *DHX36* | 170506 | 0.14 | 0.03633 |
| cg22054191 | *POU2F2* | 5452 | 0.19 | 0.03633 |
| cg22055427 | *P2RY1* | 5028 | 0.17 | 0.03633 |
| cg22065500 | *TNPO3* | 23534 | 0.09 | 0.03633 |
| cg22088368 | *MGC35206* | 339669 | 0.54 | 0.03633 |
| cg22120948 | *GIT1* | 28964 | 0.12 | 0.03633 |
| cg22131691 | *PDE1C* | 5137 | 0.11 | 0.03633 |
| cg22138278 | *MAF1* | 84232 | 0.12 | 0.03633 |
| cg22172230 | *SWAP70* | 23075 | 0.11 | 0.03633 |
| cg22183706 | *CALCA* | 796 | 0.11 | 0.03633 |
| cg22187094 | *POLG* | 5428 | 0.14 | 0.03633 |
| cg22189019 | *FGF22* | 27006 | -0.66 | 0.03633 |
| cg22190291 | *NBPF3* | 84224 | 0.14 | 0.03633 |
| cg22194305 | *MXD4* | 10608 | 0.10 | 0.03633 |
| cg22198623 | *MT1G* | 4495 | 0.17 | 0.03633 |
| cg22199080 | *CENTG1* | 116986 | 0.48 | 0.03633 |
| cg22229034 | *DUSP1* | 1843 | 0.14 | 0.03633 |
| cg22234344 | *HIBCH* | 26275 | 0.19 | 0.03633 |
| cg22258437 | *ID3* | 3399 | 1.00 | 0.03633 |
| cg22266967 | *S100P* | 6286 | 0.67 | 0.03633 |
| cg22267176 | *TCTE3* | 6991 | 0.13 | 0.03633 |
| cg22325572 | *CD53* | 963 | 0.41 | 0.03633 |
| cg22325646 | *RND2* | 8153 | 0.20 | 0.03633 |
| cg22335801 | *TNFRSF4* | 7293 | 0.89 | 0.03633 |
| cg22359217 | *FBXO4* | 26272 | 0.12 | 0.03633 |
| cg22367970 | *GON4* | 54856 | 0.16 | 0.03633 |
| cg22370370 | *ZNF652* | 22834 | 0.15 | 0.03633 |
| cg22374901 | *TESC* | 54997 | 0.14 | 0.03633 |
| cg22377963 | *BCL7C* | 9274 | -0.35 | 0.03633 |
| cg22397854 | *C1orf102* | 127700 | 0.10 | 0.03633 |
| cg22417789 | *SPRY2* | 10253 | 0.12 | 0.03633 |
| cg22432269 | *CYFIP1* | 23191 | 0.13 | 0.03633 |
| cg22436411 | *FCGR2B* | 2213 | 0.58 | 0.03633 |
| cg22461018 | *MORF4* | 10934 | 0.24 | 0.03633 |
| cg22468123 | *KLP1* | 57106 | 0.15 | 0.03633 |
| cg22477592 | *ATM* | 472 | 0.13 | 0.03633 |
| cg22490255 | *TSPAN31* | 6302 | 0.17 | 0.03633 |
| cg22502502 | *TRIM38* | 10475 | 0.52 | 0.03633 |
| cg22510943 | *LMNB2* | 84823 | 0.20 | 0.03633 |
| cg22512531 | *CRTAM* | 56253 | -0.34 | 0.03633 |
| cg22520471 | *DDX42* | 11325 | 0.15 | 0.03633 |
| cg22539738 | *KIAA1704* | 55425 | -0.71 | 0.03633 |
| cg22541143 | *HEL308* | 113510 | 0.21 | 0.03633 |
| cg22552669 | *SSSCA1* | 10534 | 0.12 | 0.03633 |
| cg22571217 | *DAPK1* | 1612 | 0.16 | 0.03633 |
| cg22572159 | *NGFB* | 4803 | 0.13 | 0.03633 |
| cg22578204 | *TIMP3* | 7078 | 0.08 | 0.03633 |
| cg22585269 | *CHEK2* | 11200 | 0.18 | 0.03633 |
| cg22593486 | *OR10H1* | 26539 | -0.22 | 0.03633 |
| cg22623062 | *TP53I11* | 9537 | 0.14 | 0.03633 |
| cg22632663 | *RNF41* | 10193 | 0.14 | 0.03633 |
| cg22636253 | *SSRP1* | 6749 | 0.14 | 0.03633 |
| cg22640452 | *NR1D1* | 9572 | 0.14 | 0.03633 |
| cg22658979 | *MMP13* | 4322 | -0.62 | 0.03633 |
| cg22659426 | *CLN3* | 1201 | 0.08 | 0.03633 |
| cg22717127 | *CBWD2* | 150472 | 0.13 | 0.03633 |
| cg22751696 | *C3orf14* | 57415 | 0.16 | 0.03633 |
| cg22769406 | *COX6B1* | 1340 | 0.20 | 0.03633 |
| cg22771603 | *HSPA4* | 3308 | 0.13 | 0.03633 |
| cg22774127 | *COX10* | 1352 | 0.13 | 0.03633 |
| cg22774472 | *COL5A2* | 1290 | 0.25 | 0.03633 |
| cg22775000 | *TMEFF1* | 8577 | 0.12 | 0.03633 |
| cg22777952 | *FOXB1* | 27023 | 0.12 | 0.03633 |
| cg22788223 | *NFYA* | 4800 | 0.13 | 0.03633 |
| cg22799132 | *TRPC5* | 7224 | 0.16 | 0.03633 |
| cg22800191 | *HPS3* | 84343 | 0.12 | 0.03633 |
| cg22805632 | *KRT10* | 3858 | -0.13 | 0.03633 |
| cg22808175 | *PHOSPHO1* | 162466 | 0.13 | 0.03633 |
| cg22825487 | *VNN3* | 55350 | 0.68 | 0.03633 |
| cg22825979 | *RFP2* | 10206 | 0.15 | 0.03633 |
| cg22828804 | *FLJ13149* | 60493 | 0.14 | 0.03633 |
| cg22831263 | *KIAA1840* | 80208 | 0.13 | 0.03633 |
| cg22836470 | *TMEM119* | 338773 | 0.20 | 0.03633 |
| cg22841169 | *YPEL5* | 51646 | 0.18 | 0.03633 |
| cg22863122 | *DUSP7* | 1849 | 0.11 | 0.03633 |
| cg22873668 | *CXorf56* | 63932 | -0.43 | 0.03633 |
| cg22882178 | *PITX3* | 5309 | 0.15 | 0.03633 |
| cg22915732 | *MGC34646* | 157807 | 0.15 | 0.03633 |
| cg22916109 | *IREB2* | 3658 | 0.11 | 0.03633 |
| cg22950391 | *ZNF192* | 7745 | 0.17 | 0.03633 |
| cg22956315 | *TTC1* | 7265 | 0.13 | 0.03633 |
| cg22960284 | *PYGL* | 5836 | 0.12 | 0.03633 |
| cg22960501 | *IKBKB* | 3551 | 0.11 | 0.03633 |
| cg22997490 | *ZNF286* | 57335 | 0.10 | 0.03633 |
| cg22998840 | *TCEAL3* | 85012 | 0.19 | 0.03633 |
| cg23029491 | *UBAP2* | 55833 | 0.19 | 0.03633 |
| cg23029494 | *BCL2L12* | 83596 | 0.14 | 0.03633 |
| cg23043438 | *TEX10* | 54881 | 0.09 | 0.03633 |
| cg23078294 | *RSBN1* | 54665 | 0.15 | 0.03633 |
| cg23091824 | *SLC12A7* | 10723 | 0.16 | 0.03633 |
| cg23093496 | *C16orf54* | 283897 | 0.75 | 0.03633 |
| cg23127998 | *FLRT3* | 23767 | 0.65 | 0.03633 |
| cg23142900 | *GTF2H4* | 2968 | 0.11 | 0.03633 |
| cg23150609 | *YARS* | 8565 | 0.12 | 0.03633 |
| cg23158811 | *PBX4* | 80714 | 0.10 | 0.03633 |
| cg23159296 | *SREBF1* | 6720 | 0.12 | 0.03633 |
| cg23178308 | *C21orf124* | 85006 | 0.13 | 0.03633 |
| cg23186643 | *NPB* | 256933 | 0.11 | 0.03633 |
| cg23202409 | *C11orf48* | 79081 | 0.11 | 0.03633 |
| cg23207990 | *SFRP2* | 6423 | 0.13 | 0.03633 |
| cg23214764 | *PRSS23* | 11098 | 0.08 | 0.03633 |
| cg23219570 | *FGF23* | 8074 | 0.84 | 0.03633 |
| cg23231873 | *USP32* | 84669 | 0.14 | 0.03633 |
| cg23248865 | *SEMA4C* | 54910 | 0.13 | 0.03633 |
| cg23258063 | *SCYL3* | 57147 | 0.12 | 0.03633 |
| cg23276695 | *CNR1* | 1268 | 0.22 | 0.03633 |
| cg23280339 | *PQBP1* | 10084 | 0.15 | 0.03633 |
| cg23290344 | *NEF3* | 4741 | 0.12 | 0.03633 |
| cg23297477 | *CMTM3* | 123920 | 0.12 | 0.03633 |
| cg23300372 | *CART* | 9607 | 0.12 | 0.03633 |
| cg23310538 | *PLEKHA9* | 51054 | 0.13 | 0.03633 |
| cg23311628 | *RAB8B* | 51762 | 0.12 | 0.03633 |
| cg23322316 | *SPATA4* | 132851 | 0.13 | 0.03633 |
| cg23328404 | *ChGn* | 55790 | 0.63 | 0.03633 |
| cg23333306 | *CDC42EP4* | 23580 | 0.11 | 0.03633 |
| cg23333420 | *OBFC1* | 79991 | 0.11 | 0.03633 |
| cg23352695 | *EVI2A* | 2123 | 0.54 | 0.03633 |
| cg23356993 | *TIMP2* | 7077 | 0.14 | 0.03633 |
| cg23392730 | *CHCHD1* | 118487 | 0.17 | 0.03633 |
| cg23402444 | *PSD* | 5662 | 0.13 | 0.03633 |
| cg23410627 | *CH25H* | 9023 | 0.11 | 0.03633 |
| cg23411876 | *NDUFB2* | 4708 | 0.21 | 0.03633 |
| cg23412777 | *PYGO1* | 26108 | -0.63 | 0.03633 |
| cg23431988 | *PIWIL2* | 55124 | -0.65 | 0.03633 |
| cg23434919 | *STK10* | 6793 | 0.13 | 0.03633 |
| cg23445511 | *NIP7* | 51388 | 0.09 | 0.03633 |
| cg23472215 | *GSTM3* | 2947 | 0.10 | 0.03633 |
| cg23478284 | *PTPN13* | 5783 | 0.12 | 0.03633 |
| cg23489497 | *ATPBD1B* | 54707 | 0.11 | 0.03633 |
| cg23506842 | *PTPN7* | 5778 | 0.52 | 0.03633 |
| cg23509869 | *LST1* | 7940 | 0.34 | 0.03633 |
| cg23517677 | *POLR2G* | 5436 | -0.53 | 0.03633 |
| cg23525180 | *ID2* | 3398 | 0.15 | 0.03633 |
| cg23548920 | *ARRDC2* | 27106 | 0.17 | 0.03633 |
| cg23566335 | *ADAM18* | 8749 | -0.86 | 0.03633 |
| cg23602684 | *NRL* | 4901 | 0.12 | 0.03633 |
| cg23610841 | *PURG* | 29942 | 0.25 | 0.03633 |
| cg23612220 | *TNFAIP8L2* | 79626 | 0.79 | 0.03633 |
| cg23619829 | *MTFR1* | 9650 | 0.15 | 0.03633 |
| cg23621689 | *C6orf25* | 80739 | -0.94 | 0.03633 |
| cg23625458 | *AFAR3* | 246181 | -1.02 | 0.03633 |
| cg23640903 | *GFOD1* | 54438 | 0.11 | 0.03633 |
| cg23653187 | *ADPN* | 80339 | 0.63 | 0.03633 |
| cg23654219 | *TCF19* | 6941 | -0.65 | 0.03633 |
| cg23667407 | *LOC51255* | 51255 | 0.14 | 0.03633 |
| cg23686014 | *TFPI2* | 7980 | 0.11 | 0.03633 |
| cg23687319 | *GGPS1* | 9453 | 0.15 | 0.03633 |
| cg23695504 | *FLJ45717* | 388759 | 0.11 | 0.03633 |
| cg23696834 | *GNMT* | 27232 | 0.10 | 0.03633 |
| cg23739036 | *PEX10* | 5192 | 0.11 | 0.03633 |
| cg23741330 | *C6orf188* | 254228 | -0.39 | 0.03633 |
| cg23748737 | *SCARF2* | 91179 | 0.11 | 0.03633 |
| cg23751922 | *CREG1* | 8804 | 0.10 | 0.03633 |
| cg23756219 | *DRP2* | 1821 | 0.83 | 0.03633 |
| cg23771603 | *MYO3A* | 53904 | 0.13 | 0.03633 |
| cg23807559 | *COG1* | 9382 | 0.12 | 0.03633 |
| cg23816572 | *CEP68* | 23177 | 0.11 | 0.03633 |
| cg23835812 | *CHMP2B* | 25978 | 0.14 | 0.03633 |
| cg23844090 | *TMEM42* | 131616 | 0.11 | 0.03633 |
| cg23862908 | *C8orf37* | 157657 | 0.12 | 0.03633 |
| cg23871659 | *TANK* | 10010 | 0.42 | 0.03633 |
| cg23881725 | *DLEC1* | 9940 | 0.15 | 0.03633 |
| cg23889021 | *CTDSP1* | 58190 | 0.18 | 0.03633 |
| cg23897067 | *NDOR1* | 27158 | 0.10 | 0.03633 |
| cg23906760 | *UBL5* | 59286 | 0.15 | 0.03633 |
| cg23910243 | *TGFB1I1* | 7041 | -0.60 | 0.03633 |
| cg23921534 | *POU3F4* | 5456 | 0.10 | 0.03633 |
| cg23922454 | *LHX5* | 64211 | 0.17 | 0.03633 |
| cg23923462 | *PGAM1* | 5223 | 0.15 | 0.03633 |
| cg23933345 | *AP4B1* | 10717 | 0.08 | 0.03633 |
| cg23940655 | *C14orf43* | 91748 | 0.13 | 0.03633 |
| cg23943360 | *LDLR* | 3949 | 0.15 | 0.03633 |
| cg23948874 | *EIF4G2* | 1982 | 0.11 | 0.03633 |
| cg23953831 | *IGSF2* | 9398 | 0.64 | 0.03633 |
| cg23983366 | *RANBP6* | 26953 | 0.20 | 0.03633 |
| cg23983884 | *DBN1* | 1627 | 0.20 | 0.03633 |
| cg24016044 | *CPEB4* | 80315 | 0.13 | 0.03633 |
| cg24027342 | *ATM* | 472 | -0.19 | 0.03633 |
| cg24053587 | *PTPRO* | 5800 | 0.13 | 0.03633 |
| cg24056562 | *RNASEH2A* | 10535 | 0.17 | 0.03633 |
| cg24068372 | *LOC349136* | 349136 | 0.15 | 0.03633 |
| cg24073051 | *CDV3* | 55573 | 0.11 | 0.03633 |
| cg24079455 | *C10orf137* | 26098 | 0.12 | 0.03633 |
| cg24082826 | *ITPR2* | 3709 | 0.10 | 0.03633 |
| cg24139739 | *EFNB1* | 1947 | 0.15 | 0.03633 |
| cg24141135 | *MARS* | 4141 | 0.10 | 0.03633 |
| cg24167037 | *ITGB2* | 3689 | 0.53 | 0.03633 |
| cg24169915 | *FLJ25773* | 283598 | 0.42 | 0.03633 |
| cg24181591 | *EIF5A* | 1984 | 0.17 | 0.03633 |
| cg24196046 | *EIF2C3* | 192669 | 0.24 | 0.03633 |
| cg24219962 | *WBP4* | 11193 | 0.12 | 0.03633 |
| cg24242194 | *MDH2* | 4191 | 0.13 | 0.03633 |
| cg24252723 | *CNNM2* | 54805 | 0.18 | 0.03633 |
| cg24269846 | *PRIM1* | 5557 | 0.12 | 0.03633 |
| cg24283842 | *RPLP1* | 6176 | 0.13 | 0.03633 |
| cg24290574 | *CPA3* | 1359 | 0.52 | 0.03633 |
| cg24315815 | *PLSCR4* | 57088 | -0.80 | 0.03633 |
| cg24320643 | *ADAM17* | 6868 | 0.11 | 0.03633 |
| cg24326142 | *CSF1* | 1435 | 0.14 | 0.03633 |
| cg24329374 | *NIPSNAP3A* | 25934 | 0.11 | 0.03633 |
| cg24330485 | *EEF1B2* | 1933 | 0.11 | 0.03633 |
| cg24337809 | *BTG2* | 7832 | 0.13 | 0.03633 |
| cg24341220 | *RetSat* | 54884 | 0.15 | 0.03633 |
| cg24371157 | *PDCD4* | 27250 | 0.14 | 0.03633 |
| cg24394865 | *MRPL11* | 65003 | 0.17 | 0.03633 |
| cg24400943 | *MGC39681* | 283197 | -0.73 | 0.03633 |
| cg24408199 | *EIF2B5* | 8893 | 0.10 | 0.03633 |
| cg24432073 | *CDKL2* | 8999 | 0.10 | 0.03633 |
| cg24444408 | *KLHDC4* | 54758 | 0.23 | 0.03633 |
| cg24448259 | *CPVL* | 54504 | 0.15 | 0.03633 |
| cg24457521 | *ATP5E* | 514 | 0.15 | 0.03633 |
| cg24467291 | *RSN* | 6249 | 0.12 | 0.03633 |
| cg24473385 | *ZNHIT3* | 9326 | 0.14 | 0.03633 |
| cg24497819 | *SELPLG* | 6404 | 0.79 | 0.03633 |
| cg24517609 | *SSR3* | 6747 | 0.09 | 0.03633 |
| cg24570987 | *RPS6KB2* | 6199 | 0.12 | 0.03633 |
| cg24576270 | *CUGBP1* | 10658 | 0.11 | 0.03633 |
| cg24587268 | *OSBPL2* | 9885 | 0.10 | 0.03633 |
| cg24592364 | *ANKH* | 56172 | 0.10 | 0.03633 |
| cg24628744 | *H2AFY* | 9555 | 0.14 | 0.03633 |
| cg24631950 | *UBE2D1* | 7321 | 0.16 | 0.03633 |
| cg24635866 | *CCT2* | 10576 | 0.10 | 0.03633 |
| cg24641737 | *DENND2D* | 79961 | 0.52 | 0.03633 |
| cg24662718 | *VAV3* | 10451 | 0.10 | 0.03633 |
| cg24664861 | *SPAG16* | 79582 | 0.13 | 0.03633 |
| cg24682740 | *UBP1* | 7342 | 0.12 | 0.03633 |
| cg24697031 | *EVPL* | 2125 | 0.73 | 0.03633 |
| cg24701874 | *DNAJB12* | 54788 | 0.17 | 0.03633 |
| cg24710073 | *TBP* | 6908 | -0.41 | 0.03633 |
| cg24713080 | *NDUFS8* | 4728 | 0.13 | 0.03633 |
| cg24714666 | *TRAPPC2* | 6399 | 0.12 | 0.03633 |
| cg24730207 | *PTGES2* | 80142 | -0.50 | 0.03633 |
| cg24731756 | *ILK* | 3611 | 0.12 | 0.03633 |
| cg24777762 | *TNFRSF19L* | 84957 | 0.11 | 0.03633 |
| cg24793903 | *SCRN2* | 90507 | 0.10 | 0.03633 |
| cg24794758 | *LENG1* | 79165 | 0.08 | 0.03633 |
| cg24798047 | *GOLT1A* | 127845 | -0.53 | 0.03633 |
| cg24807354 | *EDG8* | 53637 | 0.16 | 0.03633 |
| cg24809640 | *MED4* | 29079 | 0.18 | 0.03633 |
| cg24820508 | *TXNDC6* | 347736 | 0.13 | 0.03633 |
| cg24826867 | *IRF8* | 3394 | 0.10 | 0.03633 |
| cg24834740 | *PPP1R16B* | 26051 | 0.10 | 0.03633 |
| cg24847163 | *GABBR1* | 2550 | 0.13 | 0.03633 |
| cg24857721 | *RHD* | 6007 | 0.67 | 0.03633 |
| cg24858591 | *KIAA1143* | 57456 | 0.21 | 0.03633 |
| cg24879595 | *ARF6* | 382 | 0.14 | 0.03633 |
| cg24883276 | *PSAP* | 5660 | 0.11 | 0.03633 |
| cg24886748 | *SLC12A7* | 10723 | 0.20 | 0.03633 |
| cg24890043 | *DKFZP566N034* | 81615 | 0.12 | 0.03633 |
| cg24895052 | *MPP3* | 4356 | -0.68 | 0.03633 |
| cg24896109 | *CYP51A1* | 1595 | 0.12 | 0.03633 |
| cg24898863 | *S100A8* | 6279 | 0.86 | 0.03633 |
| cg24926276 | *LRG1* | 116844 | 0.75 | 0.03633 |
| cg24937126 | *LAMB1* | 3912 | 0.11 | 0.03633 |
| cg24938727 | *GUP1* | 57467 | 0.55 | 0.03633 |
| cg24946544 | *LOC352909* | 352909 | 0.11 | 0.03633 |
| cg24974130 | *RAD50* | 10111 | 0.10 | 0.03633 |
| cg24975842 | *GNAS* | 2778 | -0.48 | 0.03633 |
| cg24977709 | *SSSCA1* | 10534 | 0.09 | 0.03633 |
| cg25017304 | *ARHGEF11* | 9826 | 0.15 | 0.03633 |
| cg25032865 | *FLJ44186* | 346689 | -0.21 | 0.03633 |
| cg25034557 | *PACS1* | 55690 | 0.16 | 0.03633 |
| cg25044651 | *FLJ90650* | 206338 | 0.10 | 0.03633 |
| cg25053301 | *WDR13* | 64743 | 0.22 | 0.03633 |
| cg25058957 | *RAXL1* | 84839 | -0.11 | 0.03633 |
| cg25071429 | *TEX13B* | 56156 | -0.57 | 0.03633 |
| cg25077328 | *BZRP* | 706 | -0.40 | 0.03633 |
| cg25095612 | *NFKB2* | 4791 | 0.13 | 0.03633 |
| cg25096582 | *CFL2* | 1073 | 0.10 | 0.03633 |
| cg25098401 | *LCE2B* | 26239 | -0.20 | 0.03633 |
| cg25112044 | *CDK7* | 1022 | 0.10 | 0.03633 |
| cg25125453 | *C20orf38* | 55304 | 0.38 | 0.03633 |
| cg25133685 | *OR2W1* | 26692 | 0.26 | 0.03633 |
| cg25159539 | *FEM1B* | 10116 | 0.10 | 0.03633 |
| cg25181693 | *ALDH5A1* | 7915 | 0.17 | 0.03633 |
| cg25192419 | *DOCK5* | 80005 | 0.14 | 0.03633 |
| cg25193494 | *FLJ20186* | 54849 | -0.41 | 0.03633 |
| cg25194415 | *KIAA0232* | 9778 | 0.12 | 0.03633 |
| cg25202404 | *PXMP3* | 5828 | 0.10 | 0.03633 |
| cg25226891 | *ZMYM3* | 9203 | 0.14 | 0.03633 |
| cg25243721 | *XRCC4* | 7518 | 0.10 | 0.03633 |
| cg25256723 | *F5* | 2153 | -0.72 | 0.03633 |
| cg25263140 | *AIM1* | 202 | 0.09 | 0.03633 |
| cg25283823 | *HIST2H2BE* | 8349 | 0.13 | 0.03633 |
| cg25291907 | *MIB1* | 57534 | 0.09 | 0.03633 |
| cg25302419 | *CTNND2* | 1501 | 0.12 | 0.03633 |
| cg25309588 | *ATP8A1* | 10396 | 0.09 | 0.03633 |
| cg25316166 | *USP11* | 8237 | 0.09 | 0.03633 |
| cg25341726 | *IL27* | 246778 | 0.38 | 0.03633 |
| cg25345005 | *C3orf9* | 56983 | 0.15 | 0.03633 |
| cg25363445 | *ALX4* | 60529 | 0.16 | 0.03633 |
| cg25397076 | *RBP7* | 116362 | 0.12 | 0.03633 |
| cg25406735 | *FBXO32* | 114907 | 0.13 | 0.03633 |
| cg25410279 | *DXS9879E* | 8270 | 0.14 | 0.03633 |
| cg25421694 | *RAB5A* | 5868 | 0.15 | 0.03633 |
| cg25447894 | *CSDC2* | 27254 | 0.22 | 0.03633 |
| cg25457331 | *GMPR* | 2766 | 0.10 | 0.03633 |
| cg25463428 | *TBC1D10B* | 26000 | 0.11 | 0.03633 |
| cg25468723 | *SEZ6L* | 23544 | 0.14 | 0.03633 |
| cg25473396 | *MPG* | 4350 | 0.22 | 0.03633 |
| cg25478157 | *CEP350* | 9857 | 0.10 | 0.03633 |
| cg25490410 | *TUBGCP5* | 114791 | 0.16 | 0.03633 |
| cg25490411 | *MANBA* | 4126 | 0.12 | 0.03633 |
| cg25495394 | *SAP18* | 10284 | 0.10 | 0.03633 |
| cg25500285 | *EIF4EBP1* | 1978 | 0.14 | 0.03633 |
| cg25543604 | *EID-3* | 126272 | 0.09 | 0.03633 |
| cg25544551 | *NFYB* | 4801 | 0.12 | 0.03633 |
| cg25595446 | *PRMT1* | 3276 | 0.19 | 0.03633 |
| cg25598083 | *ACOT2* | 10965 | -0.49 | 0.03633 |
| cg25599211 | *P4HA3* | 283208 | -0.73 | 0.03633 |
| cg25612145 | *STK31* | 56164 | -0.75 | 0.03633 |
| cg25612480 | *UBE2V2* | 7336 | -0.66 | 0.03633 |
| cg25637824 | *TMEM48* | 55706 | 0.12 | 0.03633 |
| cg25655096 | *GPR92* | 57121 | 0.60 | 0.03633 |
| cg25671651 | *WDR12* | 55759 | 0.10 | 0.03633 |
| cg25673373 | *ARL4D* | 379 | 0.13 | 0.03633 |
| cg25683012 | *BAZ2A* | 11176 | 0.10 | 0.03633 |
| cg25716871 | *MAPKAPK3* | 7867 | 0.11 | 0.03633 |
| cg25737491 | *GABARAP* | 11337 | 0.16 | 0.03633 |
| cg25741794 | *EFHD2* | 79180 | 0.21 | 0.03633 |
| cg25752527 | *UBXD2* | 23190 | 0.11 | 0.03633 |
| cg25807810 | *DKFZp434N062* | 151313 | 0.11 | 0.03633 |
| cg25836326 | *DRD3* | 1814 | 0.29 | 0.03633 |
| cg25842356 | *MARCH3* | 115123 | -0.65 | 0.03633 |
| cg25842633 | *SCUBE1* | 80274 | 0.27 | 0.03633 |
| cg25856179 | *STAT1* | 6772 | 0.14 | 0.03633 |
| cg25858682 | *CCDC51* | 79714 | 0.10 | 0.03633 |
| cg25860314 | *SEC24D* | 9871 | 0.15 | 0.03633 |
| cg25866075 | *NALP12* | 91662 | 0.53 | 0.03633 |
| cg25898500 | *HAGH* | 3029 | 0.08 | 0.03633 |
| cg25907916 | *C10orf56* | 219654 | 0.14 | 0.03633 |
| cg25927124 | *PRKAR2A* | 5576 | 0.12 | 0.03633 |
| cg25969212 | *EXOSC9* | 5393 | 0.16 | 0.03633 |
| cg25985488 | *GALNTL4* | 374378 | 0.15 | 0.03633 |
| cg25999267 | *FLJ20551* | 54977 | 0.19 | 0.03633 |
| cg26036443 | *EHD2* | 30846 | 0.14 | 0.03633 |
| cg26039806 | *RNF121* | 55298 | 0.13 | 0.03633 |
| cg26045205 | *C1orf179* | 338094 | -0.16 | 0.03633 |
| cg26062856 | *ATP10A* | 57194 | -0.72 | 0.03633 |
| cg26074851 | *SKP1A* | 6500 | 0.13 | 0.03633 |
| cg26090652 | *C1QTNF5* | 114902 | 0.14 | 0.03633 |
| cg26091688 | *ZNF200* | 7752 | 0.12 | 0.03633 |
| cg26105232 | *IL2RA* | 3559 | 0.46 | 0.03633 |
| cg26112639 | *CIAS1* | 114548 | 0.86 | 0.03633 |
| cg26132665 | *C6orf165* | 154313 | 0.16 | 0.03633 |
| cg26132853 | *NOL5A* | 10528 | 0.16 | 0.03633 |
| cg26137794 | *ATP6V0A2* | 23545 | 0.12 | 0.03633 |
| cg26147480 | *KIAA0157* | 23172 | -0.60 | 0.03633 |
| cg26151675 | *CNR2* | 1269 | 0.56 | 0.03633 |
| cg26153642 | *HTR3E* | 285242 | -0.54 | 0.03633 |
| cg26162582 | *KCNA6* | 3742 | 0.15 | 0.03633 |
| cg26164310 | *LPPR4* | 9890 | 0.12 | 0.03633 |
| cg26187237 | *IGFBP2* | 3485 | 0.11 | 0.03633 |
| cg26190136 | *IDH3A* | 3419 | 0.19 | 0.03633 |
| cg26212924 | *CYC1* | 1537 | 0.12 | 0.03633 |
| cg26221631 | *BARX2* | 8538 | 0.10 | 0.03633 |
| cg26222229 | *PPIL1* | 51645 | 0.12 | 0.03633 |
| cg26226968 | *SMARCAD1* | 56916 | 0.13 | 0.03633 |
| cg26233253 | *SLC35E4* | 339665 | -0.78 | 0.03633 |
| cg26259575 | *TUBB* | 203068 | 0.08 | 0.03633 |
| cg26266098 | *LZTS2* | 84445 | 0.23 | 0.03633 |
| cg26282150 | *GNAI3* | 2773 | 0.16 | 0.03633 |
| cg26292864 | *ITM2C* | 81618 | 0.14 | 0.03633 |
| cg26309951 | *MORF4L2* | 9643 | 0.14 | 0.03633 |
| cg26333317 | *METTL7A* | 25840 | 0.30 | 0.03633 |
| cg26365854 | *ALX4* | 60529 | 0.15 | 0.03633 |
| cg26385743 | *IMP4* | 92856 | -0.87 | 0.03633 |
| cg26387998 | *HELZ* | 9931 | 0.18 | 0.03633 |
| cg26430450 | *RAB23* | 51715 | 0.13 | 0.03633 |
| cg26450541 | *ZNF187* | 7741 | 0.15 | 0.03633 |
| cg26490839 | *RPS11* | 6205 | 0.10 | 0.03633 |
| cg26523005 | *ZNF662* | 389114 | -0.68 | 0.03633 |
| cg26547788 | *HDHD1A* | 8226 | 0.15 | 0.03633 |
| cg26560960 | *CBWD2* | 150472 | 0.16 | 0.03633 |
| cg26561254 | *ISG20L1* | 64782 | -0.54 | 0.03633 |
| cg26561773 | *VSIG4* | 11326 | -0.36 | 0.03633 |
| cg26568722 | *MCM7* | 4176 | 0.11 | 0.03633 |
| cg26570233 | *JOSD3* | 79101 | -0.40 | 0.03633 |
| cg26577529 | *KRT6E* | 286887 | -0.34 | 0.03633 |
| cg26577945 | *STAM* | 8027 | 0.12 | 0.03633 |
| cg26596161 | *SLC26A7* | 115111 | 0.31 | 0.03633 |
| cg26608332 | *PCQAP* | 51586 | -0.72 | 0.03633 |
| cg26614346 | *SP110* | 3431 | -0.21 | 0.03633 |
| cg26623286 | *MED31* | 51003 | 0.14 | 0.03633 |
| cg26624294 | *CETN2* | 1069 | 0.13 | 0.03633 |
| cg26649005 | *ZNF32* | 7580 | 0.10 | 0.03633 |
| cg26655138 | *RBMX2* | 51634 | 0.14 | 0.03633 |
| cg26660414 | *SLC7A8* | 23428 | 0.18 | 0.03633 |
| cg26661897 | *RTCD1* | 8634 | 0.11 | 0.03633 |
| cg26674132 | *ZNF559* | 84527 | 0.36 | 0.03633 |
| cg26678313 | *LRP1* | 4035 | 0.21 | 0.03633 |
| cg26681081 | *CKAP1* | 1155 | 0.11 | 0.03633 |
| cg26685339 | *HIVEP1* | 3096 | 0.12 | 0.03633 |
| cg26701826 | *MGC26963* | 166929 | 0.60 | 0.03633 |
| cg26705553 | *MMP25* | 64386 | 0.15 | 0.03633 |
| cg26709720 | *B3GALT5* | 10317 | -0.14 | 0.03633 |
| cg26723847 | *hCAP-D3* | 23310 | 0.15 | 0.03633 |
| cg26738912 | *MAGED4* | 81557 | 0.11 | 0.03633 |
| cg26750139 | *ACBD6* | 84320 | 0.14 | 0.03633 |
| cg26753137 | *SIRT5* | 23408 | 0.14 | 0.03633 |
| cg26753512 | *RABEPK* | 10244 | 0.18 | 0.03633 |
| cg26757053 | *LYPLAL1* | 127018 | 0.17 | 0.03633 |
| cg26770281 | *FLJ14768* | 84922 | -0.68 | 0.03633 |
| cg26788215 | *SFRS8* | 6433 | 0.11 | 0.03633 |
| cg26798624 | *ATAD3B* | 83858 | 0.11 | 0.03633 |
| cg26798786 | *KLF9* | 687 | 0.16 | 0.03633 |
| cg26799802 | *PHLDA2* | 7262 | 0.12 | 0.03633 |
| cg26809911 | *COG7* | 91949 | 0.10 | 0.03633 |
| cg26817382 | *TMED4* | 222068 | 0.13 | 0.03633 |
| cg26820922 | *YIPF4* | 84272 | 0.11 | 0.03633 |
| cg26843008 | *RBL1* | 5933 | 0.13 | 0.03633 |
| cg26850503 | *DACT1* | 51339 | 0.14 | 0.03633 |
| cg26861460 | *PARVG* | 64098 | 0.83 | 0.03633 |
| cg26884376 | *ABCF1* | 23 | 0.14 | 0.03633 |
| cg26887625 | *ZNF497* | 162968 | 0.14 | 0.03633 |
| cg26904406 | *TRAF3IP2* | 10758 | 0.10 | 0.03633 |
| cg26909237 | *FBXO15* | 201456 | 0.18 | 0.03633 |
| cg26918645 | *ZNF285* | 26974 | 0.08 | 0.03633 |
| cg26928972 | *CSTA* | 1475 | 0.55 | 0.03633 |
| cg26982998 | *MBD3* | 53615 | 0.10 | 0.03633 |
| cg27005053 | *CFDP1* | 10428 | 0.10 | 0.03633 |
| cg27011193 | *LOC654342* | 654342 | 0.21 | 0.03633 |
| cg27015047 | *DMBT1* | 1755 | -0.17 | 0.03633 |
| cg27042767 | *DDX3X* | 1654 | 0.13 | 0.03633 |
| cg27062284 | *RGS2* | 5997 | 0.13 | 0.03633 |
| cg27063525 | *C6orf68* | 116150 | 0.13 | 0.03633 |
| cg27067618 | *CYP4F3* | 4051 | 0.75 | 0.03633 |
| cg27087525 | *FAM8A1* | 51439 | 0.11 | 0.03633 |
| cg27088844 | *EZH1* | 2145 | 0.14 | 0.03633 |
| cg27094188 | *EIF2C1* | 26523 | -0.56 | 0.03633 |
| cg27104271 | *SLCO2B1* | 11309 | 0.46 | 0.03633 |
| cg27109971 | *THOC1* | 9984 | 0.27 | 0.03633 |
| cg27124774 | *PCDHB10* | 56126 | 0.19 | 0.03633 |
| cg27127056 | *FLJ20280* | 54876 | 0.10 | 0.03633 |
| cg27138018 | *HAPLN2* | 60484 | 0.51 | 0.03633 |
| cg27146152 | *BIRC4BP* | 54739 | 0.61 | 0.03633 |
| cg27159719 | *TMEM71* | 137835 | 0.73 | 0.03633 |
| cg27168073 | *NOMO2* | 283820 | 0.11 | 0.03633 |
| cg27180443 | *SCARB1* | 949 | -0.69 | 0.03633 |
| cg27183007 | *ZPBP* | 11055 | -0.65 | 0.03633 |
| cg27193335 | *EIF1B* | 10289 | 0.11 | 0.03633 |
| cg27194614 | *STK4* | 6789 | 0.14 | 0.03633 |
| cg27272402 | *C20orf135* | 140701 | -0.46 | 0.03633 |
| cg27274728 | *CASP6* | 839 | 0.14 | 0.03633 |
| cg27281008 | *NARS2* | 79731 | 0.15 | 0.03633 |
| cg27293353 | *PRIM2A* | 5558 | 0.13 | 0.03633 |
| cg27303880 | *ROR1* | 4919 | 0.12 | 0.03633 |
| cg27344326 | *DMRTC1* | 63947 | -0.68 | 0.03633 |
| cg27345286 | *FBXO3* | 26273 | 0.16 | 0.03633 |
| cg27349244 | *MLXIP* | 22877 | -0.12 | 0.03633 |
| cg27376271 | *CBLN2* | 147381 | 0.09 | 0.03633 |
| cg27388792 | *DPP3* | 10072 | 0.15 | 0.03633 |
| cg27398499 | *TACSTD2* | 4070 | 0.29 | 0.03633 |
| cg27398547 | *C14orf39* | 317761 | -0.53 | 0.03633 |
| cg27409012 | *UAP1* | 6675 | 0.09 | 0.03633 |
| cg27419217 | *TROAP* | 10024 | 0.13 | 0.03633 |
| cg27427207 | *ATP8A1* | 10396 | 0.15 | 0.03633 |
| cg27431247 | *CHKA* | 1119 | 0.10 | 0.03633 |
| cg27462160 | *MGC26885* | 124044 | 0.16 | 0.03633 |
| cg27462969 | *SERBP1* | 26135 | 0.09 | 0.03633 |
| cg27489994 | *TPT1* | 7178 | 0.10 | 0.03633 |
| cg27507704 | *ETFDH* | 2110 | 0.11 | 0.03633 |
| cg27534796 | *AIG1* | 51390 | 0.17 | 0.03633 |
| cg27563210 | *USP49* | 25862 | 0.20 | 0.03633 |
| cg27582235 | *MYO1F* | 4542 | 0.81 | 0.03633 |
| cg27631817 | *OFCC1* | 266553 | 0.55 | 0.03633 |
| cg27181079 | *FLJ10292* | 55110 | 0.19 | 0.03636 |
| cg05395196 | *HSF2* | 3298 | 0.10 | 0.03637 |
| cg24832140 | *ZNF114* | 163071 | 0.18 | 0.03641 |
| cg00899641 | *SF3B1* | 23451 | 0.11 | 0.03644 |
| cg01402045 | *FLJ23356* | 84197 | 0.13 | 0.03644 |
| cg01479232 | *C20orf54* | 113278 | 0.16 | 0.03644 |
| cg02883161 | *MSI1* | 4440 | 0.18 | 0.03644 |
| cg03070588 | *USP30* | 84749 | 0.09 | 0.03644 |
| cg05263682 | *RNGTT* | 8732 | 0.12 | 0.03644 |
| cg07433344 | *HSD17B8* | 7923 | 0.11 | 0.03644 |
| cg09458237 | *HSPA12B* | 116835 | -0.31 | 0.03644 |
| cg11254522 | *FGR* | 2268 | 0.68 | 0.03644 |
| cg14892768 | *AXL* | 558 | 0.59 | 0.03644 |
| cg15725933 | *SLC19A1* | 6573 | 0.15 | 0.03644 |
| cg16770799 | *ITGA5* | 3678 | 0.13 | 0.03644 |
| cg18123677 | *SERPINI1* | 5274 | -0.37 | 0.03644 |
| cg18593668 | *GNAZ* | 2781 | 0.13 | 0.03644 |
| cg18783796 | *C6orf199* | 221264 | 0.12 | 0.03644 |
| cg19255783 | *PLAC8* | 51316 | 0.26 | 0.03644 |
| cg20051589 | *DLG3* | 1741 | 0.17 | 0.03644 |
| cg25182621 | *CHRD* | 8646 | -0.79 | 0.03644 |
| cg25835225 | *ZNF350* | 59348 | 0.13 | 0.03644 |
| cg26406994 | *RAP1GDS1* | 5910 | 0.11 | 0.03644 |
| cg05070690 | *MOV10L1* | 54456 | -0.94 | 0.03649 |
| cg07404418 | *ALKBH4* | 54784 | 0.16 | 0.03649 |
| cg25685640 | *E2F8* | 79733 | 0.11 | 0.03649 |
| cg07896225 | *BCL7C* | 9274 | 0.10 | 0.03650 |
| cg01355739 | *GNAS* | 2778 | -0.60 | 0.03651 |
| cg05064352 | *PHF21B* | 112885 | 0.12 | 0.03651 |
| cg06904813 | *PNMA6A* | 84968 | -0.75 | 0.03651 |
| cg07401901 | *DAAM1* | 23002 | 0.12 | 0.03651 |
| cg09331011 | *GNAL* | 2774 | 0.13 | 0.03651 |
| cg10098216 | *NDUFB8* | 4714 | 0.15 | 0.03651 |
| cg10423830 | *PGEA1* | 25776 | 0.13 | 0.03651 |
| cg17756392 | *CLPP* | 8192 | 0.13 | 0.03651 |
| cg18854045 | *FLJ33534* | 285150 | -0.11 | 0.03651 |
| cg24012157 | *DDX50* | 79009 | 0.13 | 0.03651 |
| cg25416014 | *MCF2L2* | 23101 | 0.10 | 0.03651 |
| cg17417054 | *CLSPN* | 63967 | 0.17 | 0.03651 |
| cg22307649 | *TRAPPC4* | 51399 | 0.11 | 0.03651 |
| cg15630759 | *SF3A1* | 10291 | 0.14 | 0.03653 |
| cg22231902 | *EN1* | 2019 | 0.13 | 0.03653 |
| cg00057593 | *GML* | 2765 | -0.31 | 0.03654 |
| cg04397441 | *OGT* | 8473 | 0.12 | 0.03655 |
| cg10141022 | *BRAF* | 673 | 0.19 | 0.03655 |
| cg02885771 | *LTV1* | 84946 | -0.39 | 0.03658 |
| cg00186141 | *PODXL* | 5420 | 0.12 | 0.03659 |
| cg07906724 | *CHRNA6* | 8973 | 0.44 | 0.03659 |
| cg01600189 | *FLJ20444* | 403323 | 0.26 | 0.03660 |
| cg08847636 | *ZNF442* | 79973 | 0.11 | 0.03660 |
| cg18813928 | *RUNX3* | 864 | -0.08 | 0.03660 |
| cg20371891 | *RAB20* | 55647 | 0.09 | 0.03660 |
| cg01657408 | *FLJ31951* | 153830 | 0.13 | 0.03660 |
| cg02043070 | *ADSSL1* | 122622 | 0.11 | 0.03660 |
| cg05019152 | *FAM82C* | 55177 | 0.10 | 0.03660 |
| cg05723825 | *TEKT4* | 150483 | 0.20 | 0.03660 |
| cg06948937 | *PCTK3* | 5129 | -0.35 | 0.03660 |
| cg08176609 | *POMGNT1* | 55624 | 0.12 | 0.03660 |
| cg10348643 | *C1orf198* | 84886 | 0.12 | 0.03660 |
| cg11147886 | *NFAT5* | 10725 | 0.33 | 0.03660 |
| cg12476968 | *SLC12A6* | 9990 | 0.44 | 0.03660 |
| cg16270091 | *QRICH1* | 54870 | 0.16 | 0.03660 |
| cg17010112 | *GENX-3414* | 8987 | 0.15 | 0.03660 |
| cg18992688 | *AVPR1B* | 553 | 0.13 | 0.03660 |
| cg19017193 | *ZNF161* | 7716 | 0.15 | 0.03660 |
| cg20164720 | *AGTRAP* | 57085 | 0.15 | 0.03660 |
| cg22051865 | *ARHGEF17* | 9828 | 0.10 | 0.03660 |
| cg22286764 | *C3orf35* | 339883 | 0.32 | 0.03660 |
| cg24909737 | *EBPL* | 84650 | 0.13 | 0.03660 |
| cg26315493 | *VCP* | 7415 | 0.14 | 0.03660 |
| cg00202711 | *HTR2C* | 3358 | 0.18 | 0.03664 |
| cg05698090 | *CERK* | 64781 | 0.14 | 0.03664 |
| cg12454725 | *SYT17* | 51760 | 0.12 | 0.03664 |
| cg18630855 | *CEBPZ* | 10153 | 0.13 | 0.03664 |
| cg19287277 | *RKHD3* | 84206 | 0.12 | 0.03664 |
| cg21235838 | *SORBS1* | 10580 | 0.12 | 0.03664 |
| cg24304714 | *LCE1C* | 353133 | 0.38 | 0.03664 |
| cg00094319 | *PABPC3* | 5042 | -0.34 | 0.03665 |
| cg01246254 | *PLEKHA1* | 59338 | 0.11 | 0.03665 |
| cg03776850 | *TPSD1* | 23430 | -0.08 | 0.03665 |
| cg05109049 | *EVI2B* | 2124 | 0.35 | 0.03665 |
| cg05911610 | *MRPL3* | 11222 | 0.12 | 0.03665 |
| cg07168556 | *WFIKKN2* | 124857 | -0.54 | 0.03665 |
| cg08048656 | *SMARCE1* | 6605 | 0.10 | 0.03665 |
| cg09194436 | *ALDH6A1* | 4329 | 0.08 | 0.03665 |
| cg09303642 | *NFE2* | 4778 | 0.64 | 0.03665 |
| cg10491648 | *WDR54* | 84058 | 0.13 | 0.03665 |
| cg11780934 | *ANKRD13B* | 124930 | 0.11 | 0.03665 |
| cg12254515 | *DERL1* | 79139 | 0.10 | 0.03665 |
| cg12532169 | *NDN* | 4692 | -0.53 | 0.03665 |
| cg13347296 | *PFDN4* | 5203 | 0.12 | 0.03665 |
| cg13932624 | *TBRG1* | 84897 | 0.13 | 0.03665 |
| cg14319409 | *GLRA1* | 2741 | 0.21 | 0.03665 |
| cg14506668 | *FHL1* | 2273 | 0.12 | 0.03665 |
| cg14727952 | *BIRC2* | 329 | 0.10 | 0.03665 |
| cg15759055 | *RAPH1* | 65059 | 0.15 | 0.03665 |
| cg17050100 | *XAB1* | 11321 | 0.12 | 0.03665 |
| cg17771150 | *LCP1* | 3936 | 0.47 | 0.03665 |
| cg18071006 | *ULBP3* | 79465 | 0.10 | 0.03665 |
| cg18895972 | *PELO* | 53918 | 0.19 | 0.03665 |
| cg19046959 | *COL8A2* | 1296 | 0.42 | 0.03665 |
| cg20283107 | *FAM91A1* | 157769 | 0.36 | 0.03665 |
| cg21434440 | *CD2AP* | 23607 | 0.09 | 0.03665 |
| cg21492378 | *CEP1* | 11064 | 0.40 | 0.03665 |
| cg22762951 | *NVL* | 4931 | 0.15 | 0.03665 |
| cg24054653 | *C1GALT1C1* | 29071 | 0.11 | 0.03665 |
| cg25466368 | *HES1* | 3280 | 0.09 | 0.03665 |
| cg25937494 | *SREBF2* | 6721 | 0.09 | 0.03665 |
| cg26668713 | *SIPA1* | 6494 | 0.11 | 0.03665 |
| cg26885858 | *NBPF3* | 84224 | 0.22 | 0.03665 |
| cg01946401 | *RUNX2* | 860 | 0.32 | 0.03666 |
| cg05365729 | *LOXL2* | 4017 | -0.52 | 0.03666 |
| cg07220939 | *SLC22A12* | 116085 | 0.41 | 0.03666 |
| cg07973390 | *CARF* | 55602 | 0.12 | 0.03666 |
| cg11539173 | *GNB1L* | 54584 | 0.11 | 0.03666 |
| cg12711814 | *ENO1* | 2023 | 0.09 | 0.03666 |
| cg12955127 | *ATP5F1* | 515 | -0.39 | 0.03666 |
| cg13506226 | *P2RY5* | 10161 | -0.12 | 0.03666 |
| cg15146720 | *GALNT11* | 63917 | 0.11 | 0.03666 |
| cg15157837 | *CNNM3* | 26505 | 0.13 | 0.03666 |
| cg18801691 | *DCC* | 1630 | 0.12 | 0.03666 |
| cg18947521 | *H3F3A* | 3020 | 0.13 | 0.03666 |
| cg19475870 | *CDH9* | 1007 | -0.46 | 0.03666 |
| cg23269489 | *BCORL1* | 63035 | 0.13 | 0.03666 |
| cg24429836 | *LDHD* | 197257 | -0.45 | 0.03666 |
| cg03544525 | *PFKFB2* | 5208 | 0.12 | 0.03667 |
| cg02300906 | *FANCF* | 2188 | 0.13 | 0.03667 |
| cg02417264 | *RAMP3* | 10268 | -0.60 | 0.03667 |
| cg02471507 | *CHMP4C* | 92421 | 0.09 | 0.03667 |
| cg02630888 | *FBXL5* | 26234 | -0.80 | 0.03667 |
| cg02932167 | *ECEL1* | 9427 | -0.80 | 0.03667 |
| cg03912013 | *SACM1L* | 22908 | 0.14 | 0.03667 |
| cg04296434 | *Kua* | 387521 | 0.10 | 0.03667 |
| cg05755354 | *FRMD4A* | 55691 | -0.44 | 0.03667 |
| cg06061892 | *SNX5* | 27131 | 0.09 | 0.03667 |
| cg06282059 | *UBE2E1* | 7324 | -0.34 | 0.03667 |
| cg09314766 | *ADAM2* | 2515 | -0.60 | 0.03667 |
| cg10294200 | *C12orf30* | 80018 | 0.11 | 0.03667 |
| cg10494773 | *SRP54* | 6729 | 0.13 | 0.03667 |
| cg13208492 | *TSN* | 7247 | 0.10 | 0.03667 |
| cg14982472 | *ABCG1* | 9619 | 0.37 | 0.03667 |
| cg15791248 | *FGFR1* | 2260 | -0.48 | 0.03667 |
| cg15910079 | *RNASE3* | 6037 | 0.45 | 0.03667 |
| cg16605590 | *MKI67IP* | 84365 | 0.15 | 0.03667 |
| cg18830459 | *IBRDC3* | 127544 | 0.10 | 0.03667 |
| cg18891387 | *NDUFA11* | 126328 | 0.09 | 0.03667 |
| cg19403023 | *TESSP1* | 360226 | -0.48 | 0.03667 |
| cg19845843 | *CXorf20* | 139105 | -0.46 | 0.03667 |
| cg20559561 | *RNF10* | 9921 | 0.12 | 0.03667 |
| cg20630207 | *GPSN2* | 9524 | 0.24 | 0.03667 |
| cg20918903 | *JMJD1A* | 55818 | 0.11 | 0.03667 |
| cg21130124 | *CALML5* | 51806 | -0.12 | 0.03667 |
| cg22967284 | *SLIT1* | 6585 | 0.10 | 0.03667 |
| cg23306832 | *KIAA0999* | 23387 | 0.14 | 0.03667 |
| cg24199834 | *POU4F2* | 5458 | 0.16 | 0.03667 |
| cg25258291 | *FARP1* | 10160 | 0.11 | 0.03667 |
| cg01747665 | *IGFALS* | 3483 | 0.10 | 0.03668 |
| cg01849789 | *ZC3H7B* | 23264 | 0.13 | 0.03668 |
| cg07457944 | *SOCS6* | 9306 | 0.12 | 0.03668 |
| cg08116137 | *NAT8* | 9027 | 0.36 | 0.03668 |
| cg10634115 | *UTS2R* | 2837 | -0.69 | 0.03668 |
| cg10703331 | *TRIM24* | 8805 | 0.13 | 0.03668 |
| cg11221265 | *SDHC* | 6391 | 0.11 | 0.03668 |
| cg14723799 | *C14orf104* | 55172 | 0.12 | 0.03668 |
| cg16418163 | *ACPL2* | 92370 | 0.12 | 0.03668 |
| cg16884539 | *CXorf55* | 139804 | -0.60 | 0.03668 |
| cg21440587 | *AIF1* | 199 | 0.54 | 0.03668 |
| cg21514871 | *MEFV* | 4210 | 0.65 | 0.03668 |
| cg23273333 | *ESRRG* | 2104 | 0.25 | 0.03668 |
| cg25374813 | *SLC23A1* | 9963 | 0.40 | 0.03668 |
| cg27626318 | *VAC14* | 55697 | 0.10 | 0.03668 |
| cg07979577 | *PPP2R5A* | 5525 | 0.12 | 0.03671 |
| cg00024812 | *CPSF3* | 51692 | 0.11 | 0.03673 |
| cg00183916 | *HS3ST3B1* | 9953 | 0.11 | 0.03673 |
| cg00268338 | *HECTD2* | 143279 | 0.09 | 0.03673 |
| cg00642303 | *CENTB2* | 23527 | 0.09 | 0.03673 |
| cg00645579 | *IRF7* | 3665 | 0.67 | 0.03673 |
| cg00673020 | *MOSPD1* | 56180 | -0.56 | 0.03673 |
| cg00852964 | *VNN1* | 8876 | 0.44 | 0.03673 |
| cg00940278 | *GATA6* | 2627 | 0.11 | 0.03673 |
| cg01065920 | *NDUFA12* | 55967 | 0.11 | 0.03673 |
| cg01086895 | *DCHS1* | 8642 | 0.16 | 0.03673 |
| cg01104423 | *EIF2AK1* | 27102 | 0.16 | 0.03673 |
| cg01124961 | *RC74* | 55756 | 0.10 | 0.03673 |
| cg01278696 | *SMARCA5* | 8467 | 0.11 | 0.03673 |
| cg01410472 | *CRISPLD1* | 83690 | 0.10 | 0.03673 |
| cg01610488 | *TRPA1* | 8989 | 0.12 | 0.03673 |
| cg01795697 | *DDX5* | 1655 | 0.12 | 0.03673 |
| cg01837719 | *FUT9* | 10690 | 0.09 | 0.03673 |
| cg01980637 | *PER1* | 5187 | 0.07 | 0.03673 |
| cg02101486 | *DLX5* | 1749 | 0.17 | 0.03673 |
| cg02113971 | *FLJ20859* | 64745 | 0.11 | 0.03673 |
| cg02131995 | *HAMP* | 57817 | 0.31 | 0.03673 |
| cg02150709 | *PPIH* | 10465 | 0.11 | 0.03673 |
| cg02153871 | *HARSL* | 23438 | 0.14 | 0.03673 |
| cg02608019 | *SYK* | 6850 | 0.14 | 0.03673 |
| cg02620769 | *CCDC65* | 85478 | 0.08 | 0.03673 |
| cg02900980 | *FOXP1* | 27086 | 0.12 | 0.03673 |
| cg02913364 | *UBE2J2* | 118424 | 0.14 | 0.03673 |
| cg02968557 | *HSPB9* | 94086 | -0.57 | 0.03673 |
| cg03007571 | *HIST1H2BD* | 3017 | 0.10 | 0.03673 |
| cg03104936 | *GRB10* | 2887 | -0.47 | 0.03673 |
| cg04281204 | *DCLRE1A* | 9937 | -0.36 | 0.03673 |
| cg04396791 | *SHANK2* | 22941 | 0.12 | 0.03673 |
| cg04401436 | *FLJ90709* | 153129 | 0.10 | 0.03673 |
| cg04605667 | *SIAH2* | 6478 | 0.15 | 0.03673 |
| cg04632671 | *PPARG* | 5468 | 0.12 | 0.03673 |
| cg04653308 | *PPP2R4* | 5524 | 0.73 | 0.03673 |
| cg04702045 | *OPHN1* | 4983 | 0.14 | 0.03673 |
| cg04960951 | *SUV420H2* | 84787 | 0.11 | 0.03673 |
| cg05348272 | *TBCD* | 6904 | 0.13 | 0.03673 |
| cg05439368 | *RNF36* | 140691 | 0.49 | 0.03673 |
| cg05590567 | *METTL2B* | 55798 | 0.08 | 0.03673 |
| cg05751148 | *PTPRCAP* | 5790 | 0.33 | 0.03673 |
| cg05798972 | *PPARBP* | 5469 | 0.12 | 0.03673 |
| cg05915281 | *IRF1* | 3659 | 0.10 | 0.03673 |
| cg06026755 | *CEP27* | 55142 | -0.40 | 0.03673 |
| cg06101324 | *SPRR1A* | 6698 | 0.67 | 0.03673 |
| cg06146910 | *KBTBD6* | 89890 | 0.09 | 0.03673 |
| cg06186973 | *YWHAG* | 7532 | 0.14 | 0.03673 |
| cg06238491 | *LAIR1* | 3903 | 0.45 | 0.03673 |
| cg06245154 | *ERCC2* | 2068 | 0.12 | 0.03673 |
| cg06557358 | *LOC124842* | 124842 | 0.11 | 0.03673 |
| cg06665984 | *TRIP11* | 9321 | 0.10 | 0.03673 |
| cg06746101 | *TMED3* | 23423 | 0.10 | 0.03673 |
| cg06786424 | *PRPF4B* | 8899 | 0.10 | 0.03673 |
| cg06886022 | *C9orf75* | 286262 | 0.10 | 0.03673 |
| cg07207789 | *CRISPLD2* | 83716 | -0.57 | 0.03673 |
| cg07211259 | *PDCD1LG2* | 80380 | 0.44 | 0.03673 |
| cg07330329 | *TRIM48* | 79097 | 0.28 | 0.03673 |
| cg07395788 | *PDSS2* | 57107 | 0.13 | 0.03673 |
| cg07570142 | *MOXD1* | 26002 | 0.11 | 0.03673 |
| cg07900766 | *SPAG16* | 79582 | 0.11 | 0.03673 |
| cg08203715 | *ST3GAL4* | 6484 | 0.12 | 0.03673 |
| cg08245789 | *FLJ25421* | 150350 | -0.52 | 0.03673 |
| cg08583049 | *FOXB1* | 27023 | 0.09 | 0.03673 |
| cg08673615 | *HMGCL* | 3155 | 0.10 | 0.03673 |
| cg08736146 | *MRPL10* | 124995 | 0.09 | 0.03673 |
| cg08827391 | *GDI2* | 2665 | 0.13 | 0.03673 |
| cg08999486 | *CDC42BPB* | 9578 | 0.13 | 0.03673 |
| cg09254939 | *KLK10* | 5655 | 0.18 | 0.03673 |
| cg09351894 | *CREM* | 1390 | 0.14 | 0.03673 |
| cg09390902 | *NSUN5C* | 260294 | 0.12 | 0.03673 |
| cg09464487 | *ACTR6* | 64431 | 0.11 | 0.03673 |
| cg09479906 | *C10orf88* | 80007 | 0.08 | 0.03673 |
| cg09503780 | *GORASP2* | 26003 | 0.10 | 0.03673 |
| cg09556292 | *RCOR1* | 23186 | 0.10 | 0.03673 |
| cg09787254 | *FA2H* | 79152 | 0.09 | 0.03673 |
| cg09916572 | *ZP3* | 7784 | 0.14 | 0.03673 |
| cg09989883 | *MRPS22* | 56945 | 0.11 | 0.03673 |
| cg10038259 | *HNRPAB* | 3182 | 0.13 | 0.03673 |
| cg10194829 | *FGF17* | 8822 | 0.41 | 0.03673 |
| cg10309835 | *KDELR1* | 10945 | 0.16 | 0.03673 |
| cg10361696 | *FAM109A* | 144717 | 0.19 | 0.03673 |
| cg10451565 | *GPR77* | 27202 | 0.34 | 0.03673 |
| cg11035216 | *RASSF1* | 11186 | 0.12 | 0.03673 |
| cg11100802 | *TRIM52* | 84851 | 0.11 | 0.03673 |
| cg11646757 | *HIG2* | 29923 | 0.14 | 0.03673 |
| cg11668844 | *MCF2L* | 23263 | 0.38 | 0.03673 |
| cg12091331 | *PLAT* | 5327 | 0.46 | 0.03673 |
| cg12322132 | *IGF2AS* | 51214 | 0.10 | 0.03673 |
| cg12403575 | *TRADD* | 8717 | 0.13 | 0.03673 |
| cg12582959 | *HSU79303* | 29903 | 0.10 | 0.03673 |
| cg12762680 | *PSMB9* | 5698 | 0.12 | 0.03673 |
| cg12841020 | *PGF* | 5228 | -0.48 | 0.03673 |
| cg12873941 | *MOAP1* | 64112 | 0.15 | 0.03673 |
| cg12909005 | *ZNRD1* | 30834 | 0.11 | 0.03673 |
| cg13150977 | *FLJ43276* | 388165 | 0.14 | 0.03673 |
| cg13230584 | *GSTK1* | 373156 | 0.11 | 0.03673 |
| cg13320626 | *ST8SIA4* | 7903 | 0.10 | 0.03673 |
| cg13650137 | *ASPM* | 259266 | 0.09 | 0.03673 |
| cg13726507 | *CTAG2* | 30848 | -0.55 | 0.03673 |
| cg14120436 | *GNB5* | 10681 | 0.31 | 0.03673 |
| cg14147105 | *LOC93343* | 93343 | 0.12 | 0.03673 |
| cg14174099 | *SLC8A3* | 6547 | 0.11 | 0.03673 |
| cg14299800 | *TOR1B* | 27348 | -0.69 | 0.03673 |
| cg14311811 | *DSG2* | 1829 | 0.17 | 0.03673 |
| cg14400118 | *MMP2* | 4313 | 0.15 | 0.03673 |
| cg14694952 | *HD* | 3064 | 0.10 | 0.03673 |
| cg14840416 | *ZFP36L2* | 678 | 0.19 | 0.03673 |
| cg14947494 | *BRP44L* | 51660 | 0.11 | 0.03673 |
| cg15025769 | *MYO9A* | 4649 | 0.11 | 0.03673 |
| cg15087147 | *LRRC4* | 64101 | 0.10 | 0.03673 |
| cg15207619 | *CAB39L* | 81617 | 0.41 | 0.03673 |
| cg15269863 | *PRSS35* | 167681 | 0.17 | 0.03673 |
| cg15357945 | *PRG2* | 5553 | 0.47 | 0.03673 |
| cg15379858 | *ChGn* | 55790 | 0.73 | 0.03673 |
| cg15383574 | *ATP5S* | 27109 | 0.15 | 0.03673 |
| cg15384717 | *PRPF3* | 9129 | 0.18 | 0.03673 |
| cg15477600 | *SDF2L1* | 23753 | 0.14 | 0.03673 |
| cg15519208 | *HIST1H1E* | 3008 | 0.16 | 0.03673 |
| cg15659828 | *CHID1* | 66005 | 0.09 | 0.03673 |
| cg15739944 | *HABP4* | 22927 | 0.13 | 0.03673 |
| cg16119128 | *FLJ43339* | 388115 | 0.33 | 0.03673 |
| cg16137862 | *BCL2L1* | 598 | 0.11 | 0.03673 |
| cg16254309 | *CNTNAP2* | 26047 | 0.15 | 0.03673 |
| cg16256643 | *NDUFB7* | 4713 | 0.18 | 0.03673 |
| cg16276199 | *PPP1CB* | 5500 | 0.10 | 0.03673 |
| cg16343696 | *KPNA6* | 23633 | 0.11 | 0.03673 |
| cg16366323 | *DUSP3* | 1845 | 0.07 | 0.03673 |
| cg16494477 | *FGF18* | 8817 | 0.11 | 0.03673 |
| cg16806597 | *GRAP* | 10750 | 0.77 | 0.03673 |
| cg16878021 |  | 3500 | -0.10 | 0.03673 |
| cg16954928 | *LOC147808* | 147808 | 0.12 | 0.03673 |
| cg17083853 | *SURF1* | 6834 | 0.14 | 0.03673 |
| cg17346022 | *SNX6* | 58533 | -0.37 | 0.03673 |
| cg17383958 | *RRAS* | 6237 | 0.12 | 0.03673 |
| cg17400229 | *U2AF1* | 7307 | 0.11 | 0.03673 |
| cg17572791 | *HAX1* | 10456 | 0.07 | 0.03673 |
| cg18042079 | *C17orf40* | 55813 | 0.10 | 0.03673 |
| cg18139769 | *SGCE* | 8910 | -0.64 | 0.03673 |
| cg18250832 | *NMUR1* | 10316 | -0.75 | 0.03673 |
| cg18272264 | *CACNB4* | 785 | 0.11 | 0.03673 |
| cg18393014 | *SETDB2* | 83852 | 0.11 | 0.03673 |
| cg18437633 | *ITGB6* | 3694 | -0.14 | 0.03673 |
| cg18596794 | *MPZL1* | 9019 | -0.49 | 0.03673 |
| cg18665023 | *ATP5O* | 539 | 0.10 | 0.03673 |
| cg18824775 | *PX19* | 27166 | 0.13 | 0.03673 |
| cg18846362 | *CA12* | 771 | 0.09 | 0.03673 |
| cg18895813 | *ADAMTS5* | 11096 | 0.11 | 0.03673 |
| cg18959478 | *PRKCDBP* | 112464 | 0.10 | 0.03673 |
| cg19055458 | *MYLIP* | 29116 | 0.11 | 0.03673 |
| cg19219920 | *FAM53B* | 9679 | 0.16 | 0.03673 |
| cg19286986 | *GPR162* | 27239 | 0.48 | 0.03673 |
| cg19526626 | *ZNF491* | 126069 | 0.15 | 0.03673 |
| cg19567046 | *VPS25* | 84313 | 0.13 | 0.03673 |
| cg19591206 | *SLCO2A1* | 6578 | 0.12 | 0.03673 |
| cg19730092 | *ADAMTS14* | 140766 | -0.45 | 0.03673 |
| cg20028470 | *UCN* | 7349 | -0.89 | 0.03673 |
| cg20053799 | *PSCD2* | 9266 | 0.12 | 0.03673 |
| cg20126158 | *MARCH6* | 10299 | 0.11 | 0.03673 |
| cg20168375 | *LOC90624* | 90624 | 0.10 | 0.03673 |
| cg20176989 | *KIR3DL2* | 3812 | 0.26 | 0.03673 |
| cg20291846 | *ADAM17* | 6868 | 0.19 | 0.03673 |
| cg20516209 | *EMILIN1* | 11117 | 0.28 | 0.03673 |
| cg20641580 | *LOC91689* | 91689 | 0.08 | 0.03673 |
| cg20701850 | *RECQL* | 5965 | 0.12 | 0.03673 |
| cg20768743 | *CD226* | 10666 | 0.20 | 0.03673 |
| cg20815063 | *MRE11A* | 4361 | 0.08 | 0.03673 |
| cg20917484 | *GAPDH* | 2597 | 0.15 | 0.03673 |
| cg20925482 | *C20orf111* | 51526 | 0.10 | 0.03673 |
| cg21132104 | *SPATA5L1* | 79029 | 0.15 | 0.03673 |
| cg21179457 | *INSM2* | 84684 | 0.14 | 0.03673 |
| cg21458116 | *GGA1* | 26088 | 0.13 | 0.03673 |
| cg21472642 | *CHN2* | 1124 | -0.52 | 0.03673 |
| cg21571937 | *FZD2* | 2535 | 0.12 | 0.03673 |
| cg21800088 | *POLR2B* | 5431 | 0.07 | 0.03673 |
| cg21803680 | *LOC92691* | 92691 | 0.11 | 0.03673 |
| cg21973276 | *SMAD6* | 4091 | 0.11 | 0.03673 |
| cg22227058 | *SLU7* | 10569 | 0.10 | 0.03673 |
| cg22318989 | *NPR1* | 4881 | 0.17 | 0.03673 |
| cg22637507 | *ALKBH3* | 221120 | 0.11 | 0.03673 |
| cg22655224 | *MT3* | 4504 | 0.10 | 0.03673 |
| cg22657536 | *LSM16* | 80153 | 0.11 | 0.03673 |
| cg22728790 | *PIN1* | 5300 | 0.08 | 0.03673 |
| cg22821930 | *RNPC2* | 9584 | 0.11 | 0.03673 |
| cg22901840 | *DIRAS3* | 9077 | -0.58 | 0.03673 |
| cg23179168 | *FAM84B* | 157638 | 0.13 | 0.03673 |
| cg23181133 | *CEACAM3* | 1084 | 0.39 | 0.03673 |
| cg23205183 | *NECAP1* | 25977 | -0.35 | 0.03673 |
| cg23632840 | *MKKS* | 8195 | 0.11 | 0.03673 |
| cg23838179 | *ZNF592* | 9640 | 0.10 | 0.03673 |
| cg24237439 | *FLJ25422* | 202151 | 0.28 | 0.03673 |
| cg24247865 | *CHORDC1* | 26973 | 0.12 | 0.03673 |
| cg24289167 | *POLR3A* | 11128 | 0.18 | 0.03673 |
| cg24323434 | *SERF2* | 10169 | 0.13 | 0.03673 |
| cg24648715 | *TCEAL3* | 85012 | 0.12 | 0.03673 |
| cg24687335 | *SLC37A4* | 2542 | 0.12 | 0.03673 |
| cg24693620 | *RAB5B* | 5869 | 0.11 | 0.03673 |
| cg24860534 | *CDC42BPA* | 8476 | -0.64 | 0.03673 |
| cg25350411 | *PLAGL1* | 5325 | -0.30 | 0.03673 |
| cg25477181 | *PITPNB* | 23760 | 0.10 | 0.03673 |
| cg25591670 | *CXorf26* | 51260 | 0.10 | 0.03673 |
| cg25624712 | *LOC92345* | 92345 | 0.11 | 0.03673 |
| cg26152597 | *ZBTB20* | 26137 | 0.18 | 0.03673 |
| cg26158194 | *LSP1* | 4046 | 0.44 | 0.03673 |
| cg26533949 | *DZIP1L* | 199221 | 0.10 | 0.03673 |
| cg26538349 | *FLJ20035* | 55601 | 0.10 | 0.03673 |
| cg26606552 | *CCDC22* | 28952 | 0.13 | 0.03673 |
| cg26608032 | *MKL1* | 57591 | 0.10 | 0.03673 |
| cg26787505 | *TLCD1* | 116238 | 0.11 | 0.03673 |
| cg26985201 | *LRCH3* | 84859 | 0.10 | 0.03673 |
| cg27258787 | *ILF3* | 3609 | 0.12 | 0.03673 |
| cg00393585 | *FLJ31659* | 152756 | 0.14 | 0.03673 |
| cg17491456 | *DAB2* | 1601 | 0.10 | 0.03673 |
| cg20811607 | *SMPD2* | 6610 | -0.58 | 0.03673 |
| cg20979602 | *PAPD4* | 167153 | 0.11 | 0.03673 |
| cg26240939 | *LOC57149* | 57149 | -0.55 | 0.03673 |
| cg26856388 | *VKORC1L1* | 154807 | 0.11 | 0.03673 |
| cg25985437 | *FAM96A* | 84191 | 0.10 | 0.03675 |
| cg01343729 | *ZNF688* | 146542 | 0.11 | 0.03675 |
| cg02527529 | *C17orf53* | 78995 | 0.09 | 0.03675 |
| cg02631622 | *SIAH1* | 6477 | 0.08 | 0.03675 |
| cg03117949 | *USP16* | 10600 | 0.16 | 0.03675 |
| cg03172991 | *NFIX* | 4784 | -0.24 | 0.03675 |
| cg03732295 | *IMPAD1* | 54928 | 0.12 | 0.03675 |
| cg04144788 | *OXCT2* | 64064 | 0.12 | 0.03675 |
| cg04383012 | *PSMD1* | 5707 | 0.09 | 0.03675 |
| cg04969878 | *TMEM19* | 55266 | -0.43 | 0.03675 |
| cg05442902 | *P2RXL1* | 9127 | -0.39 | 0.03675 |
| cg05852416 | *NFKBIA* | 4792 | 0.12 | 0.03675 |
| cg06193382 | *FAM54A* | 113115 | 0.07 | 0.03675 |
| cg07469792 | *RASSF8* | 11228 | 0.10 | 0.03675 |
| cg07711097 | *GML* | 2765 | -0.52 | 0.03675 |
| cg07851192 | *KCNG2* | 26251 | -0.91 | 0.03675 |
| cg08307963 | *GJA5* | 2702 | 0.44 | 0.03675 |
| cg08728865 | *NALP7* | 199713 | -0.10 | 0.03675 |
| cg08773844 | *CCDC25* | 55246 | 0.10 | 0.03675 |
| cg09060849 | *GGA2* | 23062 | 0.16 | 0.03675 |
| cg09113613 | *KIAA1219* | 57148 | 0.10 | 0.03675 |
| cg09351449 | *C20orf172* | 79980 | 0.11 | 0.03675 |
| cg09515805 | *PGM2L1* | 283209 | 0.11 | 0.03675 |
| cg09573435 | *SEMA6A* | 57556 | 0.12 | 0.03675 |
| cg09584711 | *HPR* | 3250 | 0.33 | 0.03675 |
| cg09914444 | *DMBX1* | 127343 | 0.22 | 0.03675 |
| cg10039928 | *MATN2* | 4147 | -0.56 | 0.03675 |
| cg10289190 | *GRHPR* | 9380 | 0.09 | 0.03675 |
| cg10507231 | *PIK3C2B* | 5287 | -0.08 | 0.03675 |
| cg10536625 | *IFT140* | 9742 | -0.10 | 0.03675 |
| cg13454184 | *TARSL1* | 80222 | 0.11 | 0.03675 |
| cg14184817 | *XTP7* | 90332 | -0.78 | 0.03675 |
| cg14700577 | *SCAP* | 22937 | 0.10 | 0.03675 |
| cg14759043 | *MGC4266* | 84766 | 0.13 | 0.03675 |
| cg14946299 | *ZNF273* | 10793 | 0.17 | 0.03675 |
| cg15374234 | *CD300LF* | 146722 | 0.50 | 0.03675 |
| cg15399561 | *HRSP12* | 10247 | 0.11 | 0.03675 |
| cg15589156 | *RAB3IL1* | 5866 | 0.09 | 0.03675 |
| cg15842884 | *KIAA1434* | 56261 | 0.09 | 0.03675 |
| cg16584573 | *FGF8* | 2253 | 0.09 | 0.03675 |
| cg16898150 | *CCBL1* | 883 | 0.11 | 0.03675 |
| cg17404915 | *OVOL2* | 58495 | 0.10 | 0.03675 |
| cg18587018 | *CRSP9* | 9443 | 0.09 | 0.03675 |
| cg18630040 | *PLA2G7* | 7941 | 0.12 | 0.03675 |
| cg19192316 | *SIRT1* | 23411 | -0.40 | 0.03675 |
| cg19317638 | *SFRS16* | 11129 | 0.19 | 0.03675 |
| cg19549068 | *CLU* | 1191 | 0.08 | 0.03675 |
| cg19713460 | *SYNGR1* | 9145 | 0.10 | 0.03675 |
| cg19836199 | *RUNX1* | 861 | 0.38 | 0.03675 |
| cg19872814 | *PLAA* | 9373 | 0.09 | 0.03675 |
| cg20206574 | *DSCR1* | 1827 | 0.19 | 0.03675 |
| cg20330472 | *EYA4* | 2070 | 0.11 | 0.03675 |
| cg21351994 | *EMX1* | 2016 | 0.13 | 0.03675 |
| cg21808053 | *DIRAS3* | 9077 | -0.54 | 0.03675 |
| cg21820677 | *GABRA2* | 2555 | 0.14 | 0.03675 |
| cg21954346 | *RBM35B* | 80004 | 0.12 | 0.03675 |
| cg22606869 | *TUBA6* | 84790 | 0.09 | 0.03675 |
| cg22831514 | *DHRS8* | 51170 | 0.14 | 0.03675 |
| cg22975913 | *WT1* | 7490 | 0.15 | 0.03675 |
| cg22984616 | *BAG2* | 9532 | 0.14 | 0.03675 |
| cg23346960 | *ZFP36* | 7538 | 0.13 | 0.03675 |
| cg23355492 | *SP6* | 80320 | 0.44 | 0.03675 |
| cg24222435 | *LOC221143* | 221143 | 0.11 | 0.03675 |
| cg24715245 | *UCHL1* | 7345 | 0.10 | 0.03675 |
| cg24749970 | *ALS2CR2* | 55437 | 0.12 | 0.03675 |
| cg24797967 | *FLJ39739* | 388685 | 0.32 | 0.03675 |
| cg25536300 | *PTBP1* | 5725 | 0.08 | 0.03675 |
| cg26399201 | *ANKRD43* | 134548 | 0.12 | 0.03675 |
| cg26428825 | *KCNV2* | 169522 | -0.52 | 0.03675 |
| cg26990660 | *RAMP2* | 10266 | 0.18 | 0.03675 |
| cg27504117 | *ANKMY1* | 51281 | -0.55 | 0.03675 |
| cg27560922 | *ARHGDIG* | 398 | 0.12 | 0.03675 |
| cg00581156 | *HSA9761* | 27292 | 0.12 | 0.03675 |
| cg07990489 | *NUP35* | 129401 | 0.09 | 0.03675 |
| cg22391400 | *SLK* | 9748 | 0.11 | 0.03675 |
| cg02322989 | *LRP1B* | 53353 | 0.14 | 0.03676 |
| cg05786809 | *CKB* | 1152 | 0.10 | 0.03676 |
| cg20478514 | *SCCPDH* | 51097 | 0.09 | 0.03676 |
| cg24098951 | *LRP12* | 29967 | 0.12 | 0.03676 |
| cg11516606 | *TDRD7* | 23424 | 0.11 | 0.03676 |
| cg19258882 | *ERBB3* | 2065 | -0.59 | 0.03680 |
| cg06307601 | *FAM49B* | 51571 | 0.12 | 0.03680 |
| cg06665799 | *TMED9* | 54732 | 0.10 | 0.03680 |
| cg12291552 | *GOLPH4* | 27333 | 0.10 | 0.03680 |
| cg14333565 | *NRTN* | 4902 | -0.10 | 0.03680 |
| cg14336578 | *LETM2* | 137994 | 0.11 | 0.03680 |
| cg15014458 | *LYPD3* | 27076 | -0.44 | 0.03680 |
| cg15422784 | *LRRC8B* | 23507 | -0.09 | 0.03680 |
| cg16953064 | *TXNL4B* | 54957 | 0.10 | 0.03680 |
| cg23074401 | *DDX10* | 1662 | 0.10 | 0.03680 |
| cg00167504 | *BLVRA* | 644 | 0.09 | 0.03682 |
| cg01427567 | *TMC4* | 147798 | 0.60 | 0.03682 |
| cg02659086 | *GSTP1* | 2950 | 0.09 | 0.03682 |
| cg08308286 | *ET* | 79157 | 0.12 | 0.03682 |
| cg09952204 | *RASGRF2* | 5924 | 0.09 | 0.03682 |
| cg13255096 | *MAP3K5* | 4217 | 0.10 | 0.03682 |
| cg14023451 | *GPLD1* | 2822 | 0.51 | 0.03682 |
| cg16284168 | *AP4M1* | 9179 | 0.11 | 0.03682 |
| cg16338035 | *C3orf58* | 205428 | 0.10 | 0.03682 |
| cg20267005 | *TYROBP* | 7305 | 0.33 | 0.03682 |
| cg23834593 | *HNF4A* | 3172 | -0.32 | 0.03682 |
| cg23966705 | *SPAG9* | 9043 | 0.11 | 0.03682 |
| cg00009407 | *TTC8* | 123016 | 0.10 | 0.03683 |
| cg00053292 | *CCDC44* | 51204 | 0.12 | 0.03683 |
| cg00204262 | *ECH1* | 1891 | -0.48 | 0.03683 |
| cg00441136 | *KIF3B* | 9371 | -0.70 | 0.03683 |
| cg01143454 | *C20orf141* | 128653 | 0.50 | 0.03683 |
| cg01228667 | *GPR101* | 83550 | 0.10 | 0.03683 |
| cg01468621 | *BRSK2* | 9024 | 0.16 | 0.03683 |
| cg01472101 | *CLEC10A* | 10462 | 0.45 | 0.03683 |
| cg01651821 | *LOC129531* | 129531 | 0.11 | 0.03683 |
| cg01843018 | *C5* | 727 | -0.18 | 0.03683 |
| cg01910452 | *BCAP31* | 10134 | 0.17 | 0.03683 |
| cg02266731 | *CPM* | 1368 | 0.39 | 0.03683 |
| cg02312170 | *BRWD2* | 55717 | 0.11 | 0.03683 |
| cg02320454 | *GPR150* | 285601 | 0.16 | 0.03683 |
| cg02516189 | *CARD9* | 64170 | 0.43 | 0.03683 |
| cg02632166 | *ITM2B* | 9445 | 0.11 | 0.03683 |
| cg02649608 | *ZBTB33* | 10009 | 0.08 | 0.03683 |
| cg02675652 | *C12orf22* | 81566 | -0.44 | 0.03683 |
| cg02756614 | *FER1L3* | 26509 | 0.12 | 0.03683 |
| cg03091512 | *RHBDD1* | 84236 | 0.09 | 0.03683 |
| cg03391040 | *ZNF420* | 147923 | 0.10 | 0.03683 |
| cg03763616 | *SPIB* | 6689 | 0.40 | 0.03683 |
| cg03776551 | *ZNF396* | 252884 | 0.12 | 0.03683 |
| cg03821311 | *HIST1H1B* | 3009 | -0.41 | 0.03683 |
| cg04036714 | *NAPE-PLD* | 222236 | 0.09 | 0.03683 |
| cg04167054 | *ATP2C1* | 27032 | 0.14 | 0.03683 |
| cg04169469 | *ODC1* | 4953 | 0.11 | 0.03683 |
| cg04619854 | *UBXD4* | 165324 | 0.19 | 0.03683 |
| cg04710918 | *GSS* | 2937 | 0.10 | 0.03683 |
| cg04741636 | *DTX3L* | 151636 | 0.11 | 0.03683 |
| cg04916311 | *PQLC3* | 130814 | 0.14 | 0.03683 |
| cg05024638 | *POLG* | 5428 | 0.10 | 0.03683 |
| cg05098471 | *MEIS1* | 4211 | 0.12 | 0.03683 |
| cg05280555 | *CNOT10* | 25904 | 0.09 | 0.03683 |
| cg05669210 | *SLC12A9* | 56996 | 0.14 | 0.03683 |
| cg05774801 | *SFRP2* | 6423 | 0.10 | 0.03683 |
| cg05798712 | *FABP7* | 2173 | -0.32 | 0.03683 |
| cg05932408 | *PTGIS* | 5740 | 0.11 | 0.03683 |
| cg06583518 | *ZNF571* | 51276 | 0.11 | 0.03683 |
| cg06616245 | *GLI1* | 2735 | 0.12 | 0.03683 |
| cg06689536 | *CCNF* | 899 | 0.09 | 0.03683 |
| cg06699365 | *OSBP* | 5007 | 0.11 | 0.03683 |
| cg06726207 | *SIX4* | 51804 | 0.09 | 0.03683 |
| cg06992027 | *TAGLN2* | 8407 | 0.10 | 0.03683 |
| cg07156669 | *CPD* | 1362 | -0.41 | 0.03683 |
| cg07195622 | *SRRM2* | 23524 | 0.13 | 0.03683 |
| cg07236943 | *PEG10* | 23089 | 0.12 | 0.03683 |
| cg07240721 | *HINT3* | 135114 | 0.09 | 0.03683 |
| cg07314984 | *PHKA2* | 5256 | 0.19 | 0.03683 |
| cg07318658 | *MTMR4* | 9110 | 0.17 | 0.03683 |
| cg07327328 | *CEP250* | 11190 | 0.11 | 0.03683 |
| cg07359545 | *GP1BB* | 2812 | 0.12 | 0.03683 |
| cg07483064 | *ENO1* | 2023 | 0.10 | 0.03683 |
| cg07519011 | *TBC1D4* | 9882 | 0.15 | 0.03683 |
| cg07638871 | *DKFZP781I1119* | 166968 | 0.16 | 0.03683 |
| cg07806164 | *UBE2E2* | 7325 | 0.10 | 0.03683 |
| cg08364102 | *OSBPL2* | 9885 | -0.38 | 0.03683 |
| cg08422599 | *KCNIP1* | 30820 | 0.10 | 0.03683 |
| cg08424427 | *GNB2L1* | 10399 | 0.14 | 0.03683 |
| cg08679985 | *KLF17* | 128209 | -0.46 | 0.03683 |
| cg09082921 | *UNQ3045* | 389383 | 0.18 | 0.03683 |
| cg09539271 | *PTHB1* | 27241 | 0.10 | 0.03683 |
| cg09674215 | *TWIST1* | 7291 | 0.09 | 0.03683 |
| cg10222574 | *RAB11FIP2* | 22841 | 0.12 | 0.03683 |
| cg10283277 | *SNF1LK2* | 23235 | 0.19 | 0.03683 |
| cg10324224 | *TTC13* | 79573 | -0.42 | 0.03683 |
| cg10493166 | *PTGS1* | 5742 | 0.13 | 0.03683 |
| cg11141013 | *ZNF688* | 146542 | 0.09 | 0.03683 |
| cg11166252 | *AHR* | 196 | 0.12 | 0.03683 |
| cg11206667 | *FBXO36* | 130888 | 0.10 | 0.03683 |
| cg11291009 | *ARHGEF9* | 23229 | 0.10 | 0.03683 |
| cg11394785 | *LTC4S* | 4056 | 0.42 | 0.03683 |
| cg11458642 | *ATR* | 545 | 0.09 | 0.03683 |
| cg11510839 | *OCA2* | 4948 | 0.12 | 0.03683 |
| cg11912049 | *AQP6* | 363 | 0.14 | 0.03683 |
| cg11993300 | *NDST2* | 8509 | 0.12 | 0.03683 |
| cg11999384 | *GRN* | 2896 | 0.12 | 0.03683 |
| cg12402495 | *C1orf43* | 25912 | 0.10 | 0.03683 |
| cg12792011 | *ZNF329* | 79673 | 0.10 | 0.03683 |
| cg12979743 | *NDUFB5* | 4711 | 0.10 | 0.03683 |
| cg12991341 | *TMEM74* | 157753 | -0.44 | 0.03683 |
| cg13134662 | *VAV2* | 7410 | 0.08 | 0.03683 |
| cg13168820 | *PTPRT* | 11122 | 0.10 | 0.03683 |
| cg13265003 | *SLC37A1* | 54020 | 0.52 | 0.03683 |
| cg13578400 | *RCD-8* | 23644 | 0.09 | 0.03683 |
| cg13781408 | *AATK* | 9625 | -0.67 | 0.03683 |
| cg13808561 | *API5* | 8539 | 0.12 | 0.03683 |
| cg13919215 | *POLR2K* | 5440 | 0.09 | 0.03683 |
| cg14308291 | *EIF2C3* | 192669 | 0.14 | 0.03683 |
| cg14396117 | *MYR8* | 23026 | 0.47 | 0.03683 |
| cg14464791 | *DDX6* | 1656 | 0.14 | 0.03683 |
| cg14782678 | *TMEM102* | 284114 | -0.62 | 0.03683 |
| cg15008991 | *ZNF673* | 55634 | 0.13 | 0.03683 |
| cg15201635 | *SMPD3* | 55512 | 0.10 | 0.03683 |
| cg15319076 | *DCTN6* | 10671 | 0.09 | 0.03683 |
| cg15373285 | *MEG3* | 55384 | -0.64 | 0.03683 |
| cg15769409 | *KIAA0649* | 9858 | 0.10 | 0.03683 |
| cg15786541 | *PVRL3* | 25945 | 0.11 | 0.03683 |
| cg16046951 | *MKKS* | 8195 | 0.16 | 0.03683 |
| cg16054422 | *SART3* | 9733 | 0.11 | 0.03683 |
| cg16484842 | *TH1L* | 51497 | 0.17 | 0.03683 |
| cg16579101 | *NOL1* | 4839 | 0.15 | 0.03683 |
| cg16688376 | *AKAP9* | 10142 | 0.11 | 0.03683 |
| cg16959606 | *ATP1B1* | 481 | 0.11 | 0.03683 |
| cg16986494 | *FHIT* | 2272 | 0.11 | 0.03683 |
| cg16998213 | *METRN* | 79006 | 0.14 | 0.03683 |
| cg17074212 | *ZNF215* | 7762 | 0.11 | 0.03683 |
| cg17108819 | *CD8A* | 925 | 0.12 | 0.03683 |
| cg17150465 | *SCRIB* | 23513 | 0.12 | 0.03683 |
| cg17162024 | *UNQ9433* | 389658 | 0.12 | 0.03683 |
| cg17428950 | *SQRDL* | 58472 | 0.10 | 0.03683 |
| cg17685628 | *NKX2-8* | 26257 | 0.09 | 0.03683 |
| cg17703554 | *WDR21C* | 138009 | -0.33 | 0.03683 |
| cg17815841 | *SCC-112* | 23244 | 0.12 | 0.03683 |
| cg17880199 | *MYH11* | 4629 | 0.09 | 0.03683 |
| cg18123596 | *PIP5K2B* | 8396 | 0.11 | 0.03683 |
| cg18372353 | *ATPBD4* | 89978 | 0.11 | 0.03683 |
| cg18457775 | *ATM* | 472 | 0.11 | 0.03683 |
| cg18576883 | *ZNF639* | 51193 | 0.09 | 0.03683 |
| cg19044630 | *C9orf65* | 158471 | 0.14 | 0.03683 |
| cg19103704 | *FCGBP* | 8857 | 0.34 | 0.03683 |
| cg19192120 | *SSH3* | 54961 | 0.23 | 0.03683 |
| cg19351638 | *AVPR2* | 554 | 0.31 | 0.03683 |
| cg19448829 | *UBR2* | 23304 | 0.14 | 0.03683 |
| cg19514469 | *ELMO3* | 79767 | -0.77 | 0.03683 |
| cg19784449 | *WASF2* | 10163 | 0.20 | 0.03683 |
| cg19891733 | *GABPA* | 2551 | 0.13 | 0.03683 |
| cg19939627 | *MRPS34* | 65993 | 0.10 | 0.03683 |
| cg19961382 | *RANGAP1* | 5905 | 0.11 | 0.03683 |
| cg20030243 | *F2R* | 2149 | 0.10 | 0.03683 |
| cg20379125 | *NOV* | 4856 | 0.13 | 0.03683 |
| cg20492121 | *GLUL* | 2752 | 0.10 | 0.03683 |
| cg20634573 | *ADRA2B* | 151 | -0.62 | 0.03683 |
| cg20663831 | *GIMAP2* | 26157 | 0.38 | 0.03683 |
| cg20678353 | *FLJ35695* | 400359 | 0.47 | 0.03683 |
| cg21172540 | *TSSK3* | 81629 | 0.11 | 0.03683 |
| cg21341807 | *RABL4* | 11020 | 0.09 | 0.03683 |
| cg21351102 | *MRCL3* | 10627 | 0.11 | 0.03683 |
| cg21421501 | *FTH1* | 2495 | 0.12 | 0.03683 |
| cg21429500 | *LAP3* | 51056 | 0.10 | 0.03683 |
| cg21578541 | *TLR9* | 54106 | 0.44 | 0.03683 |
| cg21595737 | *GMFB* | 2764 | 0.15 | 0.03683 |
| cg21675030 | *ABHD10* | 55347 | 0.09 | 0.03683 |
| cg22082243 | *C10orf84* | 63877 | 0.11 | 0.03683 |
| cg22165685 | *VENTX* | 27287 | -0.49 | 0.03683 |
| cg22214414 | *SYCP2* | 10388 | -0.35 | 0.03683 |
| cg22281207 | *MAT2A* | 4144 | 0.10 | 0.03683 |
| cg22609576 | *SPG20* | 23111 | 0.11 | 0.03683 |
| cg22836153 | *LOX* | 4015 | 0.14 | 0.03683 |
| cg23207985 | *ADRBK1* | 156 | 0.11 | 0.03683 |
| cg23350446 | *PDCD2* | 5134 | 0.10 | 0.03683 |
| cg23376526 | *SLC27A3* | 11000 | 0.15 | 0.03683 |
| cg23652939 | *C1orf160* | 84065 | 0.11 | 0.03683 |
| cg23737055 | *ANKRD46* | 157567 | 0.11 | 0.03683 |
| cg23776217 | *C18orf10* | 25941 | 0.10 | 0.03683 |
| cg24166097 | *NPM3* | 10360 | 0.11 | 0.03683 |
| cg24332433 | *SLC39A11* | 201266 | 0.13 | 0.03683 |
| cg24345247 | *HIST1H4A* | 8359 | 0.11 | 0.03683 |
| cg24415468 | *ZNF6* | 7552 | 0.12 | 0.03683 |
| cg24420778 | *PRDX6* | 9588 | 0.10 | 0.03683 |
| cg24576735 | *MARVELD1* | 83742 | 0.11 | 0.03683 |
| cg24792272 | *MCM4* | 4173 | 0.15 | 0.03683 |
| cg24920358 | *PPIE* | 10450 | -0.51 | 0.03683 |
| cg25057743 | *PTHR2* | 5746 | 0.09 | 0.03683 |
| cg25283338 | *PTPN9* | 5780 | 0.11 | 0.03683 |
| cg25294646 | *OBFC1* | 79991 | 0.11 | 0.03683 |
| cg25351036 | *NPAL1* | 152519 | 0.10 | 0.03683 |
| cg25392935 | *VCL* | 7414 | 0.10 | 0.03683 |
| cg25458920 | *EIF2AK2* | 5610 | 0.17 | 0.03683 |
| cg25623459 | *TNNI2* | 7136 | 0.30 | 0.03683 |
| cg25791888 | *HSF2BP* | 11077 | 0.10 | 0.03683 |
| cg25820693 | *SEMA6C* | 10500 | 0.09 | 0.03683 |
| cg25985103 | *MAN2C1* | 4123 | 0.15 | 0.03683 |
| cg25990314 | *GNB1L* | 54584 | 0.11 | 0.03683 |
| cg26162695 | *ELAC2* | 60528 | 0.15 | 0.03683 |
| cg26312150 | *TWIST1* | 7291 | 0.11 | 0.03683 |
| cg26374101 | *ARRDC1* | 92714 | 0.10 | 0.03683 |
| cg26466094 | *AK5* | 26289 | 0.09 | 0.03683 |
| cg26482939 | *GNA15* | 2769 | 0.74 | 0.03683 |
| cg26840318 | *IRF2BP2* | 359948 | 0.10 | 0.03683 |
| cg27270684 | *FKBP9L* | 360132 | 0.38 | 0.03683 |
| cg00755043 | *C9orf121* | 158046 | 0.14 | 0.03683 |
| cg26224139 | *C9orf116* | 138162 | -0.41 | 0.03683 |
| cg05237543 | *MTDH* | 92140 | 0.13 | 0.03684 |
| cg07043494 | *HRH1* | 3269 | -0.15 | 0.03684 |
| cg19716462 | *CCT4* | 10575 | 0.12 | 0.03684 |
| cg26464596 | *RDH10* | 157506 | 0.11 | 0.03684 |
| cg00615377 | *RBM9* | 23543 | 0.37 | 0.03688 |
| cg16225429 | *TUSC2* | 11334 | 0.12 | 0.03688 |
| cg18557086 | *MNT* | 4335 | 0.23 | 0.03688 |
| cg23662675 | *PRKCBP1* | 23613 | 0.11 | 0.03688 |
| cg00443307 | *KLRG1* | 10219 | 0.31 | 0.03689 |
| cg04495969 | *FBXO11* | 80204 | 0.09 | 0.03689 |
| cg09015232 | *SLC12A5* | 57468 | -0.46 | 0.03689 |
| cg06487986 | *PER3* | 8863 | 0.11 | 0.03693 |
| cg25095380 | *PRDM5* | 11107 | 0.11 | 0.03693 |
| cg06730468 | *FLJ10922* | 55260 | 0.14 | 0.03693 |
| cg26330371 | *LMAN1* | 3998 | 0.11 | 0.03693 |
| cg14939691 | *TKT* | 7086 | 0.14 | 0.03695 |
| cg21878918 | *LOC56901* | 56901 | 0.13 | 0.03695 |
| cg22190861 | *GPR155* | 151556 | 0.11 | 0.03695 |
| cg24903376 | *STIL* | 6491 | 0.11 | 0.03695 |
| cg04556854 | *FLJ35630* | 166379 | 0.11 | 0.03697 |
| cg11251086 | *DUSP11* | 8446 | 0.12 | 0.03697 |
| cg12435611 | *BRIP1* | 83990 | 0.14 | 0.03697 |
| cg14859460 | *GRM6* | 2916 | 0.10 | 0.03697 |
| cg16614332 | *SF3B3* | 23450 | 0.10 | 0.03697 |
| cg19311602 | *FLJ10006* | 55677 | 0.11 | 0.03697 |
| cg24443136 | *ATP2B3* | 492 | -0.09 | 0.03697 |
| cg10135717 | *ADAMTS17* | 170691 | -0.65 | 0.03698 |
| cg10758292 | *DEFA1* | 1667 | 0.38 | 0.03699 |
| cg20416767 | *TCEAL8* | 90843 | 0.11 | 0.03700 |
| cg03890877 | *PTK9L* | 11344 | 0.13 | 0.03701 |
| cg00084687 | *GATAD1* | 57798 | 0.09 | 0.03705 |
| cg00179446 | *UPF3B* | 65109 | 0.14 | 0.03705 |
| cg01991383 | *FOXRED1* | 55572 | 0.11 | 0.03705 |
| cg02815516 | *ANKRD25* | 25959 | 0.52 | 0.03705 |
| cg03658471 | *TMEM23* | 259230 | 0.16 | 0.03705 |
| cg03887787 | *CTSW* | 1521 | 0.44 | 0.03705 |
| cg07276902 | *SNRPG* | 6637 | 0.11 | 0.03705 |
| cg08080029 | *CHD5* | 26038 | 0.16 | 0.03705 |
| cg09259332 | *EEA1* | 8411 | 0.10 | 0.03705 |
| cg10274830 | *C6orf96* | 55005 | 0.09 | 0.03705 |
| cg10716163 | *MUM1* | 84939 | 0.14 | 0.03705 |
| cg10935709 | *CBWD1* | 55871 | 0.12 | 0.03705 |
| cg11787839 | *IQCG* | 84223 | 0.10 | 0.03705 |
| cg14028823 | *EEF2K* | 29904 | 0.11 | 0.03705 |
| cg14223017 | *AP3D1* | 8943 | 0.29 | 0.03705 |
| cg17525003 | *IKIP* | 121457 | 0.13 | 0.03705 |
| cg17754980 | *FAM54A* | 113115 | 0.10 | 0.03705 |
| cg17809798 | *SNW1* | 22938 | 0.10 | 0.03705 |
| cg19136717 | *ARIH1* | 25820 | 0.08 | 0.03705 |
| cg21168622 | *ZNF350* | 59348 | 0.12 | 0.03705 |
| cg23539753 | *SP100* | 6672 | 0.40 | 0.03705 |
| cg25622628 | *C19orf24* | 55009 | 0.13 | 0.03705 |
| cg26426582 | *TRIM60* | 166655 | -0.44 | 0.03705 |
| cg27477205 | *MKRN2* | 23609 | 0.14 | 0.03705 |
| cg01313514 | *WNT3A* | 89780 | 0.08 | 0.03706 |
| cg02917381 | *MEIS2* | 4212 | 0.09 | 0.03706 |
| cg06893980 | *PSMB1* | 5689 | 0.12 | 0.03706 |
| cg07011445 | *C10orf86* | 54780 | 0.11 | 0.03706 |
| cg10869376 | *MRPS31* | 10240 | 0.16 | 0.03706 |
| cg12643449 | *IFRD1* | 3475 | 0.27 | 0.03706 |
| cg13056269 | *MCM6* | 4175 | 0.09 | 0.03706 |
| cg19355685 | *ZFAND2B* | 130617 | 0.08 | 0.03706 |
| cg19358493 | *EMX2* | 2018 | 0.13 | 0.03706 |
| cg20375690 | *C1orf126* | 200197 | 0.10 | 0.03706 |
| cg25060573 | *CCND1* | 595 | 0.09 | 0.03706 |
| cg26198776 | *EIF3S5* | 8665 | 0.11 | 0.03706 |
| cg03862540 | *PPP4R1* | 9989 | 0.09 | 0.03707 |
| cg07336230 | *KIF6* | 221458 | 0.10 | 0.03708 |
| cg13946500 | *PPAN* | 56342 | 0.10 | 0.03708 |
| cg17602451 | *BCL2* | 596 | 0.10 | 0.03708 |
| cg25741319 | *FLJ32745* | 165055 | 0.12 | 0.03708 |
| cg22730830 | *PRSS21* | 10942 | -0.62 | 0.03710 |
| cg10019507 | *SSTR4* | 6754 | -0.60 | 0.03711 |
| cg00129774 | *CDK2* | 1017 | 0.13 | 0.03711 |
| cg10787197 | *C6orf105* | 84830 | 0.36 | 0.03713 |
| cg22041658 | *POP4* | 10775 | 0.11 | 0.03714 |
| cg26605086 | *CCNE1* | 898 | 0.14 | 0.03714 |
| cg00618396 | *VBP1* | 7411 | 0.13 | 0.03714 |
| cg01442799 | *YAP1* | 10413 | 0.12 | 0.03714 |
| cg02415431 | *IGLL1* | 3543 | 0.44 | 0.03714 |
| cg03464655 | *FLJ39822* | 151258 | -0.56 | 0.03714 |
| cg16645539 | *SNAPC3* | 6619 | 0.10 | 0.03714 |
| cg17115258 | *TM2D3* | 80213 | 0.09 | 0.03714 |
| cg17239761 | *SNAP29* | 9342 | 0.09 | 0.03714 |
| cg22101147 | *TMEM110* | 375346 | 0.10 | 0.03714 |
| cg22198425 | *TCEB3* | 6924 | 0.13 | 0.03714 |
| cg25179291 | *FNBP1* | 23048 | 0.08 | 0.03714 |
| cg27210136 | *SP6* | 80320 | 0.30 | 0.03714 |
| cg25955969 | *DPH2* | 1802 | 0.10 | 0.03716 |
| cg12646585 | *C8orf32* | 55093 | 0.14 | 0.03718 |
| cg27510871 | *C9orf43* | 257169 | 0.13 | 0.03718 |
| cg01936124 | *ETF1* | 2107 | 0.09 | 0.03722 |
| cg00718748 | *ZNF624* | 57547 | 0.10 | 0.03724 |
| cg01954353 | *MGC7036* | 196383 | 0.14 | 0.03724 |
| cg02066887 | *POLD4* | 57804 | 0.10 | 0.03724 |
| cg02114786 | *PTTG1IP* | 754 | 0.10 | 0.03724 |
| cg02947253 | *ADORA3* | 140 | 0.60 | 0.03724 |
| cg03439703 | *PDE3B* | 5140 | -0.28 | 0.03724 |
| cg04600406 | *UQCR* | 10975 | 0.11 | 0.03724 |
| cg04972979 | *C20orf54* | 113278 | 0.41 | 0.03724 |
| cg06501070 | *EDG7* | 23566 | 0.12 | 0.03724 |
| cg07444907 | *UBLCP1* | 134510 | 0.11 | 0.03724 |
| cg07484450 | *MGC39681* | 283197 | -0.37 | 0.03724 |
| cg07694025 | *SFRP2* | 6423 | 0.15 | 0.03724 |
| cg07871503 | *RASGEF1A* | 221002 | -0.59 | 0.03724 |
| cg07952773 | *ADPRHL2* | 54936 | 0.12 | 0.03724 |
| cg08604885 | *TRIM4* | 89122 | 0.09 | 0.03724 |
| cg09040752 | *AOC3* | 8639 | 0.46 | 0.03724 |
| cg09087901 | *PLG* | 5340 | -0.25 | 0.03724 |
| cg09185773 | *WASF3* | 10810 | 0.17 | 0.03724 |
| cg10196289 | *NSMAF* | 8439 | 0.07 | 0.03724 |
| cg10533434 | *SERPINB3* | 6317 | 0.26 | 0.03724 |
| cg11484288 | *PTPN23* | 25930 | 0.10 | 0.03724 |
| cg11814446 | *LEMD3* | 23592 | 0.11 | 0.03724 |
| cg11815363 | *KCNK1* | 3775 | 0.08 | 0.03724 |
| cg12108912 | *MGC10993* | 80775 | -0.40 | 0.03724 |
| cg12721546 | *BCLAF1* | 9774 | 0.11 | 0.03724 |
| cg12888039 | *ADIPOR1* | 51094 | 0.11 | 0.03724 |
| cg13477510 | *SEPT5* | 5413 | -0.42 | 0.03724 |
| cg13724813 | *SPSB1* | 80176 | 0.09 | 0.03724 |
| cg14430111 | *TM7SF2* | 7108 | 0.10 | 0.03724 |
| cg14714578 | *B3GTL* | 145173 | 0.12 | 0.03724 |
| cg14726525 | *LSM1* | 27257 | 0.13 | 0.03724 |
| cg14913610 | *KLRG1* | 10219 | 0.45 | 0.03724 |
| cg15459773 | *C1orf124* | 83932 | -0.50 | 0.03724 |
| cg15691841 | *ZNF687* | 57592 | 0.09 | 0.03724 |
| cg15820189 | *SAP30BP* | 29115 | 0.11 | 0.03724 |
| cg16281796 | *PEX26* | 55670 | 0.11 | 0.03724 |
| cg16477324 | *ADCK4* | 79934 | 0.11 | 0.03724 |
| cg16925486 | *RPS6* | 6194 | 0.10 | 0.03724 |
| cg17164520 | *CGB1* | 114335 | -0.69 | 0.03724 |
| cg17624892 | *HMGN4* | 10473 | 0.09 | 0.03724 |
| cg18087477 | *SYCP1* | 6847 | -0.42 | 0.03724 |
| cg19304352 | *DEFA4* | 1669 | 0.14 | 0.03724 |
| cg20821095 | *FADD* | 8772 | 0.13 | 0.03724 |
| cg21140292 | *SMARCA2* | 6595 | 0.12 | 0.03724 |
| cg21574752 | *COPG2* | 26958 | 0.09 | 0.03724 |
| cg21665000 | *MYOCD* | 93649 | 0.09 | 0.03724 |
| cg23266266 | *DCUN1D4* | 23142 | 0.10 | 0.03724 |
| cg23337289 | *ZNF513* | 130557 | 0.09 | 0.03724 |
| cg24008892 | *PMF1* | 11243 | -0.61 | 0.03724 |
| cg25999486 | *TBC1D4* | 9882 | 0.13 | 0.03724 |
| cg26196700 | *SORD* | 6652 | 0.10 | 0.03724 |
| cg26365553 | *MADD* | 8567 | 0.10 | 0.03724 |
| cg27494383 | *LTK* | 4058 | 0.12 | 0.03724 |
| cg27529628 | *GAS2L3* | 283431 | 0.09 | 0.03724 |
| cg05982504 | *IGFALS* | 3483 | -0.11 | 0.03725 |
| cg20147100 | *C19orf6* | 91304 | 0.13 | 0.03727 |
| cg01570769 | *PNRC1* | 10957 | 0.09 | 0.03731 |
| cg06754047 | *SMYD5* | 10322 | 0.09 | 0.03731 |
| cg08186362 | *HRH3* | 11255 | 0.11 | 0.03731 |
| cg08727202 | *MPST* | 4357 | -0.61 | 0.03731 |
| cg16761581 | *ADCY4* | 196883 | 0.08 | 0.03731 |
| cg17974185 | *CTNNBL1* | 56259 | -0.62 | 0.03731 |
| cg22176097 | *MGC23280* | 147015 | 0.11 | 0.03731 |
| cg24871743 | *DIRAS3* | 9077 | -0.25 | 0.03731 |
| cg26332534 | *HTR7* | 3363 | 0.09 | 0.03731 |
| cg20131596 | *PAM* | 5066 | -0.48 | 0.03731 |
| cg00076645 | *DC-UbP* | 92181 | 0.11 | 0.03735 |
| cg00290506 | *CNIH3* | 149111 | 0.08 | 0.03735 |
| cg00522034 | *CBLB* | 868 | 0.09 | 0.03735 |
| cg00793648 | *DDX24* | 57062 | -0.30 | 0.03735 |
| cg01328164 | *HINT2* | 84681 | 0.11 | 0.03735 |
| cg01664666 | *PLD5* | 200150 | 0.12 | 0.03735 |
| cg01836044 | *PCDH20* | 64881 | 0.14 | 0.03735 |
| cg02226939 | *BLMH* | 642 | 0.09 | 0.03735 |
| cg02292024 | *TIMM10* | 26519 | 0.12 | 0.03735 |
| cg02801598 | *PDP2* | 57546 | 0.12 | 0.03735 |
| cg02904365 | *DNAJC11* | 55735 | 0.10 | 0.03735 |
| cg03475172 | *USP6NL* | 9712 | 0.12 | 0.03735 |
| cg03506799 | *CHSY1* | 22856 | 0.12 | 0.03735 |
| cg03812679 | *CSF3* | 1440 | 0.37 | 0.03735 |
| cg03862760 | *MGC10911* | 84262 | 0.14 | 0.03735 |
| cg04261665 | *C12orf52* | 84934 | 0.15 | 0.03735 |
| cg04310824 | *SCP2* | 6342 | 0.09 | 0.03735 |
| cg04330449 | *NEUROG1* | 4762 | 0.12 | 0.03735 |
| cg04410862 | *E2F7* | 144455 | 0.10 | 0.03735 |
| cg04648087 | *TSC1* | 7248 | 0.11 | 0.03735 |
| cg05119831 | *PTPRN2* | 5799 | 0.13 | 0.03735 |
| cg06243556 | *ZNF447* | 65982 | 0.10 | 0.03735 |
| cg06295404 | *SOCS1* | 8651 | 0.16 | 0.03735 |
| cg06382698 | *ASB3* | 51130 | 0.20 | 0.03735 |
| cg06740897 | *NFE2L1* | 4779 | 0.16 | 0.03735 |
| cg06818415 | *PHF1* | 5252 | 0.12 | 0.03735 |
| cg07181349 | *KIT* | 3815 | 0.13 | 0.03735 |
| cg07569069 | *FLJ35119* | 126074 | 0.11 | 0.03735 |
| cg07864189 | *NADSYN1* | 55191 | 0.12 | 0.03735 |
| cg07955356 | *DNASE2* | 1777 | 0.11 | 0.03735 |
| cg08798116 | *GPC4* | 2239 | 0.10 | 0.03735 |
| cg08946117 | *MGC50273* | 408029 | -0.54 | 0.03735 |
| cg09067967 | *UGDH* | 7358 | 0.15 | 0.03735 |
| cg09879099 | *ONECUT2* | 9480 | 0.09 | 0.03735 |
| cg10074513 | *FAM79A* | 127262 | 0.14 | 0.03735 |
| cg10164137 | *DVL1* | 1855 | 0.11 | 0.03735 |
| cg10257049 | *C5orf4* | 10826 | 0.37 | 0.03735 |
| cg10398950 | *CRKRS* | 51755 | 0.10 | 0.03735 |
| cg10526888 | *DKFZp564K142* | 84061 | 0.13 | 0.03735 |
| cg11128808 | *CACNG6* | 59285 | -0.68 | 0.03735 |
| cg11601662 | *FKBP14* | 55033 | 0.15 | 0.03735 |
| cg12063359 | *KCNK15* | 60598 | 0.11 | 0.03735 |
| cg12681402 | *RPH3A* | 22895 | 0.12 | 0.03735 |
| cg12707233 | *WNK3* | 65267 | 0.14 | 0.03735 |
| cg12733430 | *KLHL8* | 57563 | 0.10 | 0.03735 |
| cg13802966 | *CASP1* | 834 | 0.31 | 0.03735 |
| cg14030359 | *NDRG2* | 57447 | 0.10 | 0.03735 |
| cg14105047 | *PDLIM5* | 10611 | 0.12 | 0.03735 |
| cg14135551 | *DAAM2* | 23500 | 0.11 | 0.03735 |
| cg14198172 | *HBXIP* | 10542 | 0.17 | 0.03735 |
| cg14425294 | *GUCY2D* | 3000 | 0.09 | 0.03735 |
| cg14717170 | *SLITRK3* | 22865 | 0.11 | 0.03735 |
| cg15247144 | *PYCARD* | 29108 | 0.10 | 0.03735 |
| cg15814508 | *P2RY14* | 9934 | 0.40 | 0.03735 |
| cg15827295 | *LYSMD1* | 388695 | 0.12 | 0.03735 |
| cg15979098 | *ZNF615* | 284370 | 0.10 | 0.03735 |
| cg16142218 | *CHMP7* | 91782 | 0.53 | 0.03735 |
| cg16249454 | *CXCL2* | 2920 | 0.11 | 0.03735 |
| cg16261965 | *ARF3* | 377 | 0.17 | 0.03735 |
| cg16547529 | *FLJ33790* | 283212 | -0.30 | 0.03735 |
| cg16743289 | *CACYBP* | 27101 | -0.44 | 0.03735 |
| cg17429424 | *DUSP1* | 1843 | 0.12 | 0.03735 |
| cg17559549 | *MAPK7* | 5598 | 0.13 | 0.03735 |
| cg17644867 | *FOS* | 2353 | 0.11 | 0.03735 |
| cg17747005 | *DEPDC2* | 80243 | 0.14 | 0.03735 |
| cg17860186 | *PLEKHG2* | 64857 | 0.10 | 0.03735 |
| cg18216709 | *RNF138* | 51444 | 0.12 | 0.03735 |
| cg18364820 | *CXorf38* | 159013 | 0.12 | 0.03735 |
| cg18453621 | *LMX1B* | 4010 | 0.14 | 0.03735 |
| cg18732541 | *MCEMP1* | 199675 | 0.38 | 0.03735 |
| cg18936744 | *AK7* | 122481 | 0.11 | 0.03735 |
| cg18958584 | *SHMT1* | 6470 | 0.11 | 0.03735 |
| cg19219437 | *PCOLCE2* | 26577 | 0.11 | 0.03735 |
| cg19219503 | *ANKRD30A* | 91074 | -0.44 | 0.03735 |
| cg19258973 | *KRTHB3* | 3889 | -0.63 | 0.03735 |
| cg19649173 | *ZNF446* | 55663 | 0.16 | 0.03735 |
| cg19771541 | *MID2* | 11043 | 0.13 | 0.03735 |
| cg19914751 | *QARS* | 5859 | 0.13 | 0.03735 |
| cg19966618 | *ACAD8* | 27034 | 0.15 | 0.03735 |
| cg20088535 | *PLK4* | 10733 | 0.08 | 0.03735 |
| cg20311501 | *APC* | 324 | 0.09 | 0.03735 |
| cg20312228 | *CCDC37* | 348807 | 0.11 | 0.03735 |
| cg20320468 | *LAIR1* | 3903 | 0.32 | 0.03735 |
| cg20451680 | *ESM1* | 11082 | -0.49 | 0.03735 |
| cg20472433 | *TKT* | 7086 | 0.12 | 0.03735 |
| cg20530223 | *KLHL5* | 51088 | 0.14 | 0.03735 |
| cg20583316 | *SERPINE1* | 5054 | 0.42 | 0.03735 |
| cg21004129 | *IL8RA* | 3577 | 0.25 | 0.03735 |
| cg21420531 | *SLC5A6* | 8884 | 0.13 | 0.03735 |
| cg21458907 | *CADPS* | 8618 | 0.11 | 0.03735 |
| cg21762589 | *BNIP3* | 664 | -0.26 | 0.03735 |
| cg21858376 | *ITPR1* | 3708 | 0.10 | 0.03735 |
| cg21950493 | *ATPBD1C* | 51184 | 0.12 | 0.03735 |
| cg21958034 | *MST1* | 4485 | -0.18 | 0.03735 |
| cg22085335 | *KCNF1* | 3754 | 0.11 | 0.03735 |
| cg22451189 | *MRPS22* | 56945 | 0.12 | 0.03735 |
| cg22560190 | *CNTN1* | 1272 | 0.12 | 0.03735 |
| cg22605063 | *DBN1* | 1627 | 0.11 | 0.03735 |
| cg22609068 | *MGC3020* | 79014 | 0.11 | 0.03735 |
| cg22799917 | *YWHAB* | 7529 | 0.13 | 0.03735 |
| cg22927153 | *MAP2K5* | 5607 | 0.14 | 0.03735 |
| cg22932215 | *COPS7B* | 64708 | 0.13 | 0.03735 |
| cg23543604 | *TAF12* | 6883 | 0.10 | 0.03735 |
| cg23644213 | *PIGO* | 84720 | 0.24 | 0.03735 |
| cg23808301 | *PLD2* | 5338 | 0.12 | 0.03735 |
| cg23906144 | *OVCA2* | 124641 | 0.16 | 0.03735 |
| cg23978557 | *SMPD2* | 6610 | -0.51 | 0.03735 |
| cg25413757 | *C1orf75* | 55248 | 0.10 | 0.03735 |
| cg25527547 | *PLOD3* | 8985 | -0.46 | 0.03735 |
| cg25671438 | *CENTB1* | 9744 | 0.41 | 0.03735 |
| cg25999867 | *LOC112937* | 112937 | 0.10 | 0.03735 |
| cg26082838 | *FLRT3* | 23767 | 0.44 | 0.03735 |
| cg26675077 | *TUBB8* | 347688 | -0.37 | 0.03735 |
| cg27537453 | *KCTD5* | 54442 | 0.13 | 0.03735 |
| cg27610561 | *SLC2A10* | 81031 | -0.42 | 0.03735 |
| cg01252496 | *MDS032* | 55850 | -0.72 | 0.03736 |
| cg19221959 | *PPP1R9A* | 55607 | 0.13 | 0.03736 |
| cg21484964 | *C15orf24* | 56851 | -0.36 | 0.03736 |
| cg02126753 | *AEBP1* | 165 | 0.13 | 0.03736 |
| cg04755933 | *AUP1* | 550 | 0.13 | 0.03736 |
| cg04891836 | *TNFSF14* | 8740 | 0.21 | 0.03736 |
| cg05029288 | *UGDH* | 7358 | 0.12 | 0.03736 |
| cg05275231 | *SLC25A32* | 81034 | 0.14 | 0.03736 |
| cg05777319 | *ARL6* | 84100 | 0.10 | 0.03736 |
| cg06906435 | *FLJ25773* | 283598 | 0.28 | 0.03736 |
| cg07080946 | *LUC7L* | 55692 | -0.25 | 0.03736 |
| cg08704884 | *UBE2M* | 9040 | 0.10 | 0.03736 |
| cg09472203 | *AP3B2* | 8120 | 0.13 | 0.03736 |
| cg09606564 | *MFAP4* | 4239 | 0.44 | 0.03736 |
| cg11278262 | *KRTCAP2* | 200185 | 0.13 | 0.03736 |
| cg11504897 | *NRN1* | 51299 | 0.21 | 0.03736 |
| cg13728650 | *GPR175* | 131601 | -0.11 | 0.03736 |
| cg20627106 | *PDE6G* | 5148 | -0.09 | 0.03736 |
| cg20757526 | *DCXR* | 51181 | 0.10 | 0.03736 |
| cg21242004 | *VTI1A* | 143187 | -0.40 | 0.03736 |
| cg21491028 | *PSMD3* | 5709 | 0.11 | 0.03736 |
| cg23674882 | *LOXL3* | 84695 | -0.32 | 0.03736 |
| cg26052367 | *GANC* | 2595 | 0.11 | 0.03736 |
| cg18533225 | *KLHDC7B* | 113730 | -0.58 | 0.03736 |
| cg01466075 | *SERP1* | 27230 | 0.12 | 0.03737 |
| cg02047577 | *UCKL1* | 54963 | 0.12 | 0.03737 |
| cg04217178 | *ITM2B* | 9445 | 0.12 | 0.03737 |
| cg04915566 | *RUNX1* | 861 | 0.41 | 0.03737 |
| cg04964702 | *CBL* | 867 | 0.13 | 0.03737 |
| cg05037688 | *EGFL7* | 51162 | 0.44 | 0.03737 |
| cg06023257 | *GAB2* | 9846 | 0.12 | 0.03737 |
| cg06175025 | *SPTLC2* | 9517 | 0.08 | 0.03737 |
| cg06834983 | *SSX7* | 280658 | -0.50 | 0.03737 |
| cg07672479 | *DYNC1H1* | 1778 | 0.12 | 0.03737 |
| cg07985359 | *EVI5L* | 115704 | 0.13 | 0.03737 |
| cg09721047 | *ARHGAP25* | 9938 | 0.47 | 0.03737 |
| cg11579069 | *MYB* | 4602 | 0.15 | 0.03737 |
| cg11693019 | *AKR1B10* | 57016 | -0.20 | 0.03737 |
| cg12940430 | *SRF* | 6722 | 0.16 | 0.03737 |
| cg14360917 | *SP2* | 6668 | 0.25 | 0.03737 |
| cg16269097 | *CASK* | 8573 | 0.14 | 0.03737 |
| cg16544272 | *PIGW* | 284098 | 0.11 | 0.03737 |
| cg16601861 | *ALDH1A1* | 216 | 0.48 | 0.03737 |
| cg16771596 | *C9orf30* | 91283 | 0.13 | 0.03737 |
| cg18491230 | *ANLN* | 54443 | 0.07 | 0.03737 |
| cg20684399 | *PHF2* | 5253 | 0.08 | 0.03737 |
| cg22759185 | *REEP6* | 92840 | -0.73 | 0.03737 |
| cg24735937 | *CLTB* | 1212 | -0.57 | 0.03737 |
| cg25869832 | *RPS4X* | 6191 | 0.18 | 0.03737 |
| cg25870420 | *ITGA9* | 3680 | 0.13 | 0.03737 |
| cg26403198 | *FBXL6* | 26233 | 0.13 | 0.03737 |
| cg02727423 | *EML1* | 2009 | -0.42 | 0.03737 |
| cg16584172 | *ZIC2* | 7546 | 0.12 | 0.03737 |
| cg19756068 | *CYP2B6* | 1555 | 0.42 | 0.03739 |
| cg04572898 | *ISG20L1* | 64782 | 0.09 | 0.03739 |
| cg07301004 | *ARPC3* | 10094 | 0.13 | 0.03739 |
| cg09391966 | *GIT2* | 9815 | 0.10 | 0.03739 |
| cg18337803 | *CACNA1G* | 8913 | 0.15 | 0.03739 |
| cg19794490 | *NCOA6* | 23054 | 0.16 | 0.03739 |
| cg20828084 | *KIAA1199* | 57214 | -0.44 | 0.03739 |
| cg24335149 | *PLA2G5* | 5322 | 0.10 | 0.03739 |
| cg10995380 | *TGFB1I1* | 7041 | -0.42 | 0.03742 |
| cg26303908 | *GNPDA2* | 132789 | 0.10 | 0.03742 |
| cg00566759 | *DSC2* | 1824 | 0.10 | 0.03745 |
| cg27342122 | *PHF20L1* | 51105 | 0.18 | 0.03747 |
| cg00472814 | *ADAMTS1* | 9510 | 0.11 | 0.03748 |
| cg00156216 | *APLP2* | 334 | 0.14 | 0.03749 |
| cg00382515 | *MRPL44* | 65080 | 0.10 | 0.03749 |
| cg00620629 | *C6orf113* | 221302 | 0.07 | 0.03749 |
| cg01424107 | *CDX2* | 1045 | 0.11 | 0.03749 |
| cg02237186 | *RRM2* | 6241 | 0.13 | 0.03749 |
| cg02635407 | *SH3TC1* | 54436 | 0.36 | 0.03749 |
| cg02648999 | *TGFBRAP1* | 9392 | 0.13 | 0.03749 |
| cg02945646 | *AP1G2* | 8906 | 0.08 | 0.03749 |
| cg03285457 | *LBX1* | 10660 | 0.13 | 0.03749 |
| cg03455874 | *BRI3BP* | 140707 | 0.12 | 0.03749 |
| cg03962522 | *SLC5A1* | 6523 | 0.61 | 0.03749 |
| cg04145477 | *QRSL1* | 55278 | 0.10 | 0.03749 |
| cg04567315 | *CLUL1* | 27098 | 0.16 | 0.03749 |
| cg04613791 | *FKBP1A* | 2280 | 0.07 | 0.03749 |
| cg04741821 | *TXNDC13* | 56255 | 0.13 | 0.03749 |
| cg05376954 | *FPR1* | 2357 | 0.20 | 0.03749 |
| cg05501357 | *HIPK3* | 10114 | 0.35 | 0.03749 |
| cg05715287 | *CDC40* | 51362 | 0.10 | 0.03749 |
| cg05886626 | *THBS1* | 7057 | 0.18 | 0.03749 |
| cg05905876 | *SEC11L1* | 23478 | 0.11 | 0.03749 |
| cg05959508 | *TRAF3IP3* | 80342 | 0.41 | 0.03749 |
| cg06191076 | *DIRAS3* | 9077 | -0.47 | 0.03749 |
| cg06369854 | *VDR* | 7421 | 0.08 | 0.03749 |
| cg06498267 | *HCN1* | 348980 | 0.07 | 0.03749 |
| cg07290435 | *CDH10* | 1008 | 0.17 | 0.03749 |
| cg07310951 | *ZIM2* | 23619 | -0.88 | 0.03749 |
| cg07697569 | *H2AFX* | 3014 | 0.11 | 0.03749 |
| cg07775501 | *JOSD2* | 126119 | 0.12 | 0.03749 |
| cg08271519 | *TM9SF2* | 9375 | 0.09 | 0.03749 |
| cg08426384 | *ADCK1* | 57143 | 0.12 | 0.03749 |
| cg08596544 | *SLC33A1* | 9197 | 0.15 | 0.03749 |
| cg08703595 | *FLJ22313* | 64224 | 0.11 | 0.03749 |
| cg09218130 | *PUS1* | 80324 | 0.10 | 0.03749 |
| cg09622447 | *CBS* | 875 | 0.10 | 0.03749 |
| cg09944147 | *COL27A1* | 85301 | 0.12 | 0.03749 |
| cg12506971 | *AADAT* | 51166 | 0.12 | 0.03749 |
| cg12513308 | *CKLF* | 51192 | 0.10 | 0.03749 |
| cg13590277 | *SYNPO* | 11346 | 0.27 | 0.03749 |
| cg13713381 | *TMEM44* | 93109 | 0.09 | 0.03749 |
| cg14545899 | *FAM46C* | 54855 | 0.12 | 0.03749 |
| cg15318532 | *RFXAP* | 5994 | 0.11 | 0.03749 |
| cg15587603 | *RPS27A* | 6233 | 0.11 | 0.03749 |
| cg16179125 | *CTSZ* | 1522 | 0.14 | 0.03749 |
| cg16200844 | *ITCH* | 83737 | 0.11 | 0.03749 |
| cg16275172 | *TMOD3* | 29766 | 0.11 | 0.03749 |
| cg16442715 | *MAP3K7IP1* | 10454 | 0.07 | 0.03749 |
| cg16504670 | *FLJ20186* | 54849 | -0.48 | 0.03749 |
| cg16545079 | *PER1* | 5187 | 0.12 | 0.03749 |
| cg16632280 | *LTBP3* | 4054 | 0.10 | 0.03749 |
| cg16728114 | *C1orf66* | 51093 | 0.14 | 0.03749 |
| cg17271365 | *MARCH1* | 55016 | 0.36 | 0.03749 |
| cg17332016 | *FLJ10847* | 55244 | 0.12 | 0.03749 |
| cg17404605 | *MAP2K7* | 5609 | 0.12 | 0.03749 |
| cg17589175 | *HSPC152* | 51504 | 0.11 | 0.03749 |
| cg17605084 | *HEM1* | 3071 | 0.41 | 0.03749 |
| cg18565355 | *RBM35A* | 54845 | -0.52 | 0.03749 |
| cg18579327 | *CREB1* | 1385 | 0.12 | 0.03749 |
| cg18865999 | *PCYOX1* | 51449 | 0.09 | 0.03749 |
| cg19273182 | *PAPOLG* | 64895 | 0.10 | 0.03749 |
| cg19545851 | *NUDCD2* | 134492 | 0.07 | 0.03749 |
| cg19783114 | *TP73* | 7161 | 0.11 | 0.03749 |
| cg20716064 | *USP6NL* | 9712 | 0.11 | 0.03749 |
| cg20769102 | *POLR2K* | 5440 | 0.10 | 0.03749 |
| cg21085553 | *PARP8* | 79668 | 0.15 | 0.03749 |
| cg21270593 | *PEG10* | 23089 | -0.61 | 0.03749 |
| cg21573601 | *PTEN* | 5728 | 0.08 | 0.03749 |
| cg21772838 | *CSTF1* | 1477 | 0.09 | 0.03749 |
| cg21774863 | *RAB28* | 9364 | 0.10 | 0.03749 |
| cg21844956 | *TRIM38* | 10475 | 0.50 | 0.03749 |
| cg22148652 | *ARL5* | 26225 | 0.09 | 0.03749 |
| cg22244122 | *SURF4* | 6836 | -0.44 | 0.03749 |
| cg22289115 | *MUCDHL* | 53841 | -0.11 | 0.03749 |
| cg22362636 | *TNIP2* | 79155 | 0.09 | 0.03749 |
| cg22557067 | *NISCH* | 11188 | 0.13 | 0.03749 |
| cg22792910 | *ANXA4* | 307 | 0.33 | 0.03749 |
| cg23239842 | *NCOA2* | 10499 | 0.10 | 0.03749 |
| cg23326785 | *TMEM25* | 84866 | 0.12 | 0.03749 |
| cg23571857 | *BIRC4BP* | 54739 | 0.30 | 0.03749 |
| cg23970523 | *FKBP8* | 23770 | 0.14 | 0.03749 |
| cg24012887 | *BTBD14B* | 112939 | 0.14 | 0.03749 |
| cg24165720 | *NACAL* | 342538 | -0.48 | 0.03749 |
| cg24343515 | *PI4KII* | 55361 | 0.13 | 0.03749 |
| cg24555656 | *TAF6* | 6878 | 0.12 | 0.03749 |
| cg24957950 | *PAK4* | 10298 | 0.12 | 0.03749 |
| cg25459778 | *CRAMP1L* | 57585 | 0.11 | 0.03749 |
| cg26191951 | *RNASE2* | 6036 | 0.42 | 0.03749 |
| cg26276530 | *CMAS* | 55907 | 0.10 | 0.03749 |
| cg26534002 | *CSAG3A* | 389903 | -0.50 | 0.03749 |
| cg26847866 | *SCARA3* | 51435 | -0.39 | 0.03749 |
| cg13821008 | *WASPIP* | 7456 | 0.35 | 0.03750 |
| cg08662074 | *PAPPA* | 5069 | 0.13 | 0.03751 |
| cg02668729 | *CTPS* | 1503 | 0.13 | 0.03752 |
| cg06186808 | *LOC161247* | 161247 | -0.10 | 0.03752 |
| cg09874127 | *UBE1L* | 7318 | 0.37 | 0.03752 |
| cg10391629 | *AKAP11* | 11215 | 0.11 | 0.03752 |
| cg10894102 | *CLIC4* | 25932 | 0.16 | 0.03752 |
| cg11207564 | *CCL3L3* | 414062 | 0.40 | 0.03752 |
| cg11326640 | *STAU* | 6780 | 0.14 | 0.03752 |
| cg11595749 | *ATP5E* | 514 | 0.10 | 0.03752 |
| cg13113656 | *CLTC* | 1213 | 0.13 | 0.03752 |
| cg13236637 | *SURB7* | 9412 | 0.11 | 0.03752 |
| cg13240598 | *FLVCR* | 28982 | 0.10 | 0.03752 |
| cg14462626 | *MRLC2* | 103910 | 0.13 | 0.03752 |
| cg14659404 | *ASTN* | 460 | 0.16 | 0.03752 |
| cg15309223 | *C1orf83* | 127428 | 0.09 | 0.03752 |
| cg15840658 | *APBA1* | 320 | 0.09 | 0.03752 |
| cg16543027 | *PLCB2* | 5330 | 0.38 | 0.03752 |
| cg16551326 | *ERF* | 2077 | 0.11 | 0.03752 |
| cg19132701 | *CHMP6* | 79643 | 0.11 | 0.03752 |
| cg20717059 | *C8orf13* | 83648 | 0.09 | 0.03752 |
| cg20770311 | *SMPD1* | 6609 | 0.11 | 0.03752 |
| cg21222430 | *CDKN1A* | 1026 | 0.17 | 0.03752 |
| cg21421701 | *SNFT* | 55509 | 0.13 | 0.03752 |
| cg22411068 | *ROPN1L* | 83853 | 0.10 | 0.03752 |
| cg23513644 | *MPHOSPH10* | 10199 | 0.09 | 0.03752 |
| cg23606023 | *CBFA2T3* | 863 | 0.35 | 0.03752 |
| cg23752923 | *MCOLN3* | 55283 | 0.13 | 0.03752 |
| cg24334790 | *ARL5* | 26225 | 0.10 | 0.03752 |
| cg25945303 | *KLF6* | 1316 | 0.09 | 0.03752 |
| cg27074297 | *SOS1* | 6654 | 0.11 | 0.03752 |
| cg27470554 | *FCGR2A* | 2212 | 0.67 | 0.03753 |
| cg10316635 | *INADL* | 10207 | 0.13 | 0.03757 |
| cg26260350 | *OTX1* | 5013 | 0.15 | 0.03760 |
| cg01056568 | *GLP2R* | 9340 | 0.20 | 0.03763 |
| cg04507972 | *CRSP2* | 9282 | 0.12 | 0.03763 |
| cg11422541 | *ADCY6* | 112 | 0.11 | 0.03763 |
| cg16882703 | *PSMB6* | 5694 | 0.11 | 0.03763 |
| cg20540913 | *EDA* | 1896 | 0.11 | 0.03763 |
| cg25070480 | *PPHLN1* | 51535 | 0.16 | 0.03763 |
| cg26421123 | *COMMD5* | 28991 | 0.14 | 0.03765 |
| cg26507094 | *PYCRL* | 65263 | 0.11 | 0.03765 |
| cg09618682 | *SEC22L2* | 26984 | 0.09 | 0.03765 |
| cg14754581 | *CCRL2* | 9034 | 0.48 | 0.03765 |
| cg15946545 | *MRPS31* | 10240 | 0.11 | 0.03765 |
| cg24073161 | *ZNF403* | 79893 | 0.10 | 0.03765 |
| cg25867173 | *UGT2B17* | 7367 | -0.21 | 0.03765 |
| cg26155617 | *DDO* | 8528 | -0.13 | 0.03765 |
| cg04367351 | *PLA2R1* | 22925 | 0.12 | 0.03766 |
| cg12380764 | *IL19* | 29949 | 0.46 | 0.03766 |
| cg26366091 | *CHI3L2* | 1117 | 0.46 | 0.03766 |
| cg26488636 | *TALDO1* | 6888 | 0.10 | 0.03766 |
| cg00184884 | *SFI1* | 9814 | 0.09 | 0.03767 |
| cg00350478 | *FRMD1* | 79981 | 0.47 | 0.03767 |
| cg00402366 | *LRRC44* | 127255 | 0.12 | 0.03767 |
| cg00616369 | *NCL* | 4691 | 0.12 | 0.03767 |
| cg00671161 | *ATP7B* | 540 | 0.10 | 0.03767 |
| cg01151686 | *LIG3* | 3980 | 0.12 | 0.03767 |
| cg01197831 | *FBP2* | 8789 | -0.10 | 0.03767 |
| cg01206970 | *PPFIA3* | 8541 | -0.75 | 0.03767 |
| cg01361446 | *IL2RG* | 3561 | 0.43 | 0.03767 |
| cg01513661 | *SAPS2* | 9701 | 0.12 | 0.03767 |
| cg01753595 | *COL5A1* | 1289 | 0.11 | 0.03767 |
| cg02144933 | *AOX1* | 316 | 0.09 | 0.03767 |
| cg02479196 | *MARCH7* | 64844 | 0.11 | 0.03767 |
| cg02741744 | *ACTR6* | 64431 | 0.12 | 0.03767 |
| cg02942159 | *RPS6* | 6194 | 0.09 | 0.03767 |
| cg02987051 | *IER3IP1* | 51124 | 0.13 | 0.03767 |
| cg03188118 | *RGS6* | 9628 | 0.16 | 0.03767 |
| cg03240509 | *KCNA6* | 3742 | -0.40 | 0.03767 |
| cg03600318 | *SFTPD* | 6441 | 0.46 | 0.03767 |
| cg04733989 | *NAGA* | 4668 | 0.11 | 0.03767 |
| cg04774694 | *DPEP2* | 64174 | -0.52 | 0.03767 |
| cg04912050 | *IDUA* | 3425 | 0.12 | 0.03767 |
| cg05135288 | *RHOT2* | 89941 | -0.65 | 0.03767 |
| cg06172871 | *HP* | 3240 | 0.35 | 0.03767 |
| cg06417225 | *PEBP1* | 5037 | 0.14 | 0.03767 |
| cg06433995 | *NOL7* | 51406 | 0.08 | 0.03767 |
| cg06449653 | *HNRPR* | 10236 | 0.10 | 0.03767 |
| cg06990379 | *DPYD* | 1806 | 0.12 | 0.03767 |
| cg07154408 | *PDE5A* | 8654 | 0.17 | 0.03767 |
| cg07288394 | *C19orf12* | 83636 | 0.09 | 0.03767 |
| cg07588664 | *PPHLN1* | 51535 | 0.13 | 0.03767 |
| cg07730301 | *ALDH3B1* | 221 | 0.38 | 0.03767 |
| cg07772309 | *NELF* | 26012 | 0.09 | 0.03767 |
| cg08157292 | *PPP1R7* | 5510 | -0.44 | 0.03767 |
| cg08428707 | *CHMP4B* | 128866 | 0.12 | 0.03767 |
| cg08752459 | *CLEC2B* | 9976 | 0.30 | 0.03767 |
| cg08942800 | *CRISP2* | 7180 | -0.33 | 0.03767 |
| cg09056317 | *POLR2I* | 5438 | -0.53 | 0.03767 |
| cg09811393 | *SEMA3B* | 7869 | -0.19 | 0.03767 |
| cg10125119 | *CSDE1* | 7812 | 0.13 | 0.03767 |
| cg10184886 | *PLA2G6* | 8398 | 0.17 | 0.03767 |
| cg10579246 | *MGC15763* | 92106 | -0.30 | 0.03767 |
| cg10615091 | *FAM13C1* | 220965 | 0.10 | 0.03767 |
| cg11044163 | *CPVL* | 54504 | 0.08 | 0.03767 |
| cg11140307 | *CMTM4* | 146223 | 0.11 | 0.03767 |
| cg11519508 | *TP53* | 7157 | 0.13 | 0.03767 |
| cg11611854 | *RPL17* | 6139 | 0.12 | 0.03767 |
| cg11612345 | *SMOC2* | 64094 | 0.11 | 0.03767 |
| cg11654620 | *ECGF1* | 1890 | 0.11 | 0.03767 |
| cg11754343 | *NUP88* | 4927 | 0.09 | 0.03767 |
| cg11932564 | *TNFRSF13C* | 115650 | 0.09 | 0.03767 |
| cg12969595 | *TMEM98* | 26022 | 0.10 | 0.03767 |
| cg13114389 | *ENTPD7* | 57089 | 0.11 | 0.03767 |
| cg13885201 | *ZNF23* | 7571 | 0.09 | 0.03767 |
| cg14014225 | *SLC16A8* | 23539 | -0.61 | 0.03767 |
| cg14203179 | *GNAS* | 2778 | -0.49 | 0.03767 |
| cg14307212 | *SLC12A6* | 9990 | 0.38 | 0.03767 |
| cg14310034 | *BMP4* | 652 | 0.15 | 0.03767 |
| cg14407437 | *FABP3* | 2170 | 0.08 | 0.03767 |
| cg14889768 | *RAB32* | 10981 | 0.10 | 0.03767 |
| cg15375239 | *SPINT2* | 10653 | 0.10 | 0.03767 |
| cg15415507 | *EPHA2* | 1969 | 0.16 | 0.03767 |
| cg15451100 | *PSMB8* | 5696 | 0.11 | 0.03767 |
| cg15609321 | *ZNF512* | 84450 | 0.13 | 0.03767 |
| cg15801967 | *NHP2L1* | 4809 | 0.10 | 0.03767 |
| cg16222698 | *REXO2* | 25996 | 0.08 | 0.03767 |
| cg16246410 | *NR4A2* | 4929 | 0.10 | 0.03767 |
| cg16307860 | *C1orf80* | 64853 | 0.11 | 0.03767 |
| cg16377880 | *CYP4F3* | 4051 | 0.17 | 0.03767 |
| cg16456919 | *LRRN5* | 10446 | -0.44 | 0.03767 |
| cg16512727 | *C12orf4* | 57102 | -0.69 | 0.03767 |
| cg16891895 | *RGS16* | 6004 | 0.14 | 0.03767 |
| cg17241657 | *C4orf16* | 55435 | 0.14 | 0.03767 |
| cg17468997 | *NCF1* | 4687 | 0.44 | 0.03767 |
| cg17771854 | *IPO13* | 9670 | 0.11 | 0.03767 |
| cg17969298 | *ANGEL2* | 90806 | 0.09 | 0.03767 |
| cg18247638 | *BIVM* | 54841 | 0.09 | 0.03767 |
| cg18534872 | *ZWINT* | 11130 | 0.10 | 0.03767 |
| cg19401923 | *MRPL19* | 9801 | 0.12 | 0.03767 |
| cg20028291 | *BCAS4* | 55653 | 0.17 | 0.03767 |
| cg20275133 | *MRPL20* | 55052 | 0.12 | 0.03767 |
| cg20519035 | *FLJ46072* | 286077 | -0.49 | 0.03767 |
| cg20525378 | *C1orf9* | 51430 | 0.18 | 0.03767 |
| cg20580177 | *LRFN3* | 79414 | -0.55 | 0.03767 |
| cg20669525 | *GPAA1* | 8733 | 0.09 | 0.03767 |
| cg20916483 | *C11orf56* | 84067 | 0.10 | 0.03767 |
| cg21224759 | *PIN4* | 5303 | 0.12 | 0.03767 |
| cg21250978 | *PRKAR2B* | 5577 | -0.53 | 0.03767 |
| cg21540749 | *MOCS2* | 4338 | 0.13 | 0.03767 |
| cg21576698 | *UQCRH* | 7388 | 0.24 | 0.03767 |
| cg21750545 | *DCUN1D4* | 23142 | 0.14 | 0.03767 |
| cg21830413 | *VPS26* | 9559 | 0.11 | 0.03767 |
| cg21934269 | *RPL39* | 6170 | 0.11 | 0.03767 |
| cg22037648 | *SEMA3B* | 7869 | 0.11 | 0.03767 |
| cg22178085 | *LKAP* | 9665 | 0.09 | 0.03767 |
| cg22309826 | *COPS3* | 8533 | 0.08 | 0.03767 |
| cg22313025 | *HACE1* | 57531 | 0.11 | 0.03767 |
| cg22459930 | *TXNL5* | 84817 | 0.08 | 0.03767 |
| cg22813220 | *C1orf83* | 127428 | 0.12 | 0.03767 |
| cg23609821 | *NP* | 4860 | 0.11 | 0.03767 |
| cg23758485 | *SMPD3* | 55512 | 0.12 | 0.03767 |
| cg23824713 | *SERPINB5* | 5268 | -0.58 | 0.03767 |
| cg23837897 | *COX11* | 1353 | 0.10 | 0.03767 |
| cg23850272 | *ZNF228* | 7771 | 0.11 | 0.03767 |
| cg23871933 | *Pfs2* | 51659 | 0.10 | 0.03767 |
| cg24520800 | *RIOK1* | 83732 | 0.10 | 0.03767 |
| cg25094927 | *RANGAP1* | 5905 | 0.10 | 0.03767 |
| cg25136926 | *DDX12* | 440081 | -0.34 | 0.03767 |
| cg25234963 | *SFRS14* | 10147 | 0.10 | 0.03767 |
| cg25494227 | *C12orf59* | 120939 | 0.44 | 0.03767 |
| cg25771195 | *GTL3* | 29105 | -0.41 | 0.03767 |
| cg26054395 | *MTF2* | 22823 | 0.11 | 0.03767 |
| cg26601286 | *NSUN4* | 387338 | 0.10 | 0.03767 |
| cg26677874 | *LRRC28* | 123355 | 0.10 | 0.03767 |
| cg26741595 | *HIST1H2BD* | 3017 | 0.17 | 0.03767 |
| cg27088877 | *MON1A* | 84315 | 0.08 | 0.03767 |
| cg27262488 | *ITCH* | 83737 | 0.09 | 0.03767 |
| cg27305662 | *C6orf15* | 29113 | -0.12 | 0.03767 |
| cg27583102 | *KLK5* | 25818 | 0.19 | 0.03767 |
| cg00679738 | *FAM89A* | 375061 | 0.09 | 0.03767 |
| cg04322978 | *ETFB* | 2109 | 0.11 | 0.03767 |
| cg08173535 | *NR6A1* | 2649 | 0.14 | 0.03767 |
| cg15774283 | *PTGS2* | 5743 | 0.11 | 0.03767 |
| cg20504202 | *RP1-112K5.2* | 90121 | 0.12 | 0.03767 |
| cg27583030 | *SLC25A4* | 291 | 0.10 | 0.03767 |
| cg15963417 | *PAH* | 5053 | 0.12 | 0.03768 |
| cg06668300 | *MAL* | 4118 | 0.10 | 0.03769 |
| cg08524525 | *FLJ35630* | 166379 | 0.10 | 0.03769 |
| cg08656294 | *CD68* | 968 | 0.10 | 0.03769 |
| cg17100322 | *FYN* | 2534 | 0.12 | 0.03769 |
| cg04684516 | *SNCAIP* | 9627 | 0.13 | 0.03769 |
| cg15046735 | *C14orf156* | 81892 | 0.10 | 0.03769 |
| cg03764352 | *ETNK1* | 55500 | 0.10 | 0.03769 |
| cg04128563 | *AMD1* | 262 | 0.13 | 0.03769 |
| cg05461841 | *LOC124220* | 124220 | 0.55 | 0.03769 |
| cg08806153 | *HOXD11* | 3237 | 0.09 | 0.03769 |
| cg17009717 | *POLR1B* | 84172 | 0.10 | 0.03769 |
| cg20314660 | *DDX39* | 10212 | 0.14 | 0.03769 |
| cg22908581 | *CD4* | 920 | 0.25 | 0.03769 |
| cg23935746 | *CCNB1* | 891 | 0.14 | 0.03769 |
| cg26331192 | *GMNN* | 51053 | 0.10 | 0.03769 |
| cg26747293 | *FLJ39155* | 133584 | 0.12 | 0.03769 |
| cg00233307 | *MAP4K1* | 11184 | 0.10 | 0.03770 |
| cg00415993 | *F2RL2* | 2151 | 0.40 | 0.03770 |
| cg00622552 | *ODF3L1* | 161753 | 0.29 | 0.03770 |
| cg00696900 | *MAT2A* | 4144 | 0.10 | 0.03770 |
| cg01546046 | *AP4S1* | 11154 | -0.47 | 0.03770 |
| cg01716083 | *ZNRD1* | 30834 | 0.14 | 0.03770 |
| cg01739167 | *CHRNE* | 1145 | 0.32 | 0.03770 |
| cg01801174 | *UFC1* | 51506 | 0.07 | 0.03770 |
| cg02039171 | *CEBPE* | 1053 | 0.33 | 0.03770 |
| cg02314308 | *GLT25D2* | 23127 | 0.09 | 0.03770 |
| cg02496295 | *ADD1* | 118 | 0.10 | 0.03770 |
| cg02501984 | *POLE* | 5426 | 0.12 | 0.03770 |
| cg02806093 | *RAB2B* | 84932 | 0.10 | 0.03770 |
| cg03243700 | *WDR5* | 11091 | 0.10 | 0.03770 |
| cg03367667 | *STOML2* | 30968 | 0.09 | 0.03770 |
| cg04726446 | *MGC16372* | 92749 | 0.10 | 0.03770 |
| cg05486551 | *FLJ25067* | 149840 | 0.14 | 0.03770 |
| cg05521696 | *SLC2A14* | 144195 | 0.10 | 0.03770 |
| cg06192753 | *GADD45GIP1* | 90480 | 0.21 | 0.03770 |
| cg06222851 | *OGDHL* | 55753 | 0.16 | 0.03770 |
| cg06335814 | *FAM59A* | 64762 | 0.08 | 0.03770 |
| cg06415153 | *PITPNM2* | 57605 | 0.39 | 0.03770 |
| cg06491924 | *ZNF613* | 79898 | 0.11 | 0.03770 |
| cg06668073 | *C20orf42* | 55612 | 0.12 | 0.03770 |
| cg06755819 | *RCN1* | 5954 | -0.52 | 0.03770 |
| cg07371530 | *RPUSD1* | 113000 | -0.22 | 0.03770 |
| cg07426960 | *CCND1* | 595 | 0.13 | 0.03770 |
| cg09616556 | *AMN* | 81693 | -0.31 | 0.03770 |
| cg09630437 | *SLC39A3* | 29985 | 0.10 | 0.03770 |
| cg09773756 | *MMS19L* | 64210 | 0.11 | 0.03770 |
| cg09829319 | *GCM2* | 9247 | 0.12 | 0.03770 |
| cg10313093 | *TPARL* | 55858 | 0.09 | 0.03770 |
| cg11118665 | *TCFL5* | 10732 | 0.13 | 0.03770 |
| cg11372831 | *PPAT* | 5471 | 0.14 | 0.03770 |
| cg11393848 | *C1QC* | 714 | 0.28 | 0.03770 |
| cg12247247 | *RPL23* | 9349 | 0.13 | 0.03770 |
| cg12460105 | *RAD17* | 5884 | 0.14 | 0.03770 |
| cg12497692 | *ZBTB32* | 27033 | -0.11 | 0.03770 |
| cg13255190 | *GALNT8* | 26290 | -0.37 | 0.03770 |
| cg13273136 | *PSD4* | 23550 | 0.38 | 0.03770 |
| cg13577407 | *FLNB* | 2317 | 0.09 | 0.03770 |
| cg14035045 | *C3orf18* | 51161 | -0.43 | 0.03770 |
| cg14939652 | *NPAS3* | 64067 | 0.11 | 0.03770 |
| cg14967066 | *IFITM1* | 8519 | 0.33 | 0.03770 |
| cg15512851 | *FGD2* | 221472 | 0.47 | 0.03770 |
| cg16306115 | *DCDC2* | 51473 | 0.14 | 0.03770 |
| cg16388829 | *ARID5A* | 10865 | 0.10 | 0.03770 |
| cg17026456 | *PXN* | 5829 | 0.12 | 0.03770 |
| cg17465304 | *KIF12* | 113220 | -0.54 | 0.03770 |
| cg17509872 | *CNOT7* | 29883 | 0.11 | 0.03770 |
| cg18379495 | *ZNF703* | 80139 | 0.11 | 0.03770 |
| cg19257550 | *CA9* | 768 | -0.18 | 0.03770 |
| cg19302283 | *BTC* | 685 | -0.49 | 0.03770 |
| cg20287234 | *GPR55* | 9290 | 0.40 | 0.03770 |
| cg20339230 | *ST8SIA2* | 8128 | 0.10 | 0.03770 |
| cg21111868 | *EGR4* | 1961 | 0.14 | 0.03770 |
| cg21678388 | *ZNF354B* | 117608 | 0.12 | 0.03770 |
| cg22082462 | *CPNE6* | 9362 | 0.31 | 0.03770 |
| cg22158956 | *UGDH* | 7358 | 0.13 | 0.03770 |
| cg22341310 | *ZNF541* | 84215 | 0.11 | 0.03770 |
| cg22395192 | *FAM46B* | 115572 | 0.11 | 0.03770 |
| cg22421766 | *SCNN1D* | 6339 | 0.44 | 0.03770 |
| cg22442730 | *SRMS* | 6725 | 0.34 | 0.03770 |
| cg22586527 | *SOX6* | 55553 | 0.25 | 0.03770 |
| cg23456692 | *EDEM1* | 9695 | 0.10 | 0.03770 |
| cg23495733 | *CPNE8* | 144402 | 0.12 | 0.03770 |
| cg23830326 | *NHN1* | 124245 | 0.11 | 0.03770 |
| cg24304712 | *C1orf101* | 257044 | 0.13 | 0.03770 |
| cg25634666 | *FOLR3* | 2352 | 0.41 | 0.03770 |
| cg25894551 | *FXYD2* | 486 | 0.30 | 0.03770 |
| cg26144265 | *BZW2* | 28969 | 0.10 | 0.03770 |
| cg26217633 | *NLE1* | 54475 | 0.08 | 0.03770 |
| cg26240433 | *FAM14B* | 122509 | 0.11 | 0.03770 |
| cg26261431 | *PBK* | 55872 | 0.10 | 0.03770 |
| cg26279025 | *IL11* | 3589 | -0.34 | 0.03770 |
| cg26704579 | *CYP1A1* | 1543 | 0.16 | 0.03770 |
| cg27037648 | *CASKIN2* | 57513 | 0.10 | 0.03770 |
| cg27185377 | *PTTG1* | 9232 | 0.11 | 0.03770 |
| cg10107186 | *PFDN5* | 5204 | 0.17 | 0.03771 |
| cg16920242 | *TEP1* | 7011 | -0.52 | 0.03772 |
| cg08530414 | *CD44* | 960 | 0.08 | 0.03773 |
| cg19803984 | *SNRPN* | 6638 | -0.40 | 0.03773 |
| cg23839398 | *KLHDC4* | 54758 | -0.36 | 0.03773 |
| cg24069559 | *HNRPL* | 3191 | 0.14 | 0.03773 |
| cg01999523 | *CLASP2* | 23122 | 0.10 | 0.03775 |
| cg14829378 | *B4GALT1* | 2683 | 0.09 | 0.03775 |
| cg18602314 | *GFPT2* | 9945 | 0.11 | 0.03776 |
| cg02912041 | *UNQ846* | 131920 | -0.19 | 0.03776 |
| cg07703401 | *HBQ1* | 3049 | 0.14 | 0.03776 |
| cg14471615 | *LRRC54* | 25987 | 0.10 | 0.03776 |
| cg17285325 | *ECGF1* | 1890 | 0.12 | 0.03776 |
| cg20141013 | *PRO0149* | 29035 | 0.07 | 0.03776 |
| cg21513553 | *COL6A2* | 1292 | 0.09 | 0.03776 |
| cg24618716 | *PTMA* | 5757 | 0.11 | 0.03776 |
| cg03309721 | *PLEKHJ1* | 55111 | 0.09 | 0.03780 |
| cg03843978 | *MSX1* | 4487 | -0.48 | 0.03780 |
| cg06459327 | *NOLA3* | 55505 | 0.14 | 0.03780 |
| cg08039281 | *PSMA6* | 5687 | 0.09 | 0.03780 |
| cg08045570 | *FOXF2* | 2295 | 0.09 | 0.03780 |
| cg09935540 | *DNAH3* | 55567 | 0.16 | 0.03780 |
| cg11730961 | *SFPQ* | 6421 | 0.14 | 0.03780 |
| cg13685294 | *GRM2* | 2912 | -0.59 | 0.03780 |
| cg15989091 | *LOXL3* | 84695 | -0.32 | 0.03780 |
| cg17413703 | *TAF11* | 6882 | 0.12 | 0.03780 |
| cg17935677 | *WDR76* | 79968 | 0.12 | 0.03780 |
| cg18219226 | *GUSB* | 2990 | -0.51 | 0.03780 |
| cg20879768 | *TCEB3B* | 51224 | -0.29 | 0.03780 |
| cg22349489 | *HIST1H2AH* | 85235 | 0.15 | 0.03780 |
| cg23843812 | *FLJ43582* | 389649 | 0.38 | 0.03780 |
| cg10224037 | *ZNF354A* | 6940 | 0.13 | 0.03781 |
| cg12437239 | *GOLPH4* | 27333 | 0.10 | 0.03781 |
| cg00948524 | *RNF135* | 84282 | 0.12 | 0.03782 |
| cg11483915 | *SLC40A1* | 30061 | 0.09 | 0.03782 |
| cg13649728 | *SLC25A31* | 83447 | -0.50 | 0.03782 |
| cg17142183 | *IL1R2* | 7850 | 0.35 | 0.03782 |
| cg08368934 | *GPR97* | 222487 | 0.45 | 0.03784 |
| cg00493400 | *STAT1* | 6772 | 0.13 | 0.03786 |
| cg01697732 | *FAM20A* | 54757 | 0.15 | 0.03786 |
| cg02656594 | *IL21R* | 50615 | 0.44 | 0.03786 |
| cg04780126 | *POLA2* | 23649 | 0.12 | 0.03786 |
| cg08321272 | *SLC30A9* | 10463 | 0.09 | 0.03786 |
| cg08614481 | *HTR1B* | 3351 | 0.10 | 0.03786 |
| cg09085639 | *MRPL12* | 6182 | 0.12 | 0.03786 |
| cg10494770 | *IGLL1* | 3543 | 0.30 | 0.03786 |
| cg13044223 | *RPS8* | 6202 | 0.11 | 0.03786 |
| cg14209569 | *COMMD4* | 54939 | 0.10 | 0.03786 |
| cg14688272 | *FN3KRP* | 79672 | -0.50 | 0.03786 |
| cg23412850 | *SOCS2* | 8835 | 0.10 | 0.03786 |
| cg24901042 | *TMPRSS2* | 7113 | 0.10 | 0.03786 |
| cg26104204 | *MAP3K10* | 4294 | 0.12 | 0.03786 |
| cg26317056 | *NNT* | 23530 | 0.08 | 0.03788 |
| cg14161730 | *TIMP1* | 7076 | 0.23 | 0.03789 |
| cg03029060 | *ZNF184* | 7738 | 0.11 | 0.03794 |
| cg00079563 | *ARFGAP3* | 26286 | 0.10 | 0.03795 |
| cg00168785 | *WDSUB1* | 151525 | 0.10 | 0.03795 |
| cg09676788 | *RNF128* | 79589 | 0.23 | 0.03795 |
| cg09937039 | *BATF* | 10538 | 0.42 | 0.03795 |
| cg14426525 | *SMPDL3A* | 10924 | 0.10 | 0.03795 |
| cg17524886 | *CHFR* | 55743 | -0.10 | 0.03795 |
| cg18546419 | *RAP80* | 51720 | 0.12 | 0.03795 |
| cg19875216 | *SRPRB* | 58477 | 0.08 | 0.03795 |
| cg22424746 | *VTCN1* | 79679 | 0.08 | 0.03795 |
| cg24904765 | *STAM2* | 10254 | 0.09 | 0.03795 |
| cg06230736 | *GATA3* | 2625 | 0.10 | 0.03796 |
| cg24809768 | *RARS* | 5917 | 0.08 | 0.03796 |
| cg07706067 | *DHRS7B* | 25979 | 0.11 | 0.03796 |
| cg13696012 | *BPIL1* | 80341 | -0.09 | 0.03796 |
| cg26943001 | *SPAG8* | 26206 | 0.08 | 0.03796 |
| cg09812049 | *WIRE* | 147179 | 0.32 | 0.03797 |
| cg14971781 | *ACBD3* | 64746 | 0.13 | 0.03797 |
| cg18671950 | *FBN1* | 2200 | 0.08 | 0.03797 |
| cg20182358 | *MLF1* | 4291 | 0.09 | 0.03797 |
| cg20391984 | *CAMK2D* | 817 | 0.10 | 0.03797 |
| cg20598389 | *C6orf89* | 221477 | 0.13 | 0.03797 |
| cg13741249 | *ZNF524* | 147807 | 0.10 | 0.03799 |
| cg15824080 | *TAF15* | 8148 | 0.13 | 0.03799 |
| cg01110312 | *GTF2H3* | 2967 | -0.36 | 0.03800 |
| cg06585893 | *CAPN9* | 10753 | 0.28 | 0.03800 |
| cg07369274 | *DNASE1L3* | 1776 | 0.36 | 0.03800 |
| cg09288658 | *ZAK* | 51776 | 0.11 | 0.03800 |
| cg14066280 | *ZNF613* | 79898 | 0.08 | 0.03800 |
| cg14350002 | *GAS1* | 2619 | 0.13 | 0.03800 |
| cg17217654 | *C3orf60* | 25915 | 0.10 | 0.03800 |
| cg23347400 | *RBM4B* | 83759 | 0.12 | 0.03800 |
| cg08085267 | *C17orf57* | 124989 | 0.12 | 0.03800 |
| cg04017769 | *TACC1* | 6867 | 0.10 | 0.03803 |
| cg11042320 | *PDGFRB* | 5159 | 0.11 | 0.03803 |
| cg11356705 | *NUP54* | 53371 | 0.10 | 0.03803 |
| cg16796590 | *MFSD2* | 84879 | 0.13 | 0.03803 |
| cg21609430 | *FAM62A* | 23344 | 0.10 | 0.03803 |
| cg25301180 | *CAST1* | 26059 | 0.11 | 0.03803 |
| cg25270201 | *WWC3* | 55841 | 0.16 | 0.03803 |
| cg21226861 | *COMMD3* | 23412 | 0.09 | 0.03807 |
| cg02859934 | *NAGPA* | 51172 | -0.48 | 0.03807 |
| cg17701785 | *YPEL5* | 51646 | 0.12 | 0.03807 |
| cg18619398 | *GNAS* | 2778 | -0.53 | 0.03807 |
| cg19105690 | *LYPLA3* | 23659 | 0.11 | 0.03807 |
| cg26129270 | *FZD5* | 7855 | 0.11 | 0.03807 |
| cg09418283 | *PAWR* | 5074 | 0.09 | 0.03807 |
| cg16319213 | *PCNT2* | 5116 | 0.09 | 0.03807 |
| cg00354258 | *ZIM2* | 23619 | -0.59 | 0.03808 |
| cg00453258 | *FAM26C* | 255022 | 0.52 | 0.03808 |
| cg01389761 | *TERC* | 7012 | 0.13 | 0.03808 |
| cg04033650 | *NSUN5* | 55695 | 0.11 | 0.03808 |
| cg04195127 | *WFDC1* | 58189 | 0.48 | 0.03808 |
| cg07506795 | *ZNF19* | 7567 | -0.38 | 0.03808 |
| cg10757144 | *PCDHB15* | 56121 | 0.16 | 0.03808 |
| cg13045611 | *SDF2* | 6388 | 0.13 | 0.03808 |
| cg16139316 | *S100A9* | 6280 | 0.48 | 0.03808 |
| cg16829154 | *NOTCH1* | 4851 | 0.13 | 0.03808 |
| cg17288121 | *DEFB103A* | 55894 | 0.42 | 0.03808 |
| cg17345480 | *SPPL3* | 121665 | 0.11 | 0.03808 |
| cg17352004 | *C14orf29* | 145447 | -0.40 | 0.03808 |
| cg23328124 | *GRLF1* | 2909 | -0.20 | 0.03808 |
| cg25462303 | *GCET2* | 257144 | 0.39 | 0.03808 |
| cg26624118 | *SLC43A1* | 8501 | 0.09 | 0.03808 |
| cg08551857 | *PAG1* | 55824 | 0.09 | 0.03810 |
| cg14354749 | *EIF1AX* | 1964 | 0.13 | 0.03810 |
| cg22228205 | *DDHD1* | 80821 | 0.12 | 0.03810 |
| cg05421946 | *ABHD6* | 57406 | 0.12 | 0.03810 |
| cg14149007 | *PRKD2* | 25865 | 0.11 | 0.03813 |
| cg17080277 | *PTPRB* | 5787 | 0.12 | 0.03813 |
| cg17233601 | *PTPN5* | 84867 | -0.36 | 0.03814 |
| cg20169062 | *HSPB3* | 8988 | 0.22 | 0.03814 |
| cg26079992 | *CDK5RAP1* | 51654 | 0.09 | 0.03814 |
| cg26475097 | *QRICH1* | 54870 | 0.08 | 0.03814 |
| cg02829654 | *LYST* | 1130 | 0.22 | 0.03815 |
| cg11564268 | *NOB1P* | 28987 | 0.10 | 0.03815 |
| cg19162106 | *GPC5* | 2262 | 0.15 | 0.03815 |
| cg01110846 | *KCTD13* | 253980 | 0.12 | 0.03815 |
| cg01151699 | *CACNG2* | 10369 | 0.13 | 0.03815 |
| cg01673583 | *BIRC1* | 4671 | -0.66 | 0.03815 |
| cg04185893 | *PTBP2* | 58155 | 0.10 | 0.03815 |
| cg05556717 | *CCL26* | 10344 | 0.39 | 0.03815 |
| cg07455975 | *PTD004* | 29789 | 0.09 | 0.03815 |
| cg08416046 | *IRS4* | 8471 | 0.17 | 0.03815 |
| cg08551633 | *CEACAM8* | 1088 | 0.19 | 0.03815 |
| cg09514589 | *ETFA* | 2108 | 0.09 | 0.03815 |
| cg09868882 | *GRM8* | 2918 | 0.13 | 0.03815 |
| cg09976774 | *FOXM1* | 2305 | 0.09 | 0.03815 |
| cg10289912 | *ZNRF2* | 223082 | 0.11 | 0.03815 |
| cg11373429 | *ICAM5* | 7087 | 0.16 | 0.03815 |
| cg12917695 | *HSPC268* | 154791 | 0.11 | 0.03815 |
| cg13043862 | *EYA1* | 2138 | 0.11 | 0.03815 |
| cg15238200 | *TRIM65* | 201292 | -0.49 | 0.03815 |
| cg15652120 | *ELK3* | 2004 | 0.14 | 0.03815 |
| cg17425616 | *SNX2* | 6643 | 0.13 | 0.03815 |
| cg17733100 | *C1orf106* | 55765 | 0.14 | 0.03815 |
| cg18081750 | *DUSP6* | 1848 | 0.11 | 0.03815 |
| cg19412675 | *SNX24* | 28966 | 0.09 | 0.03815 |
| cg20226764 | *MLN* | 4295 | 0.46 | 0.03815 |
| cg20291674 | *VIL2* | 7430 | 0.09 | 0.03815 |
| cg20427879 | *EML2* | 24139 | 0.13 | 0.03815 |
| cg21656748 | *SCARB2* | 950 | 0.11 | 0.03815 |
| cg22584335 | *ZNF512* | 84450 | 0.10 | 0.03815 |
| cg22837289 | *MDS028* | 55846 | 0.13 | 0.03815 |
| cg23213217 | *DEGS1* | 8560 | 0.11 | 0.03815 |
| cg23858565 | *CTCF* | 10664 | 0.12 | 0.03815 |
| cg24434118 | *TP53INP1* | 94241 | 0.12 | 0.03815 |
| cg27305303 | *OTOF* | 9381 | 0.27 | 0.03815 |
| cg27395450 | *GLYCTK* | 132158 | 0.12 | 0.03815 |
| cg05373276 | *C7orf28B* | 221960 | 0.08 | 0.03815 |
| cg06653796 | *LIME1* | 54923 | 0.51 | 0.03815 |
| cg16092956 | *ACSL1* | 2180 | 0.14 | 0.03815 |
| cg26005082 | *C19orf30* | 284424 | 0.10 | 0.03815 |
| cg16749930 | *IL18BP* | 10068 | 0.50 | 0.03816 |
| cg01598046 | *TRAIP* | 10293 | 0.11 | 0.03816 |
| cg03103192 | *SPATA18* | 132671 | -0.46 | 0.03816 |
| cg03679305 | *SLC2A5* | 6518 | 0.38 | 0.03816 |
| cg06994793 | *PRO1580* | 55374 | 0.11 | 0.03816 |
| cg09624565 | *NCF4* | 4689 | 0.34 | 0.03816 |
| cg10894453 | *ITM2A* | 9452 | 0.13 | 0.03816 |
| cg21667943 | *CIP29* | 84324 | 0.18 | 0.03816 |
| cg22396755 | *RAP1GA1* | 5909 | 0.14 | 0.03816 |
| cg24513045 | *RGS19* | 10287 | 0.15 | 0.03816 |
| cg26020513 | *GATA4* | 2626 | -0.47 | 0.03816 |
| cg26478074 | *HSD17B12* | 51144 | 0.08 | 0.03816 |
| cg18870712 | *ZNF551* | 90233 | 0.10 | 0.03817 |
| cg00425792 | *PEX12* | 5193 | 0.10 | 0.03818 |
| cg00746130 | *BAT5* | 7920 | 0.14 | 0.03818 |
| cg01090445 | *TRAIP* | 10293 | 0.10 | 0.03818 |
| cg02907300 | *TCEB3* | 6924 | 0.10 | 0.03818 |
| cg05566397 | *MLL5* | 55904 | 0.12 | 0.03818 |
| cg06001166 | *RPL3L* | 6123 | -0.10 | 0.03818 |
| cg07753644 | *P2RY11* | 5032 | 0.46 | 0.03818 |
| cg07917796 | *DIAPH2* | 1730 | 0.10 | 0.03818 |
| cg07928641 | *LAMC1* | 3915 | 0.17 | 0.03818 |
| cg08062469 | *SPAG5* | 10615 | 0.11 | 0.03818 |
| cg08872742 | *CDH5* | 1003 | 0.37 | 0.03818 |
| cg09088508 | *RHOF* | 54509 | 0.10 | 0.03818 |
| cg11096993 | *ACY3* | 91703 | -0.39 | 0.03818 |
| cg11837864 | *C2orf17* | 79137 | 0.09 | 0.03818 |
| cg11909310 | *DKFZP686A10121* | 85865 | 0.15 | 0.03818 |
| cg12124478 | *IQGAP3* | 128239 | 0.12 | 0.03818 |
| cg13145458 | *RNF185* | 91445 | 0.10 | 0.03818 |
| cg13502356 | *ZMPSTE24* | 10269 | 0.09 | 0.03818 |
| cg17067528 | *IER3* | 8870 | 0.13 | 0.03818 |
| cg17693270 | *DGAT2L6* | 347516 | 0.30 | 0.03818 |
| cg18204052 | *C12orf52* | 84934 | 0.09 | 0.03818 |
| cg18878891 | *GLE1L* | 2733 | 0.08 | 0.03818 |
| cg19826026 | *ARHGDIB* | 397 | 0.13 | 0.03818 |
| cg20149766 | *POLS* | 11044 | 0.10 | 0.03818 |
| cg20830531 | *NUDT6* | 11162 | 0.10 | 0.03818 |
| cg23750142 | *CA8* | 767 | 0.14 | 0.03818 |
| cg24124977 | *TBC1D16* | 125058 | 0.08 | 0.03818 |
| cg25202471 | *TFAP2A* | 7020 | 0.27 | 0.03818 |
| cg25268451 | *GNAS* | 2778 | -0.52 | 0.03818 |
| cg26149678 | *IL18BP* | 10068 | 0.43 | 0.03818 |
| cg26331247 | *FLJ33706* | 284805 | 0.23 | 0.03818 |
| cg26848126 | *CYSLTR1* | 10800 | 0.24 | 0.03818 |
| cg13828839 | *FXYD6* | 53826 | 0.14 | 0.03819 |
| cg01033160 | *CEBPG* | 1054 | 0.08 | 0.03820 |
| cg01103730 | *IL20* | 50604 | -0.51 | 0.03820 |
| cg03743584 | *PRAP1* | 118471 | 0.44 | 0.03820 |
| cg03956267 | *FLJ20297* | 55627 | 0.13 | 0.03820 |
| cg04947157 | *TMC6* | 11322 | 0.11 | 0.03820 |
| cg05111829 | *SUPV3L1* | 6832 | 0.11 | 0.03820 |
| cg05467106 | *ZNF689* | 115509 | 0.10 | 0.03820 |
| cg05937182 | *ST7* | 7982 | 0.10 | 0.03820 |
| cg06657741 | *SLC9A6* | 10479 | 0.15 | 0.03820 |
| cg06945625 | *SERPINB6* | 5269 | 0.18 | 0.03820 |
| cg07020407 | *PDCD6* | 10016 | 0.08 | 0.03820 |
| cg07423906 | *C17orf56* | 146705 | 0.11 | 0.03820 |
| cg07888234 | *TNFRSF11A* | 8792 | 0.09 | 0.03820 |
| cg10318258 | *RIPK3* | 11035 | -0.28 | 0.03820 |
| cg10539808 | *KCTD1* | 284252 | 0.15 | 0.03820 |
| cg12575181 | *PIGH* | 5283 | -0.44 | 0.03820 |
| cg12576145 | *AMMECR1* | 9949 | 0.20 | 0.03820 |
| cg13576061 | *IL6ST* | 3572 | 0.15 | 0.03820 |
| cg13613748 | *VPS4B* | 9525 | 0.12 | 0.03820 |
| cg14304761 | *SYK* | 6850 | 0.17 | 0.03820 |
| cg14602578 | *CASP8AP2* | 9994 | 0.10 | 0.03820 |
| cg15591837 | *PPIL1* | 51645 | 0.09 | 0.03820 |
| cg17206029 | *KIF4A* | 24137 | 0.11 | 0.03820 |
| cg17797182 | *SNRPD1* | 6632 | 0.11 | 0.03820 |
| cg17902858 | *NPDC1* | 56654 | 0.12 | 0.03820 |
| cg18383391 | *MON1B* | 22879 | 0.13 | 0.03820 |
| cg18738906 | *SCNN1A* | 6337 | -0.11 | 0.03820 |
| cg19663795 | *SEPT1* | 1731 | 0.44 | 0.03820 |
| cg19889584 | *LAT1-3TM* | 81893 | -0.52 | 0.03820 |
| cg19912436 | *PALM* | 5064 | 0.13 | 0.03820 |
| cg20587168 | *MGC33407* | 284382 | -0.46 | 0.03820 |
| cg20955688 | *TMEM71* | 137835 | 0.40 | 0.03820 |
| cg22881914 | *NID2* | 22795 | 0.09 | 0.03820 |
| cg22968401 | *PIPOX* | 51268 | 0.50 | 0.03820 |
| cg23504246 | *FGF7* | 2252 | 0.44 | 0.03820 |
| cg25214366 | *DEFB103A* | 55894 | 0.13 | 0.03820 |
| cg26352374 | *MGC10992* | 92922 | 0.11 | 0.03820 |
| cg26796283 | *MSX2* | 4488 | 0.17 | 0.03820 |
| cg06384053 | *FLJ20010* | 54494 | 0.19 | 0.03821 |
| cg21449655 | *CASP9* | 842 | 0.17 | 0.03825 |
| cg05528918 | *ZFP161* | 7541 | -0.54 | 0.03825 |
| cg11000221 | *DHX40* | 79665 | 0.14 | 0.03825 |
| cg17982671 | *IL28B* | 282617 | -0.09 | 0.03825 |
| cg26071978 | *HMFN0839* | 84803 | 0.10 | 0.03825 |
| cg27611665 | *FBXL12* | 54850 | 0.10 | 0.03825 |
| cg00426963 | *KIF27* | 55582 | 0.12 | 0.03825 |
| cg02171008 | *RPL27A* | 6157 | 0.12 | 0.03825 |
| cg03799530 | *LNK* | 10019 | 0.17 | 0.03825 |
| cg04454050 | *TREML1* | 340205 | 0.33 | 0.03825 |
| cg22681784 | *SPINK2* | 6691 | 0.11 | 0.03825 |
| cg23142935 | *CDR2* | 1039 | 0.08 | 0.03825 |
| cg25356214 | *POU6F1* | 5463 | 0.14 | 0.03825 |
| cg00044245 | *EPHA7* | 2045 | 0.14 | 0.03827 |
| cg00798206 | *OXSR1* | 9943 | 0.12 | 0.03827 |
| cg07566050 | *PEA15* | 8682 | -0.37 | 0.03827 |
| cg09904573 | *RNF122* | 79845 | 0.13 | 0.03827 |
| cg10177528 | *TRAF5* | 7188 | -0.30 | 0.03827 |
| cg11223864 | *GNB2* | 2783 | 0.13 | 0.03827 |
| cg11324740 | *OIP5* | 11339 | 0.11 | 0.03827 |
| cg11368791 | *PFDN6* | 10471 | 0.10 | 0.03827 |
| cg12181621 | *HIST1H3I* | 8354 | 0.18 | 0.03827 |
| cg12477012 | *NASP* | 4678 | 0.10 | 0.03827 |
| cg13240639 | *ZMAT5* | 55954 | 0.09 | 0.03827 |
| cg15817236 | *ALX4* | 60529 | 0.13 | 0.03827 |
| cg17850932 | *IL2* | 3558 | -0.15 | 0.03827 |
| cg18107827 | *ZNF593* | 51042 | 0.15 | 0.03827 |
| cg19167673 | *PDGFB* | 5155 | 0.12 | 0.03827 |
| cg21387302 | *RFX3* | 5991 | 0.12 | 0.03827 |
| cg21399079 | *GPR45* | 11250 | -0.42 | 0.03827 |
| cg22722822 | *CHIC2* | 26511 | 0.13 | 0.03827 |
| cg24104611 | *C22orf9* | 23313 | 0.11 | 0.03827 |
| cg24801210 | *PCNP* | 57092 | 0.12 | 0.03827 |
| cg24929737 | *EFNB2* | 1948 | 0.08 | 0.03827 |
| cg25085950 | *ATP5J* | 522 | 0.12 | 0.03827 |
| cg25920951 | *FAM70B* | 348013 | 0.09 | 0.03827 |
| cg26514492 | *GPR132* | 29933 | 0.43 | 0.03827 |
| cg00973286 | *TNFRSF1A* | 7132 | 0.44 | 0.03829 |
| cg05252264 | *FCAR* | 2204 | 0.40 | 0.03829 |
| cg00397740 | *PPOX* | 5498 | 0.13 | 0.03830 |
| cg01220033 | *WDR34* | 89891 | 0.11 | 0.03830 |
| cg08172935 | *AMZ2* | 51321 | 0.13 | 0.03830 |
| cg11051843 | *SEMA3C* | 10512 | 0.12 | 0.03830 |
| cg11832727 | *RERE* | 473 | 0.12 | 0.03830 |
| cg15584630 | *TSC22D2* | 9819 | 0.11 | 0.03830 |
| cg26453990 | *FIBP* | 9158 | 0.10 | 0.03830 |
| cg27190537 | *SUMF2* | 25870 | 0.13 | 0.03830 |
| cg01876612 | *ZDHHC7* | 55625 | 0.16 | 0.03831 |
| cg05725452 | *SDF2L1* | 23753 | 0.11 | 0.03831 |
| cg08254089 | *BPI* | 671 | 0.35 | 0.03831 |
| cg13062935 | *MRGPRX2* | 117194 | 0.43 | 0.03831 |
| cg15125634 | *RAB11A* | 8766 | 0.10 | 0.03831 |
| cg17307280 | *DRD1* | 1812 | 0.09 | 0.03831 |
| cg17342283 | *PRG1* | 5552 | 0.35 | 0.03831 |
| cg20050113 | *SLC9A2* | 6549 | -0.38 | 0.03831 |
| cg22253036 | *DDX6* | 1656 | 0.16 | 0.03831 |
| cg27207274 | *MYC* | 4609 | 0.15 | 0.03831 |
| cg01873645 | *C9orf85* | 138241 | 0.12 | 0.03831 |
| cg05558390 | *GNAS* | 2778 | -0.29 | 0.03831 |
| cg08996413 | *SLA* | 6503 | 0.42 | 0.03832 |
| cg21900416 | *SERTAD3* | 29946 | 0.10 | 0.03833 |
| cg00704310 | *KCTD15* | 79047 | 0.08 | 0.03835 |
| cg01630869 | *CDC42EP2* | 10435 | 0.11 | 0.03835 |
| cg02082571 | *CLEC4A* | 50856 | 0.37 | 0.03835 |
| cg02932689 | *MAP1LC3B* | 81631 | 0.13 | 0.03835 |
| cg03392046 | *CCDC75* | 253635 | 0.09 | 0.03835 |
| cg04496615 | *PPP3R1* | 5534 | 0.07 | 0.03835 |
| cg04562739 | *PCNA* | 5111 | 0.09 | 0.03835 |
| cg04856203 | *DMXL1* | 1657 | 0.09 | 0.03835 |
| cg05257479 | *WARS2* | 10352 | 0.08 | 0.03835 |
| cg07036775 | *POLR3GL* | 84265 | 0.10 | 0.03835 |
| cg07349094 | *AFF3* | 3899 | 0.47 | 0.03835 |
| cg07356771 | *CHDH* | 55349 | 0.08 | 0.03835 |
| cg08798523 | *MTMR6* | 9107 | 0.10 | 0.03835 |
| cg08974656 | *TMEM127* | 55654 | 0.11 | 0.03835 |
| cg09800519 | *RXRA* | 6256 | 0.17 | 0.03835 |
| cg10693071 | *TRIM36* | 55521 | 0.14 | 0.03835 |
| cg12572278 | *OAF* | 220323 | 0.12 | 0.03835 |
| cg13360150 | *LIN7C* | 55327 | 0.16 | 0.03835 |
| cg13692433 | *NCAM1* | 4684 | 0.10 | 0.03835 |
| cg14049461 | *GRID2* | 2895 | 0.12 | 0.03835 |
| cg14830952 | *E2F5* | 1875 | 0.10 | 0.03835 |
| cg15489301 | *AKR1B10* | 57016 | 0.22 | 0.03835 |
| cg16208136 | *GEM* | 2669 | 0.10 | 0.03835 |
| cg19622360 | *MTR* | 4548 | 0.11 | 0.03835 |
| cg20979896 | *PDRG1* | 81572 | 0.15 | 0.03835 |
| cg21228284 | *TXNL4B* | 54957 | 0.10 | 0.03835 |
| cg22499964 | *EGLN2* | 112398 | 0.12 | 0.03835 |
| cg22577136 | *IKBKE* | 9641 | 0.47 | 0.03835 |
| cg24000099 | *LOC221955* | 221955 | 0.13 | 0.03835 |
| cg24715106 | *AQP11* | 282679 | 0.10 | 0.03835 |
| cg24880821 | *TIGD1* | 200765 | 0.13 | 0.03835 |
| cg25124223 | *NUP43* | 348995 | 0.09 | 0.03835 |
| cg26508537 | *C10orf6* | 55719 | 0.11 | 0.03835 |
| cg27478651 | *ACADM* | 34 | 0.08 | 0.03835 |
| cg19318326 | *KIAA1919* | 91749 | 0.11 | 0.03836 |
| cg05508558 | *CR2* | 1380 | -0.27 | 0.03847 |
| cg03371306 | *FBXW7* | 55294 | 0.12 | 0.03848 |
| cg00689340 | *RTKN* | 6242 | -0.40 | 0.03850 |
| cg06000781 | *BST1* | 683 | -0.35 | 0.03850 |
| cg06649520 | *ARFIP1* | 27236 | 0.09 | 0.03850 |
| cg11714502 | *AK1* | 203 | -0.47 | 0.03850 |
| cg15339605 | *TFEC* | 22797 | 0.24 | 0.03850 |
| cg15839448 | *SFRP1* | 6422 | 0.09 | 0.03850 |
| cg18742441 | *ATP6AP1* | 537 | 0.18 | 0.03850 |
| cg08719081 | *ALX3* | 257 | -0.32 | 0.03855 |
| cg18502522 | *SCAMP2* | 10066 | 0.10 | 0.03855 |
| cg00848397 | *MGC4172* | 79154 | 0.10 | 0.03856 |
| cg02237119 | *WBSCR27* | 155368 | -0.44 | 0.03856 |
| cg02738086 | *POLR3H* | 171568 | -0.39 | 0.03856 |
| cg06398181 | *ZNF236* | 7776 | -0.32 | 0.03856 |
| cg07027513 | *B4GALT6* | 9331 | 0.08 | 0.03856 |
| cg12227762 | *GNPNAT1* | 64841 | 0.09 | 0.03856 |
| cg12629515 | *HIST1H2BO* | 8348 | -0.42 | 0.03856 |
| cg14034870 | *SFTPG* | 389376 | 0.19 | 0.03856 |
| cg14717946 | *RBBP5* | 5929 | 0.13 | 0.03856 |
| cg20877313 | *GLS2* | 27165 | 0.12 | 0.03856 |
| cg21652012 | *GABRP* | 2568 | -0.09 | 0.03856 |
| cg09665592 | *DPM2* | 8818 | 0.10 | 0.03857 |
| cg14708847 | *PAK3* | 5063 | 0.14 | 0.03859 |
| cg03614664 | *RBX1* | 9978 | 0.08 | 0.03860 |
| cg05511752 | *MED12* | 9968 | 0.16 | 0.03860 |
| cg05655556 | *PSMD2* | 5708 | 0.11 | 0.03860 |
| cg09997082 | *GIPR* | 2696 | 0.29 | 0.03860 |
| cg12914362 | *FZD8* | 8325 | 0.10 | 0.03860 |
| cg13897627 | *FLJ44674* | 400535 | 0.11 | 0.03860 |
| cg17177829 | *ATF5* | 22809 | 0.13 | 0.03860 |
| cg22074858 | *GBP3* | 2635 | 0.35 | 0.03860 |
| cg23877831 | *MRPL51* | 51258 | 0.10 | 0.03860 |
| cg00319761 | *PKMYT1* | 9088 | 0.11 | 0.03863 |
| cg09441152 | *PQLC1* | 80148 | 0.13 | 0.03863 |
| cg09688763 | *ZDHHC6* | 64429 | 0.13 | 0.03865 |
| cg01373706 | *TMEM86A* | 144110 | 0.11 | 0.03867 |
| cg01443452 | *GAPDHS* | 26330 | -0.45 | 0.03867 |
| cg02385791 | *ZNF570* | 148268 | 0.10 | 0.03867 |
| cg02422694 | *HOXB4* | 3214 | 0.11 | 0.03867 |
| cg02679745 | *FUT7* | 2529 | 0.37 | 0.03867 |
| cg04718414 | *CDC16* | 8881 | 0.11 | 0.03867 |
| cg09614401 | *ADRA1D* | 146 | -0.71 | 0.03867 |
| cg09881855 | *SNAI2* | 6591 | 0.10 | 0.03867 |
| cg11221513 | *C22orf9* | 23313 | 0.10 | 0.03867 |
| cg12739034 | *PTGER3* | 5733 | 0.07 | 0.03867 |
| cg13853198 | *FCER1G* | 2207 | 0.34 | 0.03867 |
| cg15894661 | *PHACS* | 84680 | 0.10 | 0.03867 |
| cg23704362 | *C8orf46* | 254778 | -0.44 | 0.03867 |
| cg24358529 | *PPIE* | 10450 | 0.11 | 0.03867 |
| cg24502084 | *MAPBPIP* | 28956 | 0.11 | 0.03867 |
| cg02254461 | *AXUD1* | 64651 | -0.50 | 0.03867 |
| cg03668539 | *PEX11G* | 92960 | -0.41 | 0.03867 |
| cg04462209 | *KLHL12* | 59349 | 0.11 | 0.03867 |
| cg04533291 | *WDR39* | 9391 | -0.53 | 0.03867 |
| cg05122605 | *MAEA* | 10296 | 0.09 | 0.03867 |
| cg05836145 | *SP8* | 221833 | -0.31 | 0.03867 |
| cg09018810 | *IDS* | 3423 | 0.11 | 0.03867 |
| cg13060405 | *MYO18A* | 399687 | 0.12 | 0.03867 |
| cg13064571 | *C8orf44* | 56260 | 0.30 | 0.03867 |
| cg13084909 | *RNF26* | 79102 | 0.11 | 0.03867 |
| cg26239233 | *CIITA* | 4261 | 0.45 | 0.03867 |
| cg17353431 | *THAP7* | 80764 | -0.13 | 0.03867 |
| cg25848060 | *SLBP* | 7884 | 0.12 | 0.03867 |
| cg01261535 | *N-PAC* | 84656 | 0.12 | 0.03870 |
| cg03925809 | *GTF2F1* | 2962 | 0.10 | 0.03870 |
| cg04871004 | *ZNF337* | 26152 | 0.09 | 0.03870 |
| cg17568996 | *NFAM1* | 150372 | 0.40 | 0.03870 |
| cg19506903 | *YIPF3* | 25844 | 0.11 | 0.03870 |
| cg19637116 | *FNTB* | 2342 | 0.14 | 0.03870 |
| cg20420433 | *HLXB9* | 3110 | 0.09 | 0.03870 |
| cg21539243 | *PHTF1* | 10745 | 0.13 | 0.03870 |
| cg27444994 | *CDH8* | 1006 | 0.10 | 0.03870 |
| cg11899895 | *TAX1BP1* | 8887 | 0.10 | 0.03871 |
| cg05978187 | *ERH* | 2079 | 0.09 | 0.03871 |
| cg01945391 | *RPL37* | 6167 | 0.09 | 0.03876 |
| cg05912121 | *TH* | 7054 | -0.09 | 0.03876 |
| cg06552050 | *CSDA* | 8531 | 0.12 | 0.03876 |
| cg06597861 | *LY6E* | 4061 | 0.11 | 0.03876 |
| cg07065941 | *KLF10* | 7071 | 0.08 | 0.03876 |
| cg09617747 | *NOB1P* | 28987 | 0.13 | 0.03876 |
| cg11041457 | *GNL3* | 26354 | 0.16 | 0.03876 |
| cg12434258 | *FLJ90396* | 163049 | 0.13 | 0.03876 |
| cg13430851 | *C10orf7* | 8872 | 0.10 | 0.03876 |
| cg14055374 | *MAF* | 4094 | 0.11 | 0.03876 |
| cg14086647 | *LIMK2* | 3985 | 0.12 | 0.03876 |
| cg14189614 | *SLC45A2* | 51151 | -0.34 | 0.03876 |
| cg14204791 | *PKNOX1* | 5316 | 0.25 | 0.03876 |
| cg15047833 | *POLR2C* | 5432 | 0.14 | 0.03876 |
| cg15415545 | *SULT1A3* | 6818 | 0.09 | 0.03876 |
| cg17471928 | *STAC2* | 342667 | 0.12 | 0.03876 |
| cg19776453 | *CEACAM1* | 634 | 0.12 | 0.03876 |
| cg21269934 | *FLJ37478* | 339983 | 0.10 | 0.03876 |
| cg23163471 | *SVIL* | 6840 | 0.09 | 0.03876 |
| cg23926526 | *MDM1* | 56890 | 0.11 | 0.03876 |
| cg24341236 | *MTCP1* | 4515 | 0.12 | 0.03876 |
| cg24526899 | *BMP4* | 652 | -0.33 | 0.03876 |
| cg24678429 | *PTPRE* | 5791 | 0.12 | 0.03876 |
| cg25682936 | *SF3A3* | 10946 | 0.11 | 0.03876 |
| cg27005179 | *ERBB2* | 2064 | 0.10 | 0.03876 |
| cg00512374 | *IGFBP4* | 3487 | 0.10 | 0.03878 |
| cg05026033 | *ELP3* | 55140 | 0.13 | 0.03878 |
| cg06204948 | *MARK2* | 2011 | 0.39 | 0.03878 |
| cg03131527 | *BDH* | 622 | 0.23 | 0.03879 |
| cg17572572 | *ATPBD1B* | 54707 | 0.08 | 0.03881 |
| cg20247102 | *RASA2* | 5922 | 0.11 | 0.03882 |
| cg10160407 | *PDGFRL* | 5157 | 0.11 | 0.03882 |
| cg00475955 | *C9orf114* | 51490 | 0.14 | 0.03882 |
| cg03165700 | *ATM* | 472 | 0.11 | 0.03882 |
| cg03985136 | *ULBP2* | 80328 | 0.12 | 0.03882 |
| cg08958913 | *LGALS14* | 56891 | -0.12 | 0.03882 |
| cg10608341 | *HESX1* | 8820 | 0.28 | 0.03882 |
| cg16580737 | *MME* | 4311 | 0.15 | 0.03882 |
| cg20994561 | *BAI1* | 575 | -0.09 | 0.03882 |
| cg23769143 | *TIMP4* | 7079 | 0.32 | 0.03882 |
| cg02358804 | *ZNF146* | 7705 | -0.14 | 0.03883 |
| cg02593766 | *EPN3* | 55040 | 0.50 | 0.03883 |
| cg03954858 | *FAM83F* | 113828 | 0.22 | 0.03883 |
| cg04798158 | *C14orf111* | 51077 | 0.13 | 0.03883 |
| cg06458239 | *ZNF549* | 256051 | 0.15 | 0.03883 |
| cg08535091 | *LOC283932* | 283932 | 0.13 | 0.03883 |
| cg09418321 | *DYRK4* | 8798 | 0.49 | 0.03883 |
| cg09470640 | *PTPRO* | 5800 | 0.10 | 0.03883 |
| cg12804976 | *NDUFA10* | 4705 | 0.13 | 0.03883 |
| cg13431205 | *RB1* | 5925 | -0.41 | 0.03883 |
| cg14602164 | *FBXO8* | 26269 | 0.09 | 0.03883 |
| cg17560327 | *ZNF417* | 147687 | 0.12 | 0.03883 |
| cg18780751 | *G6PD* | 2539 | 0.14 | 0.03883 |
| cg27225570 | *DUSP15* | 128853 | 0.08 | 0.03883 |
| cg17346115 | *POLA2* | 23649 | 0.08 | 0.03884 |
| cg21260850 | *CANT1* | 124583 | 0.12 | 0.03884 |
| cg24512973 | *MUC1* | 4582 | -0.13 | 0.03884 |
| cg06619076 | *ASPH* | 444 | 0.13 | 0.03884 |
| cg07948245 | *ARHGAP5* | 394 | 0.09 | 0.03884 |
| cg10182321 | *STK32B* | 55351 | 0.08 | 0.03884 |
| cg12163490 | *CDH11* | 1009 | 0.11 | 0.03884 |
| cg21989094 | *MRPS12* | 6183 | 0.08 | 0.03884 |
| cg24956866 | *CALD1* | 800 | -0.42 | 0.03884 |
| cg01907837 | *RNF8* | 9025 | -0.62 | 0.03884 |
| cg17371081 | *NELL1* | 4745 | 0.09 | 0.03884 |
| cg23850212 | *ZFP28* | 140612 | 0.10 | 0.03884 |
| cg24724583 | *PLXDC1* | 57125 | -0.20 | 0.03888 |
| cg06191203 | *RIF1* | 55183 | 0.10 | 0.03889 |
| cg20216935 | *TGFBR2* | 7048 | 0.11 | 0.03889 |
| cg12453288 | *FLJ25143* | 130813 | 0.11 | 0.03892 |
| cg16144006 | *CT45-2* | 158852 | -0.68 | 0.03898 |
| cg25398949 | *RPS6KA5* | 9252 | 0.13 | 0.03898 |
| cg01168201 | *HIC1* | 3090 | 0.10 | 0.03899 |
| cg01171588 | *USP38* | 84640 | 0.12 | 0.03899 |
| cg01409734 | *TRAFD1* | 10906 | 0.11 | 0.03899 |
| cg01611640 | *LOC58486* | 58486 | 0.12 | 0.03899 |
| cg04435377 | *FLJ31568* | 150244 | -0.55 | 0.03899 |
| cg04912278 | *MRPL4* | 51073 | 0.10 | 0.03899 |
| cg04988978 | *MPO* | 4353 | 0.47 | 0.03899 |
| cg05146205 | *CUTL1* | 1523 | 0.10 | 0.03899 |
| cg07785936 | *GPR39* | 2863 | -0.57 | 0.03899 |
| cg08399444 | *GSG1* | 83445 | 0.36 | 0.03899 |
| cg08775230 | *C11orf52* | 91894 | -0.44 | 0.03899 |
| cg10599444 | *MMP14* | 4323 | 0.12 | 0.03899 |
| cg12737574 | *PDIA3* | 2923 | 0.08 | 0.03899 |
| cg12902719 | *LIMD1* | 8994 | 0.12 | 0.03899 |
| cg13665593 | *ZNF435* | 80345 | 0.11 | 0.03899 |
| cg14992108 | *SNTB1* | 6641 | -0.52 | 0.03899 |
| cg15792688 | *FLJ90036* | 255403 | 0.15 | 0.03899 |
| cg17784922 | *KEL* | 3792 | -0.10 | 0.03899 |
| cg18393722 | *FLJ43276* | 388165 | 0.15 | 0.03899 |
| cg20938359 | *SLC6A12* | 6539 | 0.39 | 0.03899 |
| cg20956373 | *USP48* | 84196 | 0.12 | 0.03899 |
| cg21120249 | *FLJ36268* | 401563 | -0.31 | 0.03899 |
| cg21686900 | *DAZAP2* | 9802 | 0.11 | 0.03899 |
| cg21713257 | *ETFDH* | 2110 | 0.07 | 0.03899 |
| cg21912567 | *GADD45G* | 10912 | 0.08 | 0.03899 |
| cg21968169 | *LOC338799* | 338799 | 0.15 | 0.03899 |
| cg23261233 | *ACTB* | 60 | 0.10 | 0.03899 |
| cg24355174 | *FLJ21827* | 56912 | 0.10 | 0.03899 |
| cg26511321 | *HOXA7* | 3204 | 0.11 | 0.03899 |
| cg26757673 | *IL2RB* | 3560 | -0.11 | 0.03899 |
| cg00495442 | *KIAA1219* | 57148 | 0.12 | 0.03899 |
| cg00567479 | *FARP2* | 9855 | 0.12 | 0.03899 |
| cg01587454 | *WDR21C* | 138009 | -0.44 | 0.03899 |
| cg01663570 | *DVL3* | 1857 | 0.10 | 0.03899 |
| cg01868782 | *HEY2* | 23493 | 0.11 | 0.03899 |
| cg03159329 | *STK39* | 27347 | 0.13 | 0.03899 |
| cg04523589 | *CAMP* | 820 | 0.55 | 0.03899 |
| cg04944936 | *CXorf17* | 54954 | 0.11 | 0.03899 |
| cg07093046 | *MTR* | 4548 | 0.09 | 0.03899 |
| cg07884474 | *VPS16* | 64601 | 0.11 | 0.03899 |
| cg09726469 | *MCL1* | 4170 | 0.09 | 0.03899 |
| cg09786257 | *PCSK1* | 5122 | -0.50 | 0.03899 |
| cg10126923 | *NKG7* | 4818 | 0.39 | 0.03899 |
| cg10139846 | *INADL* | 10207 | 0.14 | 0.03899 |
| cg10234776 | *NRBF2* | 29982 | 0.12 | 0.03899 |
| cg10501065 | *IGF2AS* | 51214 | 0.15 | 0.03899 |
| cg11009736 | *MARCO* | 8685 | 0.40 | 0.03899 |
| cg12966875 | *SLPI* | 6590 | 0.43 | 0.03899 |
| cg13150441 | *RNF40* | 9810 | 0.07 | 0.03899 |
| cg13257485 | *NXT2* | 55916 | -0.22 | 0.03899 |
| cg13359415 | *LGI2* | 55203 | 0.10 | 0.03899 |
| cg13376158 | *NT5C* | 30833 | 0.09 | 0.03899 |
| cg13446235 | *AFG3L1* | 172 | 0.11 | 0.03899 |
| cg13940405 | *GOSR2* | 9570 | 0.11 | 0.03899 |
| cg14341579 | *IRS2* | 8660 | 0.11 | 0.03899 |
| cg14676817 | *C1orf108* | 79647 | 0.13 | 0.03899 |
| cg14679202 | *L3MBTL2* | 83746 | -0.40 | 0.03899 |
| cg14795968 | *ACADL* | 33 | 0.08 | 0.03899 |
| cg15309066 | *CYCS* | 54205 | 0.10 | 0.03899 |
| cg15787039 | *SGNE1* | 6447 | -0.48 | 0.03899 |
| cg16270890 | *FAHD1* | 81889 | 0.12 | 0.03899 |
| cg16732880 | *NARFL* | 64428 | 0.08 | 0.03899 |
| cg19717150 | *HNF4A* | 3172 | -0.40 | 0.03899 |
| cg20552296 | *HPSE2* | 60495 | 0.12 | 0.03899 |
| cg21070087 | *HECW1* | 23072 | -0.38 | 0.03899 |
| cg21554249 | *SUOX* | 6821 | 0.18 | 0.03899 |
| cg21788470 | *PTHLH* | 5744 | 0.24 | 0.03899 |
| cg23610018 | *VGLL4* | 9686 | 0.12 | 0.03899 |
| cg24754277 | *DAPK1* | 1612 | 0.12 | 0.03899 |
| cg24817430 | *MESDC1* | 59274 | 0.13 | 0.03899 |
| cg25694542 | *ZFYVE1* | 53349 | 0.09 | 0.03899 |
| cg27265637 | *SEC24C* | 9632 | 0.13 | 0.03899 |
| cg27318281 | *C18orf37* | 125476 | -0.41 | 0.03899 |
| cg04749104 | *STEAP3* | 55240 | 0.13 | 0.03900 |
| cg08432727 | *SOX11* | 6664 | 0.09 | 0.03900 |
| cg24992780 | *OR7C1* | 26664 | -0.49 | 0.03900 |
| cg25543676 | *SMARCE1* | 6605 | 0.10 | 0.03900 |
| cg26206598 | *PREX1* | 57580 | -0.56 | 0.03900 |
| cg08708323 | *C16orf34* | 90861 | 0.11 | 0.03902 |
| cg09190408 | *TBC1D10C* | 374403 | 0.33 | 0.03903 |
| cg09682183 | *UNC93A* | 54346 | 0.39 | 0.03903 |
| cg14377370 | *FLJ20280* | 54876 | 0.09 | 0.03903 |
| cg01837574 | *TRAPPC1* | 58485 | -0.37 | 0.03904 |
| cg05621401 | *RET* | 5979 | 0.11 | 0.03904 |
| cg22415472 | *SLC5A7* | 60482 | -0.34 | 0.03904 |
| cg25635352 | *B3GALT3* | 8706 | 0.12 | 0.03904 |
| cg22321558 | *FLJ90024* | 129303 | 0.10 | 0.03905 |
| cg05421688 | *C1orf76* | 148753 | 0.09 | 0.03906 |
| cg12759554 | *NAP1L5* | 266812 | -0.65 | 0.03906 |
| cg01465620 | *ACVR2B* | 93 | 0.14 | 0.03906 |
| cg03169715 | *HAS3* | 3038 | 0.13 | 0.03906 |
| cg09783309 | *DNAL4* | 10126 | 0.11 | 0.03906 |
| cg10016608 | *GAS2L2* | 246176 | 0.35 | 0.03906 |
| cg12040555 | *MGMT* | 4255 | -0.10 | 0.03906 |
| cg15555014 | *SYNGR2* | 9144 | -0.35 | 0.03906 |
| cg22467534 | *UNC13D* | 201294 | 0.45 | 0.03906 |
| cg24297976 | *WDR35* | 57539 | 0.11 | 0.03906 |
| cg02276686 | *PSMC1* | 5700 | 0.08 | 0.03907 |
| cg04137037 | *TEF* | 7008 | 0.11 | 0.03907 |
| cg13530946 | *IARS2* | 55699 | -0.31 | 0.03907 |
| cg02886284 | *CPE* | 1363 | 0.13 | 0.03907 |
| cg05059825 | *TTC9C* | 283237 | 0.09 | 0.03907 |
| cg05801648 | *SYK* | 6850 | 0.17 | 0.03907 |
| cg08216720 | *SLC31A1* | 1317 | 0.12 | 0.03907 |
| cg08349806 | *FLJ39599* | 255027 | 0.10 | 0.03907 |
| cg14424111 | *C20orf77* | 58490 | 0.12 | 0.03907 |
| cg14449051 | *SLC6A15* | 55117 | 0.11 | 0.03907 |
| cg21237591 | *IGF2AS* | 51214 | 0.11 | 0.03907 |
| cg22926560 | *PFKFB2* | 5208 | 0.12 | 0.03907 |
| cg26809210 | *STAMBP* | 10617 | 0.07 | 0.03907 |
| cg06655100 | *KLF8* | 11279 | 0.10 | 0.03908 |
| cg06418184 | *DCTN3* | 11258 | 0.11 | 0.03908 |
| cg19781133 | *KCNH2* | 3757 | 0.10 | 0.03908 |
| cg20131968 | *CCDC47* | 57003 | -0.35 | 0.03908 |
| cg15128898 | *FLJ23577* | 79925 | 0.09 | 0.03909 |
| cg20244073 | *MID1* | 4281 | 0.32 | 0.03909 |
| cg00728398 | *C1orf149* | 64769 | 0.10 | 0.03909 |
| cg09778734 | *FLJ40142* | 400073 | -0.36 | 0.03909 |
| cg07347137 | *ZCCHC11* | 23318 | 0.09 | 0.03910 |
| cg15241084 | *TLR7* | 51284 | 0.24 | 0.03910 |
| cg15786837 | *HOXB13* | 10481 | 0.09 | 0.03910 |
| cg01851208 | *C10orf58* | 84293 | 0.08 | 0.03911 |
| cg02000005 | *CRIP1* | 1396 | 0.11 | 0.03911 |
| cg03366896 | *ECHS1* | 1892 | 0.11 | 0.03911 |
| cg05232889 | *FOXP2* | 93986 | 0.35 | 0.03911 |
| cg06816161 | *EZH2* | 2146 | 0.12 | 0.03911 |
| cg11846956 | *KLK10* | 5655 | 0.08 | 0.03911 |
| cg11909865 | *LOC120379* | 120379 | 0.10 | 0.03911 |
| cg14212314 | *HSPD1* | 3329 | 0.10 | 0.03911 |
| cg17776353 | *BMPR1A* | 657 | 0.11 | 0.03911 |
| cg19234509 | *BCAS2* | 10286 | 0.10 | 0.03911 |
| cg19434221 | *C20orf22* | 26090 | 0.18 | 0.03911 |
| cg21972382 | *RSNL2* | 79745 | 0.11 | 0.03911 |
| cg24543400 | *TRIB2* | 28951 | 0.10 | 0.03911 |
| cg24964368 | *FPRL1* | 2358 | 0.42 | 0.03911 |
| cg25457027 | *PROC* | 5624 | -0.09 | 0.03911 |
| cg25537774 | *AGPAT2* | 10555 | 0.09 | 0.03911 |
| cg25705900 | *RNF144* | 9781 | 0.08 | 0.03911 |
| cg27465569 | *PUS7L* | 83448 | -0.56 | 0.03911 |
| cg24211388 | *AIF1* | 199 | 0.37 | 0.03912 |
| cg26754448 | *C12orf57* | 113246 | 0.10 | 0.03912 |
| cg09548084 | *SLC35B3* | 51000 | -0.41 | 0.03913 |
| cg14126210 | *MCFD2* | 90411 | 0.10 | 0.03913 |
| cg14385738 | *PTPN22* | 26191 | 0.39 | 0.03913 |
| cg07936037 | *SSR1* | 6745 | 0.08 | 0.03913 |
| cg08584430 | *IHPK1* | 9807 | 0.11 | 0.03913 |
| cg25203561 | *SFRS16* | 11129 | -0.50 | 0.03913 |
| cg10646402 | *PTPRO* | 5800 | 0.12 | 0.03915 |
| cg01632825 | *CLTCL1* | 8218 | 0.07 | 0.03915 |
| cg07929310 | *KIAA0513* | 9764 | 0.10 | 0.03915 |
| cg02169098 | *XRCC6* | 2547 | 0.13 | 0.03917 |
| cg15604008 | *FUSIP1* | 10772 | 0.13 | 0.03917 |
| cg20217872 | *NAV3* | 89795 | 0.29 | 0.03917 |
| cg00280814 | *FLJ31568* | 150244 | -0.42 | 0.03918 |
| cg03897136 | *HOOK1* | 51361 | 0.09 | 0.03918 |
| cg25276849 | *TFRC* | 7037 | 0.09 | 0.03918 |
| cg15819853 | *MFHAS1* | 9258 | 0.11 | 0.03919 |
| cg09172980 | *NAT8* | 9027 | 0.43 | 0.03919 |
| cg12277666 | *TDRD5* | 163589 | 0.10 | 0.03919 |
| cg18383160 | *C14orf24* | 283635 | 0.16 | 0.03920 |
| cg00980978 | *WFDC2* | 10406 | 0.09 | 0.03920 |
| cg01293143 | *TCEA2* | 6919 | 0.22 | 0.03920 |
| cg02269221 | *TRAF4* | 9618 | 0.07 | 0.03920 |
| cg03289369 | *AASDH* | 132949 | 0.11 | 0.03920 |
| cg03465847 | *CRSP2* | 9282 | 0.10 | 0.03920 |
| cg03605339 | *ARMC7* | 79637 | 0.09 | 0.03920 |
| cg03853987 | *CHST10* | 9486 | 0.19 | 0.03920 |
| cg03920233 | *DOC2A* | 8448 | 0.12 | 0.03920 |
| cg04490516 | *GBL* | 64223 | 0.13 | 0.03920 |
| cg05705583 | *CAMLG* | 819 | 0.09 | 0.03920 |
| cg06601473 | *MGC34830* | 120196 | -0.13 | 0.03920 |
| cg06707886 | *C7orf20* | 51608 | 0.11 | 0.03920 |
| cg08747377 | *CDH13* | 1012 | 0.11 | 0.03920 |
| cg13643585 | *HOXB9* | 3219 | 0.10 | 0.03920 |
| cg14115004 | *STARD3* | 10948 | 0.14 | 0.03920 |
| cg14306534 | *ZBTB2* | 57621 | 0.10 | 0.03920 |
| cg15201599 | *COG3* | 83548 | 0.11 | 0.03920 |
| cg16536918 | *AVP* | 551 | 0.57 | 0.03920 |
| cg16872560 | *PPP1R9A* | 55607 | 0.10 | 0.03920 |
| cg17787710 | *RAC3* | 5881 | 0.12 | 0.03920 |
| cg20636078 | *ATR* | 545 | 0.08 | 0.03920 |
| cg24693909 | *RG9MTD2* | 93587 | 0.09 | 0.03920 |
| cg25759517 | *PYCR1* | 5831 | 0.13 | 0.03920 |
| cg26041297 | *SCGB1D2* | 10647 | 0.48 | 0.03920 |
| cg17692403 | *R3HDML* | 140902 | 0.45 | 0.03921 |
| cg01532080 | *SSBP4* | 170463 | 0.11 | 0.03921 |
| cg13030582 | *MFAP4* | 4239 | 0.42 | 0.03925 |
| cg19001226 | *HOXD1* | 3231 | 0.07 | 0.03925 |
| cg08551088 | *C10orf119* | 79892 | 0.10 | 0.03925 |
| cg12072803 | *JMJD2B* | 23030 | 0.13 | 0.03925 |
| cg21379008 | *ATP8B2* | 57198 | 0.10 | 0.03925 |
| cg22411207 | *MOS* | 4342 | 0.10 | 0.03925 |
| cg11220635 | *RDH11* | 51109 | 0.06 | 0.03926 |
| cg17436805 | *MIPEP* | 4285 | 0.10 | 0.03926 |
| cg07123069 | *HOXC11* | 3227 | 0.12 | 0.03928 |
| cg01505176 | *VARS* | 7407 | 0.14 | 0.03929 |
| cg02693857 | *MXD3* | 83463 | 0.12 | 0.03929 |
| cg02763671 | *RANBP1* | 5902 | -0.09 | 0.03929 |
| cg08286169 | *PEX3* | 8504 | 0.09 | 0.03929 |
| cg09485593 | *XYLT1* | 64131 | -0.16 | 0.03929 |
| cg10994126 | *PAPPA2* | 60676 | 0.38 | 0.03929 |
| cg11703011 | *SERPINE2* | 5270 | 0.12 | 0.03929 |
| cg11747499 | *SSH1* | 54434 | 0.09 | 0.03929 |
| cg12813792 | *C20orf85* | 128602 | -0.27 | 0.03929 |
| cg21010197 | *CDCA8* | 55143 | 0.12 | 0.03929 |
| cg27606341 | *FYB* | 2533 | 0.38 | 0.03929 |
| cg00164898 | *MAPK11* | 5600 | 0.15 | 0.03930 |
| cg00324733 | *NUCB2* | 4925 | 0.11 | 0.03930 |
| cg00331433 | *SYAP1* | 94056 | 0.09 | 0.03930 |
| cg00702231 | *PLAGL1* | 5325 | -0.77 | 0.03930 |
| cg01833398 | *TARBP2* | 6895 | 0.09 | 0.03930 |
| cg02299279 | *UBL7* | 84993 | 0.08 | 0.03930 |
| cg02344238 | *FKSG44* | 83786 | 0.08 | 0.03930 |
| cg03944089 | *SGK* | 6446 | 0.15 | 0.03930 |
| cg04323365 | *GJB1* | 2705 | -0.12 | 0.03930 |
| cg04672450 | *SAMD11* | 148398 | 0.10 | 0.03930 |
| cg04828792 | *MX2* | 4600 | -0.10 | 0.03930 |
| cg05886367 | *BTNL9* | 153579 | 0.38 | 0.03930 |
| cg06384491 | *SPIN2* | 54466 | -0.40 | 0.03930 |
| cg06612452 | *PINK1* | 65018 | 0.07 | 0.03930 |
| cg06864275 | *KIAA0963* | 22904 | 0.12 | 0.03930 |
| cg07143898 | *SLIT1* | 6585 | 0.08 | 0.03930 |
| cg07477160 | *NDRG1* | 10397 | 0.08 | 0.03930 |
| cg08591489 | *CXorf56* | 63932 | 0.13 | 0.03930 |
| cg08687825 | *PRSS35* | 167681 | 0.09 | 0.03930 |
| cg08948050 | *SLC35A4* | 113829 | -0.42 | 0.03930 |
| cg09539408 | *STRN3* | 29966 | 0.07 | 0.03930 |
| cg09725013 | *GTF3C1* | 2975 | 0.09 | 0.03930 |
| cg09926892 | *RNPC2* | 9584 | 0.08 | 0.03930 |
| cg10129493 | *CD33* | 945 | 0.38 | 0.03930 |
| cg10266068 | *PGPEP1* | 54858 | 0.12 | 0.03930 |
| cg10368981 | *DPYSL2* | 1808 | 0.11 | 0.03930 |
| cg10373645 | *DUSP11* | 8446 | 0.11 | 0.03930 |
| cg10423227 | *AFTIPHILIN* | 54812 | 0.11 | 0.03930 |
| cg10435003 | *MSH5* | 4439 | 0.09 | 0.03930 |
| cg10722799 | *CLIC6* | 54102 | 0.16 | 0.03930 |
| cg10994858 | *MINPP1* | 9562 | 0.13 | 0.03930 |
| cg11659747 | *SCARB2* | 950 | 0.16 | 0.03930 |
| cg11700429 | *ANAPC1* | 64682 | 0.10 | 0.03930 |
| cg12054080 | *RAB3GAP2* | 25782 | 0.11 | 0.03930 |
| cg12320676 | *E2F6* | 1876 | 0.17 | 0.03930 |
| cg12339802 | *C1orf109* | 54955 | 0.11 | 0.03930 |
| cg12650635 | *AQP2* | 359 | 0.61 | 0.03930 |
| cg12685753 | *TEKT1* | 83659 | 0.11 | 0.03930 |
| cg12914014 | *USP7* | 7874 | -0.46 | 0.03930 |
| cg13426503 | *RBL2* | 5934 | 0.12 | 0.03930 |
| cg13619408 | *SARS* | 6301 | -0.17 | 0.03930 |
| cg14118546 | *KIAA1012* | 22878 | 0.09 | 0.03930 |
| cg14292823 | *TIGD5* | 84948 | -0.61 | 0.03930 |
| cg14846293 | *AXIN2* | 8313 | 0.09 | 0.03930 |
| cg15790852 | *CNOT6* | 57472 | 0.13 | 0.03930 |
| cg16069236 | *MRPL39* | 54148 | 0.11 | 0.03930 |
| cg16218424 | *CMAS* | 55907 | 0.14 | 0.03930 |
| cg16551261 | *LRRC6* | 23639 | 0.19 | 0.03930 |
| cg16695354 | *LYSMD2* | 256586 | 0.15 | 0.03930 |
| cg17067993 | *CCR7* | 1236 | 0.33 | 0.03930 |
| cg17199483 | *ABCC1* | 4363 | 0.12 | 0.03930 |
| cg17639290 | *TIMM8B* | 26521 | 0.13 | 0.03930 |
| cg18017908 | *C17orf76* | 388341 | -0.23 | 0.03930 |
| cg18396533 | *DYDC1* | 143241 | -0.39 | 0.03930 |
| cg19848683 | *EVI1* | 2122 | 0.09 | 0.03930 |
| cg21500966 | *NAALAD2* | 10003 | 0.08 | 0.03930 |
| cg22136365 | *TAT* | 6898 | 0.33 | 0.03930 |
| cg22392708 | *KRT23* | 25984 | -0.51 | 0.03930 |
| cg22867063 | *ZNF214* | 7761 | 0.08 | 0.03930 |
| cg22946876 | *RASSF8* | 11228 | 0.12 | 0.03930 |
| cg23180365 | *GLB1* | 2720 | 0.13 | 0.03930 |
| cg23309825 | *MLC1SA* | 140465 | 0.12 | 0.03930 |
| cg23838531 | *TTC19* | 54902 | 0.11 | 0.03930 |
| cg23919568 | *FAM32A* | 26017 | 0.14 | 0.03930 |
| cg23980887 | *POLR2J* | 5439 | 0.11 | 0.03930 |
| cg25232934 | *THEX1* | 90459 | 0.08 | 0.03930 |
| cg25344639 | *SEC14L1* | 6397 | 0.12 | 0.03930 |
| cg26229274 | *SLC22A4* | 6583 | 0.12 | 0.03930 |
| cg26454191 | *PMS1* | 5378 | 0.08 | 0.03930 |
| cg26770882 | *AIPL1* | 23746 | -0.49 | 0.03930 |
| cg27109647 | *PSMC2* | 5701 | 0.12 | 0.03930 |
| cg27360560 | *TMEM67* | 91147 | 0.09 | 0.03930 |
| cg27365426 | *ARHGAP15* | 55843 | 0.48 | 0.03930 |
| cg27445265 | *BCCIP* | 56647 | 0.10 | 0.03930 |
| cg27504991 | *CUL7* | 9820 | -0.65 | 0.03930 |
| cg02224164 | *ARHGAP9* | 64333 | -0.33 | 0.03934 |
| cg00165981 | *COL4A3BP* | 10087 | 0.08 | 0.03935 |
| cg00833777 | *ITGAM* | 3684 | 0.31 | 0.03935 |
| cg02612026 | *CASP9* | 842 | 0.09 | 0.03935 |
| cg07284407 | *GNAS* | 2778 | -0.46 | 0.03935 |
| cg07311845 | *TMLHE* | 55217 | 0.18 | 0.03935 |
| cg20507276 | *OR2L13* | 284521 | -0.28 | 0.03935 |
| cg20785344 | *PGAM5* | 192111 | 0.10 | 0.03935 |
| cg15579370 | *SMOX* | 54498 | 0.12 | 0.03937 |
| cg20937139 | *PDGFC* | 56034 | 0.10 | 0.03938 |
| cg03625911 | *CHI3L1* | 1116 | 0.33 | 0.03938 |
| cg07175883 | *HOXD13* | 3239 | 0.10 | 0.03938 |
| cg12256228 | *RING1* | 6015 | 0.11 | 0.03938 |
| cg21303386 | *RGS7* | 6000 | 0.09 | 0.03938 |
| cg00876704 | *PKN1* | 5585 | 0.12 | 0.03939 |
| cg01646665 | *TMEM79* | 84283 | -0.46 | 0.03939 |
| cg02741249 | *GGA2* | 23062 | 0.12 | 0.03939 |
| cg06147863 | *SPI1* | 6688 | 0.40 | 0.03939 |
| cg06785429 | *DCUN1D1* | 54165 | -0.16 | 0.03939 |
| cg07919162 | *SF4* | 57794 | 0.09 | 0.03939 |
| cg07930578 | *LTK* | 4058 | -0.37 | 0.03939 |
| cg09486567 | *OVOL2* | 58495 | 0.10 | 0.03939 |
| cg10585962 | *MOGAT2* | 80168 | 0.12 | 0.03939 |
| cg12593411 | *ANGPTL6* | 83854 | 0.28 | 0.03939 |
| cg14023558 | *HNRPA3* | 220988 | 0.12 | 0.03939 |
| cg14036856 | *MGC52423* | 149466 | -0.35 | 0.03939 |
| cg15118503 | *AHSA2* | 130872 | 0.22 | 0.03939 |
| cg17727529 | *C1orf61* | 10485 | 0.40 | 0.03939 |
| cg19082446 | *SGK3* | 23678 | 0.11 | 0.03939 |
| cg22188058 | *SDC4* | 6385 | 0.14 | 0.03939 |
| cg27461196 | *FXYD1* | 5348 | 0.34 | 0.03939 |
| cg27579745 | *LDB1* | 8861 | 0.08 | 0.03939 |
| cg10392768 | *ALOXE3* | 59344 | 0.08 | 0.03939 |
| cg01043831 | *PTTG2* | 10744 | -0.18 | 0.03940 |
| cg01194444 | *MYOG* | 4656 | -0.09 | 0.03940 |
| cg01335367 | *C12orf34* | 84915 | 0.37 | 0.03940 |
| cg01385430 | *VPS45A* | 11311 | 0.10 | 0.03940 |
| cg01956781 | *KIAA1838* | 84498 | 0.09 | 0.03940 |
| cg02056135 | *SP110* | 3431 | 0.09 | 0.03940 |
| cg07184439 | *MGAT2* | 4247 | 0.09 | 0.03940 |
| cg07342901 | *H19* | 283120 | -0.15 | 0.03940 |
| cg13214349 | *HBP1* | 26959 | 0.10 | 0.03940 |
| cg16521028 | *GLIPR1* | 11010 | 0.33 | 0.03940 |
| cg16648297 | *ZNF576* | 79177 | 0.10 | 0.03940 |
| cg17226343 | *DIRAS1* | 148252 | 0.11 | 0.03940 |
| cg17304878 | *WDR68* | 10238 | 0.09 | 0.03940 |
| cg18023724 | *LOC92345* | 92345 | -0.43 | 0.03940 |
| cg21842478 | *HOXB13* | 10481 | 0.10 | 0.03940 |
| cg23033866 | *MRPL12* | 6182 | 0.09 | 0.03940 |
| cg18979819 | *IQCG* | 84223 | 0.10 | 0.03942 |
| cg16236875 | *C1R* | 715 | 0.46 | 0.03943 |
| cg07080007 | *RPL27* | 6155 | 0.10 | 0.03943 |
| cg24836175 | *CSNK2B* | 1460 | 0.13 | 0.03943 |
| cg25463409 | *COX7B2* | 170712 | 0.16 | 0.03943 |
| cg17580045 | *CCND2* | 894 | 0.13 | 0.03948 |
| cg00344372 | *HGFAC* | 3083 | 0.14 | 0.03948 |
| cg09096031 | *C2orf11* | 130132 | 0.22 | 0.03948 |
| cg22048492 | *HTATIP* | 10524 | 0.10 | 0.03948 |
| cg00312919 | *STARD3NL* | 83930 | 0.12 | 0.03949 |
| cg02367951 | *HIST1H2AK* | 8330 | -0.58 | 0.03949 |
| cg10215079 | *TAC3* | 6866 | -0.14 | 0.03949 |
| cg13277160 | *LOC151194* | 151194 | 0.09 | 0.03949 |
| cg14570389 | *RAP2C* | 57826 | 0.17 | 0.03949 |
| cg18237405 | *CPNE5* | 57699 | 0.09 | 0.03949 |
| cg20271396 | *C1orf19* | 116461 | 0.11 | 0.03949 |
| cg01108476 | *TCP11* | 6954 | -0.65 | 0.03950 |
| cg02801485 | *ALS2CR2* | 55437 | 0.11 | 0.03950 |
| cg04291079 | *MEG3* | 55384 | -0.43 | 0.03950 |
| cg08307039 | *TRIM23* | 373 | 0.11 | 0.03950 |
| cg11460029 | *EFNB3* | 1949 | 0.09 | 0.03950 |
| cg15248035 | *CCIN* | 881 | 0.42 | 0.03950 |
| cg17408686 | *CHCHD6* | 84303 | -0.18 | 0.03950 |
| cg17484237 | *HAVCR2* | 84868 | 0.34 | 0.03950 |
| cg18306327 | *BFAR* | 51283 | 0.15 | 0.03950 |
| cg20538819 | *PTPN14* | 5784 | 0.07 | 0.03950 |
| cg25575065 | *ARSA* | 410 | 0.13 | 0.03950 |
| cg26255848 | *FBL* | 2091 | 0.12 | 0.03950 |
| cg20476877 | *NIFUN* | 23479 | 0.11 | 0.03951 |
| cg21972430 | *TPD52L1* | 7164 | 0.10 | 0.03951 |
| cg01221484 | *ZNF584* | 201514 | 0.09 | 0.03952 |
| cg01418124 | *ARHGAP24* | 83478 | 0.11 | 0.03952 |
| cg01988129 | *ADHFE1* | 137872 | 0.13 | 0.03952 |
| cg03386722 | *FASN* | 2194 | 0.08 | 0.03952 |
| cg03404502 | *GPR18* | 2841 | 0.22 | 0.03952 |
| cg04317031 | *FAM73B* | 84895 | 0.12 | 0.03952 |
| cg04663487 | *TFEC* | 22797 | 0.35 | 0.03952 |
| cg06655089 | *IMPDH2* | 3615 | 0.08 | 0.03952 |
| cg08878352 | *PPP1R11* | 6992 | 0.12 | 0.03952 |
| cg11868029 | *CTSC* | 1075 | 0.08 | 0.03952 |
| cg12497298 | *C1orf166* | 79594 | 0.11 | 0.03952 |
| cg14296129 | *RAB5B* | 5869 | 0.17 | 0.03952 |
| cg15576233 | *HEY1* | 23462 | 0.10 | 0.03952 |
| cg15649193 | *GPSN2* | 9524 | 0.11 | 0.03952 |
| cg15842400 | *GABPA* | 2551 | 0.08 | 0.03952 |
| cg17158059 | *MGC15407* | 112942 | 0.08 | 0.03952 |
| cg17264470 | *FGF21* | 26291 | -0.08 | 0.03952 |
| cg18341054 | *NARS2* | 79731 | 0.11 | 0.03952 |
| cg18602919 | *NDN* | 4692 | -0.41 | 0.03952 |
| cg18731014 | *ZNF639* | 51193 | 0.12 | 0.03952 |
| cg18891404 | *ZC3H12A* | 80149 | 0.09 | 0.03952 |
| cg20353465 | *ABHD1* | 84696 | 0.11 | 0.03952 |
| cg20839149 | *SH2D2A* | 9047 | 0.39 | 0.03952 |
| cg20932765 | *TRIM33* | 51592 | 0.14 | 0.03952 |
| cg23832061 | *PPIL6* | 285755 | 0.08 | 0.03952 |
| cg25270252 | *ASTE1* | 28990 | 0.13 | 0.03952 |
| cg26029248 | *PFKFB4* | 5210 | 0.11 | 0.03952 |
| cg07220442 | *NUP107* | 57122 | 0.11 | 0.03952 |
| cg07465864 | *YTHDC2* | 64848 | -0.26 | 0.03952 |
| cg03395546 | *ADCK4* | 79934 | 0.12 | 0.03954 |
| cg10000775 | *PDLIM3* | 27295 | 0.17 | 0.03956 |
| cg07967643 | *FRS3* | 10817 | 0.08 | 0.03956 |
| cg20736065 | *MSI2* | 124540 | 0.12 | 0.03956 |
| cg03266904 | *CNOT4* | 4850 | 0.11 | 0.03957 |
| cg19317715 | *AOC2* | 314 | 0.32 | 0.03957 |
| cg22764341 | *ATP10D* | 57205 | 0.09 | 0.03957 |
| cg03690162 | *BAG3* | 9531 | 0.11 | 0.03957 |
| cg21081971 | *TRPC4* | 7223 | -0.29 | 0.03959 |
| cg02248540 | *HIST1H2AI* | 8329 | 0.11 | 0.03961 |
| cg14652095 | *HIST1H1A* | 3024 | -0.40 | 0.03961 |
| cg17597195 | *TMEM126B* | 55863 | 0.12 | 0.03961 |
| cg24821554 | *GUCY1B2* | 2974 | 0.43 | 0.03962 |
| cg16293991 | *CPSF3L* | 54973 | 0.15 | 0.03963 |
| cg01699584 | *KCTD8* | 386617 | 0.07 | 0.03964 |
| cg26681123 | *SLC34A3* | 142680 | 0.08 | 0.03964 |
| cg22346765 | *UNC5CL* | 222643 | 0.11 | 0.03965 |
| cg06452647 | *MCRS1* | 10445 | 0.17 | 0.03967 |
| cg13108328 | *PLEKHA5* | 54477 | 0.13 | 0.03967 |
| cg19974223 | *ANKRD33* | 341405 | -0.28 | 0.03967 |
| cg22802739 | *DBF4* | 10926 | 0.14 | 0.03967 |
| cg03804985 | *SLC2A8* | 29988 | 0.14 | 0.03968 |
| cg27147306 | *STK3* | 6788 | 0.17 | 0.03971 |
| cg02005375 | *DDX18* | 8886 | 0.10 | 0.03971 |
| cg02640558 | *PPP3CC* | 5533 | 0.12 | 0.03971 |
| cg03133269 | *WNT4* | 54361 | 0.12 | 0.03971 |
| cg04663194 | *ACTN3* | 89 | -0.10 | 0.03971 |
| cg07075930 | *PTPRM* | 5797 | 0.06 | 0.03971 |
| cg08818984 | *NR3C1* | 2908 | 0.62 | 0.03971 |
| cg13044136 | *AES* | 166 | 0.09 | 0.03971 |
| cg13343486 | *KLK10* | 5655 | 0.10 | 0.03971 |
| cg14409083 | *EMP1* | 2012 | 0.40 | 0.03971 |
| cg15177917 | *DAND5* | 199699 | 0.45 | 0.03971 |
| cg15674997 | *GRM6* | 2916 | -0.64 | 0.03971 |
| cg18418460 | *ULBP3* | 79465 | 0.12 | 0.03971 |
| cg26565838 | *GALNT10* | 55568 | 0.14 | 0.03971 |
| cg01662650 | *YY2* | 404281 | -0.41 | 0.03971 |
| cg05348870 | *TNFSF14* | 8740 | -0.12 | 0.03971 |
| cg06540715 | *ANKRD10* | 55608 | 0.16 | 0.03971 |
| cg06981182 | *C10orf59* | 55328 | 0.12 | 0.03971 |
| cg07777378 | *GRN* | 2896 | 0.11 | 0.03971 |
| cg08077345 | *PLEKHA4* | 57664 | -0.15 | 0.03971 |
| cg12810190 | *CSAD* | 51380 | 0.11 | 0.03971 |
| cg13706352 | *COX10* | 1352 | 0.11 | 0.03971 |
| cg19977912 | *PIGQ* | 9091 | 0.11 | 0.03971 |
| cg20315136 | *RGS2* | 5997 | 0.08 | 0.03971 |
| cg20376767 | *CGB1* | 114335 | -0.64 | 0.03971 |
| cg21859434 | *CXCR4* | 7852 | 0.09 | 0.03971 |
| cg21966410 | *AR* | 367 | 0.11 | 0.03971 |
| cg22375192 | *IGF1R* | 3480 | 0.10 | 0.03971 |
| cg23093462 | *PARD3* | 56288 | 0.10 | 0.03971 |
| cg23248587 | *XRCC4* | 7518 | 0.11 | 0.03971 |
| cg25753817 | *INPP1* | 3628 | 0.09 | 0.03971 |
| cg27291501 | *PEMT* | 10400 | 0.09 | 0.03971 |
| cg01774645 | *ARHGAP30* | 257106 | 0.52 | 0.03973 |
| cg12507125 | *WWTR1* | 25937 | 0.11 | 0.03976 |
| cg24697329 | *ARHGEF4* | 50649 | -0.35 | 0.03976 |
| cg07947016 | *KLK2* | 3817 | 0.14 | 0.03976 |
| cg13696887 | *KRTHB2* | 3888 | -0.12 | 0.03976 |
| cg13729392 | *C14orf112* | 51241 | 0.07 | 0.03976 |
| cg14287742 | *BLZF1* | 8548 | -0.43 | 0.03976 |
| cg20322862 | *TGIF* | 7050 | -0.24 | 0.03976 |
| cg20966551 | *MAST1* | 22983 | 0.08 | 0.03976 |
| cg22307908 | *ZNF264* | 9422 | 0.12 | 0.03976 |
| cg22438810 | *LCN2* | 3934 | 0.32 | 0.03976 |
| cg24270244 | *RBM7* | 10179 | 0.07 | 0.03976 |
| cg27431396 | *C20orf144* | 128864 | -0.37 | 0.03976 |
| cg27100123 | *SERPINB8* | 5271 | -0.43 | 0.03977 |
| cg01177524 | *NEDD9* | 4739 | 0.12 | 0.03979 |
| cg03389133 | *SSTR3* | 6753 | -0.13 | 0.03979 |
| cg04737405 | *OR12D2* | 26529 | -0.41 | 0.03979 |
| cg06957329 | *MRPS23* | 51649 | 0.10 | 0.03979 |
| cg08307816 | *DEGS2* | 123099 | 0.12 | 0.03979 |
| cg08439468 | *BICD2* | 23299 | 0.10 | 0.03979 |
| cg08578641 | *DNAI1* | 27019 | -0.48 | 0.03979 |
| cg10294836 | *DYRK1B* | 9149 | 0.10 | 0.03979 |
| cg11323731 | *AKR1A1* | 10327 | 0.12 | 0.03979 |
| cg13250711 | *ARNTL* | 406 | 0.15 | 0.03979 |
| cg13809962 | *ZNF6* | 7552 | 0.10 | 0.03979 |
| cg14217102 | *RAB1A* | 5861 | 0.11 | 0.03979 |
| cg14645481 | *HINT1* | 3094 | 0.12 | 0.03979 |
| cg16821394 | *NDFIP1* | 80762 | 0.10 | 0.03979 |
| cg19635810 | *C2orf18* | 54978 | 0.09 | 0.03979 |
| cg21137823 | *PRKCZ* | 5590 | 0.10 | 0.03979 |
| cg21424904 | *GRK6* | 2870 | 0.11 | 0.03979 |
| cg21504624 | *IL11RA* | 3590 | 0.25 | 0.03979 |
| cg22467216 | *TUBB6* | 84617 | 0.12 | 0.03979 |
| cg27236973 | *KRT17* | 3872 | 0.43 | 0.03979 |
| cg27519140 | *RPP21* | 79897 | -0.36 | 0.03979 |
| cg03874886 | *FLJ14397* | 84865 | 0.10 | 0.03982 |
| cg14059475 | *C2orf34* | 79823 | 0.09 | 0.03982 |
| cg05508067 | *PNPLA4* | 8228 | 0.13 | 0.03982 |
| cg06899808 | *MAGEA6* | 4105 | -0.36 | 0.03982 |
| cg24399405 | *C16orf35* | 8131 | -0.59 | 0.03982 |
| cg01291404 | *COL2A1* | 1280 | 0.13 | 0.03982 |
| cg14046757 | *FLJ11806* | 79882 | 0.10 | 0.03982 |
| cg23732182 | *C21orf84* | 114038 | 0.32 | 0.03983 |
| cg02160826 | *MRPS16* | 51021 | 0.06 | 0.03983 |
| cg10201668 | *NIP* | 90527 | -0.25 | 0.03983 |
| cg16050349 | *PIK3CB* | 5291 | 0.17 | 0.03983 |
| cg16539629 | *C14orf132* | 56967 | 0.11 | 0.03983 |
| cg20083676 | *EDG3* | 1903 | 0.09 | 0.03983 |
| cg01095590 | *KIAA0040* | 9674 | 0.10 | 0.03986 |
| cg02823866 | *CD274* | 29126 | 0.11 | 0.03986 |
| cg02937479 | *FKBPL* | 63943 | 0.11 | 0.03986 |
| cg03292388 | *CPZ* | 8532 | 0.09 | 0.03986 |
| cg03386869 | *ITGBL1* | 9358 | -0.30 | 0.03986 |
| cg03608167 | *CXCR4* | 7852 | 0.08 | 0.03986 |
| cg07251788 | *CLTCL1* | 8218 | -0.37 | 0.03986 |
| cg09893068 | *CCDC17* | 149483 | -0.31 | 0.03986 |
| cg10280342 | *PSPN* | 5623 | -0.10 | 0.03986 |
| cg14074117 | *MGC35212* | 254528 | 0.24 | 0.03986 |
| cg15206330 | *WDR79* | 55135 | 0.08 | 0.03986 |
| cg15316289 | *CRELD2* | 79174 | -0.39 | 0.03986 |
| cg15488402 | *SLC9A8* | 23315 | 0.18 | 0.03986 |
| cg16142977 | *EEF2* | 1938 | 0.11 | 0.03986 |
| cg17469479 | *CLEC9A* | 283420 | -0.37 | 0.03986 |
| cg23563234 | *PCDHGB7* | 56099 | 0.09 | 0.03986 |
| cg25082710 | *IVL* | 3713 | 0.27 | 0.03986 |
| cg27573806 | *LSM2* | 57819 | 0.08 | 0.03986 |
| cg00262415 | *PUSL1* | 126789 | 0.11 | 0.03987 |
| cg02850329 | *DSCR1L2* | 11123 | 0.12 | 0.03987 |
| cg03475420 | *RTN4R* | 65078 | 0.09 | 0.03987 |
| cg08308257 | *SMNDC1* | 10285 | 0.11 | 0.03987 |
| cg13351161 | *SCARA3* | 51435 | -0.39 | 0.03987 |
| cg14747225 | *POLR3K* | 51728 | 0.13 | 0.03987 |
| cg15419274 | *DNTTIP1* | 116092 | 0.11 | 0.03987 |
| cg15963971 | *FEN1* | 2237 | 0.13 | 0.03987 |
| cg16250754 | *CARD6* | 84674 | 0.26 | 0.03987 |
| cg18312782 | *TEX2* | 55852 | 0.15 | 0.03987 |
| cg21032203 | *ZNF265* | 9406 | 0.11 | 0.03987 |
| cg25763788 | *HTR1B* | 3351 | 0.11 | 0.03987 |
| cg01931893 | *TK2* | 7084 | 0.15 | 0.03987 |
| cg06849477 | *PRR5* | 55615 | 0.39 | 0.03987 |
| cg12802819 | *PUS3* | 83480 | 0.11 | 0.03987 |
| cg00915206 | *GAD1* | 2571 | 0.14 | 0.03989 |
| cg03223878 | *BHLHB2* | 8553 | 0.12 | 0.03989 |
| cg03665605 | *ATP5J2* | 9551 | -0.43 | 0.03989 |
| cg05592527 | *ARMCX6* | 54470 | 0.10 | 0.03989 |
| cg07249409 | *C1orf123* | 54987 | 0.11 | 0.03989 |
| cg07487535 | *DHCR7* | 1717 | 0.10 | 0.03989 |
| cg10091705 | *PPP1R12B* | 4660 | 0.07 | 0.03989 |
| cg10573763 | *COPE* | 11316 | 0.10 | 0.03989 |
| cg12716838 | *B3GALT3* | 8706 | 0.11 | 0.03989 |
| cg13262752 | *YIPF1* | 54432 | 0.10 | 0.03989 |
| cg14930396 | *SF3B14* | 51639 | 0.11 | 0.03989 |
| cg14961550 | *MCM3* | 4172 | 0.15 | 0.03989 |
| cg15720535 | *AGPAT2* | 10555 | -0.34 | 0.03989 |
| cg15857475 | *RECQL4* | 9401 | 0.11 | 0.03989 |
| cg17422427 | *RNPEPL1* | 57140 | 0.10 | 0.03989 |
| cg18139900 | *ZNF140* | 7699 | 0.14 | 0.03989 |
| cg20277250 | *TINP1* | 10412 | -0.45 | 0.03989 |
| cg23687186 | *IPO9* | 55705 | 0.11 | 0.03989 |
| cg23905308 | *PER2* | 8864 | 0.12 | 0.03989 |
| cg23984434 | *GUCY1A2* | 2977 | 0.11 | 0.03989 |
| cg26986815 | *CASP8* | 841 | -0.41 | 0.03989 |
| cg27253386 | *BRCA2* | 675 | 0.09 | 0.03989 |
| cg21182322 | *POU2F1* | 5451 | 0.11 | 0.03989 |
| cg23660999 | *PDDC1* | 347862 | 0.09 | 0.03989 |
| cg26091510 | *LRIG2* | 9860 | 0.11 | 0.03990 |
| cg02936263 | *CCL7* | 6354 | -0.15 | 0.03991 |
| cg04108240 | *TRA2A* | 29896 | -0.39 | 0.03991 |
| cg22029504 | *PGLS* | 25796 | 0.16 | 0.03991 |
| cg24851490 | *RNASE2* | 6036 | 0.43 | 0.03991 |
| cg00821764 | *UMPS* | 7372 | 0.11 | 0.03993 |
| cg16878652 | *GPS1* | 2873 | 0.12 | 0.03993 |
| cg21736592 | *PSCD4* | 27128 | 0.36 | 0.03993 |
| cg27038439 | *MSX1* | 4487 | -0.40 | 0.03994 |
| cg17384145 | *HIST1H2AM* | 8336 | 0.15 | 0.03994 |
| cg00128877 | *MKNK1* | 8569 | 0.08 | 0.03996 |
| cg02512860 | *CLDN15* | 24146 | 0.34 | 0.03996 |
| cg03338064 | *KIF2* | 3796 | -0.18 | 0.03996 |
| cg04057485 | *PTGIR* | 5739 | -0.68 | 0.03996 |
| cg05676042 | *PPME1* | 51400 | 0.09 | 0.03996 |
| cg05906362 | *ARHGAP4* | 393 | 0.10 | 0.03996 |
| cg06105085 | *C10orf104* | 119504 | 0.12 | 0.03996 |
| cg06240124 | *PLK1* | 5347 | 0.10 | 0.03996 |
| cg10484958 | *PCDH8* | 5100 | 0.13 | 0.03996 |
| cg10923408 | *MGC40178* | 122046 | -0.34 | 0.03996 |
| cg11360768 | *WHSC1L1* | 54904 | 0.07 | 0.03996 |
| cg11469778 | *ARHGEF4* | 50649 | -0.42 | 0.03996 |
| cg11471666 | *EIF4B* | 1975 | 0.07 | 0.03996 |
| cg11911418 | *ELAC1* | 55520 | -0.44 | 0.03996 |
| cg13234848 | *AUTS2* | 26053 | 0.14 | 0.03996 |
| cg14386061 | *DOK2* | 9046 | 0.34 | 0.03996 |
| cg15098690 | *ZNF330* | 27309 | 0.16 | 0.03996 |
| cg15958424 | *ACPP* | 55 | 0.39 | 0.03996 |
| cg16371833 | *GPR34* | 2857 | 0.25 | 0.03996 |
| cg17711870 | *WWP1* | 11059 | -0.37 | 0.03996 |
| cg18235146 | *MGC5509* | 79074 | 0.06 | 0.03996 |
| cg18652852 | *MAFF* | 23764 | 0.11 | 0.03996 |
| cg20590849 | *MIF4GD* | 57409 | 0.10 | 0.03996 |
| cg21413009 | *DAPK2* | 23604 | 0.17 | 0.03996 |
| cg22096513 | *UBA2* | 10054 | 0.14 | 0.03996 |
| cg25084525 | *NPAL1* | 152519 | 0.11 | 0.03996 |
| cg26624273 | *SNIP1* | 79753 | 0.08 | 0.03996 |
| cg27208307 | *PRG1* | 5552 | 0.41 | 0.03996 |
| cg09087966 | *CCNG2* | 901 | 0.09 | 0.03996 |
| cg07536920 | *RORB* | 6096 | 0.09 | 0.03996 |
| cg21032583 | *LMLN* | 89782 | -0.44 | 0.03996 |
| cg23637791 | *ODF3L1* | 161753 | 0.27 | 0.03996 |
| cg12732155 | *LAPTM5* | 7805 | 0.42 | 0.03997 |
| cg17841529 | *OSBPL8* | 114882 | 0.10 | 0.03997 |
| cg18160978 | *ZNF668* | 79759 | 0.16 | 0.03997 |
| cg21086464 | *PAK1* | 5058 | 0.12 | 0.03997 |
| cg04581377 | *SLC35F2* | 54733 | 0.10 | 0.03998 |
| cg11341528 | *NBLA04196* | 64921 | 0.13 | 0.03998 |
| cg11391732 | *HSPB3* | 8988 | 0.44 | 0.03998 |
| cg12197254 | *C16orf9* | 83986 | 0.11 | 0.03998 |
| cg18325289 | *USP48* | 84196 | 0.10 | 0.03998 |
| cg25002911 | *RB1* | 5925 | -0.23 | 0.03998 |
| cg26675934 | *NDUFB10* | 4716 | 0.10 | 0.03998 |
| cg10501629 | *ATP2B1* | 490 | -0.15 | 0.03999 |
| cg10735116 | *GPR89A* | 51463 | 0.12 | 0.03999 |
| cg16501028 | *WT1* | 7490 | -0.33 | 0.03999 |
| cg02238504 | *ZNF423* | 23090 | -0.09 | 0.03999 |
| cg20792833 | *PTPRCAP* | 5790 | 0.38 | 0.03999 |
| cg25922239 | *LEMD2* | 221496 | 0.10 | 0.03999 |
| cg04997967 | *KCNIP2* | 30819 | 0.12 | 0.03999 |
| cg18062196 | *DHX40* | 79665 | -0.41 | 0.03999 |
| cg03684977 | *GRB7* | 2886 | -0.39 | 0.04000 |
| cg17823175 | *AZU1* | 566 | 0.31 | 0.04000 |
| cg17617223 | *ZYG11BL* | 10444 | 0.12 | 0.04000 |
| cg07490776 | *AP3M2* | 10947 | 0.09 | 0.04001 |
| cg22498840 | *MCART1* | 92014 | 0.12 | 0.04001 |
| cg24315860 | *SRPK2* | 6733 | 0.14 | 0.04001 |
| cg25503559 | *MRPL24* | 79590 | 0.10 | 0.04001 |
| cg27304754 | *CALB2* | 794 | -0.36 | 0.04001 |
| cg11665588 | *SESN3* | 143686 | 0.10 | 0.04002 |
| cg19965511 | *HCRT* | 3060 | 0.11 | 0.04002 |
| cg25336579 | *PSAT1* | 29968 | 0.07 | 0.04002 |
| cg00793774 | *TRIM8* | 81603 | 0.11 | 0.04002 |
| cg01352882 | *STUB1* | 10273 | 0.10 | 0.04002 |
| cg01570086 | *ARID3B* | 10620 | 0.09 | 0.04002 |
| cg02442161 | *PI3* | 5266 | 0.46 | 0.04002 |
| cg03077492 | *CCL28* | 56477 | -0.13 | 0.04002 |
| cg03634997 | *GAS8* | 2622 | 0.08 | 0.04002 |
| cg04217218 | *ZDHHC21* | 340481 | -0.46 | 0.04002 |
| cg04531710 | *EIF4G1* | 1981 | 0.10 | 0.04002 |
| cg04662594 | *EPB49* | 2039 | 0.28 | 0.04002 |
| cg05830416 | *C9orf100* | 84904 | 0.07 | 0.04002 |
| cg05935584 | *HMGB3* | 3149 | 0.21 | 0.04002 |
| cg06334284 | *PPAP2B* | 8613 | 0.09 | 0.04002 |
| cg06508445 | *LOX* | 4015 | 0.14 | 0.04002 |
| cg07218880 | *UPF3A* | 65110 | -0.37 | 0.04002 |
| cg08331960 | *SLC9A3R2* | 9351 | -0.28 | 0.04002 |
| cg09253125 | *IQCD* | 115811 | 0.09 | 0.04002 |
| cg11018904 | *C20orf28* | 25876 | 0.14 | 0.04002 |
| cg11395917 | *PLEKHB2* | 55041 | 0.09 | 0.04002 |
| cg12788061 | *TIGD3* | 220359 | 0.12 | 0.04002 |
| cg14043411 | *SIVA* | 10572 | 0.07 | 0.04002 |
| cg14358502 | *NAT6* | 24142 | 0.12 | 0.04002 |
| cg14424579 | *FLJ21839* | 60509 | 0.10 | 0.04002 |
| cg14572485 | *AMZ2* | 51321 | 0.11 | 0.04002 |
| cg15530356 | *ZNF414* | 84330 | -0.52 | 0.04002 |
| cg16687447 | *PTEN* | 5728 | 0.11 | 0.04002 |
| cg16896371 | *CAND1* | 55832 | 0.12 | 0.04002 |
| cg18138552 | *PSMD8* | 5714 | 0.13 | 0.04002 |
| cg18855178 | *PCSK4* | 54760 | 0.09 | 0.04002 |
| cg19461344 | *SLC30A3* | 7781 | 0.08 | 0.04002 |
| cg20500081 | *LRCH4* | 4034 | -0.45 | 0.04002 |
| cg20646490 | *FOSB* | 2354 | 0.13 | 0.04002 |
| cg10246520 | *OLFM4* | 10562 | -0.10 | 0.04003 |
| cg16394138 | *XPR1* | 9213 | 0.09 | 0.04003 |
| cg04126427 | *EIF3S10* | 8661 | 0.11 | 0.04004 |
| cg22065439 | *C5orf15* | 56951 | 0.11 | 0.04004 |
| cg25492727 | *PTD004* | 29789 | 0.10 | 0.04004 |
| cg03469054 | *KIAA1944* | 121256 | 0.11 | 0.04005 |
| cg05670408 | *MAN1C1* | 57134 | 0.11 | 0.04005 |
| cg23185402 | *PHKB* | 5257 | 0.06 | 0.04005 |
| cg07940485 | *SLC16A10* | 117247 | 0.11 | 0.04005 |
| cg08872550 | *CA2* | 760 | 0.10 | 0.04006 |
| cg10936763 | *CDKN1A* | 1026 | 0.10 | 0.04006 |
| cg12478978 | *FLOT1* | 10211 | 0.07 | 0.04006 |
| cg13183539 | *FZR1* | 51343 | -0.09 | 0.04006 |
| cg13306164 | *HPCA* | 3208 | 0.10 | 0.04006 |
| cg14338548 | *LALBA* | 3906 | -0.08 | 0.04006 |
| cg15210596 | *SCCPDH* | 51097 | 0.10 | 0.04006 |
| cg15566783 | *NIPA2* | 81614 | 0.09 | 0.04006 |
| cg17248183 | *LOC162427* | 162427 | 0.12 | 0.04006 |
| cg19180541 | *MOBKL2B* | 79817 | -0.30 | 0.04006 |
| cg19311812 | *TAF7* | 6879 | 0.11 | 0.04006 |
| cg20199629 | *SAMD14* | 201191 | 0.12 | 0.04006 |
| cg23735442 | *SMG7* | 9887 | 0.13 | 0.04006 |
| cg24041995 | *LIG3* | 3980 | 0.11 | 0.04006 |
| cg24133115 | *PDE10A* | 10846 | 0.11 | 0.04006 |
| cg07404485 | *PON1* | 5444 | -0.43 | 0.04008 |
| cg15269294 | *NUP155* | 9631 | 0.07 | 0.04008 |
| cg01124420 | *EDAR* | 10913 | 0.20 | 0.04008 |
| cg01593385 | *FGG* | 2266 | 0.30 | 0.04008 |
| cg08637669 | *C9orf102* | 56959 | -0.15 | 0.04008 |
| cg12158019 | *OXCT2* | 64064 | -0.24 | 0.04008 |
| cg19182046 | *C14orf111* | 51077 | 0.14 | 0.04008 |
| cg19596653 | *KIAA0261* | 23063 | 0.10 | 0.04008 |
| cg27024922 | *POMT1* | 10585 | 0.10 | 0.04008 |
| cg27287808 | *PLK3* | 1263 | 0.13 | 0.04008 |
| cg04109220 | *SPAG8* | 26206 | 0.11 | 0.04009 |
| cg01120165 | *HDAC10* | 83933 | 0.10 | 0.04010 |
| cg01693108 | *SRPK1* | 6732 | 0.09 | 0.04010 |
| cg02702510 | *MARCH8* | 220972 | 0.16 | 0.04010 |
| cg03533811 | *HISPPD2A* | 9677 | -0.11 | 0.04010 |
| cg03751414 | *AP1S1* | 1174 | 0.12 | 0.04010 |
| cg04872689 | *PLEK* | 5341 | 0.41 | 0.04010 |
| cg06682185 | *SERPINB5* | 5268 | -0.40 | 0.04010 |
| cg07114024 | *BAHD1* | 22893 | 0.12 | 0.04010 |
| cg08458487 | *SFTPD* | 6441 | 0.39 | 0.04010 |
| cg08724563 | *RAX* | 30062 | 0.10 | 0.04010 |
| cg09818732 | *FLJ20489* | 55652 | 0.13 | 0.04010 |
| cg09985279 | *MT1G* | 4495 | 0.18 | 0.04010 |
| cg10171125 | *SCARA5* | 286133 | 0.14 | 0.04010 |
| cg10275770 | *ICAM2* | 3384 | 0.42 | 0.04010 |
| cg11973052 | *SOCS1* | 8651 | 0.10 | 0.04010 |
| cg12281657 | *C14orf50* | 145376 | 0.11 | 0.04010 |
| cg12864903 | *FLJ46247* | 374786 | -0.10 | 0.04010 |
| cg13906548 | *GABARAPL2* | 11345 | 0.11 | 0.04010 |
| cg14173969 | *CD300C* | 10871 | 0.44 | 0.04010 |
| cg15981753 | *SMA3* | 10571 | -0.35 | 0.04010 |
| cg18587364 | *ZNF562* | 54811 | 0.18 | 0.04010 |
| cg18844850 | *COPS7A* | 50813 | 0.11 | 0.04010 |
| cg19524049 | *NCDN* | 23154 | 0.10 | 0.04010 |
| cg19632760 | *LACTB2* | 51110 | -0.27 | 0.04010 |
| cg23234999 | *MKRN3* | 7681 | -0.61 | 0.04010 |
| cg25117362 | *C10orf77* | 79847 | 0.07 | 0.04010 |
| cg26644395 | *UCN3* | 114131 | -0.34 | 0.04010 |
| cg09343150 | *MEN1* | 4221 | -0.30 | 0.04011 |
| cg09585834 | *ANKS3* | 124401 | 0.13 | 0.04011 |
| cg15887846 | *CST6* | 1474 | -0.24 | 0.04012 |
| cg00061059 | *GPSN2* | 9524 | 0.15 | 0.04012 |
| cg00692549 | *TARDBP* | 23435 | 0.20 | 0.04012 |
| cg00887101 | *KIAA1698* | 80789 | 0.16 | 0.04012 |
| cg02318535 | *PPM1E* | 22843 | -0.35 | 0.04012 |
| cg06717104 | *TIMM23* | 10431 | 0.11 | 0.04012 |
| cg09227136 | *GOLGA1* | 2800 | 0.08 | 0.04012 |
| cg11155265 | *DDEFL1* | 55616 | -0.42 | 0.04012 |
| cg12438037 | *C9orf116* | 138162 | -0.43 | 0.04012 |
| cg13383572 | *PRH1* | 5554 | -0.08 | 0.04012 |
| cg13613532 | *MGC33948* | 160140 | 0.10 | 0.04012 |
| cg17229388 | *MGC35169* | 121793 | -0.15 | 0.04012 |
| cg17698643 | *C16orf63* | 123811 | 0.11 | 0.04012 |
| cg17989572 | *RAB5C* | 5878 | 0.07 | 0.04012 |
| cg18384403 | *TUBB2A* | 7280 | 0.11 | 0.04012 |
| cg18864581 | *ZWILCH* | 55055 | 0.14 | 0.04012 |
| cg20645649 | *RPL35* | 11224 | 0.09 | 0.04012 |
| cg21610516 | *MTHFD1L* | 25902 | 0.11 | 0.04012 |
| cg22591065 | *OBFC2B* | 79035 | 0.13 | 0.04012 |
| cg23724447 | *BUB3* | 9184 | 0.09 | 0.04012 |
| cg24022829 | *EIF2AK2* | 5610 | 0.09 | 0.04012 |
| cg14520511 | *KRIT1* | 889 | 0.10 | 0.04013 |
| cg21290885 | *TMEM47* | 83604 | 0.12 | 0.04013 |
| cg02210123 | *RHOJ* | 57381 | 0.12 | 0.04015 |
| cg02595263 | *MRPS28* | 28957 | 0.11 | 0.04015 |
| cg07308943 | *FLJ20628* | 55006 | 0.08 | 0.04015 |
| cg14550066 | *NCR1* | 9437 | 0.52 | 0.04015 |
| cg11221508 | *CCT6B* | 10693 | 0.09 | 0.04016 |
| cg00910168 | *DHX37* | 57647 | -0.39 | 0.04016 |
| cg04940515 | *RABGGTB* | 5876 | 0.09 | 0.04016 |
| cg09197965 | *TUBA1* | 7277 | 0.10 | 0.04016 |
| cg12431401 | *FLJ45909* | 126432 | 0.36 | 0.04016 |
| cg12530021 | *SIGLEC12* | 89858 | 0.32 | 0.04016 |
| cg14719055 | *RBBP7* | 5931 | -0.47 | 0.04016 |
| cg20070090 | *S100A8* | 6279 | 0.37 | 0.04016 |
| cg21259253 | *PHLDA2* | 7262 | 0.14 | 0.04016 |
| cg24450157 | *DHX16* | 8449 | 0.11 | 0.04016 |
| cg00800095 | *PNMA3* | 29944 | -0.58 | 0.04017 |
| cg07103819 | *DGAT1* | 8694 | 0.11 | 0.04017 |
| cg11600161 | *TBC1D10C* | 374403 | 0.37 | 0.04017 |
| cg16898420 | *PRG-3* | 54886 | 0.07 | 0.04017 |
| cg02965078 | *GPR44* | 11251 | 0.34 | 0.04018 |
| cg01282432 | *C14orf101* | 54916 | 0.11 | 0.04020 |
| cg07602026 | *PSME1* | 5720 | 0.09 | 0.04020 |
| cg23018891 | *BCAS2* | 10286 | 0.11 | 0.04020 |
| cg25023245 | *AXIN1* | 8312 | 0.12 | 0.04020 |
| cg02200132 | *EIF4A2* | 1974 | 0.08 | 0.04020 |
| cg09978128 | *NDEL1* | 81565 | 0.10 | 0.04020 |
| cg10307548 | *SOD3* | 6649 | 0.40 | 0.04020 |
| cg10613381 | *UPB1* | 51733 | -0.44 | 0.04020 |
| cg11393453 | *FAM59A* | 64762 | 0.11 | 0.04020 |
| cg11411707 | *MGC29891* | 126626 | 0.09 | 0.04020 |
| cg12953249 | *CRB2* | 286204 | -0.14 | 0.04020 |
| cg16606185 | *C21orf33* | 8209 | 0.09 | 0.04020 |
| cg17002259 | *CDC42SE1* | 56882 | 0.10 | 0.04020 |
| cg18796520 | *NDUFA9* | 4704 | 0.12 | 0.04020 |
| cg21126943 | *CEACAM6* | 4680 | 0.44 | 0.04020 |
| cg21183872 | *LONRF3* | 79836 | 0.16 | 0.04020 |
| cg21615127 | *TMCO4* | 255104 | 0.10 | 0.04020 |
| cg21728447 | *ALDH4A1* | 8659 | 0.11 | 0.04020 |
| cg22820108 | *NCOR2* | 9612 | 0.29 | 0.04020 |
| cg23858360 | *TSPAN14* | 81619 | 0.15 | 0.04020 |
| cg26866325 | *PPBPL2* | 10895 | 0.28 | 0.04020 |
| cg15743985 | *CD22* | 933 | 0.34 | 0.04020 |
| cg19149125 | *PROSC* | 11212 | -0.36 | 0.04020 |
| cg25531166 | *CTAG1B* | 1485 | -0.58 | 0.04020 |
| cg00658007 | *C1orf36* | 343035 | 0.47 | 0.04022 |
| cg04636194 | *PAGE4* | 9506 | -0.46 | 0.04022 |
| cg08341978 | *RERE* | 473 | 0.13 | 0.04022 |
| cg10184881 | *PTP4A2* | 8073 | 0.06 | 0.04022 |
| cg12879297 | *C16orf46* | 123775 | 0.07 | 0.04022 |
| cg16908782 | *WDHD1* | 11169 | 0.11 | 0.04022 |
| cg10920427 | *BXDC1* | 84154 | 0.10 | 0.04022 |
| cg21406461 | *IFI16* | 3428 | 0.32 | 0.04022 |
| cg26083396 | *IMPDH1* | 3614 | 0.08 | 0.04022 |
| cg27303882 | *PAGE2* | 203569 | -0.47 | 0.04022 |
| cg21321735 | *KIF1A* | 547 | 0.12 | 0.04025 |
| cg05088512 | *DKKL1* | 27120 | 0.14 | 0.04026 |
| cg17015234 | *RABL2A* | 11159 | 0.13 | 0.04027 |
| cg11058932 | *TSGA13* | 114960 | 0.22 | 0.04029 |
| cg07977490 | *C16orf45* | 89927 | -0.50 | 0.04030 |
| cg16245844 | *ADIPOR2* | 79602 | 0.09 | 0.04030 |
| cg13007988 | *MLL2* | 8085 | -0.15 | 0.04033 |
| cg02899723 | *DDX4* | 54514 | -0.44 | 0.04035 |
| cg13438543 | *TBCA* | 6902 | 0.08 | 0.04035 |
| cg18986165 | *SIGLEC12* | 89858 | -0.09 | 0.04035 |
| cg26615830 | *MSX1* | 4487 | 0.10 | 0.04035 |
| cg03025569 | *TRIM16* | 10626 | 0.30 | 0.04037 |
| cg05658858 | *TUBGCP2* | 10844 | 0.09 | 0.04037 |
| cg14493899 | *TAP2* | 6891 | -0.40 | 0.04038 |
| cg21460081 | *HOXB4* | 3214 | 0.14 | 0.04038 |
| cg15231886 | *UTP14A* | 10813 | 0.12 | 0.04039 |
| cg23126915 | *XRCC2* | 7516 | -0.42 | 0.04040 |
| cg02626929 | *PAQR4* | 124222 | 0.38 | 0.04042 |
| cg14254380 | *WDR63* | 126820 | 0.08 | 0.04044 |
| cg16050784 | *ASPH* | 444 | 0.10 | 0.04045 |
| cg19517525 | *ACTG1* | 71 | 0.13 | 0.04045 |
| cg24477567 | *EPOR* | 2057 | -0.27 | 0.04045 |
| cg26648592 | *HEBP1* | 50865 | 0.09 | 0.04045 |
| cg02061229 | *MMP10* | 4319 | 0.25 | 0.04048 |
| cg08799191 | *FLJ22222* | 79701 | 0.16 | 0.04048 |
| cg15425811 | *FLJ35801* | 150291 | -0.47 | 0.04048 |
| cg26055770 | *PDZRN3* | 23024 | 0.12 | 0.04048 |
| cg00893636 | *EPM2AIP1* | 9852 | 0.06 | 0.04048 |
| cg01193293 | *SIGLEC7* | 27036 | 0.18 | 0.04048 |
| cg01661993 | *TTYH1* | 57348 | 0.09 | 0.04048 |
| cg01746088 | *SFRS7* | 6432 | 0.08 | 0.04048 |
| cg02005755 | *SLC13A4* | 26266 | -0.32 | 0.04048 |
| cg02032115 | *IGSF8* | 93185 | 0.09 | 0.04048 |
| cg02678356 | *ZXDA* | 7789 | 0.11 | 0.04048 |
| cg03386903 | *KCNAB3* | 9196 | -0.39 | 0.04048 |
| cg03425110 | *NEUROG3* | 50674 | 0.09 | 0.04048 |
| cg03801286 | *KCNE1* | 3753 | 0.40 | 0.04048 |
| cg03846767 | *TP73* | 7161 | -0.08 | 0.04048 |
| cg03966541 | *PGGT1B* | 5229 | 0.09 | 0.04048 |
| cg03977657 | *LAMB3* | 3914 | -0.10 | 0.04048 |
| cg04453065 | *CYR61* | 3491 | 0.08 | 0.04048 |
| cg05593300 | *PRRX1* | 5396 | 0.16 | 0.04048 |
| cg05714479 | *LATS2* | 26524 | 0.10 | 0.04048 |
| cg06436504 | *DOC1* | 11259 | -0.11 | 0.04048 |
| cg06445611 | *GABRR2* | 2570 | -0.11 | 0.04048 |
| cg06504820 | *DLK1* | 8788 | -0.36 | 0.04048 |
| cg06691299 | *PCTK1* | 5127 | 0.11 | 0.04048 |
| cg06777274 | *ME2* | 4200 | 0.09 | 0.04048 |
| cg07331725 | *SERPINB5* | 5268 | -0.43 | 0.04048 |
| cg07596494 | *KIF3C* | 3797 | 0.09 | 0.04048 |
| cg07674153 | *TSHR* | 7253 | 0.10 | 0.04048 |
| cg07789083 | *GPRC5C* | 55890 | 0.09 | 0.04048 |
| cg08296824 | *OSBPL3* | 26031 | 0.11 | 0.04048 |
| cg08380539 | *CTBS* | 1486 | 0.13 | 0.04048 |
| cg08393541 | *NUDC* | 10726 | 0.14 | 0.04048 |
| cg08552999 | *ZNF644* | 84146 | 0.12 | 0.04048 |
| cg08792314 | *COASY* | 80347 | 0.12 | 0.04048 |
| cg08990057 | *PLS3* | 5358 | 0.14 | 0.04048 |
| cg09059635 | *NTNG2* | 84628 | 0.56 | 0.04048 |
| cg09096950 | *UBE4A* | 9354 | -0.43 | 0.04048 |
| cg09618028 | *COX11* | 1353 | 0.12 | 0.04048 |
| cg10520887 | *UNC5C* | 8633 | 0.10 | 0.04048 |
| cg10669058 | *CILP2* | 148113 | -0.38 | 0.04048 |
| cg10681137 | *ATPBD3* | 90353 | -0.61 | 0.04048 |
| cg11680158 | *C9orf52* | 158219 | 0.14 | 0.04048 |
| cg11754676 | *ZNF10* | 7556 | 0.14 | 0.04048 |
| cg11879480 | *SLC36A4* | 120103 | 0.13 | 0.04048 |
| cg11995732 | *MGC3731* | 79159 | 0.10 | 0.04048 |
| cg12200412 | *CD1E* | 913 | 0.16 | 0.04048 |
| cg12628062 | *TBPIP* | 29893 | 0.08 | 0.04048 |
| cg12693634 | *TMEM23* | 259230 | 0.15 | 0.04048 |
| cg12738197 | *ZNF572* | 137209 | 0.14 | 0.04048 |
| cg12871124 | *STARD4* | 134429 | 0.08 | 0.04048 |
| cg12949760 | *KCNQ1* | 3784 | 0.33 | 0.04048 |
| cg13216351 | *MOCS3* | 27304 | 0.14 | 0.04048 |
| cg13581941 | *CDC23* | 8697 | 0.08 | 0.04048 |
| cg13800022 | *ITGB1* | 3688 | 0.11 | 0.04048 |
| cg14159818 | *C20orf27* | 54976 | 0.06 | 0.04048 |
| cg14290451 | *RPL10A* | 4736 | 0.10 | 0.04048 |
| cg14324675 | *LST1* | 7940 | 0.45 | 0.04048 |
| cg15191648 | *SALL3* | 27164 | 0.09 | 0.04048 |
| cg15298594 | *NAG* | 51594 | 0.12 | 0.04048 |
| cg15703690 | *IL6* | 3569 | 0.07 | 0.04048 |
| cg16773028 | *KCNA2* | 3737 | -0.32 | 0.04048 |
| cg16793061 | *EYA1* | 2138 | 0.10 | 0.04048 |
| cg17044611 | *ASAH1* | 427 | 0.10 | 0.04048 |
| cg17096191 | *NOS1AP* | 9722 | 0.16 | 0.04048 |
| cg17177699 | *COL5A3* | 50509 | -0.23 | 0.04048 |
| cg17366325 | *RPL19* | 6143 | 0.08 | 0.04048 |
| cg17802847 | *CFLAR* | 8837 | 0.07 | 0.04048 |
| cg18074297 | *CLIC6* | 54102 | 0.09 | 0.04048 |
| cg18384168 | *FGF22* | 27006 | 0.10 | 0.04048 |
| cg18655584 | *SLC22A18* | 5002 | -0.15 | 0.04048 |
| cg19009921 | *RPL23A* | 6147 | 0.10 | 0.04048 |
| cg19112780 | *PTPRU* | 10076 | 0.10 | 0.04048 |
| cg19150884 | *TSTA3* | 7264 | 0.08 | 0.04048 |
| cg19432894 | *DNAJA1* | 3301 | 0.12 | 0.04048 |
| cg19875656 | *TSP50* | 29122 | -0.54 | 0.04048 |
| cg20303303 | *WDR1* | 9948 | 0.11 | 0.04048 |
| cg20376899 | *EDN1* | 1906 | 0.08 | 0.04048 |
| cg20458981 | *NARG1L* | 79612 | 0.09 | 0.04048 |
| cg20477070 | *ARG2* | 384 | 0.11 | 0.04048 |
| cg20641280 | *DXS9879E* | 8270 | 0.11 | 0.04048 |
| cg20648108 | *CTTNBP2NL* | 55917 | 0.11 | 0.04048 |
| cg21183846 | *FMO4* | 2329 | 0.15 | 0.04048 |
| cg21212956 | *ZDHHC4* | 55146 | 0.10 | 0.04048 |
| cg21621114 | *CRX* | 1406 | 0.29 | 0.04048 |
| cg21682902 | *HAL* | 3034 | 0.17 | 0.04048 |
| cg22129364 | *GPX7* | 2882 | 0.10 | 0.04048 |
| cg22464186 | *TNFRSF21* | 27242 | 0.12 | 0.04048 |
| cg22660578 | *LHX1* | 3975 | 0.09 | 0.04048 |
| cg22674717 | *SALL1* | 6299 | 0.10 | 0.04048 |
| cg23303782 | *GRK5* | 2869 | 0.07 | 0.04048 |
| cg24091474 | *TYROBP* | 7305 | 0.39 | 0.04048 |
| cg24192663 | *HSPA6* | 3310 | -0.37 | 0.04048 |
| cg24318412 | *ASH2L* | 9070 | 0.09 | 0.04048 |
| cg24353466 | *RNF13* | 11342 | 0.08 | 0.04048 |
| cg24482234 | *LASS1* | 10715 | 0.13 | 0.04048 |
| cg24975564 | *PDE3A* | 5139 | 0.12 | 0.04048 |
| cg24994350 | *LATS1* | 9113 | 0.13 | 0.04048 |
| cg25149927 | *SSH1* | 54434 | 0.12 | 0.04048 |
| cg25653103 | *SEPX1* | 51734 | 0.07 | 0.04048 |
| cg25766774 | *ZDHHC3* | 51304 | -0.36 | 0.04048 |
| cg25885771 | *HIST1H2AK* | 8330 | 0.13 | 0.04048 |
| cg26043391 | *FBXO28* | 23219 | 0.13 | 0.04048 |
| cg26093687 | *EIF3S2* | 8668 | -0.38 | 0.04048 |
| cg26171231 | *CLCN3* | 1182 | 0.14 | 0.04048 |
| cg26251101 | *RHOQ* | 23433 | 0.11 | 0.04048 |
| cg26285698 | *C16orf54* | 283897 | 0.40 | 0.04048 |
| cg26491213 | *SIX3* | 6496 | 0.11 | 0.04048 |
| cg26540515 | *ANGPT4* | 51378 | 0.22 | 0.04048 |
| cg26736450 | *OTX1* | 5013 | 0.10 | 0.04048 |
| cg26808606 | *COX17* | 10063 | 0.40 | 0.04048 |
| cg26928682 | *TREML2* | 79865 | 0.29 | 0.04048 |
| cg27011042 | *MFGE8* | 4240 | 0.10 | 0.04048 |
| cg27147004 | *PIWIL1* | 9271 | -0.47 | 0.04048 |
| cg27287498 | *IBRDC3* | 127544 | 0.11 | 0.04048 |
| cg27556566 | *RNF138* | 51444 | 0.12 | 0.04048 |
| cg27649653 | *ZNF8* | 7554 | 0.28 | 0.04048 |
| cg00897329 | *LACE1* | 246269 | 0.11 | 0.04050 |
| cg03144357 | *LRRC14* | 9684 | -0.28 | 0.04050 |
| cg07376232 | *AMICA1* | 120425 | 0.43 | 0.04050 |
| cg07437033 | *ZBTB17* | 7709 | 0.08 | 0.04050 |
| cg10643489 | *RQCD1* | 9125 | -0.47 | 0.04050 |
| cg10823157 | *ATP9B* | 374868 | 0.13 | 0.04050 |
| cg11140785 | *ZNF264* | 9422 | 0.09 | 0.04050 |
| cg13271963 | *ANKRD2* | 26287 | -0.07 | 0.04050 |
| cg15594133 | *TSPAN14* | 81619 | 0.11 | 0.04050 |
| cg16519321 | *MYOD1* | 4654 | 0.10 | 0.04050 |
| cg18149919 | *SIAHBP1* | 22827 | -0.49 | 0.04050 |
| cg20757758 | *ZMYND12* | 84217 | 0.12 | 0.04050 |
| cg21637033 | *TEAD4* | 7004 | 0.11 | 0.04050 |
| cg22262168 | *MOBKL2B* | 79817 | 0.13 | 0.04050 |
| cg00412805 | *KBTBD5* | 131377 | -0.47 | 0.04052 |
| cg16481281 | *IL11* | 3589 | 0.08 | 0.04052 |
| cg15235832 | *ACTR2* | 10097 | 0.13 | 0.04054 |
| cg03443922 | *CBLL1* | 79872 | 0.09 | 0.04055 |
| cg00292971 | *KIAA0773* | 9715 | -0.25 | 0.04058 |
| cg00754253 | *HRASLS5* | 117245 | 0.10 | 0.04058 |
| cg00837103 | *COL9A3* | 1299 | -0.24 | 0.04058 |
| cg01211097 | *USP10* | 9100 | -0.33 | 0.04058 |
| cg01313966 | *C18orf24* | 220134 | 0.10 | 0.04058 |
| cg01513611 | *NDUFA11* | 126328 | 0.10 | 0.04058 |
| cg01625242 | *GRP* | 2922 | -0.18 | 0.04058 |
| cg01797527 | *BMP2* | 650 | 0.10 | 0.04058 |
| cg02154956 | *PCCA* | 5095 | 0.08 | 0.04058 |
| cg02240226 | *GLB1L* | 79411 | 0.09 | 0.04058 |
| cg02889982 | *M-RIP* | 23164 | 0.36 | 0.04058 |
| cg03139377 | *SLC6A12* | 6539 | 0.29 | 0.04058 |
| cg03161453 | *BCOR* | 54880 | -0.32 | 0.04058 |
| cg03212129 | *MGC13096* | 84306 | 0.10 | 0.04058 |
| cg03214212 | *TSPAN13* | 27075 | 0.07 | 0.04058 |
| cg03251655 | *FLJ10260* | 55106 | -0.29 | 0.04058 |
| cg03491181 | *FKBP3* | 2287 | 0.10 | 0.04058 |
| cg03529436 | *ORF1-FL49* | 84418 | 0.09 | 0.04058 |
| cg03802231 | *FNTA* | 2339 | 0.09 | 0.04058 |
| cg03947362 | *FLJ37953* | 129450 | 0.12 | 0.04058 |
| cg04155026 | *TSNAXIP1* | 55815 | 0.08 | 0.04058 |
| cg04444771 | *C10orf10* | 11067 | 0.42 | 0.04058 |
| cg04518106 | *RPL26* | 6154 | 0.12 | 0.04058 |
| cg04541607 | *CRYBB1* | 1414 | 0.32 | 0.04058 |
| cg04619859 | *MGC5242* | 78996 | 0.10 | 0.04058 |
| cg04681849 | *PDGFRL* | 5157 | 0.07 | 0.04058 |
| cg04718306 | *GADD45A* | 1647 | 0.15 | 0.04058 |
| cg05342835 | *SYNC1* | 81493 | 0.31 | 0.04058 |
| cg05475904 | *BCL10* | 8915 | -0.45 | 0.04058 |
| cg05593780 | *PHF5A* | 84844 | 0.13 | 0.04058 |
| cg05641882 | *CXCL3* | 2921 | 0.10 | 0.04058 |
| cg05882522 | *EHD3* | 30845 | 0.09 | 0.04058 |
| cg06384026 | *PPFIA1* | 8500 | 0.11 | 0.04058 |
| cg06491116 | *LOC196264* | 196264 | 0.11 | 0.04058 |
| cg06505666 | *FLJ40629* | 150468 | 0.07 | 0.04058 |
| cg06808983 | *G6PC3* | 92579 | 0.10 | 0.04058 |
| cg07403255 | *PAX8* | 7849 | -0.37 | 0.04058 |
| cg07414384 | *BAZ1A* | 11177 | 0.11 | 0.04058 |
| cg07717632 | *FLJ20245* | 54863 | 0.13 | 0.04058 |
| cg08285151 | *HDAC9* | 9734 | 0.29 | 0.04058 |
| cg08313788 | *ROD1* | 9991 | 0.09 | 0.04058 |
| cg08570275 | *P2RY1* | 5028 | 0.10 | 0.04058 |
| cg08851943 | *C6orf64* | 55776 | 0.10 | 0.04058 |
| cg09160477 | *SUSD3* | 203328 | 0.13 | 0.04058 |
| cg09989134 | *C12orf44* | 60673 | 0.10 | 0.04058 |
| cg10218426 | *CKAP2* | 26586 | 0.09 | 0.04058 |
| cg10226546 | *NFATC2* | 4773 | 0.16 | 0.04058 |
| cg10266490 | *ACOT11* | 26027 | 0.46 | 0.04058 |
| cg10348863 | *ING2* | 3622 | 0.10 | 0.04058 |
| cg10404950 | *ALKBH8* | 91801 | 0.12 | 0.04058 |
| cg10591174 | *MYL2* | 4633 | 0.24 | 0.04058 |
| cg10645943 | *STRA13* | 201254 | 0.11 | 0.04058 |
| cg10784030 | *INPP5B* | 3633 | 0.35 | 0.04058 |
| cg11484872 | *TNF* | 7124 | 0.19 | 0.04058 |
| cg11792664 | *UQCR* | 10975 | 0.09 | 0.04058 |
| cg12078929 | *SERHL* | 94009 | 0.09 | 0.04058 |
| cg12167564 | *LYST* | 1130 | 0.23 | 0.04058 |
| cg12554857 | *PGDS* | 27306 | 0.45 | 0.04058 |
| cg13127386 | *SLC4A8* | 9498 | 0.10 | 0.04058 |
| cg13206017 | *SST* | 6750 | 0.14 | 0.04058 |
| cg13249256 | *MAEL* | 84944 | -0.48 | 0.04058 |
| cg13302154 | *MGP* | 4256 | 0.26 | 0.04058 |
| cg13625403 | *TRIM29* | 23650 | -0.63 | 0.04058 |
| cg13653456 | *VARS* | 7407 | 0.12 | 0.04058 |
| cg13726191 | *FGFBP1* | 9982 | 0.22 | 0.04058 |
| cg13739417 | *IL8RB* | 3579 | 0.46 | 0.04058 |
| cg13997068 | *TPSG1* | 25823 | 0.13 | 0.04058 |
| cg14196790 | *SLC22A5* | 6584 | 0.21 | 0.04058 |
| cg14341030 | *C12orf60* | 144608 | 0.08 | 0.04058 |
| cg15059511 | *DNAJC17* | 55192 | 0.12 | 0.04058 |
| cg15161785 | *ADI1* | 55256 | 0.08 | 0.04058 |
| cg15171237 | *CASP3* | 836 | -0.40 | 0.04058 |
| cg15230273 | *EFNA5* | 1946 | 0.11 | 0.04058 |
| cg15239703 | *CBR4* | 84869 | 0.11 | 0.04058 |
| cg15427774 | *GORASP1* | 64689 | 0.15 | 0.04058 |
| cg15446391 | *WT1* | 7490 | -0.48 | 0.04058 |
| cg15492003 | *SEC61B* | 10952 | 0.11 | 0.04058 |
| cg15640375 | *PRG2* | 79948 | 0.11 | 0.04058 |
| cg15779381 | *PFAAP5* | 10443 | 0.10 | 0.04058 |
| cg16547341 | *USP29* | 57663 | -0.29 | 0.04058 |
| cg16606638 | *GALNT4* | 8693 | 0.08 | 0.04058 |
| cg17489622 | *TMEM24* | 9854 | 0.11 | 0.04058 |
| cg18103150 | *C11orf47* | 283294 | -0.40 | 0.04058 |
| cg18240496 | *SCAMP1* | 9522 | 0.09 | 0.04058 |
| cg18287975 | *MEF2C* | 4208 | 0.08 | 0.04058 |
| cg18540871 | *FUCA2* | 2519 | 0.11 | 0.04058 |
| cg18577494 | *MGC22793* | 221908 | 0.10 | 0.04058 |
| cg18627308 | *FLJ10661* | 55199 | 0.15 | 0.04058 |
| cg18776056 | *FKBP4* | 2288 | 0.15 | 0.04058 |
| cg18862260 | *GDF8* | 2660 | -0.14 | 0.04058 |
| cg18942631 | *CASQ2* | 845 | 0.53 | 0.04058 |
| cg19287114 | *SLC44A1* | 23446 | 0.10 | 0.04058 |
| cg19497444 | *SLC22A18* | 5002 | -0.79 | 0.04058 |
| cg19679281 | *FANCL* | 55120 | 0.18 | 0.04058 |
| cg19728055 | *OXSR1* | 9943 | 0.11 | 0.04058 |
| cg19766460 | *C21orf128* | 150147 | 0.35 | 0.04058 |
| cg20033864 | *VAPA* | 9218 | 0.10 | 0.04058 |
| cg20315643 | *TAF1C* | 9013 | 0.09 | 0.04058 |
| cg20541456 | *CYFIP2* | 26999 | 0.43 | 0.04058 |
| cg20776317 | *SNAPC4* | 6621 | -0.17 | 0.04058 |
| cg20782689 | *OXA1L* | 5018 | -0.46 | 0.04058 |
| cg21650900 | *ARL4C* | 10123 | 0.13 | 0.04058 |
| cg21712678 | *ZC3H7B* | 23264 | 0.09 | 0.04058 |
| cg21746459 | *MRPL50* | 54534 | 0.09 | 0.04058 |
| cg21922731 | *HSD17B6* | 8630 | 0.28 | 0.04058 |
| cg22063056 | *CLCC1* | 23155 | 0.09 | 0.04058 |
| cg22226765 | *GGA3* | 23163 | 0.11 | 0.04058 |
| cg22253945 | *GPR45* | 11250 | -0.53 | 0.04058 |
| cg23076086 | *FYN* | 2534 | 0.13 | 0.04058 |
| cg23282771 | *ASS* | 445 | 0.10 | 0.04058 |
| cg23432345 | *HOXA7* | 3204 | 0.15 | 0.04058 |
| cg23704082 | *TRIM59* | 286827 | 0.11 | 0.04058 |
| cg24041453 | *CCDC11* | 220136 | 0.20 | 0.04058 |
| cg24127989 | *IMPDH1* | 3614 | 0.10 | 0.04058 |
| cg24216701 | *CDX1* | 1044 | -0.54 | 0.04058 |
| cg24302095 | *GRB10* | 2887 | 0.08 | 0.04058 |
| cg24471555 | *RAI16* | 64760 | -0.43 | 0.04058 |
| cg24580001 | *FLJ37970* | 283234 | -0.09 | 0.04058 |
| cg24824266 | *SV2B* | 9899 | 0.11 | 0.04058 |
| cg24975222 | *CDCP1* | 64866 | 0.09 | 0.04058 |
| cg24993443 | *SNRPN* | 6638 | -0.37 | 0.04058 |
| cg25011395 | *CNTROB* | 116840 | -0.37 | 0.04058 |
| cg25028542 | *ANLN* | 54443 | 0.09 | 0.04058 |
| cg25167838 | *SHH* | 6469 | 0.10 | 0.04058 |
| cg25396537 | *TERF1* | 7013 | 0.09 | 0.04058 |
| cg25978208 | *SNRPN* | 6638 | -0.37 | 0.04058 |
| cg26001030 | *RABGAP1L* | 9910 | 0.08 | 0.04058 |
| cg26277709 | *C9orf3* | 84909 | 0.08 | 0.04058 |
| cg26413827 | *DACH1* | 1602 | 0.15 | 0.04058 |
| cg26520371 | *M6PR* | 4074 | 0.07 | 0.04058 |
| cg26782833 | *MGC4268* | 83607 | -0.42 | 0.04058 |
| cg26831415 | *EFHA2* | 286097 | 0.08 | 0.04058 |
| cg26894575 | *S100A4* | 6275 | 0.46 | 0.04058 |
| cg27072387 | *LTBP1* | 4052 | 0.10 | 0.04058 |
| cg27657283 | *LEPRE1* | 64175 | 0.10 | 0.04058 |
| cg05164634 | *WNT10B* | 7480 | 0.40 | 0.04058 |
| cg10238818 | *CYYR1* | 116159 | 0.10 | 0.04058 |
| cg10124201 | *BOLL* | 66037 | -0.31 | 0.04059 |
| cg10851775 | *SLC26A7* | 115111 | -0.21 | 0.04059 |
| cg17414431 | *PPFIA3* | 8541 | 0.16 | 0.04059 |
| cg21432513 | *CHFR* | 55743 | -0.09 | 0.04059 |
| cg23452458 | *OSBPL7* | 114881 | 0.46 | 0.04059 |
| cg27094076 | *ZPBP* | 11055 | 0.17 | 0.04059 |
| cg27402949 | *FLJ20097* | 55610 | 0.10 | 0.04059 |
| cg02240622 | *PLCB2* | 5330 | 0.38 | 0.04059 |
| cg04240200 | *ATRN* | 8455 | 0.11 | 0.04059 |
| cg13918581 | *ANKRD11* | 29123 | 0.15 | 0.04059 |
| cg15586352 | *PSG5* | 5673 | 0.60 | 0.04059 |
| cg17526300 | *PTER* | 9317 | 0.11 | 0.04059 |
| cg18516268 | *RPL23* | 9349 | -0.37 | 0.04059 |
| cg26294850 | *TSPAN1* | 10103 | 0.41 | 0.04059 |
| cg26399035 | *THUMPD1* | 55623 | -0.62 | 0.04059 |
| cg12627583 | *AOX1* | 316 | 0.10 | 0.04060 |
| cg03905094 | *DEPDC1B* | 55789 | 0.07 | 0.04060 |
| cg06297145 | *NFXL1* | 152518 | 0.10 | 0.04060 |
| cg17341358 | *MRPL1* | 65008 | 0.09 | 0.04060 |
| cg12371177 | *PLEKHG6* | 55200 | -0.46 | 0.04061 |
| cg24765079 | *CDH1* | 999 | -0.39 | 0.04061 |
| cg04557383 | *MT1H* | 4496 | 0.10 | 0.04062 |
| cg25775449 | *LTB4R* | 1241 | 0.19 | 0.04062 |
| cg27457941 | *RBP1* | 5947 | 0.12 | 0.04062 |
| cg00677811 | *CCM2* | 83605 | 0.15 | 0.04064 |
| cg10673984 | *PPM1D* | 8493 | 0.08 | 0.04065 |
| cg00095674 | *PLEKHO1* | 51177 | 0.16 | 0.04066 |
| cg00686022 | *C12orf40* | 283461 | -0.48 | 0.04066 |
| cg01251255 | *NSUN5* | 55695 | 0.10 | 0.04066 |
| cg01593190 | *ADAMTS2* | 9509 | 0.13 | 0.04066 |
| cg03700308 | *MOCS3* | 27304 | 0.11 | 0.04066 |
| cg03960217 | *LCE2C* | 353140 | 0.39 | 0.04066 |
| cg04549333 | *ALX4* | 60529 | 0.11 | 0.04066 |
| cg04790874 | *CD79A* | 973 | 0.41 | 0.04066 |
| cg05290904 | *RBBP5* | 5929 | 0.11 | 0.04066 |
| cg05368341 | *SYT6* | 148281 | 0.12 | 0.04066 |
| cg05413282 | *PRSS3* | 5646 | -0.10 | 0.04066 |
| cg06324094 | *JTB* | 10899 | 0.12 | 0.04066 |
| cg07533148 | *TRIM58* | 25893 | 0.10 | 0.04066 |
| cg08665772 | *KCNJ9* | 3765 | 0.25 | 0.04066 |
| cg08731300 | *FZD8* | 8325 | 0.10 | 0.04066 |
| cg09546345 | *DHRS1* | 115817 | 0.09 | 0.04066 |
| cg10713320 | *CLDND1* | 56650 | 0.07 | 0.04066 |
| cg10962407 | *ALS2CR15* | 130026 | 0.09 | 0.04066 |
| cg11233228 | *CDC26* | 246184 | 0.09 | 0.04066 |
| cg11258532 | *CA7* | 766 | -0.36 | 0.04066 |
| cg11649001 | *KIAA0082* | 23070 | 0.12 | 0.04066 |
| cg11840168 | *RBBP8* | 5932 | 0.08 | 0.04066 |
| cg12376277 | *TUG1* | 55000 | 0.12 | 0.04066 |
| cg13823791 | *COPS3* | 8533 | 0.11 | 0.04066 |
| cg15980408 | *TMEM22* | 80723 | 0.10 | 0.04066 |
| cg16370737 | *SYP* | 6855 | 0.18 | 0.04066 |
| cg16666160 | *NR5A1* | 2516 | -0.08 | 0.04066 |
| cg17145652 | *HOOK3* | 84376 | -0.37 | 0.04066 |
| cg18333807 | *GSK3A* | 2931 | 0.13 | 0.04066 |
| cg18752880 | *C1QTNF3* | 114899 | 0.37 | 0.04066 |
| cg19168338 | *CORO7* | 79585 | 0.08 | 0.04066 |
| cg19382714 | *MOG* | 4340 | -0.11 | 0.04066 |
| cg20441175 | *DNAJC9* | 23234 | 0.09 | 0.04066 |
| cg21368354 | *FLJ10769* | 55739 | 0.09 | 0.04066 |
| cg22155248 | *TAPBPL* | 55080 | 0.10 | 0.04066 |
| cg22983529 | *GNG11* | 2791 | 0.07 | 0.04066 |
| cg23526055 | *TCEB1* | 6921 | 0.11 | 0.04066 |
| cg26293512 | *TEPP* | 374739 | 0.16 | 0.04066 |
| cg26651830 | *BCAP29* | 55973 | 0.13 | 0.04066 |
| cg26926521 | *ADRA2A* | 150 | 0.09 | 0.04066 |
| cg06200013 | *ATP1B2* | 482 | 0.12 | 0.04067 |
| cg16385933 | *PDCD4* | 27250 | 0.12 | 0.04067 |
| cg23732024 | *LY96* | 23643 | 0.38 | 0.04067 |
| cg01462829 | *HS3ST1* | 9957 | 0.08 | 0.04067 |
| cg02661879 | *FLNC* | 2318 | 0.11 | 0.04067 |
| cg05159732 | *HCRT* | 3060 | -0.25 | 0.04067 |
| cg06590711 | *CPXCR1* | 53336 | -0.53 | 0.04067 |
| cg08315277 | *PKDREJ* | 10343 | -0.54 | 0.04067 |
| cg21099221 | *SRM* | 6723 | 0.13 | 0.04067 |
| cg22367264 | *FLJ90579* | 283310 | 0.31 | 0.04067 |
| cg27665659 | *AP1S1* | 1174 | 0.09 | 0.04067 |
| cg08132767 | *ARL1* | 400 | 0.09 | 0.04068 |
| cg01957330 | *SCAP2* | 8935 | 0.12 | 0.04068 |
| cg07190917 | *STX4A* | 6810 | 0.11 | 0.04069 |
| cg24448231 | *ZNFX1* | 57169 | 0.09 | 0.04069 |
| cg09296212 | *C9orf90* | 203245 | -0.38 | 0.04069 |
| cg11397854 | *IQSEC1* | 9922 | 0.11 | 0.04070 |
| cg22716127 | *SMAP1* | 60682 | 0.08 | 0.04070 |
| cg09515778 | *CDK5* | 1020 | 0.12 | 0.04071 |
| cg25514304 | *PSEN2* | 5664 | -0.42 | 0.04071 |
| cg01556075 | *MCEMP1* | 199675 | 0.46 | 0.04071 |
| cg01731341 | *FGF6* | 2251 | -0.43 | 0.04071 |
| cg02556718 | *SEZ6L2* | 26470 | 0.13 | 0.04071 |
| cg06263943 | *CACNA1H* | 8912 | -0.37 | 0.04071 |
| cg07496861 | *LOC151194* | 151194 | 0.08 | 0.04071 |
| cg08420085 | *ZDHHC24* | 254359 | 0.08 | 0.04071 |
| cg10715231 | *SOX30* | 11063 | -0.44 | 0.04071 |
| cg11141561 | *CAMLG* | 819 | 0.10 | 0.04071 |
| cg11200929 | *CRABP1* | 1381 | 0.10 | 0.04071 |
| cg12274479 | *HSPG2* | 3339 | -0.35 | 0.04071 |
| cg12738007 | *MRPL16* | 54948 | 0.11 | 0.04071 |
| cg14377791 | *LOC400696* | 400696 | 0.21 | 0.04071 |
| cg15111734 | *C10orf13* | 143282 | 0.08 | 0.04071 |
| cg16725130 | *MMP19* | 4327 | 0.26 | 0.04071 |
| cg17451138 | *C12orf38* | 79867 | 0.08 | 0.04071 |
| cg18389752 | *AMOT* | 154796 | 0.15 | 0.04071 |
| cg19556572 | *AKNA* | 80709 | 0.17 | 0.04071 |
| cg22763181 | *YY1* | 7528 | 0.10 | 0.04071 |
| cg23442323 | *CD109* | 135228 | 0.08 | 0.04071 |
| cg25307081 | *BRD7* | 29117 | -0.25 | 0.04071 |
| cg01909833 | *BRD2* | 6046 | 0.13 | 0.04072 |
| cg15037004 | *ZNF366* | 167465 | 0.20 | 0.04073 |
| cg18463686 | *CLEC5A* | 23601 | 0.42 | 0.04073 |
| cg02788102 | *IGSF4* | 23705 | 0.10 | 0.04073 |
| cg03127334 | *ERG* | 2078 | 0.15 | 0.04073 |
| cg06954481 | *GBX2* | 2637 | 0.15 | 0.04073 |
| cg07634204 | *VPS72* | 6944 | 0.13 | 0.04073 |
| cg08290628 | *CORO2B* | 10391 | 0.19 | 0.04073 |
| cg08781448 | *GALNS* | 2588 | 0.11 | 0.04073 |
| cg10162464 | *ZNF282* | 8427 | 0.09 | 0.04073 |
| cg14590817 | *ADARB2* | 105 | 0.08 | 0.04073 |
| cg14834436 | *MNAT1* | 4331 | 0.10 | 0.04073 |
| cg18542098 | *FLJ36445* | 163183 | -0.20 | 0.04073 |
| cg19217872 | *ZNF187* | 7741 | 0.09 | 0.04073 |
| cg24528523 | *SLC22A18* | 5002 | 0.18 | 0.04073 |
| cg25226014 | *CXCR6* | 10663 | 0.19 | 0.04073 |
| cg26475085 | *OSMR* | 9180 | 0.08 | 0.04073 |
| cg08256027 | *HYMAI* | 57061 | 0.32 | 0.04073 |
| cg08468689 | *LGP1* | 84514 | 0.35 | 0.04073 |
| cg00888561 | *WDFY2* | 115825 | 0.09 | 0.04079 |
| cg09037858 | *POLR3D* | 661 | 0.10 | 0.04080 |
| cg10660136 | *MGC43122* | 151477 | 0.10 | 0.04080 |
| cg00474004 | *IFNA14* | 3448 | -0.16 | 0.04082 |
| cg03464689 | *NFKB1* | 4790 | 0.14 | 0.04082 |
| cg08290212 | *GNG3* | 2785 | 0.12 | 0.04082 |
| cg14614901 | *LOC401498* | 401498 | 0.14 | 0.04082 |
| cg14915263 | *PTPN1* | 5770 | 0.09 | 0.04082 |
| cg15387123 | *CLIC3* | 9022 | 0.57 | 0.04082 |
| cg19123863 | *JUNB* | 3726 | 0.10 | 0.04082 |
| cg22140675 | *G6PC3* | 92579 | 0.14 | 0.04082 |
| cg25574548 | *C20orf45* | 51012 | 0.08 | 0.04082 |
| cg25799433 | *SIRPB1* | 10326 | 0.36 | 0.04082 |
| cg19857538 | *TRUB2* | 26995 | 0.07 | 0.04082 |
| cg22630160 | *HIST1H2AB* | 8335 | 0.14 | 0.04083 |
| cg25971347 | *FOXF1* | 2294 | 0.10 | 0.04083 |
| cg07197059 | *EFS* | 10278 | -0.41 | 0.04084 |
| cg17846100 | *MT* | 27349 | 0.09 | 0.04084 |
| cg20091959 | *L3MBTL* | 26013 | -0.37 | 0.04084 |
| cg00293409 | *LRRC33* | 375387 | 0.12 | 0.04085 |
| cg05961595 | *APEX2* | 27301 | 0.11 | 0.04085 |
| cg10203483 | *LOC284948* | 284948 | -0.36 | 0.04085 |
| cg22324153 | *SLC35D2* | 11046 | -0.35 | 0.04085 |
| cg10417559 | *LMO6* | 4007 | -0.45 | 0.04086 |
| cg10839521 | *FLJ32786* | 136332 | 0.11 | 0.04086 |
| cg13473336 | *SLC5A2* | 6524 | 0.20 | 0.04086 |
| cg00040861 | *LSM7* | 51690 | 0.09 | 0.04086 |
| cg24549507 | *C6orf145* | 221749 | 0.09 | 0.04086 |
| cg12825252 | *FLJ14397* | 84865 | 0.13 | 0.04087 |
| cg02994216 | *RALB* | 5899 | 0.09 | 0.04087 |
| cg09533063 | *ALDH8A1* | 64577 | 0.20 | 0.04087 |
| cg17889103 | *UQCRB* | 7381 | 0.09 | 0.04088 |
| cg13736875 | *UBE2C* | 11065 | 0.12 | 0.04089 |
| cg13850625 | *FLJ11155* | 55314 | 0.09 | 0.04089 |
| cg20161179 | *MSX1* | 4487 | 0.10 | 0.04089 |
| cg20851097 | *SPTB* | 6710 | 0.40 | 0.04089 |
| cg00859655 | *CYB5R2* | 51700 | -0.48 | 0.04090 |
| cg02310296 | *MMP9* | 4318 | 0.47 | 0.04090 |
| cg03298413 | *CREBL1* | 1388 | 0.10 | 0.04090 |
| cg03536787 | *NSD1* | 64324 | 0.09 | 0.04090 |
| cg03574571 | *CD22* | 933 | 0.40 | 0.04090 |
| cg04141379 | *SPATA6* | 54558 | 0.08 | 0.04090 |
| cg04394967 | *RDH12* | 145226 | 0.43 | 0.04090 |
| cg04499381 | *CXorf9* | 54440 | 0.46 | 0.04090 |
| cg04636557 | *C1orf124* | 83932 | -0.33 | 0.04090 |
| cg05492845 | *VEGFB* | 7423 | 0.11 | 0.04090 |
| cg06211871 | *ZNF684* | 127396 | 0.06 | 0.04090 |
| cg06268694 | *CELSR1* | 9620 | 0.14 | 0.04090 |
| cg06855803 | *ICAM3* | 3385 | 0.47 | 0.04090 |
| cg07581973 | *PRDX4* | 10549 | 0.13 | 0.04090 |
| cg10318443 | *RNMTL1* | 55178 | 0.09 | 0.04090 |
| cg10845200 | *DRD1IP* | 50632 | 0.10 | 0.04090 |
| cg11208235 | *BCAR3* | 8412 | 0.13 | 0.04090 |
| cg12202913 | *MGC2803* | 79002 | -0.36 | 0.04090 |
| cg12806381 | *PIGV* | 55650 | 0.07 | 0.04090 |
| cg13058581 | *PRDM10* | 56980 | 0.11 | 0.04090 |
| cg14519000 | *GATA5* | 140628 | -0.09 | 0.04090 |
| cg15198335 | *MGC4562* | 115752 | 0.11 | 0.04090 |
| cg15225105 | *SAC3D1* | 29901 | 0.13 | 0.04090 |
| cg15521097 | *ITIH5L* | 347365 | -0.15 | 0.04090 |
| cg15765532 | *C11orf30* | 56946 | 0.10 | 0.04090 |
| cg15836722 | *IL1B* | 3553 | 0.33 | 0.04090 |
| cg15844609 | *YIF1B* | 90522 | 0.14 | 0.04090 |
| cg15880738 | *CD3G* | 917 | -0.09 | 0.04090 |
| cg17140815 | *ATP6V1F* | 9296 | 0.11 | 0.04090 |
| cg17173423 | *MS4A3* | 932 | 0.36 | 0.04090 |
| cg18044482 | *PSMD10* | 5716 | 0.11 | 0.04090 |
| cg18714560 | *SCT* | 6343 | -0.43 | 0.04090 |
| cg20218060 | *CLK1* | 1195 | 0.09 | 0.04090 |
| cg21475255 | *DAG1* | 1605 | 0.09 | 0.04090 |
| cg24012034 | *ETNK1* | 55500 | 0.12 | 0.04090 |
| cg24334879 | *NKTR* | 4820 | 0.09 | 0.04090 |
| cg24713204 | *ZNF471* | 57573 | 0.09 | 0.04090 |
| cg25013852 | *PGM1* | 5236 | 0.11 | 0.04090 |
| cg25033972 | *RIPK4* | 54101 | 0.14 | 0.04090 |
| cg25547939 | *VEGFB* | 7423 | -0.22 | 0.04090 |
| cg26408003 | *C14orf94* | 54930 | -0.46 | 0.04090 |
| cg27212359 | *FRAT2* | 23401 | 0.10 | 0.04090 |
| cg06353948 | *SETX* | 23064 | -0.57 | 0.04091 |
| cg20317180 | *ZNF524* | 147807 | 0.08 | 0.04091 |
| cg05678175 | *TRIM28* | 10155 | 0.10 | 0.04093 |
| cg05940536 | *ACVR1B* | 91 | 0.14 | 0.04093 |
| cg09039698 | *FLJ90652* | 283899 | 0.09 | 0.04093 |
| cg11295902 | *SF1* | 7536 | 0.09 | 0.04093 |
| cg13234643 | *CTSL2* | 1515 | 0.08 | 0.04093 |
| cg16612562 | *RRP22* | 10633 | -0.37 | 0.04093 |
| cg17243643 | *RDH5* | 5959 | -0.10 | 0.04093 |
| cg18729973 | *TFF1* | 7031 | 0.48 | 0.04093 |
| cg18931815 | *COL8A2* | 1296 | 0.30 | 0.04093 |
| cg21452443 | *NMT1* | 4836 | 0.07 | 0.04093 |
| cg22617367 | *NKRF* | 55922 | 0.14 | 0.04093 |
| cg24341536 | *SQSTM1* | 8878 | 0.12 | 0.04093 |
| cg02760007 | *SRF* | 6722 | 0.12 | 0.04094 |
| cg04063348 | *IL17F* | 112744 | -0.20 | 0.04094 |
| cg04733388 | *UBE1DC1* | 79876 | 0.10 | 0.04094 |
| cg04970994 | *ZNF142* | 7701 | -0.10 | 0.04094 |
| cg11706111 | *ZNF19* | 7567 | -0.47 | 0.04094 |
| cg16452396 | *PHF6* | 84295 | 0.10 | 0.04094 |
| cg20999934 | *NFX1* | 4799 | 0.07 | 0.04094 |
| cg21685565 | *GNA12* | 2768 | 0.11 | 0.04094 |
| cg23568913 | *PES1* | 23481 | 0.08 | 0.04094 |
| cg26625319 | *BRRN1* | 23397 | 0.09 | 0.04094 |
| cg27269921 | *MN1* | 4330 | 0.11 | 0.04094 |
| cg27586455 | *ZNF688* | 146542 | 0.12 | 0.04094 |
| cg05508048 | *DDX26* | 26512 | 0.09 | 0.04094 |
| cg07857181 | *POLH* | 5429 | 0.13 | 0.04096 |
| cg15746620 | *EMR3* | 84658 | 0.42 | 0.04096 |
| cg21825027 | *SH3YL1* | 26751 | 0.11 | 0.04096 |
| cg14479329 | *RNASET2* | 8635 | 0.16 | 0.04096 |
| cg18674980 | *CA3* | 761 | 0.09 | 0.04096 |
| cg06940792 | *MEGF10* | 84466 | 0.09 | 0.04097 |
| cg13235447 | *IFNA2* | 3440 | 0.25 | 0.04097 |
| cg18211447 | *RAGE* | 5891 | 0.07 | 0.04097 |
| cg02972551 | *JMJD1A* | 55818 | 0.07 | 0.04097 |
| cg03293770 | *FLJ20487* | 54949 | 0.11 | 0.04097 |
| cg06347996 | *DPYD* | 1806 | 0.07 | 0.04097 |
| cg10780112 | *HEBP2* | 23593 | 0.11 | 0.04097 |
| cg15936718 | *GAS8* | 2622 | 0.12 | 0.04097 |
| cg19011603 | *RP11-311P8.3* | 139596 | 0.17 | 0.04097 |
| cg03606258 | *GNAS* | 2778 | -0.42 | 0.04099 |
| cg05419984 | *PIGX* | 54965 | 0.14 | 0.04099 |
| cg06132342 | *KRTHB5* | 3891 | -0.32 | 0.04099 |
| cg08771429 | *KRIT1* | 889 | 0.12 | 0.04099 |
| cg10367730 | *PRDX3* | 10935 | 0.13 | 0.04099 |
| cg12867448 | *ODF2* | 4957 | -0.47 | 0.04099 |
| cg14924091 | *D4ST1* | 113189 | 0.08 | 0.04099 |
| cg15883761 | *MUS81* | 80198 | 0.06 | 0.04099 |
| cg16348491 | *MRLC2* | 103910 | 0.08 | 0.04099 |
| cg19423311 | *IL21R* | 50615 | 0.42 | 0.04099 |
| cg19853703 | *APOL6* | 80830 | 0.41 | 0.04099 |
| cg24276491 | *C14orf11* | 55837 | -0.43 | 0.04099 |
| cg25261329 | *RAD51AP1* | 10635 | 0.08 | 0.04099 |
| cg25479216 | *NANS* | 54187 | 0.12 | 0.04099 |
| cg26295990 | *FLJ23436* | 79724 | 0.09 | 0.04099 |
| cg21789545 | *COL9A1* | 1297 | 0.20 | 0.04099 |
| cg12465398 | *PPIL3* | 53938 | 0.12 | 0.04100 |
| cg17676096 | *LEO1* | 123169 | 0.11 | 0.04100 |
| cg06634267 | *C6orf151* | 154007 | 0.10 | 0.04100 |
| cg21111471 | *FLRT1* | 23769 | 0.09 | 0.04100 |
| cg21443584 | *MBIP* | 51562 | 0.08 | 0.04100 |
| cg09527021 | *TADA1L* | 117143 | 0.09 | 0.04100 |
| cg15105081 | *PTD015* | 28971 | 0.13 | 0.04100 |
| cg15965510 | *BTAF1* | 9044 | 0.10 | 0.04100 |
| cg16750914 | *KIAA1212* | 55704 | 0.11 | 0.04100 |
| cg20512303 | *PDLIM4* | 8572 | -0.41 | 0.04100 |
| cg22524267 | *STX18* | 53407 | 0.09 | 0.04100 |
| cg25662680 | *NUTF2* | 10204 | 0.10 | 0.04100 |
| cg12340144 | *XYLB* | 9942 | -0.38 | 0.04100 |
| cg26872564 | *ZNF185* | 7739 | 0.29 | 0.04100 |
| cg19042950 | *IGF2BP3* | 10643 | 0.12 | 0.04102 |
| cg27299588 | *PTPRS* | 5802 | 0.09 | 0.04102 |
| cg23087130 | *ABI1* | 10006 | 0.14 | 0.04104 |
| cg05244766 | *GSTP1* | 2950 | -0.43 | 0.04104 |
| cg11432797 | *SPN* | 6693 | 0.38 | 0.04104 |
| cg21750887 | *CPA6* | 57094 | 0.24 | 0.04104 |
| cg25128803 | *SLC16A1* | 6566 | 0.10 | 0.04104 |
| cg06090864 | *FFAR1* | 2864 | -0.69 | 0.04105 |
| cg07073964 | *PRSSL1* | 400668 | 0.36 | 0.04105 |
| cg13752043 | *CCBL1* | 883 | 0.10 | 0.04105 |
| cg22325715 | *CNTD* | 124817 | -0.49 | 0.04105 |
| cg23217005 | *MT1F* | 4494 | 0.11 | 0.04105 |
| cg24043192 | *FLJ32363* | 375444 | 0.12 | 0.04105 |
| cg27121132 | *DERL1* | 79139 | 0.09 | 0.04105 |
| cg02259997 | *FGF9* | 2254 | 0.07 | 0.04105 |
| cg01785568 | *MSX1* | 4487 | -0.37 | 0.04106 |
| cg01816336 | *MINPP1* | 9562 | 0.10 | 0.04106 |
| cg06150803 | *TNFRSF19* | 55504 | 0.28 | 0.04106 |
| cg06537230 | *DLX5* | 1749 | -0.50 | 0.04106 |
| cg06838394 | *ARFGEF2* | 10564 | 0.11 | 0.04106 |
| cg10073875 | *GEMIN7* | 79760 | 0.11 | 0.04106 |
| cg10227191 | *KRT18* | 3875 | 0.10 | 0.04106 |
| cg16794633 | *C3orf58* | 205428 | 0.11 | 0.04106 |
| cg17894008 | *NACAL* | 342538 | -0.47 | 0.04106 |
| cg19441691 | *MAOA* | 4128 | 0.10 | 0.04106 |
| cg20282199 | *SEC13L1* | 6396 | 0.11 | 0.04106 |
| cg20957193 | *ZNRF1* | 84937 | 0.09 | 0.04106 |
| cg21332946 | *LIX1L* | 128077 | 0.08 | 0.04106 |
| cg24997562 | *OASL* | 8638 | 0.44 | 0.04106 |
| cg00427635 | *TBC1D21* | 161514 | 0.25 | 0.04107 |
| cg00672638 | *ARHGAP26* | 23092 | 0.05 | 0.04107 |
| cg02689825 | *PCNA* | 5111 | 0.10 | 0.04107 |
| cg06317209 | *AVIL* | 10677 | 0.35 | 0.04107 |
| cg18715868 | *HIST1H2BN* | 8341 | 0.14 | 0.04107 |
| cg24812167 | *SNAPC4* | 6621 | -0.23 | 0.04107 |
| cg15272684 | *KIAA0319L* | 79932 | -0.55 | 0.04108 |
| cg02283643 | *SULF1* | 23213 | 0.52 | 0.04110 |
| cg12004730 | *TAX1BP3* | 30851 | 0.10 | 0.04110 |
| cg12906740 | *NUDT15* | 55270 | 0.08 | 0.04110 |
| cg20899053 | *HLXB9* | 3110 | 0.13 | 0.04110 |
| cg00687674 | *TMEM84* | 283673 | 0.38 | 0.04110 |
| cg11249173 | *HERV-FRD* | 405754 | 0.47 | 0.04110 |
| cg12153542 | *REM1* | 28954 | 0.09 | 0.04110 |
| cg23393291 | *LINS1* | 55180 | 0.12 | 0.04110 |
| cg27416067 | *IL12RB2* | 3595 | 0.15 | 0.04110 |
| cg06752594 | *PSRC1* | 84722 | 0.08 | 0.04111 |
| cg09547190 | *C9orf89* | 84270 | -0.31 | 0.04111 |
| cg24102360 | *ZNF333* | 84449 | 0.14 | 0.04111 |
| cg03251079 | *HMGA2* | 8091 | 0.08 | 0.04111 |
| cg11038081 | *ZNF235* | 9310 | -0.51 | 0.04111 |
| cg12239750 | *ALDH18A1* | 5832 | 0.07 | 0.04111 |
| cg01911082 | *CSMD3* | 114788 | 0.09 | 0.04112 |
| cg09580336 | *ATP1A1* | 476 | 0.09 | 0.04112 |
| cg03340261 | *HBS1L* | 10767 | 0.08 | 0.04112 |
| cg07845390 | *KCNT2* | 343450 | 0.07 | 0.04112 |
| cg10897648 | *MALT1* | 10892 | 0.11 | 0.04112 |
| cg17726022 | *SLC38A1* | 81539 | 0.11 | 0.04112 |
| cg17801256 | *ZMYM4* | 9202 | 0.12 | 0.04112 |
| cg18470891 | *FOXM1* | 2305 | 0.07 | 0.04112 |
| cg22956254 | *GDF3* | 9573 | 0.35 | 0.04112 |
| cg25674286 | *ADORA3* | 140 | 0.45 | 0.04112 |
| cg26060255 | *MRPS26* | 64949 | 0.13 | 0.04112 |
| cg01331554 | *G3BP2* | 9908 | 0.07 | 0.04112 |
| cg04014889 | *MAGEL2* | 54551 | -0.38 | 0.04112 |
| cg06131859 | *KYNU* | 8942 | 0.24 | 0.04112 |
| cg06869899 | *RBKS* | 64080 | 0.10 | 0.04112 |
| cg10720654 |  | 11191 | 0.12 | 0.04112 |
| cg16703647 | *CRABP1* | 1381 | 0.12 | 0.04112 |
| cg17289734 | *SEMA3B* | 7869 | 0.09 | 0.04112 |
| cg17998964 | *MARK2* | 2011 | 0.41 | 0.04112 |
| cg19949137 | *HTATSF1* | 27336 | 0.10 | 0.04112 |
| cg20988728 | *MGC17330* | 113791 | 0.08 | 0.04112 |
| cg21743649 | *NUP98* | 4928 | -0.47 | 0.04112 |
| cg22269795 | *EFHD2* | 79180 | 0.10 | 0.04112 |
| cg22660544 | *RPS11* | 6205 | 0.09 | 0.04112 |
| cg24063470 | *FOXC2* | 2303 | 0.14 | 0.04112 |
| cg26815229 | *CYP2J2* | 1573 | 0.13 | 0.04112 |
| cg26829318 | *GLRX* | 2745 | 0.09 | 0.04112 |
| cg02277509 | *TIMM9* | 26520 | 0.09 | 0.04112 |
| cg09086179 | *NSBP1* | 79366 | 0.12 | 0.04112 |
| cg17100200 | *GUCA2B* | 2981 | -0.07 | 0.04112 |
| cg21614638 | *DAPP1* | 27071 | 0.37 | 0.04116 |
| cg00635481 | *ZNF76* | 7629 | 0.08 | 0.04117 |
| cg04835638 | *FHIT* | 2272 | -0.43 | 0.04117 |
| cg13656360 | *MLL* | 4297 | 0.08 | 0.04117 |
| cg13795840 | *C19orf24* | 55009 | -0.44 | 0.04117 |
| cg19324627 | *APOA1* | 335 | -0.10 | 0.04117 |
| cg25788012 | *EXOSC4* | 54512 | 0.13 | 0.04117 |
| cg13669465 | *EXOSC1* | 51013 | 0.10 | 0.04117 |
| cg04736140 | *GPR161* | 23432 | 0.12 | 0.04118 |
| cg15078479 | *PARP2* | 10038 | 0.09 | 0.04118 |
| cg05073845 | *FOXO1A* | 2308 | 0.09 | 0.04118 |
| cg16342780 | *PLXNB3* | 5365 | -0.10 | 0.04118 |
| cg21389013 | *PPM1G* | 5496 | 0.10 | 0.04118 |
| cg07560096 | *ME3* | 10873 | 0.09 | 0.04119 |
| cg15628498 | *ONECUT1* | 3175 | 0.08 | 0.04119 |
| cg17237813 | *TTLL7* | 79739 | -0.33 | 0.04119 |
| cg27214652 | *PPP2R5E* | 5529 | 0.10 | 0.04119 |
| cg24608308 | *RAPGEF3* | 10411 | 0.10 | 0.04121 |
| cg11676599 | *PLEKHA1* | 59338 | 0.10 | 0.04122 |
| cg12737801 | *TCEA1* | 6917 | 0.10 | 0.04122 |
| cg06609475 | *NDUFS6* | 4726 | 0.09 | 0.04122 |
| cg14569415 | *GMPPA* | 29926 | 0.09 | 0.04122 |
| cg15977816 | *KCNN4* | 3783 | 0.33 | 0.04122 |
| cg19712821 | *KSP37* | 83888 | 0.36 | 0.04122 |
| cg14799446 | *STRN4* | 29888 | -0.32 | 0.04122 |
| cg13386234 | *DAD1* | 1603 | 0.08 | 0.04124 |
| cg20398399 | *KLHL6* | 89857 | 0.46 | 0.04124 |
| cg24522076 | *MLLT7* | 4303 | 0.08 | 0.04124 |
| cg16666115 | *MRPS18A* | 55168 | 0.13 | 0.04124 |
| cg03673687 | *EML1* | 2009 | 0.08 | 0.04125 |
| cg05494069 | *PRKAA2* | 5563 | 0.09 | 0.04125 |
| cg05671018 | *LYSMD2* | 256586 | -0.33 | 0.04125 |
| cg08788717 | *STK33* | 65975 | 0.10 | 0.04125 |
| cg10300684 | *FOXG1B* | 2290 | 0.10 | 0.04125 |
| cg14063008 | *DAB2IP* | 153090 | 0.12 | 0.04125 |
| cg19815928 | *ATP6V0D1* | 9114 | 0.12 | 0.04125 |
| cg12111697 | *ZNF555* | 148254 | 0.10 | 0.04125 |
| cg18847227 | *SUMF1* | 285362 | -0.35 | 0.04125 |
| cg26778754 | *SSFA2* | 6744 | 0.12 | 0.04125 |
| cg00519627 | *CORO7* | 79585 | 0.09 | 0.04125 |
| cg00756058 | *DZIP1* | 22873 | 0.11 | 0.04125 |
| cg01765641 | *TBC1D5* | 9779 | -0.40 | 0.04125 |
| cg01835489 | *KRT8* | 3856 | -0.24 | 0.04125 |
| cg01938354 | *KIAA0423* | 23116 | 0.10 | 0.04125 |
| cg01994779 | *ZNRF1* | 84937 | 0.12 | 0.04125 |
| cg02433905 | *ATP6V1A* | 523 | 0.14 | 0.04125 |
| cg02549424 | *AIFL* | 150209 | 0.14 | 0.04125 |
| cg02735133 | *DCTN5* | 84516 | 0.11 | 0.04125 |
| cg02854090 | *HIST1H2AA* | 221613 | -0.31 | 0.04125 |
| cg03042149 | *PDE8A* | 5151 | 0.11 | 0.04125 |
| cg03361068 | *TRIM9* | 114088 | 0.09 | 0.04125 |
| cg03623878 | *MCF2L* | 23263 | 0.48 | 0.04125 |
| cg03950009 | *STX17* | 55014 | 0.09 | 0.04125 |
| cg05140736 | *SGSH* | 6448 | -0.38 | 0.04125 |
| cg05228408 | *CLCN6* | 1185 | -0.46 | 0.04125 |
| cg05291178 | *C3orf24* | 115795 | -0.28 | 0.04125 |
| cg05576974 | *UCHL5* | 51377 | 0.10 | 0.04125 |
| cg06429195 | *DAZL* | 1618 | -0.45 | 0.04125 |
| cg07326074 | *FLJ13909* | 80178 | 0.07 | 0.04125 |
| cg07443954 | *CA11* | 770 | 0.11 | 0.04125 |
| cg07520158 | *KIAA0241* | 23080 | 0.11 | 0.04125 |
| cg07952391 | *FLJ10916* | 55258 | 0.09 | 0.04125 |
| cg08417687 | *SNX3* | 8724 | 0.12 | 0.04125 |
| cg08419026 | *SLCO5A1* | 81796 | 0.09 | 0.04125 |
| cg08687163 | *MST1R* | 4486 | -0.46 | 0.04125 |
| cg09138627 | *TNRC15* | 26058 | 0.10 | 0.04125 |
| cg09279263 | *TADA3L* | 10474 | 0.18 | 0.04125 |
| cg09299388 | *PGK2* | 5232 | -0.40 | 0.04125 |
| cg09384159 | *TUBE1* | 51175 | 0.12 | 0.04125 |
| cg09456329 | *MGC39581* | 257062 | 0.10 | 0.04125 |
| cg10137155 | *KBTBD8* | 84541 | 0.13 | 0.04125 |
| cg10503138 | *CNTN4* | 152330 | -0.32 | 0.04125 |
| cg10792326 | *DNCL1* | 8655 | 0.08 | 0.04125 |
| cg11452221 | *GEFT* | 115557 | 0.09 | 0.04125 |
| cg11759378 | *DPPA2* | 151871 | -0.35 | 0.04125 |
| cg12024906 | *HKR1* | 284459 | -0.34 | 0.04125 |
| cg12845808 | *PCDH12* | 51294 | 0.36 | 0.04125 |
| cg13274254 | *GULP1* | 51454 | 0.10 | 0.04125 |
| cg13383490 | *TSG101* | 7251 | 0.11 | 0.04125 |
| cg14155482 | *PIP5K1A* | 8394 | 0.14 | 0.04125 |
| cg14448104 | *MATN4* | 8785 | -0.34 | 0.04125 |
| cg15652212 | *FLJ90586* | 135932 | -0.46 | 0.04125 |
| cg15757271 | *WNT5A* | 7474 | 0.09 | 0.04125 |
| cg15770654 | *IIP45* | 60672 | -0.47 | 0.04125 |
| cg15774153 | *FGF19* | 9965 | 0.10 | 0.04125 |
| cg16463460 | *WT1* | 7490 | -0.41 | 0.04125 |
| cg16718678 | *NRXN2* | 9379 | 0.11 | 0.04125 |
| cg17388484 | *JUP* | 3728 | 0.12 | 0.04125 |
| cg17509807 | *GTF3C2* | 2976 | 0.08 | 0.04125 |
| cg18081104 | *THEM5* | 284486 | 0.14 | 0.04125 |
| cg18403396 | *B3GAT2* | 135152 | 0.10 | 0.04125 |
| cg18505908 | *APBA1* | 320 | 0.10 | 0.04125 |
| cg18509239 | *IGSF10* | 285313 | -0.09 | 0.04125 |
| cg18565479 | *DISC1* | 27185 | 0.11 | 0.04125 |
| cg19196684 | *SLC1A7* | 6512 | -0.08 | 0.04125 |
| cg19373170 | *SFMBT1* | 51460 | 0.12 | 0.04125 |
| cg19668255 | *LOC81558* | 81558 | 0.08 | 0.04125 |
| cg19873701 | *PSG9* | 5678 | 0.56 | 0.04125 |
| cg20377677 | *ZNF496* | 84838 | 0.07 | 0.04125 |
| cg20888386 | *CRYBA2* | 1412 | 0.08 | 0.04125 |
| cg21255732 | *GPD1L* | 23171 | 0.13 | 0.04125 |
| cg21607649 | *GPR123* | 84435 | 0.12 | 0.04125 |
| cg21932800 | *TMEM32* | 93380 | 0.10 | 0.04125 |
| cg22575540 | *TRIM54* | 57159 | -0.09 | 0.04125 |
| cg22647507 | *PEG10* | 23089 | -0.31 | 0.04125 |
| cg22807551 | *MTUS1* | 57509 | 0.09 | 0.04125 |
| cg22880820 | *C9orf100* | 84904 | 0.14 | 0.04125 |
| cg23166362 | *PIK3CD* | 5293 | 0.11 | 0.04125 |
| cg23455517 | *BCAT2* | 587 | 0.15 | 0.04125 |
| cg23540745 | *HIST1H4G* | 8369 | -0.47 | 0.04125 |
| cg23850377 | *LAPTM4A* | 9741 | 0.10 | 0.04125 |
| cg25013838 | *MYO18B* | 84700 | -0.40 | 0.04125 |
| cg26146086 | *RPS12* | 6206 | 0.10 | 0.04125 |
| cg26491484 | *LAMC3* | 10319 | -0.46 | 0.04125 |
| cg26627486 | *C7orf27* | 221927 | 0.07 | 0.04125 |
| cg26831968 | *GBF1* | 8729 | 0.10 | 0.04125 |
| cg26932976 | *KIAA0664* | 23277 | 0.07 | 0.04125 |
| cg26940725 | *RPL21* | 6144 | 0.11 | 0.04125 |
| cg02710105 | *MAP4K3* | 8491 | 0.10 | 0.04127 |
| cg02879662 | *HIF3A* | 64344 | 0.09 | 0.04127 |
| cg07347645 | *SYCP2* | 10388 | -0.35 | 0.04127 |
| cg14046986 | *RCSD1* | 92241 | 0.12 | 0.04127 |
| cg01665633 | *NDNL2* | 56160 | 0.11 | 0.04129 |
| cg04123507 | *KRTHB6* | 3892 | -0.36 | 0.04129 |
| cg05517572 | *STAP2* | 55620 | 0.25 | 0.04129 |
| cg08351143 | *UBE2R2* | 54926 | 0.12 | 0.04129 |
| cg11608114 | *LOC338799* | 338799 | 0.13 | 0.04129 |
| cg26530341 | *TNFRSF10A* | 8797 | -0.41 | 0.04129 |
| cg17404081 | *SAPS3* | 55291 | 0.10 | 0.04129 |
| cg03555203 | *ZNF537* | 57616 | -0.09 | 0.04130 |
| cg03724463 | *CCK* | 885 | -0.41 | 0.04130 |
| cg15249164 | *ATP5O* | 539 | 0.10 | 0.04130 |
| cg21243909 | *RCC2* | 55920 | 0.10 | 0.04130 |
| cg02765328 | *CCND2* | 894 | 0.11 | 0.04130 |
| cg11738011 | *PUS1* | 80324 | 0.13 | 0.04130 |
| cg10618882 | *LRRC25* | 126364 | -0.10 | 0.04131 |
| cg13882835 | *TLK1* | 9874 | 0.14 | 0.04131 |
| cg07473175 | *AMIGO2* | 347902 | 0.11 | 0.04132 |
| cg09069593 | *GPR114* | 221188 | 0.45 | 0.04132 |
| cg16129797 | *MRPL15* | 29088 | 0.09 | 0.04132 |
| cg25661884 | *RDH8* | 50700 | 0.22 | 0.04132 |
| cg04808672 | *SMURF2* | 64750 | 0.10 | 0.04132 |
| cg22209624 | *SERTAD4* | 56256 | 0.12 | 0.04132 |
| cg25175370 | *C7orf27* | 221927 | 0.13 | 0.04132 |
| cg07112210 | *MGAT5B* | 146664 | -0.51 | 0.04133 |
| cg10635061 | *FHL2* | 2274 | 0.46 | 0.04133 |
| cg13333141 | *BTG1* | 694 | 0.09 | 0.04133 |
| cg21177096 | *MATN3* | 4148 | 0.09 | 0.04133 |
| cg21219744 | *GUF1* | 60558 | 0.10 | 0.04133 |
| cg21686523 | *PHF22* | 57117 | 0.10 | 0.04133 |
| cg20008332 | *SOX11* | 6664 | 0.09 | 0.04135 |
| cg05656364 | *VAMP8* | 8673 | 0.44 | 0.04135 |
| cg17341703 | *SLC35A1* | 10559 | 0.10 | 0.04135 |
| cg21488617 | *DNALI1* | 7802 | -0.29 | 0.04137 |
| cg06720424 | *RAP80* | 51720 | 0.09 | 0.04137 |
| cg03218019 | *DDOST* | 1650 | -0.35 | 0.04138 |
| cg12754733 | *FLJ35119* | 126074 | 0.09 | 0.04139 |
| cg15416577 | *CCDC52* | 152185 | 0.08 | 0.04140 |
| cg18849169 | *GPX3* | 2878 | -0.33 | 0.04140 |
| cg19438514 | *C20orf102* | 128434 | -0.50 | 0.04140 |
| cg05877109 | *BCL2L2* | 599 | 0.27 | 0.04141 |
| cg22305782 | *FBXL19* | 54620 | -0.23 | 0.04141 |
| cg11921829 | *FCRL2* | 79368 | 0.28 | 0.04143 |
| cg00239685 | *GORASP1* | 64689 | 0.12 | 0.04143 |
| cg22184145 | *POLR2B* | 5431 | 0.08 | 0.04143 |
| cg09451092 | *CCDC19* | 25790 | -0.51 | 0.04146 |
| cg13409216 | *SUPV3L1* | 6832 | 0.10 | 0.04146 |
| cg23979923 | *CIB1* | 10519 | 0.09 | 0.04146 |
| cg24091698 | *ERCC4* | 2072 | 0.09 | 0.04146 |
| cg12983442 | *ZNF552* | 79818 | 0.10 | 0.04146 |
| cg16390856 | *MAGEA3* | 4102 | -0.38 | 0.04146 |
| cg03430998 | *TLR1* | 7096 | 0.23 | 0.04146 |
| cg05492270 | *RNF121* | 55298 | 0.10 | 0.04146 |
| cg06077733 | *UBE2D1* | 7321 | 0.09 | 0.04146 |
| cg06497752 | *COL9A3* | 1299 | 0.07 | 0.04146 |
| cg06641366 | *LRRC8C* | 84230 | -0.42 | 0.04146 |
| cg08229694 | *IMMT* | 10989 | 0.10 | 0.04146 |
| cg11973688 | *FPGS* | 2356 | 0.07 | 0.04146 |
| cg18331396 | *RAF1* | 5894 | 0.11 | 0.04146 |
| cg27196129 | *C16orf58* | 64755 | 0.14 | 0.04146 |
| cg05341878 | *RIMS2* | 9699 | 0.14 | 0.04147 |
| cg13303256 | *PHF1* | 5252 | 0.11 | 0.04147 |
| cg13954292 | *ZFR* | 51663 | -0.50 | 0.04147 |
| cg25813820 | *MPP1* | 4354 | 0.13 | 0.04147 |
| cg01962969 | *CBR1* | 873 | 0.07 | 0.04147 |
| cg02091100 | *GUCA1A* | 2978 | 0.31 | 0.04147 |
| cg04563996 | *SPATA12* | 353324 | 0.28 | 0.04147 |
| cg05705366 | *SNAG1* | 112574 | 0.10 | 0.04147 |
| cg09996240 | *HMG20B* | 10362 | -0.53 | 0.04147 |
| cg12359315 | *SLC6A20* | 54716 | 0.11 | 0.04147 |
| cg15531099 | *LCE1D* | 353134 | 0.43 | 0.04147 |
| cg15677355 | *OTUD6A* | 139562 | -0.41 | 0.04147 |
| cg20691408 | *FADS3* | 3995 | 0.13 | 0.04147 |
| cg23123694 | *SLC38A2* | 54407 | 0.12 | 0.04147 |
| cg24073022 | *TAL2* | 6887 | -0.09 | 0.04147 |
| cg25533774 | *SOSTDC1* | 25928 | 0.30 | 0.04147 |
| cg27431150 | *SLIC1* | 124460 | 0.36 | 0.04147 |
| cg27505538 | *STARD5* | 80765 | 0.08 | 0.04147 |
| cg02264284 | *HPRT1* | 3251 | 0.14 | 0.04149 |
| cg03020271 | *M6PRBP1* | 10226 | 0.14 | 0.04149 |
| cg04278905 | *GCN5L2* | 2648 | -0.37 | 0.04149 |
| cg06617418 | *RGAG1* | 57529 | -0.33 | 0.04149 |
| cg07475247 | *PMPCB* | 9512 | 0.11 | 0.04149 |
| cg10337772 | *KIAA1143* | 57456 | 0.11 | 0.04149 |
| cg12266024 | *DRCTNNB1A* | 84668 | 0.11 | 0.04149 |
| cg13682722 | *C14orf102* | 55051 | 0.10 | 0.04149 |
| cg14986136 | *WBP5* | 51186 | 0.11 | 0.04149 |
| cg19669439 | *BTRC* | 8945 | 0.10 | 0.04149 |
| cg26415633 | *KLK1* | 3816 | 0.20 | 0.04149 |
| cg26922202 | *OR2S2* | 56656 | -0.17 | 0.04149 |
| cg09985635 | *PCDH1* | 5097 | 0.10 | 0.04149 |
| cg13471990 | *ENTPD1* | 953 | 0.12 | 0.04150 |
| cg18388972 | *CCT3* | 7203 | 0.09 | 0.04150 |
| cg05973262 | *NOTCH4* | 4855 | 0.22 | 0.04151 |
| cg19784477 | *TFPI2* | 7980 | 0.14 | 0.04151 |
| cg22336633 | *RPS13* | 6207 | 0.10 | 0.04151 |
| cg23776892 | *MAGEA1* | 4100 | -0.43 | 0.04151 |
| cg23845507 | *CDC7* | 8317 | 0.07 | 0.04151 |
| cg26879282 | *KCNQ1* | 3784 | -0.07 | 0.04154 |
| cg08713365 | *C20orf98* | 80023 | 0.18 | 0.04154 |
| cg15003158 | *KENAE* | 202243 | 0.10 | 0.04155 |
| cg12589433 | *AR* | 367 | 0.12 | 0.04156 |
| cg24489015 | *LPO* | 4025 | 0.36 | 0.04157 |
| cg26628847 | *PIP* | 5304 | 0.46 | 0.04157 |
| cg18328933 | *ABHD14A* | 25864 | -0.38 | 0.04160 |
| cg22252999 | *ART3* | 419 | 0.32 | 0.04160 |
| cg11821536 | *TTC8* | 123016 | 0.09 | 0.04161 |
| cg00577464 | *SHH* | 6469 | 0.08 | 0.04161 |
| cg01091448 | *AMACR* | 23600 | 0.09 | 0.04161 |
| cg01275830 | *C10orf95* | 79946 | -0.36 | 0.04161 |
| cg01519742 | *JAKMIP1* | 152789 | 0.11 | 0.04161 |
| cg01607495 | *BAG4* | 9530 | -0.27 | 0.04161 |
| cg02275830 | *ZADH1* | 145482 | 0.09 | 0.04161 |
| cg04032871 | *QPCTL* | 54814 | 0.10 | 0.04161 |
| cg05045383 | *RAE1* | 8480 | 0.09 | 0.04161 |
| cg05650764 | *SAPS3* | 55291 | 0.11 | 0.04161 |
| cg06111305 | *RAP2A* | 5911 | 0.12 | 0.04161 |
| cg06397510 | *PTP4A2* | 8073 | 0.17 | 0.04161 |
| cg08136893 | *C7orf11* | 136647 | 0.12 | 0.04161 |
| cg08372326 | *HABP4* | 22927 | 0.11 | 0.04161 |
| cg08631151 | *RPRML* | 388394 | 0.08 | 0.04161 |
| cg09588653 | *SLC6A7* | 6534 | -0.21 | 0.04161 |
| cg10173075 | *RLBP1* | 6017 | 0.36 | 0.04161 |
| cg10524192 | *OPRS1* | 10280 | 0.09 | 0.04161 |
| cg12836863 | *BRCA2* | 675 | 0.10 | 0.04161 |
| cg13140464 | *AP3B2* | 8120 | -0.46 | 0.04161 |
| cg13592003 | *ZNF250* | 58500 | 0.08 | 0.04161 |
| cg14230685 | *SPIN* | 10927 | 0.09 | 0.04161 |
| cg17854440 | *ENPEP* | 2028 | -0.34 | 0.04161 |
| cg18233735 | *PDZD4* | 57595 | 0.11 | 0.04161 |
| cg18621299 | *EVL* | 51466 | 0.36 | 0.04161 |
| cg20401181 | *SLC30A7* | 148867 | 0.12 | 0.04161 |
| cg21896452 | *TPRKB* | 51002 | 0.10 | 0.04161 |
| cg23278885 | *TGM6* | 343641 | 0.43 | 0.04161 |
| cg23494853 | *C2orf25* | 27249 | 0.08 | 0.04161 |
| cg24744425 | *RABIF* | 5877 | 0.08 | 0.04161 |
| cg25136045 | *SDCCAG10* | 10283 | 0.12 | 0.04161 |
| cg00253200 | *C3orf59* | 151963 | 0.11 | 0.04161 |
| cg01447420 | *FEN1* | 2237 | 0.11 | 0.04161 |
| cg04463638 | *CLDN5* | 7122 | -0.38 | 0.04161 |
| cg09509576 | *GRB2* | 2885 | 0.08 | 0.04161 |
| cg12696750 | *DNALI1* | 7802 | 0.11 | 0.04161 |
| cg15753757 | *AUTS2* | 26053 | 0.11 | 0.04161 |
| cg23322523 | *TRIM55* | 84675 | -0.15 | 0.04161 |
| cg23507131 | *DARC* | 2532 | 0.50 | 0.04161 |
| cg26862286 | *NCL* | 4691 | -0.49 | 0.04161 |
| cg02989940 | *ERAF* | 51327 | -0.43 | 0.04162 |
| cg00930194 | *PROP1* | 5626 | 0.34 | 0.04163 |
| cg15028436 | *TXNDC3* | 51314 | -0.40 | 0.04163 |
| cg21703138 | *PPP2CA* | 5515 | 0.08 | 0.04163 |
| cg05960024 | *CLOCK* | 9575 | -0.13 | 0.04163 |
| cg04561804 | *TLOC1* | 7095 | -0.30 | 0.04163 |
| cg11476211 | *PRKCE* | 5581 | 0.08 | 0.04163 |
| cg13135180 | *RPP21* | 79897 | 0.09 | 0.04163 |
| cg00078867 | *WAS* | 7454 | 0.32 | 0.04164 |
| cg00962799 | *SSX8* | 280659 | -0.24 | 0.04164 |
| cg01127428 | *SYCP3* | 50511 | -0.43 | 0.04164 |
| cg03062665 | *CALM1* | 801 | 0.11 | 0.04164 |
| cg03309050 | *ZNF688* | 146542 | 0.08 | 0.04164 |
| cg03855656 | *SERHL* | 94009 | -0.34 | 0.04164 |
| cg04451988 | *C10orf38* | 221061 | 0.12 | 0.04164 |
| cg08003150 | *KCNQ2* | 3785 | 0.12 | 0.04164 |
| cg10698549 | *SRRM1* | 10250 | 0.12 | 0.04164 |
| cg10771262 | *TCF21* | 6943 | -0.39 | 0.04164 |
| cg11038843 | *B3GAT1* | 27087 | 0.14 | 0.04164 |
| cg14602658 | *TMEM39A* | 55254 | 0.10 | 0.04164 |
| cg19678067 | *ZNF403* | 79893 | 0.12 | 0.04164 |
| cg24068708 | *GDPD5* | 81544 | 0.10 | 0.04164 |
| cg25156443 | *SFRP5* | 6425 | 0.21 | 0.04164 |
| cg26465666 | *PDE1A* | 5136 | 0.18 | 0.04164 |
| cg10246296 | *CLCN4* | 1183 | 0.10 | 0.04165 |
| cg24003542 | *MCC* | 4163 | 0.09 | 0.04165 |
| cg03717979 | *MSX1* | 4487 | 0.09 | 0.04166 |
| cg20648149 | *SYNE2* | 23224 | -0.26 | 0.04166 |
| cg23424962 | *GRIA3* | 2892 | 0.13 | 0.04167 |
| cg04393897 | *BAT2D1* | 23215 | 0.09 | 0.04168 |
| cg02068676 | *COBRA1* | 25920 | 0.10 | 0.04168 |
| cg02844051 | *ZD52F10* | 93099 | -0.12 | 0.04168 |
| cg02845923 | *C12orf26* | 84190 | -0.37 | 0.04168 |
| cg03565782 | *IER5* | 51278 | 0.10 | 0.04168 |
| cg03916787 | *RPL35* | 11224 | 0.14 | 0.04168 |
| cg04211807 | *ZCCHC13* | 389874 | -0.41 | 0.04168 |
| cg04936062 | *THRAP3* | 9967 | 0.07 | 0.04168 |
| cg08097657 | *SEMA3B* | 7869 | -0.22 | 0.04168 |
| cg08141873 | *HRAS* | 3265 | -0.37 | 0.04168 |
| cg11192270 | *ATP13A2* | 23400 | 0.07 | 0.04168 |
| cg13818368 | *SPAG7* | 9552 | 0.11 | 0.04168 |
| cg16122592 | *MAGEB6* | 158809 | -0.30 | 0.04168 |
| cg16616734 | *ANKRD13* | 88455 | 0.07 | 0.04168 |
| cg17309462 | *C20orf38* | 55304 | 0.32 | 0.04168 |
| cg19869139 | *OPRD1* | 4985 | -0.41 | 0.04168 |
| cg21984310 | *C9orf127* | 51754 | 0.11 | 0.04168 |
| cg24822446 | *ZFAND3* | 60685 | 0.10 | 0.04168 |
| cg24977027 | *FLJ10916* | 55258 | -0.31 | 0.04168 |
| cg25018755 | *BIK* | 638 | 0.09 | 0.04168 |
| cg13120519 | *ALKBH7* | 84266 | 0.08 | 0.04169 |
| cg15006973 | *GJA4* | 2701 | -0.45 | 0.04169 |
| cg25797366 | *HSPA2* | 3306 | -0.17 | 0.04169 |
| cg03907454 | *KIF2C* | 11004 | 0.12 | 0.04170 |
| cg11603096 | *EIF3S7* | 8664 | 0.09 | 0.04170 |
| cg04683240 | *HLX1* | 3142 | 0.12 | 0.04172 |
| cg00864867 | *PAWR* | 5074 | 0.10 | 0.04172 |
| cg18627852 | *SNRPF* | 6636 | 0.09 | 0.04175 |
| cg10044101 | *VNN2* | 8875 | 0.33 | 0.04177 |
| cg25954354 | *C9orf58* | 83543 | 0.12 | 0.04178 |
| cg18464559 | *ITGB3BP* | 23421 | 0.08 | 0.04178 |
| cg18910313 | *P2RY11* | 5032 | 0.34 | 0.04178 |
| cg21034676 | *TGFBI* | 7045 | 0.11 | 0.04178 |
| cg09425215 | *CHD2* | 1106 | 0.12 | 0.04181 |
| cg20972543 | *HERC3* | 8916 | 0.12 | 0.04182 |
| cg08574156 | *CAMK2N2* | 94032 | 0.11 | 0.04183 |
| cg22058458 | *HMGN3* | 9324 | 0.08 | 0.04183 |
| cg22437699 | *ARX* | 170302 | -0.44 | 0.04183 |
| cg22918700 | *LOC161247* | 161247 | -0.10 | 0.04183 |
| cg00630583 | *POLE* | 5426 | 0.08 | 0.04184 |
| cg06615861 | *KIF1B* | 23095 | 0.11 | 0.04184 |
| cg15046693 | *CEBPG* | 1054 | -0.34 | 0.04184 |
| cg26233914 | *ITGAX* | 3687 | 0.49 | 0.04184 |
| cg26413355 | *UNQ9217* | 167555 | 0.10 | 0.04184 |
| cg00775197 | *MGC33600* | 202500 | 0.10 | 0.04185 |
| cg06625767 | *F12* | 2161 | 0.34 | 0.04185 |
| cg11208483 | *SOX21* | 11166 | -0.45 | 0.04185 |
| cg15077637 | *EFTUD2* | 9343 | 0.09 | 0.04185 |
| cg20149998 | *CHCHD8* | 51287 | 0.13 | 0.04185 |
| cg20676303 | *GAGE7B* | 26748 | -0.38 | 0.04185 |
| cg23621115 | *PRKCABP* | 9463 | -0.35 | 0.04185 |
| cg25020459 | *COL6A1* | 1291 | 0.10 | 0.04185 |
| cg25118574 | *PSMB7* | 5695 | 0.09 | 0.04185 |
| cg26783353 | *SLC25A2* | 83884 | -0.46 | 0.04186 |
| cg05767404 | *C1orf150* | 148823 | 0.31 | 0.04188 |
| cg10556064 | *SMPD3* | 55512 | -0.41 | 0.04188 |
| cg02336128 | *DCK* | 1633 | 0.09 | 0.04188 |
| cg02552945 | *C5* | 727 | -0.12 | 0.04188 |
| cg03332113 | *RAI14* | 26064 | 0.11 | 0.04188 |
| cg03447931 | *BMP6* | 654 | 0.09 | 0.04188 |
| cg04275985 | *PRUNE* | 58497 | 0.09 | 0.04188 |
| cg04384398 | *PMM1* | 5372 | 0.15 | 0.04188 |
| cg04589904 | *ADRB1* | 153 | 0.13 | 0.04188 |
| cg05308484 | *C12orf5* | 57103 | 0.09 | 0.04188 |
| cg06465194 | *GRIK4* | 2900 | 0.24 | 0.04188 |
| cg06687562 | *ACTL8* | 81569 | 0.13 | 0.04188 |
| cg08735298 | *C1orf122* | 127687 | 0.11 | 0.04188 |
| cg10248733 | *CALR3* | 125972 | 0.11 | 0.04188 |
| cg10753073 | *UBE2U* | 148581 | -0.30 | 0.04188 |
| cg11730100 | *FEN1* | 2237 | 0.14 | 0.04188 |
| cg11833861 | *TMEM98* | 26022 | 0.21 | 0.04188 |
| cg11882252 | *LOC134147* | 134147 | 0.10 | 0.04188 |
| cg12598364 | *GCAT* | 23464 | 0.16 | 0.04188 |
| cg12661229 | *C3orf54* | 389119 | 0.10 | 0.04188 |
| cg12938998 | *GAB3* | 139716 | 0.08 | 0.04188 |
| cg13294509 | *GLE1L* | 2733 | 0.19 | 0.04188 |
| cg14011734 | *TATDN2* | 9797 | 0.09 | 0.04188 |
| cg16185365 | *RNF168* | 165918 | 0.10 | 0.04188 |
| cg17640322 | *CD44* | 960 | 0.10 | 0.04188 |
| cg22377142 | *C1orf85* | 112770 | 0.37 | 0.04188 |
| cg22587514 | *MRPS2* | 51116 | 0.09 | 0.04188 |
| cg25072179 | *AGTRL1* | 187 | 0.40 | 0.04188 |
| cg25168545 | *GIMAP1* | 170575 | 0.35 | 0.04188 |
| cg25372135 | *DARS2* | 55157 | 0.09 | 0.04188 |
| cg25400358 | *GPR137* | 56834 | -0.34 | 0.04188 |
| cg25933726 | *RPGR* | 6103 | 0.08 | 0.04188 |
| cg26095194 | *DUSP1* | 1843 | 0.09 | 0.04188 |
| cg27056119 | *SERPINB1* | 1992 | 0.10 | 0.04188 |
| cg07841014 | *REG1B* | 5968 | 0.50 | 0.04189 |
| cg11328541 | *OR7C1* | 26664 | -0.29 | 0.04189 |
| cg15470298 | *COL18A1* | 80781 | 0.38 | 0.04189 |
| cg09128050 | *SAMHD1* | 25939 | 0.09 | 0.04190 |
| cg12827188 | *FGA* | 2243 | 0.13 | 0.04190 |
| cg06090351 | *ADSS* | 159 | 0.10 | 0.04190 |
| cg07815386 | *RNF149* | 284996 | 0.07 | 0.04190 |
| cg13976366 | *TRIM21* | 6737 | 0.09 | 0.04190 |
| cg23490074 | *C19orf2* | 8725 | 0.14 | 0.04190 |
| cg21800439 | *STAT2* | 6773 | 0.09 | 0.04190 |
| cg23702568 | *KIAA0261* | 23063 | 0.09 | 0.04190 |
| cg18477315 | *GOLGA5* | 9950 | 0.10 | 0.04191 |
| cg20141751 | *GLT8D1* | 55830 | 0.10 | 0.04191 |
| cg10478221 | *WFIKKN1* | 117166 | 0.36 | 0.04191 |
| cg16007680 | *GLI4* | 2738 | 0.10 | 0.04191 |
| cg06938878 | *CALCB* | 797 | -0.46 | 0.04192 |
| cg15882999 | *P4HB* | 5034 | 0.08 | 0.04192 |
| cg23070585 | *RXRB* | 6257 | 0.08 | 0.04192 |
| cg24968786 | *FLJ10786* | 55231 | 0.11 | 0.04192 |
| cg25003924 | *ARPP-19* | 10776 | -0.32 | 0.04192 |
| cg05807444 | *VPS28* | 51160 | -0.43 | 0.04193 |
| cg20357806 | *PPBP* | 5473 | 0.14 | 0.04194 |
| cg15752043 | *NANOS1* | 340719 | 0.09 | 0.04195 |
| cg03805364 | *PAFAH1B2* | 5049 | 0.08 | 0.04195 |
| cg01182585 | *C7orf30* | 115416 | 0.09 | 0.04196 |
| cg02901122 | *P15RS* | 55197 | 0.08 | 0.04196 |
| cg07872652 | *CNIH2* | 254263 | 0.12 | 0.04196 |
| cg11718315 | *PARP16* | 54956 | 0.12 | 0.04196 |
| cg11891583 | *GLUD2* | 2747 | -0.24 | 0.04196 |
| cg13216057 | *DKK3* | 27122 | 0.10 | 0.04196 |
| cg14081015 | *RIOK2* | 55781 | 0.12 | 0.04196 |
| cg17511416 | *C9orf93* | 203238 | 0.11 | 0.04196 |
| cg25533361 | *EIF3S9* | 8662 | 0.10 | 0.04196 |
| cg25650811 | *LOC223075* | 223075 | -0.35 | 0.04196 |
| cg26863324 | *DDX52* | 11056 | 0.12 | 0.04196 |
| cg10562586 | *MAP2* | 4133 | -0.32 | 0.04197 |
| cg25411534 | *C2orf15* | 150590 | 0.10 | 0.04197 |
| cg00890257 | *DEGS2* | 123099 | 0.12 | 0.04198 |
| cg03759077 | *ARL6IP6* | 151188 | 0.10 | 0.04199 |
| cg24480859 | *SLC2A5* | 6518 | 0.39 | 0.04199 |
| cg08299045 | *MRPL55* | 128308 | 0.07 | 0.04199 |
| cg10168628 | *WBSCR19* | 285955 | -0.25 | 0.04199 |
| cg13785207 | *RPS19BP1* | 91582 | 0.07 | 0.04199 |
| cg14037665 | *PXDN* | 7837 | 0.10 | 0.04199 |
| cg14519525 | *GANAB* | 23193 | 0.09 | 0.04199 |
| cg19691797 | *TMEM66* | 51669 | 0.09 | 0.04199 |
| cg19853760 | *LGALS1* | 3956 | -0.41 | 0.04199 |
| cg22284975 | *C7orf11* | 136647 | 0.07 | 0.04199 |
| cg22285621 | *SSH3* | 54961 | 0.09 | 0.04199 |
| cg22767079 | *BIVM* | 54841 | 0.07 | 0.04199 |
| cg23508540 | *CLSTN1* | 22883 | 0.11 | 0.04199 |
| cg02060988 | *LASP1* | 3927 | 0.12 | 0.04203 |
| cg09970593 | *OPRD1* | 4985 | -0.37 | 0.04203 |
| cg21030400 | *MKNK2* | 2872 | 0.17 | 0.04203 |
| cg23898073 | *GFRA1* | 2674 | 0.11 | 0.04203 |
| cg25466974 | *SLC30A1* | 7779 | 0.14 | 0.04204 |
| cg00024396 | *ELOVL5* | 60481 | 0.11 | 0.04205 |
| cg02027010 | *ALAD* | 210 | 0.08 | 0.04205 |
| cg02863947 | *NR1I2* | 8856 | 0.41 | 0.04205 |
| cg03054529 | *SCRN1* | 9805 | -0.27 | 0.04205 |
| cg03414321 | *HCK* | 3055 | 0.13 | 0.04205 |
| cg04080057 | *CACNG6* | 59285 | -0.40 | 0.04205 |
| cg04121771 | *TM4SF4* | 7104 | 0.30 | 0.04205 |
| cg04348872 | *ADCY3* | 109 | -0.51 | 0.04205 |
| cg05654164 | *C1orf52* | 148423 | -0.45 | 0.04205 |
| cg05815893 | *C17orf80* | 55028 | 0.09 | 0.04205 |
| cg05965188 | *KIAA0179* | 23076 | -0.43 | 0.04205 |
| cg07175007 | *UHMK1* | 127933 | -0.44 | 0.04205 |
| cg07265300 | *PFN4* | 375189 | 0.10 | 0.04205 |
| cg07379574 | *C19orf4* | 25789 | 0.14 | 0.04205 |
| cg07461582 | *RINT-1* | 60561 | 0.06 | 0.04205 |
| cg08291000 | *PEG10* | 23089 | -0.27 | 0.04205 |
| cg08803144 | *ATIC* | 471 | 0.12 | 0.04205 |
| cg09755102 | *YARS* | 8565 | 0.06 | 0.04205 |
| cg09845785 | *PDE1A* | 5136 | 0.33 | 0.04205 |
| cg10920302 | *LARS2* | 23395 | 0.11 | 0.04205 |
| cg10964421 | *TNFRSF10D* | 8793 | 0.12 | 0.04205 |
| cg11951446 | *COX17* | 10063 | 0.12 | 0.04205 |
| cg13164537 | *CD226* | 10666 | 0.52 | 0.04205 |
| cg13514050 | *GGTL3* | 2686 | 0.10 | 0.04205 |
| cg13619915 | *SLITRK3* | 22865 | 0.11 | 0.04205 |
| cg13697378 | *DIRAS3* | 9077 | -0.46 | 0.04205 |
| cg14681629 | *LOC153684* | 153684 | 0.13 | 0.04205 |
| cg15133363 | *HIG2* | 29923 | 0.13 | 0.04205 |
| cg15454483 | *SNX12* | 29934 | 0.09 | 0.04205 |
| cg15676084 | *ENAH* | 55740 | 0.08 | 0.04205 |
| cg16098981 | *C20orf39* | 79953 | 0.12 | 0.04205 |
| cg16546489 | *HSD17B1* | 3292 | -0.11 | 0.04205 |
| cg16564824 | *SNAP25* | 6616 | 0.09 | 0.04205 |
| cg18130100 | *PKD1* | 5310 | 0.44 | 0.04205 |
| cg18289156 | *FLJ37357* | 284944 | 0.08 | 0.04205 |
| cg18299211 | *PIGK* | 10026 | 0.08 | 0.04205 |
| cg18380974 | *WIG1* | 64393 | 0.11 | 0.04205 |
| cg18939260 | *MTSS1* | 9788 | -0.52 | 0.04205 |
| cg20716209 | *STAT3* | 6774 | 0.11 | 0.04205 |
| cg21553524 | *MGC33926* | 130733 | 0.08 | 0.04205 |
| cg23164327 | *OSRF* | 23548 | 0.12 | 0.04205 |
| cg24530795 | *FAM109A* | 144717 | 0.10 | 0.04205 |
| cg24794433 | *DAB2IP* | 153090 | 0.10 | 0.04205 |
| cg25077654 | *DSCR2* | 8624 | 0.11 | 0.04205 |
| cg08100497 | *NRG2* | 9542 | 0.09 | 0.04205 |
| cg08383315 | *RIC3* | 79608 | 0.12 | 0.04205 |
| cg17738194 | *GK2* | 2712 | -0.26 | 0.04205 |
| cg25781202 | *RNF122* | 79845 | 0.10 | 0.04205 |
| cg26833602 | *SUSD3* | 203328 | 0.09 | 0.04205 |
| cg08977028 | *CSAG1* | 158511 | -0.46 | 0.04206 |
| cg02943497 | *PARP4* | 143 | 0.11 | 0.04206 |
| cg08993172 | *ALX4* | 60529 | 0.11 | 0.04207 |
| cg04660617 | *RABL2B* | 11158 | 0.09 | 0.04207 |
| cg06590533 | *C20orf108* | 116151 | 0.12 | 0.04207 |
| cg13501999 | *CACNB1* | 782 | 0.10 | 0.04207 |
| cg14119236 | *FGF23* | 8074 | 0.26 | 0.04207 |
| cg10806140 | *SFRP4* | 6424 | 0.10 | 0.04207 |
| cg21068339 | *DSP* | 1832 | 0.10 | 0.04208 |
| cg00859193 | *C13orf12* | 51371 | 0.08 | 0.04208 |
| cg02910574 | *PCOLN3* | 5119 | -0.45 | 0.04208 |
| cg20670302 | *PSTPIP2* | 9050 | 0.07 | 0.04208 |
| cg12985164 | *TSEN2* | 80746 | 0.11 | 0.04209 |
| cg23037133 | *OXGR1* | 27199 | 0.10 | 0.04209 |
| cg07042144 | *NALP12* | 91662 | 0.39 | 0.04209 |
| cg18881723 | *SLAMF1* | 6504 | 0.40 | 0.04209 |
| cg12556134 | *TGIF2* | 60436 | 0.10 | 0.04210 |
| cg13425637 | *PRKCH* | 5583 | 0.09 | 0.04210 |
| cg19280014 | *MTX2* | 10651 | 0.09 | 0.04210 |
| cg21325760 | *MAGEL2* | 54551 | -0.40 | 0.04210 |
| cg00461841 | *ATF7IP2* | 80063 | -0.12 | 0.04211 |
| cg01999787 | *ALG5* | 29880 | 0.14 | 0.04211 |
| cg02099418 | *CD28* | 940 | 0.32 | 0.04211 |
| cg04113075 | *RAB32* | 10981 | 0.09 | 0.04211 |
| cg11804789 | *CST7* | 8530 | 0.36 | 0.04211 |
| cg15329642 | *PTCH2* | 8643 | -0.44 | 0.04211 |
| cg15534084 | *ZNF649* | 65251 | 0.09 | 0.04211 |
| cg25557858 | *EXTL3* | 2137 | 0.07 | 0.04211 |
| cg01141769 | *USP25* | 29761 | 0.12 | 0.04211 |
| cg04879696 | *SPG7* | 6687 | 0.09 | 0.04211 |
| cg12005703 | *HEMK1* | 51409 | 0.10 | 0.04211 |
| cg25729068 | *MTAC2D1* | 123036 | 0.09 | 0.04211 |
| cg26916927 | *GLO1* | 2739 | 0.10 | 0.04211 |
| cg27076139 | *ADCYAP1R1* | 117 | 0.09 | 0.04212 |
| cg02831294 | *CALML3* | 810 | -0.43 | 0.04212 |
| cg08647446 | *RASSF6* | 166824 | 0.08 | 0.04212 |
| cg15156367 | *ABHD7* | 253152 | -0.45 | 0.04213 |
| cg03830408 | *DCPS* | 28960 | 0.11 | 0.04213 |
| cg07356189 | *CXorf2* | 1527 | -0.43 | 0.04213 |
| cg23185262 | *PDXK* | 8566 | -0.12 | 0.04213 |
| cg11449935 | *TGIF2* | 60436 | 0.11 | 0.04213 |
| cg14178895 | *C6orf105* | 84830 | 0.36 | 0.04214 |
| cg03790787 | *FLJ22639* | 79854 | 0.11 | 0.04214 |
| cg13222081 | *MRPL52* | 122704 | 0.13 | 0.04214 |
| cg01215061 | *TGIF2LX* | 90316 | -0.34 | 0.04216 |
| cg03171924 | *RUNX3* | 864 | -0.51 | 0.04216 |
| cg05429895 | *TLR4* | 7099 | 0.44 | 0.04217 |
| cg05512756 | *UGT3A1* | 133688 | -0.19 | 0.04217 |
| cg06351503 | *RDBP* | 7936 | -0.08 | 0.04217 |
| cg11039072 | *FLJ20582* | 54989 | 0.09 | 0.04217 |
| cg13929328 | *FLJ46831* | 399823 | 0.08 | 0.04217 |
| cg14521746 | *PAK1* | 5058 | 0.09 | 0.04217 |
| cg06344807 | *SLC9A3R1* | 9368 | 0.11 | 0.04219 |
| cg12619162 | *FXYD4* | 53828 | -0.12 | 0.04220 |
| cg22619563 | *EPHA3* | 2042 | 0.09 | 0.04220 |
| cg23776012 | *KA21* | 125113 | -0.28 | 0.04220 |
| cg18595258 | *HSPC196* | 51524 | -0.24 | 0.04221 |
| cg27242945 | *CAV1* | 857 | 0.07 | 0.04221 |
| cg02212737 | *EXOC7* | 23265 | 0.10 | 0.04222 |
| cg04779191 | *CIRH1A* | 84916 | 0.11 | 0.04222 |
| cg14837165 | *D4ST1* | 113189 | 0.09 | 0.04222 |
| cg19822214 | *CNOT3* | 4849 | 0.12 | 0.04222 |
| cg20367072 | *ETFB* | 2109 | 0.10 | 0.04222 |
| cg04775502 | *C6orf114* | 85411 | 0.11 | 0.04224 |
| cg08251399 | *EHD3* | 30845 | -0.39 | 0.04224 |
| cg10763059 | *PTPN12* | 5782 | 0.15 | 0.04224 |
| cg11599505 | *C20orf102* | 128434 | -0.50 | 0.04224 |
| cg11820734 | *MRPL35* | 51318 | 0.10 | 0.04224 |
| cg12056618 | *KLF13* | 51621 | 0.09 | 0.04224 |
| cg12125117 | *GPR97* | 222487 | 0.46 | 0.04224 |
| cg13634319 | *DGKA* | 1606 | 0.27 | 0.04224 |
| cg20018806 | *TCN1* | 6947 | 0.18 | 0.04224 |
| cg22409383 | *MATR3* | 9782 | 0.09 | 0.04224 |
| cg22812892 | *IDE* | 3416 | 0.10 | 0.04224 |
| cg11191337 | *PLTP* | 5360 | -0.22 | 0.04225 |
| cg25928579 | *HOXB8* | 3218 | 0.10 | 0.04225 |
| cg04691375 | *LRRC42* | 115353 | 0.13 | 0.04225 |
| cg02059813 | *SMARCA3* | 6596 | -0.60 | 0.04225 |
| cg02489552 | *FLJ40365* | 126402 | -0.39 | 0.04225 |
| cg04274487 | *RAB31* | 11031 | 0.10 | 0.04225 |
| cg06495924 | *CYC1* | 1537 | 0.16 | 0.04225 |
| cg09646392 | *TNFSF13B* | 10673 | 0.31 | 0.04225 |
| cg09902130 | *CD6* | 923 | 0.37 | 0.04225 |
| cg11460364 | *DPP4* | 1803 | -0.23 | 0.04225 |
| cg13405161 | *EIF2C2* | 27161 | 0.12 | 0.04225 |
| cg17349753 | *RTN4RL2* | 349667 | 0.14 | 0.04225 |
| cg22192762 | *PLXNC1* | 10154 | 0.10 | 0.04225 |
| cg22603964 | *MAN2A1* | 4124 | 0.10 | 0.04225 |
| cg23323671 | *STMN1* | 3925 | -0.24 | 0.04225 |
| cg23753610 | *DNAHL1* | 284176 | -0.08 | 0.04225 |
| cg26384034 | *C6orf117* | 112609 | 0.11 | 0.04225 |
| cg12288365 | *KCTD7* | 154881 | 0.08 | 0.04226 |
| cg01240931 | *APC* | 324 | -0.23 | 0.04227 |
| cg03135127 | *TRMT1* | 55621 | 0.09 | 0.04227 |
| cg16239406 | *MTCH1* | 23787 | 0.09 | 0.04227 |
| cg20452583 | *C16orf51* | 25880 | 0.09 | 0.04227 |
| cg23795623 | *FLJ10769* | 55739 | 0.11 | 0.04227 |
| cg23984059 | *COPB* | 1315 | 0.08 | 0.04227 |
| cg00451635 | *EMP2* | 2013 | -0.30 | 0.04227 |
| cg07265310 | *K5B* | 196374 | 0.14 | 0.04227 |
| cg11197066 | *PGAP1* | 80055 | 0.09 | 0.04227 |
| cg12569173 | *FAM104A* | 84923 | 0.06 | 0.04227 |
| cg24523000 | *GABRA1* | 2554 | -0.35 | 0.04227 |
| cg04287191 | *RENBP* | 5973 | 0.24 | 0.04228 |
| cg05573563 | *OFD1* | 8481 | -0.23 | 0.04228 |
| cg17765461 | *DAZAP1* | 26528 | 0.14 | 0.04229 |
| cg00171161 | *NUDT9* | 53343 | 0.13 | 0.04229 |
| cg02170525 | *CD8A* | 925 | 0.12 | 0.04229 |
| cg10347418 | *ZNF436* | 80818 | -0.36 | 0.04229 |
| cg11676109 | *MIZF* | 25988 | 0.08 | 0.04229 |
| cg12485992 | *SPATA22* | 84690 | -0.57 | 0.04229 |
| cg13656062 | *CYP4F2* | 8529 | -0.11 | 0.04229 |
| cg14386312 | *ZNF544* | 27300 | -0.44 | 0.04229 |
| cg15118204 | *SIRT7* | 51547 | 0.16 | 0.04229 |
| cg18333690 | *PADI4* | 23569 | 0.36 | 0.04229 |
| cg26065841 | *CHAC1* | 79094 | -0.43 | 0.04229 |
| cg26612644 | *KCTD2* | 23510 | -0.21 | 0.04229 |
| cg27285278 | *TRIM41* | 90933 | 0.09 | 0.04229 |
| cg11234457 | *FTMT* | 94033 | -0.41 | 0.04230 |
| cg04256470 | *CORT* | 1325 | -0.07 | 0.04231 |
| cg25167643 | *PTPRZ1* | 5803 | 0.14 | 0.04231 |
| cg15927263 | *PLEKHJ1* | 55111 | 0.09 | 0.04231 |
| cg06587969 | *WDR79* | 55135 | 0.10 | 0.04233 |
| cg06346081 | *ZMYND12* | 84217 | 0.12 | 0.04234 |
| cg12189880 | *RHOV* | 171177 | 0.10 | 0.04234 |
| cg13757640 | *ARHGAP8* | 23779 | 0.08 | 0.04234 |
| cg13926569 | *PAPSS2* | 9060 | -0.41 | 0.04234 |
| cg17393296 | *FLJ10986* | 55277 | 0.11 | 0.04234 |
| cg10328573 | *KRT9* | 3857 | 0.23 | 0.04234 |
| cg13917504 | *MEST* | 4232 | -0.36 | 0.04234 |
| cg00839802 | *MAN1A1* | 4121 | 0.09 | 0.04236 |
| cg01495509 | *KCNMB1* | 3779 | 0.32 | 0.04236 |
| cg03114244 | *FEN1* | 2237 | 0.08 | 0.04236 |
| cg09885086 | *GCNT1* | 2650 | 0.08 | 0.04236 |
| cg10250107 | *LOC389199* | 389199 | 0.08 | 0.04236 |
| cg12391048 | *PRSS27* | 83886 | -0.42 | 0.04236 |
| cg17034082 | *C5orf3* | 10827 | 0.09 | 0.04236 |
| cg19948014 | *SOD1* | 6647 | 0.12 | 0.04236 |
| cg21796825 | *TMEM5* | 10329 | 0.09 | 0.04236 |
| cg23953396 | *GPRC5C* | 55890 | 0.10 | 0.04236 |
| cg11628487 | *SET* | 6418 | 0.11 | 0.04236 |
| cg14550007 | *SCMH1* | 22955 | 0.09 | 0.04236 |
| cg03405173 | *ZNF441* | 126068 | 0.08 | 0.04237 |
| cg03160508 | *RHOD* | 29984 | -0.24 | 0.04238 |
| cg04826883 | *CA12* | 771 | 0.09 | 0.04238 |
| cg05661333 | *GABBR1* | 2550 | 0.08 | 0.04238 |
| cg10583097 | *RBM15B* | 29890 | 0.14 | 0.04238 |
| cg13652653 | *TMEM33* | 55161 | 0.10 | 0.04238 |
| cg15311933 | *PQLC2* | 54896 | 0.10 | 0.04238 |
| cg15972294 | *GNAI2* | 2771 | 0.10 | 0.04238 |
| cg26738010 | *CETN1* | 1068 | -0.51 | 0.04238 |
| cg19890858 | *PP2447* | 80305 | 0.08 | 0.04238 |
| cg21684385 | *PEX3* | 8504 | 0.10 | 0.04239 |
| cg22933847 | *MRGPRF* | 219928 | 0.42 | 0.04239 |
| cg26456957 | *PPP1R12C* | 54776 | 0.11 | 0.04240 |
| cg21872129 | *PTP4A1* | 7803 | 0.09 | 0.04243 |
| cg00903375 | *SBDS* | 51119 | 0.07 | 0.04244 |
| cg01214209 | *MANEAL* | 149175 | -0.28 | 0.04244 |
| cg02409282 | *ULBP1* | 80329 | 0.08 | 0.04244 |
| cg03751813 | *ZNF585B* | 92285 | 0.09 | 0.04244 |
| cg09395732 | *SLC6A11* | 6538 | -0.43 | 0.04244 |
| cg13002939 | *PDGFRA* | 5156 | 0.13 | 0.04244 |
| cg18758482 | *ZBTB8OS* | 339487 | 0.15 | 0.04244 |
| cg26287783 | *C6orf64* | 55776 | 0.11 | 0.04244 |
| cg24262376 | *SCNM1* | 79005 | -0.30 | 0.04244 |
| cg00199549 | *MCM5* | 4174 | -0.47 | 0.04245 |
| cg00971396 | *POLR2H* | 5437 | 0.08 | 0.04245 |
| cg04941721 | *EREG* | 2069 | 0.09 | 0.04245 |
| cg09600520 | *CDYL* | 9425 | 0.40 | 0.04245 |
| cg12205230 | *TPM3* | 7170 | 0.42 | 0.04245 |
| cg16628918 | *GPR172A* | 79581 | -0.39 | 0.04245 |
| cg19532954 | *ID3* | 3399 | 0.38 | 0.04245 |
| cg21028326 | *TUBGCP6* | 85378 | 0.09 | 0.04245 |
| cg21245135 | *PGAM1* | 5223 | 0.12 | 0.04245 |
| cg21633698 | *THY1* | 7070 | -0.39 | 0.04245 |
| cg21700070 | *CNOT3* | 4849 | 0.10 | 0.04245 |
| cg22262549 | *POLR3C* | 10623 | 0.10 | 0.04245 |
| cg22534509 | *GPR81* | 27198 | -0.32 | 0.04245 |
| cg25511807 | *MMP7* | 4316 | 0.32 | 0.04245 |
| cg02142461 | *LYAR* | 55646 | -0.29 | 0.04245 |
| cg04371779 | *C14orf118* | 55668 | 0.09 | 0.04245 |
| cg10705951 | *TAOK2* | 9344 | 0.09 | 0.04245 |
| cg12297221 | *ART5* | 116969 | 0.10 | 0.04245 |
| cg18283554 | *U1SNRNPBP* | 11066 | 0.13 | 0.04245 |
| cg19534945 | *LCN12* | 286256 | -0.10 | 0.04245 |
| cg20259534 | *BUB1B* | 701 | 0.09 | 0.04245 |
| cg27512828 | *UVRAG* | 7405 | 0.09 | 0.04245 |
| cg07935151 | *LSM2* | 57819 | 0.11 | 0.04245 |
| cg00819696 | *TIMELESS* | 8914 | 0.08 | 0.04245 |
| cg02486845 | *C6orf166* | 55122 | 0.09 | 0.04245 |
| cg06684910 | *POLR3E* | 55718 | 0.09 | 0.04245 |
| cg07934026 | *ABCA11* | 79963 | 0.12 | 0.04245 |
| cg20114394 | *FSTL1* | 11167 | -0.43 | 0.04245 |
| cg01007201 | *CLEC4A* | 50856 | 0.32 | 0.04247 |
| cg02440177 | *ZNF702* | 79986 | 0.09 | 0.04247 |
| cg06006444 | *UBE2D3* | 7323 | 0.08 | 0.04247 |
| cg07899016 | *PCDHB12* | 56124 | -0.24 | 0.04247 |
| cg08837884 | *LRG1* | 116844 | 0.24 | 0.04247 |
| cg12641660 | *TTF2* | 8458 | 0.09 | 0.04247 |
| cg14853515 | *LSM5* | 23658 | 0.09 | 0.04247 |
| cg15344726 | *GJA3* | 2700 | -0.52 | 0.04247 |
| cg00441382 | *STOML1* | 9399 | 0.07 | 0.04247 |
| cg01136458 | *CSMD1* | 64478 | -0.23 | 0.04247 |
| cg02303361 | *AP2S1* | 1175 | 0.07 | 0.04247 |
| cg04098585 | *CD28* | 940 | 0.32 | 0.04247 |
| cg05945622 | *YIF1A* | 10897 | 0.07 | 0.04247 |
| cg07908874 | *TUBGCP2* | 10844 | -0.46 | 0.04247 |
| cg08254263 | *GDPD2* | 54857 | -0.11 | 0.04247 |
| cg11827101 | *LOC339789* | 339789 | 0.26 | 0.04247 |
| cg12164282 | *PXDN* | 7837 | 0.13 | 0.04247 |
| cg12937807 | *LPHN1* | 22859 | 0.11 | 0.04247 |
| cg13588835 | *LGALS7* | 3963 | 0.32 | 0.04247 |
| cg14116596 | *TBXAS1* | 6916 | 0.19 | 0.04247 |
| cg15109207 | *PLA2G4C* | 8605 | -0.37 | 0.04247 |
| cg15900519 | *RPL32* | 6161 | 0.12 | 0.04247 |
| cg17963089 | *PHPT1* | 29085 | 0.16 | 0.04247 |
| cg18085206 | *CRK* | 1398 | 0.08 | 0.04247 |
| cg20264731 | *POLB* | 5423 | 0.11 | 0.04247 |
| cg20868410 | *HBEGF* | 1839 | 0.10 | 0.04247 |
| cg21849289 | *C1orf123* | 54987 | 0.09 | 0.04247 |
| cg23097006 | *VSX1* | 30813 | 0.12 | 0.04247 |
| cg23588553 | *SHMT2* | 6472 | 0.13 | 0.04247 |
| cg23854921 | *AFTIPHILIN* | 54812 | 0.10 | 0.04247 |
| cg24515202 | *TTC9C* | 283237 | 0.12 | 0.04247 |
| cg26910651 | *PPP2CB* | 5516 | 0.10 | 0.04247 |
| cg00151607 | *GALNS* | 2588 | 0.11 | 0.04248 |
| cg03704031 | *INGX* | 27160 | 0.12 | 0.04248 |
| cg07342016 | *RAB33A* | 9363 | 0.13 | 0.04248 |
| cg08680731 | *VPS41* | 27072 | 0.10 | 0.04248 |
| cg10574499 | *UNQ2446* | 123904 | -0.49 | 0.04248 |
| cg04558861 | *F25965* | 55957 | 0.08 | 0.04248 |
| cg05590294 | *CD69* | 969 | 0.31 | 0.04248 |
| cg06643227 | *C9orf84* | 158401 | -0.43 | 0.04248 |
| cg07293947 | *SFXN1* | 94081 | 0.08 | 0.04248 |
| cg14002682 | *RBJ* | 51277 | 0.11 | 0.04248 |
| cg21087137 | *MGC26856* | 256710 | 0.07 | 0.04248 |
| cg25341032 | *ADD3* | 120 | -0.29 | 0.04248 |
| cg02330106 | *MGMT* | 4255 | -0.38 | 0.04248 |
| cg03011500 | *CGN* | 57530 | 0.11 | 0.04248 |
| cg03942436 | *ZNF688* | 146542 | 0.10 | 0.04248 |
| cg04737046 | *ABCB4* | 5244 | -0.11 | 0.04248 |
| cg06333435 | *POLR3D* | 661 | 0.12 | 0.04248 |
| cg10128868 | *PDIA4* | 9601 | 0.07 | 0.04248 |
| cg13577076 | *PRKAR1B* | 5575 | 0.13 | 0.04248 |
| cg09546307 | *CLEC4D* | 338339 | -0.12 | 0.04248 |
| cg25141995 | *VDAC1* | 7416 | -0.39 | 0.04248 |
| cg21065990 | *TASP1* | 55617 | 0.08 | 0.04249 |
| cg25876934 | *WEE1* | 7465 | 0.09 | 0.04249 |
| cg11212609 | *DULLARD* | 23399 | 0.06 | 0.04250 |
| cg10639440 | *SCRN1* | 9805 | 0.11 | 0.04253 |
| cg17293641 | *TNFRSF11B* | 4982 | 0.10 | 0.04253 |
| cg14850026 | *ECAT11* | 54596 | -0.39 | 0.04253 |
| cg04882894 | *IGSF1* | 3547 | 0.13 | 0.04255 |
| cg17663463 | *ZIM2* | 23619 | -0.22 | 0.04255 |
| cg11206634 | *SFT2D3* | 84826 | -0.36 | 0.04256 |
| cg04402875 | *RET* | 5979 | 0.09 | 0.04256 |
| cg10891025 | *EIF3S1* | 8669 | 0.07 | 0.04257 |
| cg03406535 | *RBP7* | 116362 | 0.11 | 0.04257 |
| cg03634982 | *MS4A4A* | 51338 | 0.42 | 0.04257 |
| cg21660130 | *CLCNKB* | 1188 | -0.09 | 0.04257 |
| cg08491025 | *SLC16A1* | 6566 | 0.11 | 0.04258 |
| cg01783386 | *RFT1* | 91869 | 0.09 | 0.04258 |
| cg02092466 | *NTSR2* | 23620 | -0.32 | 0.04258 |
| cg03747695 | *ZNF227* | 7770 | 0.13 | 0.04258 |
| cg04720330 | *PHLDA2* | 7262 | 0.09 | 0.04258 |
| cg07545925 | *CD3G* | 917 | 0.11 | 0.04258 |
| cg19346193 | *UROS* | 7390 | -0.38 | 0.04258 |
| cg21680717 | *AKAP8* | 10270 | 0.07 | 0.04258 |
| cg26137790 | *ACAA2* | 10449 | 0.07 | 0.04258 |
| cg26599006 | *GSCL* | 2928 | 0.09 | 0.04258 |
| cg00025991 | *DIP2C* | 22982 | -0.24 | 0.04259 |
| cg00359325 | *USP4* | 7375 | 0.07 | 0.04259 |
| cg01940974 | *MTAP* | 4507 | 0.09 | 0.04259 |
| cg04970117 | *SLC6A20* | 54716 | 0.11 | 0.04259 |
| cg06043042 | *NEUROG3* | 50674 | 0.07 | 0.04259 |
| cg06270401 | *DYRK4* | 8798 | 0.33 | 0.04259 |
| cg07774025 | *HSD17B7* | 51478 | 0.09 | 0.04259 |
| cg07912181 | *HSD17B12* | 51144 | 0.09 | 0.04259 |
| cg08620470 | *SLC25A16* | 8034 | -0.54 | 0.04259 |
| cg10791260 | *TNFSF15* | 9966 | 0.43 | 0.04259 |
| cg14617642 | *H2AFY2* | 55506 | 0.08 | 0.04259 |
| cg19292008 | *FOXE1* | 2304 | 0.09 | 0.04259 |
| cg22467071 | *PECAM1* | 5175 | 0.39 | 0.04259 |
| cg23881601 | *C1orf66* | 51093 | 0.07 | 0.04259 |
| cg25689649 | *FLJ22709* | 79629 | -0.29 | 0.04259 |
| cg25729765 | *SP3* | 6670 | 0.13 | 0.04259 |
| cg26415655 | *IL31RA* | 133396 | 0.19 | 0.04259 |
| cg24705286 | *JMJD5* | 79831 | 0.08 | 0.04260 |
| cg10220156 | *KIF13B* | 23303 | 0.13 | 0.04261 |
| cg06422910 | *COPG2* | 26958 | 0.11 | 0.04261 |
| cg10421192 | *CTAGE1* | 64693 | -0.42 | 0.04263 |
| cg13888886 | *SPO11* | 23626 | -0.45 | 0.04264 |
| cg09194650 | *TCHP* | 84260 | 0.11 | 0.04264 |
| cg21692936 | *SLC3A2* | 6520 | 0.09 | 0.04264 |
| cg23036555 | *PGD* | 5226 | 0.09 | 0.04264 |
| cg25261908 | *TRPM4* | 54795 | 0.11 | 0.04264 |
| cg00977690 | *ZFX* | 7543 | 0.12 | 0.04264 |
| cg19238398 | *DNAJC18* | 202052 | 0.13 | 0.04264 |
| cg20322876 | *FLJ45803* | 399948 | 0.07 | 0.04264 |
| cg25000382 | *LOC124216* | 124216 | 0.50 | 0.04264 |
| cg10821722 | *MAP4K2* | 5871 | -0.45 | 0.04264 |
| cg12556325 | *C7orf29* | 113763 | 0.14 | 0.04265 |
| cg05412531 | *C8orf78* | 157376 | -0.33 | 0.04265 |
| cg08899626 | *LDB2* | 9079 | 0.24 | 0.04266 |
| cg06711560 | *MAMDC4* | 158056 | -0.08 | 0.04266 |
| cg04898443 | *HDLBP* | 3069 | 0.12 | 0.04267 |
| cg06320982 | *FLJ10815* | 55238 | 0.13 | 0.04267 |
| cg21575929 | *ADRA1B* | 147 | -0.53 | 0.04267 |
| cg24435704 | *PARP12* | 64761 | -0.37 | 0.04267 |
| cg25665697 | *LCTL* | 197021 | 0.11 | 0.04267 |
| cg17718302 | *HIST1H3J* | 8356 | 0.09 | 0.04267 |
| cg02567144 | *EFHC1* | 114327 | 0.10 | 0.04267 |
| cg11185569 | *CHN2* | 1124 | 0.12 | 0.04267 |
| cg11738543 | *SOCS2* | 8835 | 0.09 | 0.04267 |
| cg05750321 | *CGNL1* | 84952 | -0.46 | 0.04268 |
| cg17709466 | *OBFC2B* | 79035 | 0.11 | 0.04268 |
| cg26170660 | *GPX5* | 2880 | 0.36 | 0.04268 |
| cg00008493 | *COX8C* | 341947 | -0.49 | 0.04268 |
| cg01169624 | *SGCE* | 8910 | -0.21 | 0.04268 |
| cg04118154 | *TMEM53* | 79639 | 0.10 | 0.04268 |
| cg05044994 | *FLJ42393* | 401105 | 0.11 | 0.04268 |
| cg05306006 | *RPE* | 6120 | 0.09 | 0.04268 |
| cg06425556 | *NCOA6* | 23054 | 0.08 | 0.04268 |
| cg09656405 | *RPLP1* | 6176 | 0.09 | 0.04268 |
| cg09874752 | *SFRP5* | 6425 | 0.09 | 0.04268 |
| cg10474368 | *SPCS2* | 9789 | 0.08 | 0.04268 |
| cg11399527 | *PDP2* | 57546 | 0.11 | 0.04268 |
| cg12910797 | *HOXB3* | 3213 | -0.42 | 0.04268 |
| cg13469311 | *PGRMC2* | 10424 | 0.11 | 0.04268 |
| cg13766096 | *ING1* | 3621 | 0.07 | 0.04268 |
| cg13810225 | *MYH9* | 4627 | 0.14 | 0.04268 |
| cg14365123 | *ARPM2* | 140625 | -0.08 | 0.04268 |
| cg14871544 | *DSG3* | 1830 | -0.16 | 0.04268 |
| cg15411984 | *LRRC23* | 10233 | 0.08 | 0.04268 |
| cg15480941 | *PIM2* | 11040 | 0.10 | 0.04268 |
| cg15541230 | *COG1* | 9382 | 0.09 | 0.04268 |
| cg15572745 | *NRXN3* | 9369 | 0.19 | 0.04268 |
| cg15836660 | *EDNRB* | 1910 | 0.08 | 0.04268 |
| cg16823737 | *CKAP5* | 9793 | 0.08 | 0.04268 |
| cg17749384 | *MPP7* | 143098 | 0.11 | 0.04268 |
| cg19402885 | *PTPRO* | 5800 | 0.14 | 0.04268 |
| cg19618706 | *BGN* | 633 | 0.22 | 0.04268 |
| cg21586107 | *TMSB10* | 9168 | 0.09 | 0.04268 |
| cg23285573 | *PON2* | 5445 | 0.07 | 0.04268 |
| cg23705973 | *HIST1H1D* | 3007 | 0.08 | 0.04268 |
| cg25371560 | *MED18* | 54797 | 0.09 | 0.04268 |
| cg25438440 | *CLDND1* | 56650 | 0.10 | 0.04268 |
| cg26499286 | *KRTAP17-1* | 83902 | -0.25 | 0.04268 |
| cg27341860 | *OR2L13* | 284521 | -0.51 | 0.04268 |
| cg22685251 | *MAP3K4* | 4216 | 0.14 | 0.04274 |
| cg08585897 | *TERF2IP* | 54386 | -0.32 | 0.04274 |
| cg20845050 | *ZNF7* | 7553 | -0.34 | 0.04274 |
| cg13752005 | *CCDC64* | 92558 | 0.10 | 0.04274 |
| cg23983340 | *CD46* | 4179 | 0.10 | 0.04274 |
| cg20340596 | *HPS1* | 3257 | -0.39 | 0.04276 |
| cg12986021 | *DIRAS3* | 9077 | -0.34 | 0.04278 |
| cg20227213 | *GOLGA4* | 2803 | 0.08 | 0.04278 |
| cg01806928 | *SYT9* | 143425 | -0.49 | 0.04279 |
| cg17788682 | *ARNT2* | 9915 | 0.09 | 0.04279 |
| cg04534765 | *GALR1* | 2587 | 0.11 | 0.04280 |
| cg10057218 | *GSDML* | 55876 | -0.14 | 0.04280 |
| cg11884792 | *OBFC2A* | 64859 | 0.12 | 0.04280 |
| cg15832292 | *MANEA* | 79694 | 0.13 | 0.04280 |
| cg21529533 | *HLA-G* | 3135 | 0.08 | 0.04280 |
| cg23824815 | *ZNF333* | 84449 | 0.10 | 0.04280 |
| cg24371225 | *MGC42105* | 167359 | 0.08 | 0.04280 |
| cg27286999 | *OLFM1* | 10439 | 0.08 | 0.04280 |
| cg23640701 | *ACVRL1* | 94 | -0.45 | 0.04281 |
| cg24471268 | *SPSB2* | 84727 | 0.09 | 0.04281 |
| cg12813922 | *RAB3GAP1* | 22930 | 0.09 | 0.04281 |
| cg02304930 | *SERTAD4* | 56256 | 0.11 | 0.04282 |
| cg05417985 | *ENPP1* | 5167 | 0.07 | 0.04282 |
| cg18109231 | *RFX2* | 5990 | 0.12 | 0.04282 |
| cg18313702 | *EIF2B5* | 8893 | 0.07 | 0.04282 |
| cg21087043 | *DCX* | 1641 | 0.10 | 0.04282 |
| cg02451362 | *MAFG* | 4097 | 0.09 | 0.04283 |
| cg07322981 | *RNF103* | 7844 | 0.08 | 0.04283 |
| cg08331313 | *SPARC* | 6678 | 0.09 | 0.04283 |
| cg16860587 | *ELAVL1* | 1994 | 0.12 | 0.04283 |
| cg17316750 | *FAM82C* | 55177 | 0.10 | 0.04283 |
| cg22492966 | *JMJD1C* | 221037 | 0.31 | 0.04283 |
| cg24910675 | *ENG* | 2022 | 0.35 | 0.04283 |
| cg17560332 | *BOLL* | 66037 | -0.35 | 0.04283 |
| cg08996502 | *SCRIB* | 23513 | 0.09 | 0.04286 |
| cg12799895 | *NPTX2* | 4885 | 0.10 | 0.04286 |
| cg18676764 | *WDR40A* | 25853 | -0.44 | 0.04286 |
| cg12090242 | *ATP6V1E1* | 529 | 0.14 | 0.04287 |
| cg16689634 | *CYP4X1* | 260293 | 0.12 | 0.04287 |
| cg14826683 | *SPRR2D* | 6703 | 0.19 | 0.04287 |
| cg17777592 | *CAB39L* | 81617 | 0.25 | 0.04288 |
| cg13468685 | *PLEK* | 5341 | 0.37 | 0.04289 |
| cg00600110 | *FAM9A* | 171482 | -0.54 | 0.04289 |
| cg10564498 | *AXL* | 558 | 0.23 | 0.04289 |
| cg07410075 | *CCDC6* | 8030 | 0.11 | 0.04289 |
| cg11585425 | *ACTR2* | 10097 | 0.10 | 0.04289 |
| cg00540544 | *CSRP1* | 1465 | 0.10 | 0.04290 |
| cg02181494 | *ARSA* | 410 | 0.09 | 0.04290 |
| cg05938725 | *NUAK2* | 81788 | 0.09 | 0.04290 |
| cg07105440 | *CISH* | 1154 | 0.13 | 0.04290 |
| cg09305478 | *MGC15416* | 84331 | -0.21 | 0.04290 |
| cg10751811 | *CYP4Z1* | 199974 | 0.17 | 0.04290 |
| cg13435792 | *C12orf46* | 121506 | 0.17 | 0.04290 |
| cg13905388 | *CDCA4* | 55038 | -0.34 | 0.04290 |
| cg16677885 | *FASTK* | 10922 | 0.08 | 0.04290 |
| cg18533466 | *RREB1* | 6239 | 0.08 | 0.04290 |
| cg19221369 | *TAF13* | 6884 | 0.09 | 0.04290 |
| cg20047230 | *CP110* | 9738 | 0.12 | 0.04290 |
| cg26282384 | *PCDHGB4* | 8641 | 0.08 | 0.04290 |
| cg05494459 | *FTSJ1* | 24140 | 0.07 | 0.04291 |
| cg25902460 | *STRBP* | 55342 | 0.12 | 0.04291 |
| cg04680304 | *RRM1* | 6240 | 0.09 | 0.04291 |
| cg06616147 | *ICK* | 22858 | 0.16 | 0.04291 |
| cg08373610 | *C2orf11* | 130132 | 0.23 | 0.04291 |
| cg13319664 | *C21orf55* | 54943 | 0.11 | 0.04292 |
| cg20633621 | *GAS2L1* | 10634 | 0.11 | 0.04292 |
| cg19384697 | *UPK3B* | 80761 | 0.19 | 0.04293 |
| cg01091565 | *MESP1* | 55897 | -0.33 | 0.04296 |
| cg23772500 | *PSMD3* | 5709 | 0.11 | 0.04296 |
| cg12439899 | *TFAP2A* | 7020 | 0.11 | 0.04296 |
| cg02062650 | *HCFC1R1* | 54985 | 0.11 | 0.04296 |
| cg08176694 | *PITPNM2* | 57605 | 0.47 | 0.04296 |
| cg12107692 | *BCL2L12* | 83596 | 0.12 | 0.04296 |
| cg23909633 | *IL24* | 11009 | -0.08 | 0.04296 |
| cg24664957 | *VPS37B* | 79720 | -0.27 | 0.04296 |
| cg06168050 | *ZNF207* | 7756 | -0.50 | 0.04298 |
| cg00733682 | *ZNF44* | 51710 | 0.09 | 0.04298 |
| cg00943909 | *GNAS* | 2778 | -0.38 | 0.04298 |
| cg07008350 | *TCEB3C* | 162699 | -0.37 | 0.04298 |
| cg09113530 | *MALL* | 7851 | -0.33 | 0.04301 |
| cg20369763 | *FNDC4* | 64838 | 0.10 | 0.04301 |
| cg05286874 | *ABCA1* | 19 | 0.08 | 0.04303 |
| cg08124030 | *TM4SF1* | 4071 | 0.22 | 0.04303 |
| cg00620024 | *PPP6C* | 5537 | -0.30 | 0.04305 |
| cg00873037 | *NEIL2* | 252969 | 0.08 | 0.04305 |
| cg11903880 | *GSTM4* | 2948 | -0.29 | 0.04305 |
| cg14500718 | *TMEM79* | 84283 | -0.21 | 0.04305 |
| cg25886875 | *PSMC4* | 5704 | 0.09 | 0.04305 |
| cg02902770 | *RARRES2* | 5919 | -0.39 | 0.04306 |
| cg05740244 | *LDHC* | 3948 | -0.27 | 0.04306 |
| cg11371160 | *UBL4A* | 8266 | 0.12 | 0.04306 |
| cg11466908 | *AUH* | 549 | 0.08 | 0.04306 |
| cg12263485 | *CBX5* | 23468 | 0.12 | 0.04306 |
| cg15881088 | *ARMET* | 7873 | 0.07 | 0.04306 |
| cg17788832 | *PSKH2* | 85481 | -0.37 | 0.04306 |
| cg18923230 | *RPL10* | 6134 | 0.12 | 0.04306 |
| cg21880328 | *CTTNBP2* | 83992 | -0.20 | 0.04306 |
| cg23470272 | *GPSN2* | 9524 | 0.10 | 0.04306 |
| cg26271255 | *ARHGAP12* | 94134 | 0.08 | 0.04306 |
| cg16028934 | *TP53BP2* | 7159 | 0.10 | 0.04306 |
| cg03109701 | *TMEM118* | 84900 | -0.34 | 0.04309 |
| cg07686479 | *LMX1A* | 4009 | 0.07 | 0.04309 |
| cg09891761 | *F5* | 2153 | 0.09 | 0.04309 |
| cg12555334 | *PSPH* | 5723 | -0.28 | 0.04309 |
| cg18931750 | *MGC33407* | 284382 | -0.32 | 0.04309 |
| cg20083730 | *MT1E* | 4493 | 0.08 | 0.04309 |
| cg20444256 | *CKM* | 1158 | -0.31 | 0.04309 |
| cg25355803 | *MARVELD1* | 83742 | -0.40 | 0.04309 |
| cg00512031 | *CYTL1* | 54360 | -0.08 | 0.04309 |
| cg00594952 | *RIMS3* | 9783 | -0.34 | 0.04309 |
| cg00630164 | *KCNQ4* | 9132 | 0.17 | 0.04309 |
| cg01952458 | *ATP6V1B2* | 526 | 0.08 | 0.04309 |
| cg02351381 | *C12orf34* | 84915 | 0.28 | 0.04309 |
| cg03549571 | *DHH* | 50846 | 0.11 | 0.04309 |
| cg03680758 | *SLC2A4RG* | 56731 | 0.08 | 0.04309 |
| cg03782727 | *FFAR1* | 2864 | -0.57 | 0.04309 |
| cg07621046 | *C10orf82* | 143379 | -0.41 | 0.04309 |
| cg13745346 | *CBFA2T3* | 863 | 0.34 | 0.04309 |
| cg14580567 | *HHIP* | 64399 | 0.21 | 0.04309 |
| cg16468910 | *SNX4* | 8723 | -0.40 | 0.04309 |
| cg17351385 | *ALKBH6* | 84964 | 0.12 | 0.04309 |
| cg17699374 | *MGC35206* | 339669 | 0.43 | 0.04309 |
| cg18340214 | *MRPL33* | 9553 | 0.07 | 0.04309 |
| cg19297232 | *SMPD3* | 55512 | -0.25 | 0.04309 |
| cg21022247 | *BIN2* | 51411 | -0.36 | 0.04309 |
| cg21516478 | *GPX3* | 2878 | 0.12 | 0.04309 |
| cg23912823 | *NAB2* | 4665 | 0.10 | 0.04309 |
| cg24478145 | *SCFD1* | 23256 | 0.11 | 0.04309 |
| cg25215340 | *CDKN1C* | 1028 | 0.09 | 0.04309 |
| cg27518692 | *WDR68* | 10238 | 0.15 | 0.04309 |
| cg00223186 | *ACBD6* | 84320 | 0.07 | 0.04309 |
| cg02675896 | *LOC90355* | 90355 | 0.09 | 0.04309 |
| cg06720956 | *ONECUT1* | 3175 | 0.08 | 0.04309 |
| cg11344614 | *JAM2* | 58494 | 0.35 | 0.04309 |
| cg16232979 | *TPM4* | 7171 | 0.12 | 0.04309 |
| cg16341373 | *CDCA8* | 55143 | 0.14 | 0.04309 |
| cg17641104 | *PCTK2* | 5128 | 0.07 | 0.04309 |
| cg22403851 | *PDIA6* | 10130 | 0.10 | 0.04309 |
| cg25346972 | *C14orf112* | 51241 | 0.09 | 0.04309 |
| cg00081975 | *ABCC3* | 8714 | 0.09 | 0.04310 |
| cg23029193 | *FLJ39155* | 133584 | 0.09 | 0.04310 |
| cg06421800 | *CDKN2B* | 1030 | -0.41 | 0.04310 |
| cg26265060 | *SON* | 6651 | 0.12 | 0.04310 |
| cg21570818 | *FUT5* | 2527 | -0.10 | 0.04311 |
| cg13707395 | *ADAMTS15* | 170689 | 0.09 | 0.04311 |
| cg15160445 | *GNAS* | 2778 | -0.27 | 0.04311 |
| cg16776350 | *CD84* | 8832 | 0.32 | 0.04311 |
| cg03382797 | *C9orf98* | 158067 | 0.18 | 0.04311 |
| cg04886198 | *ALG3* | 10195 | 0.13 | 0.04311 |
| cg02973971 | *CXorf40B* | 541578 | 0.10 | 0.04311 |
| cg03387723 | *SCMH1* | 22955 | -0.36 | 0.04311 |
| cg03558807 | *TIGA1* | 114915 | -0.39 | 0.04311 |
| cg08332868 | *RBM19* | 9904 | 0.08 | 0.04311 |
| cg10784813 | *SOCS1* | 8651 | -0.41 | 0.04311 |
| cg13849825 | *CCNB1* | 891 | -0.45 | 0.04311 |
| cg14405589 | *TUBGCP3* | 10426 | 0.10 | 0.04311 |
| cg16787352 | *ANKRD9* | 122416 | -0.37 | 0.04311 |
| cg17804302 | *NKD1* | 85407 | 0.06 | 0.04311 |
| cg18519564 | *RCL1* | 10171 | 0.10 | 0.04311 |
| cg19008809 | *SFMBT1* | 51460 | 0.09 | 0.04311 |
| cg19221044 | *TAP2* | 6891 | 0.07 | 0.04311 |
| cg22024281 | *EXT1* | 2131 | 0.08 | 0.04311 |
| cg23920917 | *CPSF6* | 11052 | 0.10 | 0.04311 |
| cg24877842 | *TRAK1* | 22906 | 0.23 | 0.04311 |
| cg27076799 | *POLD3* | 10714 | 0.10 | 0.04311 |
| cg13735697 | *MYOG* | 4656 | -0.08 | 0.04313 |
| cg26911140 | *RPL8* | 6132 | 0.10 | 0.04313 |
| cg23448096 | *LYSMD4* | 145748 | 0.12 | 0.04313 |
| cg25556101 | *RPS19BP1* | 91582 | 0.11 | 0.04315 |
| cg19369556 | *CDH13* | 1012 | 0.08 | 0.04322 |
| cg00410419 | *COMMD6* | 170622 | 0.07 | 0.04323 |
| cg01137065 | *FOXK2* | 3607 | 0.08 | 0.04323 |
| cg01325515 | *CTAG2* | 30848 | -0.53 | 0.04323 |
| cg01678091 | *MAGEL2* | 54551 | -0.48 | 0.04323 |
| cg02397720 | *RAB17* | 64284 | 0.41 | 0.04323 |
| cg03640944 | *KIAA1754* | 85450 | 0.44 | 0.04323 |
| cg04603031 | *CHRNA3* | 1136 | 0.08 | 0.04323 |
| cg05365670 | *AYTL2* | 79888 | -0.33 | 0.04323 |
| cg06044117 | *CEP57* | 9702 | 0.12 | 0.04323 |
| cg06543761 | *PPM2C* | 54704 | 0.11 | 0.04323 |
| cg06585027 | *PIK4CB* | 5298 | 0.12 | 0.04323 |
| cg07314414 | *SAP130* | 79595 | 0.12 | 0.04323 |
| cg07572435 | *LY6D* | 8581 | -0.09 | 0.04323 |
| cg08367838 | *YIPF5* | 81555 | 0.11 | 0.04323 |
| cg09079275 | *ADAM19* | 8728 | 0.10 | 0.04323 |
| cg09313439 | *CDH2* | 1000 | 0.08 | 0.04323 |
| cg09514188 | *TOB1* | 10140 | 0.11 | 0.04323 |
| cg09577651 | *SIRPB1* | 10326 | 0.34 | 0.04323 |
| cg09971811 | *CST7* | 8530 | 0.36 | 0.04323 |
| cg10590292 | *BIN2* | 51411 | 0.11 | 0.04323 |
| cg10935064 | *SYCP3* | 50511 | -0.56 | 0.04323 |
| cg12261786 | *C10orf116* | 10974 | -0.37 | 0.04323 |
| cg14059963 | *GPR19* | 2842 | -0.09 | 0.04323 |
| cg14353162 | *TMEM55B* | 90809 | 0.10 | 0.04323 |
| cg15645605 | *MRPL35* | 51318 | 0.07 | 0.04323 |
| cg16509569 | *HEM1* | 3071 | 0.33 | 0.04323 |
| cg16540704 | *ASZ1* | 136991 | -0.41 | 0.04323 |
| cg17387916 | *RPL28* | 6158 | 0.09 | 0.04323 |
| cg17705081 | *GPKOW* | 27238 | -0.38 | 0.04323 |
| cg18156583 | *IL18RAP* | 8807 | 0.21 | 0.04323 |
| cg19744131 | *PRUNE* | 58497 | 0.13 | 0.04323 |
| cg20342105 | *BSCL2* | 26580 | -0.34 | 0.04323 |
| cg22937320 | *C9orf138* | 158297 | 0.56 | 0.04323 |
| cg23248452 | *TAS2R1* | 50834 | 0.16 | 0.04323 |
| cg23326301 | *ADAM30* | 11085 | -0.28 | 0.04323 |
| cg23557926 | *CFH* | 3075 | 0.29 | 0.04323 |
| cg24034992 | *YIPF6* | 286451 | 0.12 | 0.04323 |
| cg24449371 | *CIB2* | 10518 | 0.07 | 0.04323 |
| cg24675098 | *FLJ23577* | 79925 | 0.09 | 0.04323 |
| cg27584171 | *KIAA0256* | 9728 | 0.13 | 0.04323 |
| cg25580018 | *ADAMTSL2* | 9719 | 0.12 | 0.04325 |
| cg19100810 | *RPL32* | 6161 | 0.10 | 0.04325 |
| cg20798152 | *CART* | 9607 | -0.19 | 0.04325 |
| cg23190203 | *SFXN3* | 81855 | 0.08 | 0.04325 |
| cg24844534 | *C1QTNF1* | 114897 | 0.09 | 0.04325 |
| cg04101379 | *DZIP1* | 22873 | 0.08 | 0.04326 |
| cg05668853 | *RAB34* | 83871 | -0.36 | 0.04326 |
| cg08811349 | *ZHX1* | 11244 | 0.11 | 0.04326 |
| cg09076077 | *FLJ33860* | 284756 | 0.34 | 0.04326 |
| cg11554937 | *HSD17B3* | 3293 | -0.07 | 0.04326 |
| cg15296858 | *PPM1G* | 5496 | -0.45 | 0.04326 |
| cg15501381 | *NGB* | 58157 | -0.31 | 0.04326 |
| cg15679098 | *RAPSN* | 5913 | -0.08 | 0.04326 |
| cg17261830 | *NEK9* | 91754 | 0.08 | 0.04326 |
| cg18556220 | *FBXW5* | 54461 | 0.09 | 0.04326 |
| cg21561173 | *C21orf81* | 114035 | -0.15 | 0.04326 |
| cg21946698 | *RGS11* | 8786 | -0.39 | 0.04326 |
| cg22679120 | *SNX8* | 29886 | -0.27 | 0.04326 |
| cg22821834 | *GPR135* | 64582 | 0.10 | 0.04326 |
| cg23517605 | *TUBB2B* | 347733 | -0.35 | 0.04326 |
| cg24912023 | *LOC205251* | 205251 | 0.19 | 0.04326 |
| cg03803589 | *STYX* | 6815 | 0.11 | 0.04328 |
| cg07854244 | *IHPK2* | 51447 | 0.08 | 0.04328 |
| cg00766729 | *LOC147808* | 147808 | 0.10 | 0.04329 |
| cg01214321 | *DPYSL2* | 1808 | 0.11 | 0.04329 |
| cg03373857 | *WBSCR16* | 81554 | 0.07 | 0.04329 |
| cg04172043 | *RELA* | 5970 | 0.08 | 0.04329 |
| cg05322222 | *MGC33486* | 256472 | -0.20 | 0.04329 |
| cg05397738 | *PGRMC1* | 10857 | -0.43 | 0.04329 |
| cg06438300 | *FAM19A2* | 338811 | 0.13 | 0.04329 |
| cg06790324 | *GRB10* | 2887 | 0.08 | 0.04329 |
| cg11050527 | *ATP6V1H* | 51606 | 0.06 | 0.04329 |
| cg11546621 | *PTGDS* | 5730 | -0.30 | 0.04329 |
| cg12569516 | *TRPS1* | 7227 | 0.10 | 0.04329 |
| cg15926585 | *COMT* | 1312 | -0.38 | 0.04329 |
| cg20360244 | *SLC35E3* | 55508 | 0.07 | 0.04329 |
| cg22485810 | *CENTA2* | 55803 | 0.08 | 0.04329 |
| cg23579062 | *DNAI1* | 27019 | -0.49 | 0.04329 |
| cg05222924 | *WT1* | 7490 | 0.11 | 0.04329 |
| cg17213552 | *DLAT* | 1737 | 0.07 | 0.04329 |
| cg19624775 | *MORC3* | 23515 | 0.10 | 0.04329 |
| cg24139421 | *SLC22A18* | 5002 | 0.10 | 0.04329 |
| cg25906332 | *SLC25A29* | 123096 | 0.12 | 0.04329 |
| cg05156613 | *ACTL7B* | 10880 | -0.46 | 0.04330 |
| cg05910970 | *CYP2A6* | 1548 | -0.08 | 0.04330 |
| cg15227982 | *C10orf26* | 54838 | 0.28 | 0.04330 |
| cg21011830 | *RPL18A* | 6142 | 0.14 | 0.04330 |
| cg24528447 | *NR2F1* | 7025 | 0.11 | 0.04330 |
| cg00597076 | *MYO1C* | 4641 | -0.39 | 0.04330 |
| cg00731459 | *HDAC4* | 9759 | 0.09 | 0.04330 |
| cg03308985 | *DGCR6L* | 85359 | 0.11 | 0.04330 |
| cg03617367 | *C14orf43* | 91748 | 0.10 | 0.04330 |
| cg04824716 | *CCHCR1* | 54535 | -0.45 | 0.04330 |
| cg05129930 | *PFKL* | 5211 | 0.08 | 0.04330 |
| cg06186861 | *ZNF501* | 115560 | 0.12 | 0.04330 |
| cg09214920 | *SLC25A24* | 29957 | 0.13 | 0.04330 |
| cg09283007 | *FAM47B* | 170062 | -0.47 | 0.04330 |
| cg10273210 | *TRIM59* | 286827 | 0.07 | 0.04330 |
| cg10896774 | *C7orf34* | 135927 | 0.20 | 0.04330 |
| cg13771579 | *TCL1B* | 9623 | -0.54 | 0.04330 |
| cg13966710 | *FLJ36116* | 388666 | 0.09 | 0.04330 |
| cg14658900 | *ZNF434* | 54925 | 0.10 | 0.04330 |
| cg15275890 | *SLC25A17* | 10478 | -0.38 | 0.04330 |
| cg15427448 | *BACE1* | 23621 | 0.09 | 0.04330 |
| cg18088775 | *RKHD2* | 51320 | 0.09 | 0.04330 |
| cg18434152 | *PROK1* | 84432 | 0.20 | 0.04330 |
| cg18937321 | *SLC6A9* | 6536 | 0.09 | 0.04330 |
| cg20880234 | *ZNF198* | 7750 | 0.11 | 0.04330 |
| cg21306775 | *FLJ44881* | 400661 | 0.14 | 0.04330 |
| cg23131777 | *ADAM30* | 11085 | -0.35 | 0.04330 |
| cg23269225 | *FBXL4* | 26235 | 0.11 | 0.04330 |
| cg23888423 | *SEPT2* | 4735 | 0.07 | 0.04330 |
| cg26309134 | *ZNF542* | 147947 | 0.09 | 0.04330 |
| cg26358246 | *TMEM60* | 85025 | 0.07 | 0.04330 |
| cg27015174 | *LCMT2* | 9836 | 0.09 | 0.04330 |
| cg27152280 | *NRG2* | 9542 | 0.09 | 0.04330 |
| cg02298612 | *CHST6* | 4166 | -0.44 | 0.04331 |
| cg02701137 | *DLGAP4* | 22839 | -0.08 | 0.04331 |
| cg05607472 | *CSNK1A1* | 1452 | 0.10 | 0.04331 |
| cg06763078 | *KCNC1* | 3746 | 0.12 | 0.04331 |
| cg07231132 | *TMEM92* | 162461 | 0.12 | 0.04331 |
| cg08993267 | *BRCA1* | 672 | 0.09 | 0.04331 |
| cg15965913 | *C1orf121* | 51029 | 0.07 | 0.04331 |
| cg20399252 | *EBPL* | 84650 | -0.50 | 0.04331 |
| cg21771250 | *FAM83F* | 113828 | 0.32 | 0.04331 |
| cg25414165 | *C10orf11* | 83938 | 0.15 | 0.04331 |
| cg26147657 | *FLJ13946* | 92104 | 0.09 | 0.04333 |
| cg22007326 | *TMED2* | 10959 | 0.10 | 0.04334 |
| cg00400263 | *C20orf177* | 63939 | -0.42 | 0.04335 |
| cg14201617 | *FLJ40172* | 285051 | -0.42 | 0.04335 |
| cg11387131 | *KCNJ5* | 3762 | 0.16 | 0.04336 |
| cg00059930 | *RB1* | 5925 | -0.39 | 0.04337 |
| cg08705994 | *RUNX3* | 864 | -0.09 | 0.04337 |
| cg20778294 | *PPRC1* | 23082 | 0.10 | 0.04337 |
| cg08411049 | *SERPINB5* | 5268 | -0.42 | 0.04337 |
| cg07664027 | *RPL13A* | 23521 | -0.41 | 0.04337 |
| cg01245656 | *ASNS* | 440 | 0.09 | 0.04339 |
| cg04525773 | *DBF4* | 10926 | 0.09 | 0.04339 |
| cg10947146 | *XKR6* | 286046 | 0.11 | 0.04339 |
| cg01794711 | *CNDP2* | 55748 | 0.09 | 0.04340 |
| cg09539538 | *C20orf42* | 55612 | -0.28 | 0.04340 |
| cg19184518 | *UNQ9217* | 167555 | 0.10 | 0.04340 |
| cg20358834 | *LRFN4* | 78999 | 0.10 | 0.04340 |
| cg20503329 | *COL15A1* | 1306 | -0.39 | 0.04340 |
| cg24407308 | *DGKZ* | 8525 | 0.11 | 0.04340 |
| cg27061366 | *FARSLA* | 2193 | 0.07 | 0.04340 |
| cg00119079 | *LAMP3* | 27074 | 0.11 | 0.04340 |
| cg01958916 | *SEMA6C* | 10500 | 0.07 | 0.04340 |
| cg02674804 | *REEP6* | 92840 | -0.37 | 0.04340 |
| cg02699167 | *FBXL2* | 25827 | 0.10 | 0.04340 |
| cg03840259 | *GRAP2* | 9402 | 0.30 | 0.04340 |
| cg04109382 | *C20orf10* | 27296 | 0.48 | 0.04340 |
| cg04711050 | *SLC1A1* | 6505 | 0.10 | 0.04340 |
| cg04737319 | *HARS2* | 92675 | 0.12 | 0.04340 |
| cg04880762 | *VAMP1* | 6843 | 0.08 | 0.04340 |
| cg07625840 | *PRSS12* | 8492 | 0.12 | 0.04340 |
| cg07846220 | *LAMA1* | 284217 | 0.08 | 0.04340 |
| cg12572011 | *UBE3A* | 7337 | 0.09 | 0.04340 |
| cg12730381 | *YWHAG* | 7532 | 0.19 | 0.04340 |
| cg13387232 | *FLJ34443* | 285464 | -0.14 | 0.04340 |
| cg13625113 | *ZNF482* | 10773 | 0.11 | 0.04340 |
| cg15812957 | *VCY* | 9084 | -0.51 | 0.04340 |
| cg16824731 | *ILK* | 3611 | 0.09 | 0.04340 |
| cg17520176 | *ZMYM4* | 9202 | 0.12 | 0.04340 |
| cg18616655 | *SSX5* | 6758 | -0.41 | 0.04340 |
| cg18913951 | *TMEM45B* | 120224 | -0.34 | 0.04340 |
| cg19410364 | *P2RX4* | 5025 | -0.20 | 0.04340 |
| cg23696886 | *PDLIM2* | 64236 | 0.11 | 0.04340 |
| cg23867624 | *FOXC1* | 2296 | 0.11 | 0.04340 |
| cg24800887 | *FANCA* | 2175 | -0.40 | 0.04340 |
| cg25437402 | *PICALM* | 8301 | 0.11 | 0.04340 |
| cg26454299 | *PPP3CA* | 5530 | 0.08 | 0.04340 |
| cg05010058 | *CEP68* | 23177 | 0.10 | 0.04341 |
| cg11651717 | *ABCA5* | 23461 | 0.09 | 0.04341 |
| cg15970621 | *PHKG2* | 5261 | 0.10 | 0.04341 |
| cg24402880 | *PLAC8* | 51316 | 0.29 | 0.04341 |
| cg14106632 | *EVI5L* | 115704 | 0.10 | 0.04341 |
| cg18676237 | *SERPINB9* | 5272 | 0.10 | 0.04341 |
| cg26317111 | *UBE2U* | 148581 | -0.53 | 0.04341 |
| cg26682717 | *ATP6V1F* | 9296 | 0.11 | 0.04341 |
| cg02305723 | *SNRPN* | 6638 | -0.18 | 0.04342 |
| cg25384595 | *LILRA1* | 11024 | 0.38 | 0.04342 |
| cg06848073 | *FBXO44* | 93611 | -0.29 | 0.04344 |
| cg06840226 | *GPC1* | 2817 | 0.11 | 0.04345 |
| cg07190485 | *STOM* | 2040 | 0.07 | 0.04345 |
| cg16718094 | *SAP30* | 8819 | 0.09 | 0.04345 |
| cg02657360 | *H19* | 283120 | 0.24 | 0.04346 |
| cg02686662 | *PPAP2C* | 8612 | -0.40 | 0.04346 |
| cg05060602 | *DCTD* | 1635 | 0.12 | 0.04346 |
| cg06542614 | *PDLIM1* | 9124 | -0.24 | 0.04346 |
| cg07654843 | *MYO18B* | 84700 | -0.34 | 0.04346 |
| cg11158729 | *CNNM4* | 26504 | 0.08 | 0.04346 |
| cg15854296 | *MAP3K11* | 4296 | 0.13 | 0.04346 |
| cg20373326 | *HSD17B2* | 3294 | 0.13 | 0.04346 |
| cg21342728 | *GPR24* | 2847 | 0.33 | 0.04346 |
| cg22874695 | *C8orf40* | 114926 | 0.09 | 0.04346 |
| cg23509027 | *CSAG3A* | 389903 | -0.41 | 0.04346 |
| cg27655905 | *C11orf24* | 53838 | 0.14 | 0.04346 |
| cg04368919 | *EFNB1* | 1947 | 0.12 | 0.04347 |
| cg10708675 | *NQO1* | 1728 | 0.07 | 0.04347 |
| cg05951993 | *GYS1* | 2997 | 0.07 | 0.04347 |
| cg11681073 | *CUL1* | 8454 | 0.12 | 0.04347 |
| cg19764555 | *AHNAK* | 79026 | 0.28 | 0.04347 |
| cg22800631 | *TRRAP* | 8295 | 0.15 | 0.04347 |
| cg13744304 | *UNC50* | 25972 | 0.09 | 0.04348 |
| cg20368904 | *TNFAIP2* | 7127 | -0.49 | 0.04349 |
| cg24110063 | *COX6A2* | 1339 | -0.41 | 0.04349 |
| cg02600430 | *ZIC5* | 85416 | -0.36 | 0.04349 |
| cg07076342 | *MEA1* | 4201 | 0.13 | 0.04350 |
| cg11193041 | *GSTZ1* | 2954 | 0.13 | 0.04350 |
| cg12736438 | *RAB20* | 55647 | 0.09 | 0.04350 |
| cg22282672 | *FLJ32921* | 133923 | 0.24 | 0.04350 |
| cg19595170 | *SRF* | 6722 | 0.16 | 0.04351 |
| cg17076921 | *CAMK2G* | 818 | 0.15 | 0.04351 |
| cg24202119 | *CAPSL* | 133690 | 0.24 | 0.04351 |
| cg12130692 | *EPM2A* | 7957 | 0.14 | 0.04351 |
| cg05200311 | *DEK* | 7913 | 0.10 | 0.04351 |
| cg10548978 | *C10orf27* | 219793 | 0.38 | 0.04351 |
| cg13264208 | *SEC61A1* | 29927 | 0.12 | 0.04351 |
| cg13823136 | *ST6GALNAC5* | 81849 | 0.08 | 0.04351 |
| cg15160742 | *C1orf121* | 51029 | 0.08 | 0.04351 |
| cg16112129 | *DRD1* | 1812 | 0.10 | 0.04351 |
| cg16155588 | *CUL4A* | 8451 | -0.23 | 0.04351 |
| cg16218377 | *GPIAP1* | 4076 | 0.07 | 0.04351 |
| cg18043104 | *TMEM50B* | 757 | 0.13 | 0.04351 |
| cg25047001 | *GRIN2A* | 2903 | 0.11 | 0.04351 |
| cg15897310 | *CTGF* | 1490 | 0.16 | 0.04351 |
| cg23026920 | *TP53RK* | 112858 | 0.10 | 0.04353 |
| cg11843760 | *DFFA* | 1676 | 0.08 | 0.04353 |
| cg07654934 | *LXN* | 56925 | 0.07 | 0.04354 |
| cg13313036 | *SLC29A2* | 3177 | 0.10 | 0.04354 |
| cg24354652 | *PTAFR* | 5724 | 0.25 | 0.04354 |
| cg24392574 | *CALML5* | 51806 | -0.54 | 0.04354 |
| cg26774312 | *CAV2* | 858 | 0.08 | 0.04354 |
| cg13597949 | *CKAP1* | 1155 | 0.06 | 0.04356 |
| cg19007602 | *SOCS2* | 8835 | 0.08 | 0.04357 |
| cg23663476 | *LAT* | 27040 | 0.34 | 0.04358 |
| cg05200628 | *CD48* | 962 | 0.29 | 0.04359 |
| cg09275137 | *CXorf12* | 8269 | 0.09 | 0.04359 |
| cg12832565 | *CD160* | 11126 | 0.15 | 0.04359 |
| cg20496643 | *RASL10B* | 91608 | 0.09 | 0.04359 |
| cg24691453 | *S100A4* | 6275 | 0.34 | 0.04359 |
| cg26222407 | *STAG2* | 10735 | 0.13 | 0.04359 |
| cg22244118 | *TTK* | 7272 | 0.09 | 0.04359 |
| cg20616836 | *AES* | 166 | -0.07 | 0.04363 |
| cg05749577 | *KIF23* | 9493 | 0.09 | 0.04363 |
| cg20611872 | *XAGE3* | 170626 | -0.42 | 0.04363 |
| cg21872393 | *MRCL3* | 10627 | 0.06 | 0.04363 |
| cg23657252 | *BMP2K* | 55589 | 0.12 | 0.04363 |
| cg25454755 | *NR4A3* | 8013 | 0.07 | 0.04363 |
| cg18449462 | *COX7B* | 1349 | 0.11 | 0.04364 |
| cg14654385 | *URP2* | 83706 | 0.39 | 0.04364 |
| cg04135543 | *ORF1-FL49* | 84418 | 0.11 | 0.04365 |
| cg08427977 | *C10orf72* | 196740 | -0.37 | 0.04365 |
| cg10591659 | *NYX* | 60506 | 0.20 | 0.04367 |
| cg02141675 | *GALNT10* | 55568 | 0.10 | 0.04367 |
| cg05125838 | *UCN2* | 90226 | 0.27 | 0.04367 |
| cg06367117 | *ALDOC* | 230 | 0.11 | 0.04367 |
| cg09911755 | *OSBPL7* | 114881 | 0.15 | 0.04367 |
| cg12468056 | *MYST3* | 7994 | 0.10 | 0.04367 |
| cg15910208 | *KLK10* | 5655 | 0.11 | 0.04367 |
| cg24349555 | *HECA* | 51696 | 0.08 | 0.04367 |
| cg00332745 | *NOL3* | 8996 | 0.11 | 0.04368 |
| cg04569233 | *CX3CR1* | 1524 | 0.16 | 0.04368 |
| cg04983977 | *GPR25* | 2848 | -0.45 | 0.04368 |
| cg12691366 | *HSPC159* | 29094 | 0.08 | 0.04368 |
| cg14859074 | *CLDN1* | 9076 | 0.10 | 0.04368 |
| cg14986420 | *YIPF6* | 286451 | 0.12 | 0.04368 |
| cg17386433 | *FAM76A* | 199870 | 0.13 | 0.04368 |
| cg21818252 | *SEC24B* | 10427 | 0.10 | 0.04368 |
| cg23829949 | *ZNF238* | 10472 | -0.26 | 0.04368 |
| cg26534508 | *GPR137B* | 7107 | 0.09 | 0.04368 |
| cg07688234 | *PFC* | 5199 | 0.32 | 0.04368 |
| cg21846488 | *LCE4A* | 199834 | 0.39 | 0.04368 |
| cg01184449 | *EML4* | 27436 | -0.35 | 0.04370 |
| cg00332153 | *BANK1* | 55024 | 0.09 | 0.04371 |
| cg01491225 | *ZCCHC9* | 84240 | 0.08 | 0.04371 |
| cg09628601 | *NPAS1* | 4861 | 0.14 | 0.04371 |
| cg11512771 | *ECM1* | 1893 | -0.10 | 0.04371 |
| cg12631737 | *FUT4* | 2526 | 0.10 | 0.04371 |
| cg13053608 | *LGP1* | 84514 | 0.47 | 0.04371 |
| cg15544721 | *PPP1R9A* | 55607 | -0.26 | 0.04371 |
| cg17430393 | *VSNL1* | 7447 | 0.07 | 0.04371 |
| cg18591973 | *SMARCD1* | 6602 | 0.10 | 0.04371 |
| cg24670715 | *ANGPT2* | 285 | -0.32 | 0.04371 |
| cg03479797 | *CREBL1* | 1388 | 0.09 | 0.04371 |
| cg12815916 | *PTPN18* | 26469 | -0.46 | 0.04371 |
| cg12864853 | *TPBG* | 7162 | 0.09 | 0.04371 |
| cg25572812 | *DCDC1* | 341019 | 0.10 | 0.04371 |
| cg23141855 | *TFPI2* | 7980 | 0.06 | 0.04372 |
| cg10741369 | *TRIM7* | 81786 | 0.15 | 0.04372 |
| cg09059548 | *PHC1* | 1911 | 0.10 | 0.04373 |
| cg10185638 | *ATG9A* | 79065 | 0.09 | 0.04373 |
| cg09204187 | *FLJ14346* | 80097 | -0.26 | 0.04373 |
| cg25990230 | *PMS2L3* | 5387 | 0.09 | 0.04373 |
| cg09923855 | *HTATSF1* | 27336 | 0.14 | 0.04374 |
| cg07960846 | *STK11* | 6794 | 0.08 | 0.04374 |
| cg24035370 | *PRKCZ* | 5590 | 0.14 | 0.04374 |
| cg04628008 | *ZBTB16* | 7704 | 0.12 | 0.04375 |
| cg14094063 | *BRAF* | 673 | 0.08 | 0.04375 |
| cg22982528 | *BRWD1* | 54014 | 0.11 | 0.04375 |
| cg25973092 | *SESN2* | 83667 | 0.09 | 0.04375 |
| cg17696091 | *SYCP1* | 6847 | -0.43 | 0.04375 |
| cg19211800 | *MARCKS* | 4082 | 0.09 | 0.04375 |
| cg24901474 | *RGS5* | 8490 | 0.19 | 0.04375 |
| cg01871963 | *LCN2* | 3934 | -0.07 | 0.04376 |
| cg03667047 | *KL* | 9365 | 0.13 | 0.04376 |
| cg05111110 | *LRFN4* | 78999 | -0.56 | 0.04376 |
| cg20089715 | *CACNB1* | 782 | -0.53 | 0.04376 |
| cg00619207 | *DENND2D* | 79961 | 0.20 | 0.04376 |
| cg00762512 | *LIAS* | 11019 | 0.16 | 0.04376 |
| cg02953306 | *CHRNA2* | 1135 | 0.17 | 0.04376 |
| cg04418771 | *COPS4* | 51138 | 0.09 | 0.04376 |
| cg06242827 | *WWP1* | 11059 | 0.09 | 0.04376 |
| cg06816063 | *RAB8A* | 4218 | 0.08 | 0.04376 |
| cg07341907 | *UNC13B* | 10497 | 0.10 | 0.04376 |
| cg07473550 | *TSC22D3* | 1831 | 0.08 | 0.04376 |
| cg07485777 | *TRIM60* | 166655 | -0.39 | 0.04376 |
| cg08179907 | *RAB39* | 54734 | 0.08 | 0.04376 |
| cg10786622 | *NUP153* | 9972 | 0.12 | 0.04376 |
| cg10878998 | *CYB5A* | 1528 | 0.12 | 0.04376 |
| cg12205591 | *CRYAA* | 1409 | 0.32 | 0.04376 |
| cg13022129 | *PHYHD1* | 254295 | -0.59 | 0.04376 |
| cg13813391 | *CMTM2* | 146225 | -0.34 | 0.04376 |
| cg14289461 | *LAMA4* | 3910 | -0.26 | 0.04376 |
| cg15590526 | *ZNF124* | 7678 | -0.35 | 0.04376 |
| cg16029760 | *OSTbeta* | 123264 | 0.10 | 0.04376 |
| cg17269277 | *FGFR3* | 2261 | 0.14 | 0.04376 |
| cg18934187 | *STARD6* | 147323 | 0.28 | 0.04376 |
| cg19951443 | *C9orf46* | 55848 | 0.07 | 0.04376 |
| cg20430101 | *RBM10* | 8241 | -0.22 | 0.04376 |
| cg21550442 | *FLJ36445* | 163183 | -0.37 | 0.04376 |
| cg21660687 | *GLI1* | 2735 | 0.09 | 0.04376 |
| cg22019980 | *RNF146* | 81847 | 0.10 | 0.04376 |
| cg24524435 | *UCKL1* | 54963 | 0.11 | 0.04376 |
| cg25650110 | *ZDHHC8* | 29801 | 0.12 | 0.04376 |
| cg27387222 | *DNM1L* | 10059 | -0.40 | 0.04376 |
| cg09305224 | *FUT7* | 2529 | 0.35 | 0.04379 |
| cg10493739 | *TMEM38B* | 55151 | 0.12 | 0.04379 |
| cg00195561 | *CHRM4* | 1132 | -0.07 | 0.04379 |
| cg18194038 | *CIRBP* | 1153 | 0.13 | 0.04380 |
| cg23786576 | *ATPAF1* | 64756 | 0.08 | 0.04383 |
| cg00351011 | *LASS4* | 79603 | 0.09 | 0.04385 |
| cg00568128 | *C10orf118* | 55088 | 0.09 | 0.04385 |
| cg10549973 | *UNQ9438* | 387990 | 0.09 | 0.04385 |
| cg21098323 | *OSCAR* | 126014 | -0.41 | 0.04385 |
| cg23771661 | *B3GNT3* | 10331 | -0.39 | 0.04385 |
| cg25812776 | *TRAF5* | 7188 | 0.09 | 0.04385 |
| cg27537561 | *VDR* | 7421 | 0.10 | 0.04385 |
| cg24235633 | *CDIPT* | 10423 | -0.52 | 0.04387 |
| cg00136736 | *C22orf19* | 8563 | 0.08 | 0.04389 |
| cg04096767 | *WT1* | 7490 | -0.46 | 0.04389 |
| cg08571203 | *FOXJ2* | 55810 | 0.06 | 0.04389 |
| cg23172864 | *GOPC* | 57120 | 0.08 | 0.04389 |
| cg23626798 | *L3MBTL* | 26013 | -0.34 | 0.04389 |
| cg01309671 | *RP3-473B4.1* | 159091 | 0.13 | 0.04392 |
| cg01566404 | *ETNK2* | 55224 | -0.29 | 0.04392 |
| cg03985657 | *C1orf2* | 10712 | 0.10 | 0.04392 |
| cg05445326 | *TM4SF19* | 116211 | -0.40 | 0.04392 |
| cg08034216 | *IDE* | 3416 | 0.11 | 0.04392 |
| cg24668150 | *ANKRD26* | 22852 | 0.10 | 0.04392 |
| cg26645834 | *SPO11* | 23626 | -0.43 | 0.04392 |
| cg01177956 | *SLC4A2* | 6522 | 0.12 | 0.04393 |
| cg04086012 | *FLJ36180* | 339976 | 0.41 | 0.04393 |
| cg07364841 | *HGFAC* | 3083 | -0.12 | 0.04393 |
| cg08972170 | *Ells1* | 222166 | 0.18 | 0.04393 |
| cg10795646 | *S100A10* | 6281 | 0.25 | 0.04393 |
| cg12187567 | *CCNE2* | 9134 | 0.10 | 0.04393 |
| cg13613682 | *ZIC3* | 7547 | 0.12 | 0.04393 |
| cg14179628 | *TCEAL7* | 56849 | -0.33 | 0.04393 |
| cg15452204 | *CDX1* | 1044 | -0.27 | 0.04393 |
| cg16415058 | *SORCS1* | 114815 | -0.45 | 0.04393 |
| cg21892708 | *PDIA6* | 10130 | -0.31 | 0.04393 |
| cg23653008 | *CHFR* | 55743 | 0.08 | 0.04393 |
| cg10485724 | *PMS2* | 5395 | 0.12 | 0.04394 |
| cg13160888 | *NIPSNAP3B* | 55335 | 0.07 | 0.04396 |
| cg07084709 | *Gcom1* | 145781 | -0.36 | 0.04397 |
| cg12697789 | *TLR3* | 7098 | 0.34 | 0.04397 |
| cg17657618 | *FLJ43806* | 399563 | 0.43 | 0.04397 |
| cg22421699 | *SMPDL3B* | 27293 | 0.40 | 0.04397 |
| cg23057732 | *C3orf39* | 84892 | 0.15 | 0.04397 |
| cg25593040 | *DDX18* | 8886 | 0.09 | 0.04397 |
| cg26033681 | *SNRPN* | 6638 | -0.29 | 0.04397 |
| cg20777437 | *CDCP2* | 200008 | 0.24 | 0.04398 |
| cg17738133 | *CDKN1C* | 1028 | 0.10 | 0.04399 |
| cg12219915 | *FAM50A* | 9130 | 0.09 | 0.04400 |
| cg13551243 | *ZNF7* | 7553 | 0.11 | 0.04400 |
| cg20592700 | *WIPI2* | 26100 | 0.24 | 0.04400 |
| cg04275881 | *SLAMF8* | 56833 | 0.41 | 0.04401 |
| cg24241455 | *KLHL20* | 27252 | 0.10 | 0.04402 |
| cg25195673 | *GFPT1* | 2673 | -0.25 | 0.04402 |
| cg10148841 | *ROBO4* | 54538 | 0.25 | 0.04403 |
| cg02397514 | *HES4* | 57801 | 0.09 | 0.04403 |
| cg16499669 | *PEPP-2* | 84528 | -0.40 | 0.04403 |
| cg24573310 | *C10orf70* | 55847 | 0.10 | 0.04403 |
| cg08723608 | *FLJ37538* | 222950 | -0.32 | 0.04404 |
| cg16152813 | *FAM57B* | 83723 | 0.29 | 0.04405 |
| cg02007844 | *KCNJ8* | 3764 | 0.10 | 0.04407 |
| cg03521600 | *MTBP* | 27085 | 0.08 | 0.04407 |
| cg09118625 | *DIRAS3* | 9077 | -0.36 | 0.04407 |
| cg11961618 | *PHLDA2* | 7262 | 0.13 | 0.04407 |
| cg14560064 | *ZNF482* | 10773 | 0.07 | 0.04407 |
| cg14780914 | *POLR3F* | 10621 | 0.07 | 0.04407 |
| cg17867194 | *SLC30A6* | 55676 | 0.11 | 0.04407 |
| cg18056600 | *ZMYND15* | 84225 | 0.08 | 0.04407 |
| cg20300246 | *LHX3* | 8022 | 0.12 | 0.04407 |
| cg01249910 | *SAT2* | 112483 | 0.13 | 0.04407 |
| cg09686635 | *C1orf107* | 27042 | 0.11 | 0.04407 |
| cg07942995 | *ZSCAN1* | 284312 | -0.38 | 0.04408 |
| cg09558502 | *OVGP1* | 5016 | 0.21 | 0.04408 |
| cg13564075 | *PHF12* | 57649 | 0.08 | 0.04408 |
| cg23322112 | *ABCC13* | 150000 | -0.25 | 0.04408 |
| cg00524288 | *UPP1* | 7378 | 0.10 | 0.04408 |
| cg04380669 | *NME1* | 4830 | 0.09 | 0.04408 |
| cg04633513 | *AVPR1B* | 553 | 0.11 | 0.04408 |
| cg15368822 | *TMEM63A* | 9725 | 0.06 | 0.04408 |
| cg15750102 | *MGC16169* | 93627 | 0.09 | 0.04408 |
| cg17606785 | *EFS* | 10278 | -0.33 | 0.04408 |
| cg21459867 | *IDUA* | 3425 | -0.33 | 0.04408 |
| cg22459359 | *ZMYM6* | 9204 | 0.08 | 0.04408 |
| cg24797830 | *COPS6* | 10980 | 0.13 | 0.04408 |
| cg25758314 | *ARD1A* | 8260 | -0.34 | 0.04408 |
| cg10035922 | *C1orf102* | 127700 | 0.11 | 0.04410 |
| cg23731764 | *EXOSC10* | 5394 | 0.10 | 0.04410 |
| cg22045288 | *C10orf91* | 170393 | 0.34 | 0.04410 |
| cg02261346 | *MPPED1* | 758 | 0.08 | 0.04411 |
| cg06675496 | *ITSN2* | 50618 | 0.11 | 0.04411 |
| cg17834443 | *C8orf35* | 55174 | 0.14 | 0.04411 |
| cg21380294 | *ZSCAN2* | 54993 | 0.15 | 0.04411 |
| cg23812886 | *SSX5* | 6758 | -0.32 | 0.04411 |
| cg25383093 | *PGM3* | 5238 | -0.29 | 0.04411 |
| cg05346140 | *HSPB9* | 94086 | -0.38 | 0.04411 |
| cg06382459 | *SKI* | 6497 | 0.17 | 0.04414 |
| cg11300809 | *SGPP2* | 130367 | -0.28 | 0.04414 |
| cg04564030 | *CDH7* | 1005 | 0.11 | 0.04415 |
| cg05485060 | *CTNNAL1* | 8727 | -0.39 | 0.04415 |
| cg09926889 | *RNF34* | 80196 | 0.10 | 0.04415 |
| cg02301754 | *TMOD4* | 29765 | -0.09 | 0.04415 |
| cg02827328 | *TRIM14* | 9830 | 0.11 | 0.04415 |
| cg08089301 | *HOXB4* | 3214 | 0.13 | 0.04415 |
| cg14058345 | *TXNDC6* | 347736 | 0.06 | 0.04415 |
| cg23047271 | *PRICKLE2* | 166336 | 0.33 | 0.04415 |
| cg24088438 | *URP2* | 83706 | 0.44 | 0.04415 |
| cg10158181 | *GRK4* | 2868 | 0.09 | 0.04416 |
| cg17084361 | *CDS1* | 1040 | 0.09 | 0.04416 |
| cg14061619 | *SH3BP4* | 23677 | 0.09 | 0.04416 |
| cg14816748 | *PRPSAP1* | 5635 | 0.11 | 0.04416 |
| cg15258980 | *ARHGAP25* | 9938 | 0.37 | 0.04416 |
| cg20615951 | *C1orf71* | 163882 | 0.07 | 0.04416 |
| cg22409223 | *PIK3R4* | 30849 | 0.12 | 0.04416 |
| cg12846567 | *ZNF556* | 80032 | -0.35 | 0.04417 |
| cg19586645 | *MIF4GD* | 57409 | 0.11 | 0.04417 |
| cg15568167 | *SAMD10* | 140700 | 0.08 | 0.04418 |
| cg07678643 | *LOC113444* | 113444 | 0.10 | 0.04419 |
| cg00185839 | *LRDD* | 55367 | 0.09 | 0.04419 |
| cg08525145 | *RLN3R2* | 339403 | 0.25 | 0.04419 |
| cg19339841 | *CENPE* | 1062 | 0.08 | 0.04419 |
| cg21046940 | *DAP* | 1611 | 0.12 | 0.04419 |
| cg21378206 | *IL1F5* | 26525 | 0.46 | 0.04419 |
| cg25191725 | *PYY2* | 23615 | -0.39 | 0.04419 |
| cg26484333 | *TMEM34* | 55751 | 0.09 | 0.04419 |
| cg05697976 | *MLSTD1* | 55711 | 0.33 | 0.04419 |
| cg25109024 | *DDX21* | 9188 | 0.11 | 0.04419 |
| cg19093820 | *GPR156* | 165829 | 0.27 | 0.04420 |
| cg21383720 | *C9orf10* | 23196 | 0.07 | 0.04420 |
| cg08623383 | *C1orf38* | 9473 | 0.29 | 0.04421 |
| cg00504595 | *TNFRSF19* | 55504 | 0.36 | 0.04421 |
| cg00935430 | *ZNF678* | 339500 | 0.08 | 0.04421 |
| cg01009664 | *TRH* | 7200 | 0.09 | 0.04421 |
| cg01044722 | *MYH10* | 4628 | 0.12 | 0.04421 |
| cg01443630 | *CLCN7* | 1186 | -0.47 | 0.04421 |
| cg01651593 | *CDC20* | 991 | 0.09 | 0.04421 |
| cg01808706 | *ITPKA* | 3706 | 0.07 | 0.04421 |
| cg01856970 | *IL15* | 3600 | 0.09 | 0.04421 |
| cg03264209 | *CREBBP* | 1387 | -0.40 | 0.04421 |
| cg03883348 | *KLC3* | 147700 | -0.48 | 0.04421 |
| cg04513422 | *LOC283487* | 283487 | 0.34 | 0.04421 |
| cg04826422 | *LOC129285* | 129285 | -0.39 | 0.04421 |
| cg05004940 | *C20orf195* | 79025 | 0.14 | 0.04421 |
| cg05307923 | *ADARB2* | 105 | 0.09 | 0.04421 |
| cg05822532 | *ELN* | 2006 | 0.55 | 0.04421 |
| cg05824432 | *DCI* | 1632 | 0.09 | 0.04421 |
| cg06334496 | *TMEM70* | 54968 | 0.10 | 0.04421 |
| cg06834261 | *WDR12* | 55759 | 0.10 | 0.04421 |
| cg07749074 | *HRG* | 3273 | 0.12 | 0.04421 |
| cg08074477 | *TNS4* | 84951 | 0.12 | 0.04421 |
| cg08123074 | *PHACTR4* | 65979 | 0.30 | 0.04421 |
| cg08147886 | *ACSL3* | 2181 | 0.11 | 0.04421 |
| cg08670691 | *CCDC27* | 148870 | 0.12 | 0.04421 |
| cg08722122 | *FGFR1* | 2260 | -0.43 | 0.04421 |
| cg09400966 | *PROS1* | 5627 | 0.09 | 0.04421 |
| cg09633588 | *C1orf174* | 339448 | 0.12 | 0.04421 |
| cg09869042 | *C22orf18* | 79019 | 0.10 | 0.04421 |
| cg10172318 | *DRAP1* | 10589 | 0.11 | 0.04421 |
| cg10642330 | *NNAT* | 4826 | -0.21 | 0.04421 |
| cg10689889 | *MAF* | 4094 | 0.08 | 0.04421 |
| cg11110686 | *MAGEC1* | 9947 | -0.56 | 0.04421 |
| cg11498156 | *TLX1* | 3195 | -0.33 | 0.04421 |
| cg11638200 | *PRKCQ* | 5588 | 0.13 | 0.04421 |
| cg11977634 | *DDR1* | 780 | 0.08 | 0.04421 |
| cg12240824 | *ZNF271* | 10778 | 0.10 | 0.04421 |
| cg13118748 | *C6orf136* | 221545 | 0.08 | 0.04421 |
| cg13144004 | *UMPS* | 7372 | 0.07 | 0.04421 |
| cg13398993 | *ARMC1* | 55156 | 0.10 | 0.04421 |
| cg13873733 | *MASA* | 58478 | 0.08 | 0.04421 |
| cg14914852 | *ZPBP2* | 124626 | -0.34 | 0.04421 |
| cg15166089 | *CD164L2* | 388611 | -0.33 | 0.04421 |
| cg16404689 | *C1orf156* | 92342 | 0.11 | 0.04421 |
| cg17561435 | *BMPER* | 168667 | 0.10 | 0.04421 |
| cg18604842 | *FLJ36046* | 164592 | -0.50 | 0.04421 |
| cg18652121 | *NDUFB5* | 4711 | 0.12 | 0.04421 |
| cg19238840 | *GP2* | 2813 | 0.16 | 0.04421 |
| cg19717326 | *MYADM* | 91663 | -0.38 | 0.04421 |
| cg21379816 | *SRM* | 6723 | 0.07 | 0.04421 |
| cg21517055 | *MGC11271* | 79173 | -0.30 | 0.04421 |
| cg21547976 | *STK11* | 6794 | 0.08 | 0.04421 |
| cg21991396 | *CIAS1* | 114548 | 0.41 | 0.04421 |
| cg23131950 | *AP2S1* | 1175 | -0.41 | 0.04421 |
| cg23213170 | *FCMD* | 2218 | 0.09 | 0.04421 |
| cg23878206 | *ATF5* | 22809 | -0.37 | 0.04421 |
| cg24137081 | *IQCC* | 55721 | 0.09 | 0.04421 |
| cg24692716 | *C6orf192* | 116843 | 0.10 | 0.04421 |
| cg24892074 | *PIGY* | 84992 | 0.08 | 0.04421 |
| cg25063710 | *BRWD3* | 254065 | 0.11 | 0.04421 |
| cg26944151 | *FAM69B* | 138311 | 0.11 | 0.04421 |
| cg27108154 | *PDHA2* | 5161 | -0.42 | 0.04421 |
| cg27239921 | *CACNA1D* | 776 | 0.08 | 0.04421 |
| cg18986332 | *CLASP2* | 23122 | 0.08 | 0.04422 |
| cg08063724 | *MYCL1* | 4610 | 0.10 | 0.04422 |
| cg13973816 | *SCAND2* | 54581 | 0.08 | 0.04422 |
| cg04845063 | *FAM13A1* | 10144 | 0.09 | 0.04423 |
| cg25219333 | *FXC1* | 26515 | -0.34 | 0.04423 |
| cg05306735 | *ZNF273* | 10793 | 0.09 | 0.04424 |
| cg13334277 | *SPACA1* | 81833 | -0.28 | 0.04426 |
| cg00448720 | *MGC48915* | 338872 | -0.42 | 0.04426 |
| cg01998785 | *AYTL1* | 54947 | -0.45 | 0.04426 |
| cg02361557 | *NTNG1* | 22854 | 0.10 | 0.04426 |
| cg05428978 | *CDCA7* | 83879 | 0.10 | 0.04426 |
| cg09234859 | *KLHL7* | 55975 | 0.10 | 0.04426 |
| cg14297029 | *SSTR3* | 6753 | -0.08 | 0.04426 |
| cg15494458 | *BPI* | 671 | 0.38 | 0.04426 |
| cg18508125 | *FBXO16* | 157574 | 0.11 | 0.04426 |
| cg22154562 | *HRBL* | 3268 | 0.11 | 0.04426 |
| cg22328890 | *SLC7A6* | 9057 | 0.11 | 0.04426 |
| cg24378421 | *MTMR9* | 66036 | -0.27 | 0.04426 |
| cg25505974 | *C21orf59* | 56683 | 0.10 | 0.04426 |
| cg01400401 | *C19orf30* | 284424 | 0.14 | 0.04427 |
| cg13434852 | *DOCK1* | 1793 | 0.45 | 0.04427 |
| cg25298754 | *ZBED2* | 79413 | 0.16 | 0.04427 |
| cg06772202 | *STYXL1* | 51657 | -0.32 | 0.04430 |
| cg01172899 | *SLC16A14* | 151473 | 0.10 | 0.04431 |
| cg14851685 | *FLJ39501* | 126410 | -0.62 | 0.04431 |
| cg16408565 | *ORM2* | 5005 | -0.08 | 0.04431 |
| cg25268718 | *PSME1* | 5720 | -0.32 | 0.04431 |
| cg24077317 | *UBE1C* | 9039 | 0.10 | 0.04431 |
| cg00436603 | *CYP2E1* | 1571 | -0.38 | 0.04432 |
| cg15215114 | *C17orf61* | 254863 | 0.08 | 0.04432 |
| cg26824467 | *LASS6* | 253782 | 0.07 | 0.04433 |
| cg10920957 | *JPH3* | 57338 | 0.10 | 0.04434 |
| cg01130192 | *ACO1* | 48 | 0.07 | 0.04435 |
| cg07387450 | *TMED5* | 50999 | 0.09 | 0.04435 |
| cg19973135 | *MARVELD3* | 91862 | 0.09 | 0.04435 |
| cg02988947 | *LIMD2* | 80774 | -0.28 | 0.04435 |
| cg05876918 | *PIP5K2C* | 79837 | 0.10 | 0.04435 |
| cg17084151 | *NCALD* | 83988 | 0.21 | 0.04435 |
| cg09083627 | *SLITRK5* | 26050 | 0.08 | 0.04435 |
| cg12620111 | *ZNF70* | 7621 | 0.09 | 0.04435 |
| cg13806670 | *MARCH8* | 220972 | 0.09 | 0.04435 |
| cg16360444 | *ITGA7* | 3679 | 0.14 | 0.04435 |
| cg17527798 | *LTF* | 4057 | 0.12 | 0.04435 |
| cg18011401 | *CACNA1A* | 773 | 0.15 | 0.04435 |
| cg20903926 | *C1orf177* | 163747 | -0.53 | 0.04435 |
| cg23839680 | *CCT6A* | 908 | 0.14 | 0.04435 |
| cg01958189 | *DEFB125* | 245938 | 0.35 | 0.04436 |
| cg07053469 | *EID-3* | 126272 | 0.08 | 0.04436 |
| cg04838107 | *ARF6* | 382 | 0.09 | 0.04437 |
| cg08529852 | *EGFL7* | 51162 | 0.32 | 0.04437 |
| cg09434995 | *SRCRB4D* | 136853 | 0.07 | 0.04437 |
| cg17997329 | *MGC11332* | 84804 | 0.09 | 0.04437 |
| cg25040783 | *APPL* | 26060 | 0.11 | 0.04437 |
| cg25268283 | *DMRTC1* | 63947 | -0.31 | 0.04437 |
| cg04527918 | *UCN* | 7349 | -0.52 | 0.04438 |
| cg05647859 | *LIN7A* | 8825 | 0.11 | 0.04438 |
| cg07485588 | *FOXD4L4* | 349334 | -0.46 | 0.04438 |
| cg11115427 | *PHYH* | 5264 | -0.21 | 0.04438 |
| cg11489580 | *G3BP2* | 9908 | 0.11 | 0.04438 |
| cg15185804 | *PGM3* | 5238 | 0.10 | 0.04438 |
| cg17260124 | *KCTD14* | 65987 | -0.23 | 0.04438 |
| cg18930892 | *OTEX* | 158800 | -0.40 | 0.04438 |
| cg19279346 | *LILRB2* | 10288 | 0.15 | 0.04438 |
| cg25361844 | *SIPA1* | 6494 | 0.11 | 0.04438 |
| cg26974738 | *CUTA* | 51596 | 0.09 | 0.04438 |
| cg05194726 | *NRIP2* | 83714 | 0.28 | 0.04438 |
| cg05674444 | *C21orf99* | 149992 | 0.31 | 0.04438 |
| cg11086066 | *NFATC2* | 4773 | 0.06 | 0.04438 |
| cg21185421 | *C9orf42* | 116224 | 0.08 | 0.04438 |
| cg21960882 | *TP53TG3* | 24150 | -0.51 | 0.04438 |
| cg23828212 | *MAN1A2* | 10905 | 0.10 | 0.04438 |
| cg00514895 | *CPZ* | 8532 | 0.11 | 0.04439 |
| cg11458974 | *HMOX1* | 3162 | -0.23 | 0.04439 |
| cg12484193 | *DUSP19* | 142679 | 0.08 | 0.04439 |
| cg14175593 | *MMS19L* | 64210 | 0.12 | 0.04439 |
| cg14623805 | *CCAR1* | 55749 | 0.09 | 0.04439 |
| cg15600342 | *WBP4* | 11193 | 0.07 | 0.04439 |
| cg19664945 | *GPR75* | 10936 | -0.40 | 0.04439 |
| cg09509673 | *CCR10* | 2826 | -0.40 | 0.04440 |
| cg15613982 | *SEC23B* | 10483 | 0.14 | 0.04440 |
| cg16769376 | *MDP-1* | 145553 | 0.10 | 0.04440 |
| cg17794261 | *ZNF688* | 146542 | 0.09 | 0.04440 |
| cg19516009 | *SFTPC* | 6440 | -0.09 | 0.04440 |
| cg22026853 | *POU3F2* | 5454 | 0.10 | 0.04440 |
| cg27033479 | *TMEM44* | 93109 | 0.13 | 0.04440 |
| cg27067621 | *NOX1* | 27035 | 0.22 | 0.04440 |
| cg13784855 | *PVT1* | 5820 | 0.08 | 0.04441 |
| cg21503093 | *EMP2* | 2013 | 0.09 | 0.04442 |
| cg26301689 | *PAQR9* | 344838 | -0.52 | 0.04442 |
| cg27363486 | *TRIM17* | 51127 | -0.42 | 0.04442 |
| cg23538854 | *IL7* | 3574 | 0.08 | 0.04442 |
| cg11553177 | *SDF2* | 6388 | 0.09 | 0.04442 |
| cg20225681 | *CILP* | 8483 | -0.05 | 0.04442 |
| cg01804429 | *YTHDC1* | 91746 | -0.25 | 0.04443 |
| cg07288587 | *CSNK1G1* | 53944 | 0.07 | 0.04443 |
| cg09664442 | *ERICH1* | 157697 | 0.09 | 0.04443 |
| cg13287780 | *WRB* | 7485 | -0.31 | 0.04443 |
| cg15156844 | *DLGAP1* | 9229 | -0.43 | 0.04443 |
| cg19691267 | *ACYP1* | 97 | 0.15 | 0.04443 |
| cg27631256 | *TNFSF8* | 944 | 0.12 | 0.04443 |
| cg03591594 | *VLDLR* | 7436 | 0.12 | 0.04447 |
| cg00108454 | *C1QA* | 712 | 0.20 | 0.04447 |
| cg07865790 | *IKIP* | 121457 | 0.09 | 0.04448 |
| cg11423990 | *TXN2* | 25828 | 0.10 | 0.04448 |
| cg00658626 | *CCDC63* | 160762 | 0.09 | 0.04450 |
| cg12575751 | *NUCB2* | 4925 | 0.10 | 0.04450 |
| cg05781767 | *PODXL2* | 50512 | -0.30 | 0.04451 |
| cg04720358 | *RCV1* | 5957 | -0.49 | 0.04451 |
| cg10402626 | *RPS5* | 6193 | 0.10 | 0.04451 |
| cg10432177 | *BIK* | 638 | 0.07 | 0.04451 |
| cg18177682 | *PEA15* | 8682 | 0.08 | 0.04451 |
| cg18380975 | *C6orf1* | 221491 | 0.12 | 0.04451 |
| cg25381844 | *SH3GL3* | 6457 | 0.09 | 0.04451 |
| cg25750259 | *C20orf121* | 79183 | 0.08 | 0.04451 |
| cg27625732 | *TBC1D13* | 54662 | -0.34 | 0.04451 |
| cg01025762 | *DLD* | 1738 | 0.15 | 0.04451 |
| cg03873930 | *ARFRP1* | 10139 | -0.29 | 0.04451 |
| cg24469114 | *RCC1* | 1104 | 0.11 | 0.04451 |
| cg03742214 | *CRY1* | 1407 | 0.11 | 0.04451 |
| cg08190044 | *ATP8B2* | 57198 | 0.10 | 0.04452 |
| cg22244418 | *OGFOD1* | 55239 | -0.36 | 0.04452 |
| cg18043455 | *SEC61A1* | 29927 | 0.09 | 0.04452 |
| cg18704047 | *PART1* | 25859 | -0.37 | 0.04452 |
| cg27465849 | *THEDC1* | 55301 | -0.17 | 0.04452 |
| cg18573383 | *KCNC2* | 3747 | 0.10 | 0.04453 |
| cg01145396 | *CHRNG* | 1146 | 0.40 | 0.04454 |
| cg01510051 | *ZNF542* | 147947 | -0.42 | 0.04454 |
| cg05722918 | *SLC5A8* | 160728 | 0.07 | 0.04454 |
| cg15760840 | *HOXA11* | 3207 | 0.10 | 0.04454 |
| cg16204289 | *FLJ13391* | 84141 | -0.05 | 0.04454 |
| cg17483510 | *GNB4* | 59345 | 0.09 | 0.04454 |
| cg22828602 | *RNF133* | 168433 | 0.17 | 0.04454 |
| cg25371038 | *TOP3B* | 8940 | 0.10 | 0.04454 |
| cg12441964 | *FLJ35894* | 283847 | -0.41 | 0.04454 |
| cg02025407 | *C20orf155* | 54675 | 0.09 | 0.04454 |
| cg06980460 | *FOLH1* | 2346 | -0.34 | 0.04454 |
| cg07755653 | *STX11* | 8676 | 0.09 | 0.04454 |
| cg07876586 | *PGRMC1* | 10857 | 0.11 | 0.04454 |
| cg09457245 | *ZNF385* | 25946 | 0.40 | 0.04454 |
| cg10106388 | *CD244* | 51744 | 0.39 | 0.04454 |
| cg10934032 | *RNASE6* | 6039 | 0.32 | 0.04454 |
| cg11715999 | *ATP5G1* | 516 | 0.07 | 0.04454 |
| cg12280407 | *KCTD12* | 115207 | 0.12 | 0.04454 |
| cg13153865 | *C8orf1* | 734 | 0.07 | 0.04454 |
| cg13340269 | *TMEM35* | 59353 | 0.11 | 0.04454 |
| cg13501117 | *WDR8* | 49856 | 0.07 | 0.04454 |
| cg14473123 | *UBE2V2* | 7336 | 0.08 | 0.04454 |
| cg16756998 | *ZDHHC8* | 29801 | 0.12 | 0.04454 |
| cg23639989 | *FAM105A* | 54491 | 0.09 | 0.04454 |
| cg06736444 | *SRRM2* | 23524 | -0.48 | 0.04455 |
| cg07550267 | *RPS8* | 6202 | 0.09 | 0.04455 |
| cg16379513 | *TSSK3* | 81629 | -0.49 | 0.04455 |
| cg26832154 | *DKFZp434N035* | 84222 | -0.26 | 0.04455 |
| cg12245996 | *PRKCQ* | 5588 | 0.09 | 0.04455 |
| cg04180868 | *PRTFDC1* | 56952 | 0.07 | 0.04456 |
| cg12578480 | *MSRB2* | 22921 | 0.10 | 0.04456 |
| cg20767356 | *HORMAD1* | 84072 | -0.35 | 0.04456 |
| cg10798171 | *ICA1* | 3382 | 0.11 | 0.04456 |
| cg01172735 | *SESN2* | 83667 | 0.07 | 0.04458 |
| cg10159529 | *IL5RA* | 3568 | -0.18 | 0.04458 |
| cg22995176 | *UPK3B* | 80761 | 0.38 | 0.04458 |
| cg25430696 | *TSR1* | 55720 | 0.10 | 0.04458 |
| cg03013422 | *AACS* | 65985 | 0.11 | 0.04459 |
| cg01390445 | *LIPH* | 200879 | -0.44 | 0.04459 |
| cg01852131 | *PPP1R10* | 5514 | 0.09 | 0.04459 |
| cg03560597 | *DUSP18* | 150290 | 0.08 | 0.04459 |
| cg04326040 | *VPS53* | 55275 | 0.10 | 0.04459 |
| cg06347928 | *IFIH1* | 64135 | 0.11 | 0.04459 |
| cg08190291 | *ADAMTS5* | 11096 | 0.12 | 0.04459 |
| cg10264187 | *SLC39A13* | 91252 | 0.10 | 0.04459 |
| cg11715966 | *CCDC8* | 83987 | -0.33 | 0.04459 |
| cg12783776 | *SERPING1* | 710 | 0.08 | 0.04459 |
| cg14186992 | *HKR3* | 3104 | -0.48 | 0.04459 |
| cg14315198 | *UNC119* | 9094 | -0.17 | 0.04459 |
| cg14358709 | *RNF19* | 25897 | 0.07 | 0.04459 |
| cg14611174 | *SIX6* | 4990 | 0.08 | 0.04459 |
| cg14736210 | *KCNB1* | 3745 | -0.43 | 0.04459 |
| cg14870461 | *AER61* | 285203 | 0.09 | 0.04459 |
| cg17881637 | *MRPL40* | 64976 | 0.11 | 0.04459 |
| cg18871670 | *ANAPC13* | 25847 | 0.12 | 0.04459 |
| cg19837131 | *PIK3C3* | 5289 | 0.09 | 0.04459 |
| cg24041977 | *CERK* | 64781 | 0.14 | 0.04459 |
| cg24777950 | *CTSG* | 1511 | 0.35 | 0.04459 |
| cg25088874 | *BMPR1B* | 658 | 0.09 | 0.04459 |
| cg25288155 | *PLEKHB1* | 58473 | -0.34 | 0.04459 |
| cg26623224 | *TRPM4* | 54795 | 0.13 | 0.04459 |
| cg26661257 | *HIF1A* | 3091 | 0.09 | 0.04459 |
| cg15565533 | *GMPPB* | 29925 | 0.11 | 0.04460 |
| cg12626411 | *PRG4* | 10216 | 0.15 | 0.04460 |
| cg01994328 | *MAP3K8* | 1326 | 0.08 | 0.04461 |
| cg02194878 | *EPHA8* | 2046 | 0.07 | 0.04461 |
| cg03399971 | *INHBC* | 3626 | -0.09 | 0.04461 |
| cg06363801 | *MOSPD1* | 56180 | 0.13 | 0.04461 |
| cg06456512 | *ANP32A* | 8125 | 0.09 | 0.04461 |
| cg07177852 | *CCDC68* | 80323 | 0.10 | 0.04461 |
| cg13588645 | *R3HDM1* | 23518 | 0.07 | 0.04461 |
| cg15396310 | *BCL7B* | 9275 | 0.11 | 0.04461 |
| cg17918239 | *ZNF281* | 23528 | 0.19 | 0.04461 |
| cg26267561 | *OXT* | 5020 | -0.32 | 0.04461 |
| cg03521347 | *NUDCD3* | 23386 | 0.11 | 0.04462 |
| cg06620254 | *NONO* | 4841 | 0.10 | 0.04462 |
| cg14580737 | *RFXANK* | 8625 | -0.26 | 0.04462 |
| cg15553522 | *HSPA5BP1* | 54972 | 0.13 | 0.04462 |
| cg16008138 | *RNF190* | 162333 | -0.31 | 0.04462 |
| cg18119407 | *CFLAR* | 8837 | -0.34 | 0.04462 |
| cg23032316 | *AAAS* | 8086 | 0.13 | 0.04462 |
| cg06130787 | *KLK10* | 5655 | 0.35 | 0.04463 |
| cg16778903 | *BRUNOL6* | 60677 | 0.06 | 0.04463 |
| cg02742971 | *OSR1* | 130497 | 0.07 | 0.04466 |
| cg22879515 | *BTG4* | 54766 | 0.10 | 0.04466 |
| cg07980518 | *RNF152* | 220441 | 0.09 | 0.04466 |
| cg00121640 | *ASTN2* | 23245 | 0.11 | 0.04467 |
| cg01376672 | *ANAPC5* | 51433 | 0.08 | 0.04469 |
| cg01817029 | *TRHDE* | 29953 | 0.10 | 0.04469 |
| cg04382920 | *SLC25A31* | 83447 | -0.50 | 0.04469 |
| cg04868764 | *SPINT1* | 6692 | 0.10 | 0.04469 |
| cg09001777 | *FUT3* | 2525 | 0.39 | 0.04469 |
| cg11265941 | *SNRPN* | 6638 | -0.35 | 0.04469 |
| cg21201659 | *SEC23IP* | 11196 | 0.08 | 0.04469 |
| cg24409107 | *GALE* | 2582 | 0.08 | 0.04469 |
| cg24879335 | *TF* | 7018 | 0.10 | 0.04469 |
| cg13041032 | *MMP13* | 4322 | -0.30 | 0.04469 |
| cg13562542 | *GPR27* | 2850 | 0.07 | 0.04469 |
| cg19264571 | *APCDD1* | 147495 | -0.49 | 0.04469 |
| cg03642357 | *ADRBK2* | 157 | 0.08 | 0.04471 |
| cg21022435 | *NFRKB* | 4798 | -0.44 | 0.04471 |
| cg25384157 | *ARRDC1* | 92714 | -0.20 | 0.04471 |
| cg06822229 | *ACSL4* | 2182 | 0.12 | 0.04472 |
| cg05856931 | *MSI2* | 124540 | 0.16 | 0.04472 |
| cg17651821 | *HIST1H4L* | 8368 | 0.11 | 0.04472 |
| cg03616357 | *FLJ21159* | 79884 | 0.07 | 0.04474 |
| cg00520135 | *TPM1* | 7168 | -0.45 | 0.04475 |
| cg04351979 | *CTXN1* | 404217 | 0.10 | 0.04475 |
| cg12353516 | *MAPK7* | 5598 | 0.08 | 0.04475 |
| cg12833011 | *KCNIP1* | 30820 | -0.31 | 0.04475 |
| cg25098095 | *C6orf55* | 51534 | 0.12 | 0.04475 |
| cg26336277 | *FYTTD1* | 84248 | 0.11 | 0.04475 |
| cg23244913 | *HCG9* | 10255 | 0.12 | 0.04475 |
| cg06622725 | *PEX16* | 9409 | 0.08 | 0.04478 |
| cg07561400 | *ZC3HAV1* | 56829 | 0.09 | 0.04478 |
| cg07881407 | *CCL14* | 6358 | 0.29 | 0.04478 |
| cg10179196 | *CXCL14* | 9547 | -0.17 | 0.04478 |
| cg15059932 | *ZNF236* | 7776 | 0.12 | 0.04478 |
| cg23191772 | *IDH3B* | 3420 | 0.09 | 0.04478 |
| cg02198582 | *TSC2* | 7249 | -0.08 | 0.04480 |
| cg04856685 | *MAP4* | 4134 | 0.11 | 0.04480 |
| cg07516225 | *C8orf41* | 80185 | 0.11 | 0.04480 |
| cg07739803 | *SLC30A7* | 148867 | 0.08 | 0.04480 |
| cg08823182 | *S100A5* | 6276 | 0.26 | 0.04480 |
| cg15465321 | *PEPP-2* | 84528 | -0.35 | 0.04480 |
| cg15516226 | *BTNL9* | 153579 | -0.08 | 0.04480 |
| cg19133903 | *AVPI1* | 60370 | 0.09 | 0.04480 |
| cg21553596 | *FAM3B* | 54097 | 0.12 | 0.04480 |
| cg26240353 | *LTA4H* | 4048 | 0.09 | 0.04480 |
| cg26353877 | *APCS* | 325 | 0.52 | 0.04480 |
| cg27547703 | *TDRD1* | 56165 | -0.28 | 0.04480 |
| cg08635249 | *RBM28* | 55131 | 0.10 | 0.04481 |
| cg15774495 | *GRB10* | 2887 | 0.09 | 0.04481 |
| cg18003231 | *SLC25A18* | 83733 | -0.09 | 0.04481 |
| cg18552413 | *DARC* | 2532 | 0.28 | 0.04481 |
| cg20856436 | *RPS27A* | 6233 | 0.09 | 0.04481 |
| cg23889010 | *SLPI* | 6590 | 0.34 | 0.04481 |
| cg10468702 | *PTGER1* | 5731 | -0.38 | 0.04482 |
| cg07818646 | *FLJ11712* | 79621 | 0.11 | 0.04482 |
| cg10910525 | *HRC* | 3270 | 0.12 | 0.04482 |
| cg25948993 | *UBXD6* | 7993 | 0.07 | 0.04483 |
| cg00238052 | *TCEAL2* | 140597 | 0.11 | 0.04484 |
| cg00307685 | *BRDT* | 676 | -0.35 | 0.04484 |
| cg00603172 | *BOK* | 666 | -0.42 | 0.04484 |
| cg00711916 | *CNNM4* | 26504 | 0.09 | 0.04484 |
| cg01138020 | *MGC29671* | 201305 | 0.42 | 0.04484 |
| cg03550002 | *ARSK* | 153642 | 0.10 | 0.04484 |
| cg03691812 | *CORO1A* | 11151 | 0.11 | 0.04484 |
| cg04551655 | *FLJ13646* | 79635 | -0.14 | 0.04484 |
| cg04733302 | *TTC18* | 118491 | 0.11 | 0.04484 |
| cg05011232 | *ETV4* | 2118 | 0.06 | 0.04484 |
| cg05689121 | *TNRC6A* | 27327 | 0.10 | 0.04484 |
| cg05782292 | *SCT* | 6343 | -0.35 | 0.04484 |
| cg06792911 | *SLC2A1* | 6513 | 0.10 | 0.04484 |
| cg08093211 | *CSTF2* | 1478 | 0.10 | 0.04484 |
| cg08558873 | *ZNF214* | 7761 | 0.06 | 0.04484 |
| cg10193817 | *IGSF4* | 23705 | 0.12 | 0.04484 |
| cg12558519 | *KLHL26* | 55295 | 0.09 | 0.04484 |
| cg14445437 | *PIGS* | 94005 | 0.09 | 0.04484 |
| cg14643978 | *TMC1* | 117531 | -0.52 | 0.04484 |
| cg14645972 | *DKFZp686I15217* | 401232 | 0.14 | 0.04484 |
| cg14797438 | *PPP4R1* | 9989 | 0.07 | 0.04484 |
| cg14800883 | *ARPC4* | 10093 | 0.16 | 0.04484 |
| cg15613048 | *KIF17* | 57576 | 0.09 | 0.04484 |
| cg16312916 | *BRAP* | 8315 | 0.08 | 0.04484 |
| cg17051440 | *CLDN2* | 9075 | 0.31 | 0.04484 |
| cg18469326 | *C17orf41* | 79915 | 0.09 | 0.04484 |
| cg19029220 | *UGP2* | 7360 | 0.09 | 0.04484 |
| cg19108718 | *LOC317671* | 317671 | 0.10 | 0.04484 |
| cg19839588 | *TRERF1* | 55809 | 0.07 | 0.04484 |
| cg19972619 | *MYC* | 4609 | 0.10 | 0.04484 |
| cg20103201 | *PANK4* | 55229 | 0.17 | 0.04484 |
| cg20313722 | *FKRP* | 79147 | -0.44 | 0.04484 |
| cg21085625 | *DBI* | 1622 | 0.08 | 0.04484 |
| cg21717724 | *PSMD5* | 5711 | -0.31 | 0.04484 |
| cg22782492 | *VHL* | 7428 | 0.10 | 0.04484 |
| cg23232084 | *UBL4A* | 8266 | -0.27 | 0.04484 |
| cg23707493 | *TNKS2* | 80351 | 0.10 | 0.04484 |
| cg24623694 | *PRX* | 57716 | 0.43 | 0.04484 |
| cg24971490 | *C8orf53* | 84294 | 0.08 | 0.04484 |
| cg25193278 | *BTN3A3* | 10384 | 0.34 | 0.04484 |
| cg26218269 | *MAB21L2* | 10586 | -0.32 | 0.04484 |
| cg26750319 | *KCNQ1* | 3784 | -0.07 | 0.04484 |
| cg16506346 | *OR1E1* | 8387 | -0.10 | 0.04486 |
| cg02005336 | *FEN1* | 2237 | 0.08 | 0.04486 |
| cg05564266 | *C6orf204* | 387119 | -0.30 | 0.04486 |
| cg10885338 | *ECRG4* | 84417 | -0.35 | 0.04486 |
| cg19937039 | *SERPINA10* | 51156 | 0.42 | 0.04486 |
| cg22105582 | *RAB27B* | 5874 | 0.07 | 0.04486 |
| cg22874960 | *LKAP* | 9665 | 0.09 | 0.04486 |
| cg04126816 | *HSU79303* | 29903 | 0.08 | 0.04487 |
| cg16312163 | *ALDH1L1* | 10840 | 0.09 | 0.04487 |
| cg24946133 | *JUN* | 3725 | 0.08 | 0.04487 |
| cg03506684 | *GYG2* | 8908 | 0.10 | 0.04488 |
| cg03693601 | *SHCBP1* | 79801 | 0.08 | 0.04488 |
| cg19027571 | *CHFR* | 55743 | 0.10 | 0.04488 |
| cg20141916 | *PLEK2* | 26499 | 0.07 | 0.04488 |
| cg26746469 | *KIAA0406* | 9675 | -0.35 | 0.04488 |
| cg00350702 | *ERGIC3* | 51614 | 0.08 | 0.04488 |
| cg02794695 | *SLA* | 6503 | 0.34 | 0.04488 |
| cg04765929 | *TNFAIP8L1* | 126282 | 0.11 | 0.04488 |
| cg07957491 | *UCK1* | 83549 | -0.24 | 0.04488 |
| cg08035942 | *ZADH2* | 284273 | 0.09 | 0.04488 |
| cg10098541 | *PDIA5* | 10954 | -0.32 | 0.04488 |
| cg10349665 | *NSDHL* | 50814 | -0.35 | 0.04488 |
| cg11120551 | *CHD1L* | 9557 | -0.43 | 0.04488 |
| cg13565157 | *PDE1B* | 5153 | -0.20 | 0.04488 |
| cg17009433 | *GLDC* | 2731 | 0.10 | 0.04488 |
| cg17720231 | *IGSF9* | 57549 | 0.12 | 0.04488 |
| cg19177941 | *ALDH1A3* | 220 | -0.41 | 0.04488 |
| cg24352736 | *YIPF2* | 78992 | 0.09 | 0.04488 |
| cg25811820 | *POFUT1* | 23509 | 0.13 | 0.04488 |
| cg27177839 | *JAG2* | 3714 | 0.08 | 0.04488 |
| cg27449489 | *FLJ35767* | 400629 | 0.26 | 0.04488 |
| cg11879514 | *SLC16A6* | 9120 | 0.12 | 0.04489 |
| cg00858899 | *CCND1* | 595 | 0.16 | 0.04489 |
| cg08448751 | *SEMA3G* | 56920 | -0.10 | 0.04489 |
| cg17067942 | *C5orf14* | 79770 | -0.33 | 0.04489 |
| cg22238923 | *DOK1* | 1796 | 0.09 | 0.04489 |
| cg07668036 | *PSMD4* | 5710 | 0.07 | 0.04490 |
| cg11261264 | *PTPRH* | 5794 | -0.09 | 0.04490 |
| cg24211742 | *NOC4L* | 79050 | 0.11 | 0.04490 |
| cg09003876 | *C18orf21* | 83608 | 0.08 | 0.04490 |
| cg12880658 | *CDO1* | 1036 | 0.11 | 0.04492 |
| cg00230368 | *IGF2R* | 3482 | 0.09 | 0.04493 |
| cg08395365 | *MCCC1* | 56922 | -0.42 | 0.04493 |
| cg11582100 | *GAD1* | 2571 | 0.16 | 0.04493 |
| cg16557944 | *GPX7* | 2882 | 0.10 | 0.04493 |
| cg18485955 | *TNFRSF17* | 608 | -0.10 | 0.04493 |
| cg19020694 | *C12orf24* | 29902 | 0.10 | 0.04493 |
| cg20639263 | *NP* | 4860 | 0.13 | 0.04493 |
| cg08097755 | *VGF* | 7425 | 0.12 | 0.04493 |
| cg10527457 | *ECT2* | 1894 | 0.09 | 0.04493 |
| cg18886444 | *USP4* | 7375 | 0.09 | 0.04493 |
| cg23165541 | *DAPK2* | 23604 | -0.36 | 0.04493 |
| cg13518327 | *THRB* | 7068 | 0.10 | 0.04495 |
| cg23830037 | *DRG2* | 1819 | 0.10 | 0.04495 |
| cg19853494 | *SELL* | 6402 | 0.28 | 0.04495 |
| cg13530039 | *CHRM1* | 1128 | 0.37 | 0.04495 |
| cg14473145 | *CLEC14A* | 161198 | 0.10 | 0.04495 |
| cg15372098 | *C3orf26* | 84319 | 0.09 | 0.04495 |
| cg18783781 | *MGC4399* | 84275 | -0.38 | 0.04495 |
| cg20714328 | *THBS4* | 7060 | -0.31 | 0.04495 |
| cg26267038 | *CNKSR2* | 22866 | 0.10 | 0.04495 |
| cg20063650 | *ZNF645* | 158506 | -0.40 | 0.04495 |
| cg01344518 | *RGS11* | 8786 | -0.31 | 0.04496 |
| cg09588210 | *FLJ39739* | 388685 | 0.22 | 0.04496 |
| cg10127415 | *MAGEB6* | 158809 | -0.38 | 0.04496 |
| cg10245048 | *TNFRSF10A* | 8797 | 0.08 | 0.04496 |
| cg11298616 | *CKAP2* | 26586 | 0.11 | 0.04496 |
| cg17832162 | *SEC11L3* | 90701 | 0.08 | 0.04496 |
| cg21975377 | *FZD6* | 8323 | 0.11 | 0.04496 |
| cg05532892 | *PSMA1* | 5682 | 0.08 | 0.04496 |
| cg07688613 | *C1orf60* | 65123 | 0.07 | 0.04496 |
| cg09196959 | *TRIM40* | 135644 | 0.32 | 0.04496 |
| cg09377899 | *DDX11* | 1663 | 0.07 | 0.04496 |
| cg15585987 | *SNTG1* | 54212 | -0.22 | 0.04496 |
| cg17029151 | *CHMP4C* | 92421 | 0.08 | 0.04496 |
| cg22313024 | *UQCRB* | 7381 | -0.40 | 0.04496 |
| cg25004981 | *LASS3* | 204219 | -0.43 | 0.04496 |
| cg08124399 | *DDX43* | 55510 | -0.37 | 0.04497 |
| cg18557145 | *CD72* | 971 | -0.19 | 0.04497 |
| cg18091964 | *CXorf9* | 54440 | 0.28 | 0.04497 |
| cg01531431 | *C20orf149* | 79144 | -0.40 | 0.04497 |
| cg25467043 | *THAP6* | 152815 | 0.08 | 0.04498 |
| cg05471521 | *STK6* | 6790 | -0.24 | 0.04499 |
| cg14900471 | *GATA4* | 2626 | 0.08 | 0.04500 |
| cg05819268 | *CACNG1* | 786 | -0.06 | 0.04500 |
| cg17272843 | *KCTD14* | 65987 | -0.15 | 0.04502 |
| cg00269932 | *LAIR2* | 3904 | 0.19 | 0.04504 |
| cg01155039 | *AMN* | 81693 | -0.39 | 0.04505 |
| cg02901679 | *IL10* | 3586 | 0.26 | 0.04505 |
| cg12467090 | *PIK3C2B* | 5287 | 0.37 | 0.04505 |
| cg23617760 | *C16orf44* | 79786 | -0.32 | 0.04505 |
| cg06144905 | *PIPOX* | 51268 | 0.28 | 0.04506 |
| cg10871523 | *FLJ30655* | 132320 | 0.09 | 0.04506 |
| cg13174077 | *CUL4B* | 8450 | -0.38 | 0.04507 |
| cg26089280 | *ARMC9* | 80210 | 0.08 | 0.04507 |
| cg09657265 | *SMPDL3A* | 10924 | 0.08 | 0.04507 |
| cg11291200 | *MAGEB2* | 4113 | -0.45 | 0.04507 |
| cg25682559 | *FHOD3* | 80206 | 0.10 | 0.04507 |
| cg14100184 | *GNG13* | 51764 | 0.30 | 0.04508 |
| cg01402255 | *GATAD2B* | 57459 | 0.38 | 0.04508 |
| cg01497527 | *ANKDD1A* | 348094 | -0.08 | 0.04509 |
| cg10498963 | *GALK1* | 2584 | 0.09 | 0.04509 |
| cg11750883 | *C1orf42* | 54544 | -0.35 | 0.04509 |
| cg17232861 | *TRIM10* | 10107 | -0.09 | 0.04509 |
| cg18928687 | *DCUN1D5* | 84259 | 0.07 | 0.04509 |
| cg18953280 | *TBC1D19* | 55296 | 0.11 | 0.04509 |
| cg19515446 | *HIST1H1T* | 3010 | -0.34 | 0.04509 |
| cg19570574 | *THBS1* | 7057 | 0.13 | 0.04509 |
| cg21685427 | *SGK2* | 10110 | 0.28 | 0.04509 |
| cg25372103 | *DLL1* | 28514 | 0.11 | 0.04509 |
| cg00178790 | *C18orf19* | 125228 | 0.08 | 0.04510 |
| cg16467694 | *POLR2G* | 5436 | 0.09 | 0.04511 |
| cg17176395 | *RAB2* | 5862 | 0.11 | 0.04512 |
| cg20643362 | *C19orf12* | 83636 | 0.13 | 0.04512 |
| cg26096837 | *FGF19* | 9965 | 0.14 | 0.04512 |
| cg04936930 | *PTS* | 5805 | 0.08 | 0.04512 |
| cg16040953 | *MGC10911* | 84262 | 0.12 | 0.04512 |
| cg27301343 | *EML2* | 24139 | 0.13 | 0.04512 |
| cg10516359 | *SLC35C1* | 55343 | -0.41 | 0.04514 |
| cg13906416 | *PVR* | 5817 | 0.09 | 0.04514 |
| cg15996947 | *L2HGDH* | 79944 | -0.36 | 0.04514 |
| cg16948979 | *WDR21A* | 26094 | 0.10 | 0.04514 |
| cg04095468 | *SECISBP2* | 79048 | 0.15 | 0.04514 |
| cg13589108 | *FAM5B* | 57795 | 0.10 | 0.04514 |
| cg01525376 | *LCK* | 3932 | 0.34 | 0.04515 |
| cg01600782 | *CALM2* | 805 | 0.10 | 0.04515 |
| cg08768421 | *GDA* | 9615 | 0.07 | 0.04516 |
| cg04180460 | *CTNNB1* | 1499 | 0.11 | 0.04519 |
| cg20252016 | *CDCA5* | 113130 | 0.09 | 0.04519 |
| cg26542130 | *PCBP1* | 5093 | 0.11 | 0.04519 |
| cg11648289 | *EN2* | 2020 | 0.10 | 0.04519 |
| cg01490535 | *FLJ10661* | 55199 | 0.20 | 0.04521 |
| cg25583174 | *FGF2* | 2247 | 0.16 | 0.04521 |
| cg00669623 | *CDC2L2* | 985 | 0.08 | 0.04521 |
| cg02268424 | *ZNF136* | 7695 | 0.09 | 0.04521 |
| cg02309594 | *HCRTR1* | 3061 | -0.20 | 0.04521 |
| cg05475277 | *FLJ23322* | 80020 | -0.53 | 0.04521 |
| cg07294870 | *PIGT* | 51604 | 0.10 | 0.04521 |
| cg11768416 | *C12orf12* | 196477 | -0.59 | 0.04521 |
| cg13314167 | *C9orf84* | 158401 | -0.26 | 0.04521 |
| cg20178764 | *GFRA3* | 2676 | -0.39 | 0.04521 |
| cg22199779 | *SMU1* | 55234 | 0.08 | 0.04521 |
| cg23152667 | *TBCA* | 6902 | 0.26 | 0.04521 |
| cg25764570 | *HLA-DRA* | 3122 | -0.13 | 0.04521 |
| cg26065952 | *MRPL21* | 219927 | 0.13 | 0.04521 |
| cg00397545 | *APOC1* | 341 | -0.20 | 0.04522 |
| cg08965337 | *RAP2C* | 57826 | 0.14 | 0.04522 |
| cg21218093 | *ZNF335* | 63925 | 0.10 | 0.04522 |
| cg11880211 | *CSH1* | 1442 | -0.25 | 0.04526 |
| cg14452392 | *CCNH* | 902 | 0.10 | 0.04526 |
| cg23074453 | *FRMD5* | 84978 | 0.10 | 0.04526 |
| cg13587740 | *HEXA* | 3073 | 0.07 | 0.04526 |
| cg07569756 | *LRRC59* | 55379 | 0.09 | 0.04527 |
| cg08397758 | *C10orf33* | 84795 | 0.36 | 0.04527 |
| cg01404197 | *ZW10* | 9183 | 0.12 | 0.04527 |
| cg03222066 | *ABCG4* | 64137 | -0.21 | 0.04527 |
| cg08747889 | *PTK7* | 5754 | 0.08 | 0.04527 |
| cg10919329 | *SLC35A1* | 10559 | 0.09 | 0.04527 |
| cg14439353 | *EEF1E1* | 9521 | 0.07 | 0.04527 |
| cg16124821 | *ZNF706* | 51123 | 0.08 | 0.04527 |
| cg18885346 | *PKHD1* | 5314 | 0.21 | 0.04527 |
| cg21785260 | *C2orf24* | 27013 | 0.20 | 0.04527 |
| cg26025944 | *SCNN1G* | 6340 | 0.11 | 0.04527 |
| cg00514407 | *SERPINE2* | 5270 | 0.11 | 0.04527 |
| cg03054162 | *PCYOX1* | 51449 | 0.11 | 0.04528 |
| cg05782445 | *KCNK3* | 3777 | -0.22 | 0.04528 |
| cg06688848 | *RSPRY1* | 89970 | 0.07 | 0.04528 |
| cg10829134 | *ZA20D3* | 54469 | 0.10 | 0.04528 |
| cg22630748 | *INHBE* | 83729 | 0.29 | 0.04528 |
| cg15836394 | *FDFT1* | 2222 | 0.13 | 0.04529 |
| cg16097772 | *LYZ* | 4069 | 0.32 | 0.04529 |
| cg20831708 | *SEC31L2* | 25956 | -0.36 | 0.04529 |
| cg21914262 | *MTIF3* | 219402 | 0.07 | 0.04529 |
| cg23797100 | *LAT* | 27040 | 0.42 | 0.04529 |
| cg03029616 | *KRTAP6-1* | 337966 | 0.37 | 0.04530 |
| cg04876451 | *PET112L* | 5188 | 0.08 | 0.04530 |
| cg09229231 | *DHRS3* | 9249 | 0.11 | 0.04530 |
| cg13519373 | *IL8RA* | 3577 | 0.12 | 0.04531 |
| cg16964535 | *DNAJC5G* | 285126 | -0.44 | 0.04531 |
| cg24743283 | *GNA12* | 2768 | 0.12 | 0.04532 |
| cg15761233 | *FLJ22555* | 79568 | -0.43 | 0.04533 |
| cg24485466 | *KIF14* | 9928 | 0.09 | 0.04533 |
| cg17680902 | *SKIV2L* | 6499 | 0.11 | 0.04533 |
| cg22719586 | *NDOR1* | 27158 | 0.06 | 0.04534 |
| cg13015534 | *ST6GALNAC1* | 55808 | 0.17 | 0.04535 |
| cg05010623 | *ACVR1C* | 130399 | 0.10 | 0.04535 |
| cg07080358 | *C2orf32* | 25927 | 0.10 | 0.04535 |
| cg09966445 | *AMPH* | 273 | -0.35 | 0.04535 |
| cg12312863 | *AMDHD2* | 51005 | -0.34 | 0.04535 |
| cg24922045 | *GAS2L2* | 246176 | 0.37 | 0.04535 |
| cg27027803 | *GNAS* | 2778 | -0.42 | 0.04535 |
| cg27205791 | *COPZ1* | 22818 | 0.09 | 0.04535 |
| cg17237063 | *RBMS3* | 27303 | 0.17 | 0.04535 |
| cg09446127 | *UBADC1* | 10422 | 0.11 | 0.04536 |
| cg10313633 | *TP53I11* | 9537 | -0.44 | 0.04536 |
| cg14450506 | *DDX56* | 54606 | -0.48 | 0.04536 |
| cg08865050 | *DDX12* | 440081 | 0.08 | 0.04538 |
| cg22165175 | *KCNA2* | 3737 | 0.10 | 0.04538 |
| cg06178072 | *HSPC176* | 51693 | -0.20 | 0.04538 |
| cg22463915 | *EEF1A2* | 1917 | -0.32 | 0.04538 |
| cg02583151 | *SIX1* | 6495 | 0.12 | 0.04540 |
| cg03401114 | *BMP8B* | 656 | -0.33 | 0.04540 |
| cg13892015 | *PPIL5* | 122769 | 0.10 | 0.04540 |
| cg17386700 | *MVD* | 4597 | 0.07 | 0.04542 |
| cg07054095 | *ZNF549* | 256051 | 0.06 | 0.04543 |
| cg08840010 | *TNFRSF9* | 3604 | 0.26 | 0.04544 |
| cg09872233 | *ALOX15* | 246 | 0.08 | 0.04544 |
| cg00725777 | *NGFRAP1* | 27018 | -0.35 | 0.04544 |
| cg00479269 | *C2orf29* | 55571 | 0.08 | 0.04544 |
| cg04269351 | *VTI1B* | 10490 | -0.40 | 0.04544 |
| cg08103676 | *MARCH6* | 10299 | 0.08 | 0.04544 |
| cg12066181 | *DNMT3A* | 1788 | 0.10 | 0.04544 |
| cg12554573 | *PARP3* | 10039 | -0.29 | 0.04544 |
| cg16834431 | *ATPIF1* | 93974 | 0.11 | 0.04544 |
| cg17303299 | *ENO2* | 2026 | 0.10 | 0.04544 |
| cg17920197 | *LGI4* | 163175 | -0.08 | 0.04544 |
| cg23282949 | *RENBP* | 5973 | 0.48 | 0.04544 |
| cg02387679 | *IQGAP2* | 10788 | 0.10 | 0.04545 |
| cg22688260 | *ZCSL2* | 285381 | 0.06 | 0.04545 |
| cg03793131 | *ELOVL1* | 64834 | 0.09 | 0.04546 |
| cg06856528 | *TMEFF2* | 23671 | 0.07 | 0.04546 |
| cg08805338 | *PPP3CB* | 5532 | 0.10 | 0.04546 |
| cg09089053 | *FLJ13265* | 79935 | -0.15 | 0.04546 |
| cg10037005 | *CD37* | 951 | 0.31 | 0.04546 |
| cg14132995 | *SLC35A2* | 7355 | 0.08 | 0.04546 |
| cg21969640 | *GPR84* | 53831 | 0.44 | 0.04546 |
| cg22979433 | *CKMT2* | 1160 | -0.32 | 0.04546 |
| cg08825571 | *MAF1* | 84232 | 0.12 | 0.04546 |
| cg08778586 | *ZCCHC17* | 51538 | 0.08 | 0.04546 |
| cg16249711 | *TBPL2* | 387332 | -0.44 | 0.04546 |
| cg22059008 | *TESSP1* | 360226 | -0.38 | 0.04546 |
| cg22108175 | *LPL* | 4023 | 0.12 | 0.04546 |
| cg22264946 | *C6orf84* | 22832 | 0.10 | 0.04546 |
| cg22986999 | *MRGPRF* | 219928 | 0.20 | 0.04546 |
| cg00449941 | *SPAG5* | 10615 | 0.12 | 0.04547 |
| cg02173484 | *GJA5* | 2702 | 0.33 | 0.04547 |
| cg04042106 | *MUS81* | 80198 | 0.09 | 0.04547 |
| cg24030630 | *TCIRG1* | 10312 | 0.08 | 0.04547 |
| cg10823926 | *ANKRD35* | 148741 | 0.11 | 0.04548 |
| cg15824705 | *BCAR1* | 9564 | 0.10 | 0.04549 |
| cg25699533 | *UBA52* | 7311 | 0.12 | 0.04549 |
| cg07955887 | *KIAA0802* | 23255 | -0.07 | 0.04550 |
| cg06943865 | *PEG10* | 23089 | -0.42 | 0.04551 |
| cg10983208 | *SPOCK2* | 9806 | 0.10 | 0.04552 |
| cg24336531 | *ARL6IP5* | 10550 | 0.08 | 0.04552 |
| cg11822964 | *PTGIR* | 5739 | 0.13 | 0.04552 |
| cg21604615 | *SYTL1* | 84958 | 0.39 | 0.04552 |
| cg22377389 | *GJB6* | 10804 | 0.08 | 0.04552 |
| cg00744433 | *CXADR* | 1525 | -0.28 | 0.04553 |
| cg14171523 | *KIAA0446* | 9673 | 0.09 | 0.04553 |
| cg17866455 | *SCAND2* | 54581 | -0.39 | 0.04553 |
| cg15520279 | *HOXD8* | 3234 | 0.11 | 0.04554 |
| cg24838010 | *SLC26A11* | 284129 | -0.35 | 0.04555 |
| cg15538837 | *H3F3A* | 3020 | 0.11 | 0.04556 |
| cg00795268 | *C11orf55* | 399879 | -0.08 | 0.04556 |
| cg02213716 | *PHGDHL1* | 337867 | 0.09 | 0.04556 |
| cg03750407 | *RAD9A* | 5883 | 0.09 | 0.04556 |
| cg07868688 | *ORC3L* | 23595 | 0.09 | 0.04556 |
| cg08682341 | *INPP5A* | 3632 | 0.13 | 0.04556 |
| cg08878304 | *FBXO7* | 25793 | 0.06 | 0.04556 |
| cg16486059 | *C1orf135* | 79000 | 0.09 | 0.04556 |
| cg21902327 | *FGF6* | 2251 | -0.52 | 0.04556 |
| cg22524514 | *CST6* | 1474 | -0.11 | 0.04556 |
| cg25421647 | *UBE2L3* | 7332 | 0.10 | 0.04556 |
| cg25923018 | *C1orf75* | 55248 | 0.07 | 0.04556 |
| cg27226949 | *EFNB2* | 1948 | 0.12 | 0.04556 |
| cg02296128 | *GTF2F2* | 2963 | 0.06 | 0.04556 |
| cg06134936 | *HERC4* | 26091 | 0.12 | 0.04556 |
| cg12432709 | *CCDC68* | 80323 | -0.30 | 0.04556 |
| cg13084525 | *EHF* | 26298 | 0.20 | 0.04556 |
| cg24356289 | *C12orf22* | 81566 | 0.12 | 0.04558 |
| cg10331048 | *MRPL11* | 65003 | 0.10 | 0.04558 |
| cg13021725 | *C20orf172* | 79980 | 0.09 | 0.04558 |
| cg13439299 | *DNAJC5G* | 285126 | -0.27 | 0.04558 |
| cg15422147 | *SERPINB5* | 5268 | -0.34 | 0.04558 |
| cg19362572 | *SHC4* | 399694 | 0.16 | 0.04558 |
| cg07671976 | *TNFRSF18* | 8784 | 0.41 | 0.04560 |
| cg03370542 | *ATG4C* | 84938 | 0.11 | 0.04562 |
| cg14711016 | *NARFL* | 64428 | 0.11 | 0.04562 |
| cg21396405 | *MAFG* | 4097 | 0.10 | 0.04562 |
| cg24727203 | *PPP1R3B* | 79660 | 0.08 | 0.04562 |
| cg01057962 | *BCL6* | 604 | 0.09 | 0.04563 |
| cg10663973 | *MRFAP1* | 93621 | 0.12 | 0.04563 |
| cg18330611 | *NPPC* | 4880 | 0.08 | 0.04563 |
| cg23367478 | *PDE3A* | 5139 | 0.09 | 0.04563 |
| cg24760768 | *DNAH8* | 1769 | -0.34 | 0.04563 |
| cg25241845 | *NEURL2* | 140825 | 0.09 | 0.04563 |
| cg27173322 | *FADS1* | 3992 | 0.10 | 0.04563 |
| cg02937060 | *ANP32E* | 81611 | 0.09 | 0.04564 |
| cg15727249 | *APOA4* | 337 | 0.08 | 0.04564 |
| cg22506059 | *CARD10* | 29775 | -0.38 | 0.04564 |
| cg27662379 | *RPN1* | 6184 | 0.11 | 0.04564 |
| cg00292662 | *LGALS1* | 3956 | 0.11 | 0.04564 |
| cg00417297 | *ZNF619* | 285267 | 0.09 | 0.04564 |
| cg01897036 | *SCTR* | 6344 | -0.40 | 0.04564 |
| cg03764161 | *FAM111A* | 63901 | 0.09 | 0.04564 |
| cg04025889 | *AHCY* | 191 | 0.08 | 0.04564 |
| cg05241571 | *UNQ467* | 388533 | -0.07 | 0.04564 |
| cg05382565 | *TIGD6* | 81789 | 0.10 | 0.04564 |
| cg05556202 | *TM4SF19* | 116211 | -0.39 | 0.04564 |
| cg06762858 | *PILRA* | 29992 | 0.17 | 0.04564 |
| cg06953768 | *FLJ37562* | 134553 | 0.16 | 0.04564 |
| cg07499806 | *ZSCAN5* | 79149 | -0.24 | 0.04564 |
| cg07803864 | *TUBAL3* | 79861 | -0.16 | 0.04564 |
| cg08935243 | *CETN3* | 1070 | 0.11 | 0.04564 |
| cg09049660 | *PANK2* | 80025 | 0.09 | 0.04564 |
| cg09299381 | *DNAL4* | 10126 | 0.11 | 0.04564 |
| cg10164686 | *SERPINB6* | 5269 | 0.09 | 0.04564 |
| cg11679069 | *DNAJC15* | 29103 | -0.27 | 0.04564 |
| cg12315997 | *RPL9* | 6133 | 0.13 | 0.04564 |
| cg13144783 | *CCR1* | 1230 | 0.27 | 0.04564 |
| cg14078518 | *GK2* | 2712 | -0.39 | 0.04564 |
| cg14093125 | *MGC13170* | 84798 | 0.08 | 0.04564 |
| cg14329976 | *NEIL2* | 252969 | 0.06 | 0.04564 |
| cg14792480 | *CDH16* | 1014 | 0.20 | 0.04564 |
| cg16098726 | *GP9* | 2815 | 0.43 | 0.04564 |
| cg16356516 | *EXOSC6* | 118460 | -0.37 | 0.04564 |
| cg18875839 | *KIF13B* | 23303 | 0.07 | 0.04564 |
| cg18958531 | *M6PRBP1* | 10226 | 0.11 | 0.04564 |
| cg20261167 | *SPP1* | 6696 | 0.37 | 0.04564 |
| cg20542800 | *LTB4R* | 1241 | 0.33 | 0.04564 |
| cg20603424 | *DDX31* | 64794 | 0.08 | 0.04564 |
| cg23271318 | *ZSWIM1* | 90204 | 0.10 | 0.04564 |
| cg25861458 | *FLJ23356* | 84197 | 0.10 | 0.04564 |
| cg26360732 | *PPP1R2* | 5504 | 0.09 | 0.04564 |
| cg03359285 | *TOP2A* | 7153 | 0.10 | 0.04564 |
| cg26751195 | *SPN* | 6693 | 0.45 | 0.04564 |
| cg01488147 | *PEG10* | 23089 | -0.29 | 0.04565 |
| cg04222374 | *CXorf40A* | 91966 | 0.08 | 0.04565 |
| cg12428604 | *TMEM60* | 85025 | 0.09 | 0.04565 |
| cg14560895 | *JARID1B* | 10765 | 0.12 | 0.04565 |
| cg22493172 | *POU6F1* | 5463 | 0.09 | 0.04565 |
| cg25094569 | *WT1* | 7490 | 0.11 | 0.04565 |
| cg01852328 | *LOC221955* | 221955 | 0.10 | 0.04566 |
| cg24545967 | *SH2D3C* | 10044 | 0.32 | 0.04566 |
| cg00551244 | *C8orf78* | 157376 | -0.32 | 0.04567 |
| cg01535453 | *ACADS* | 35 | 0.09 | 0.04567 |
| cg02312751 | *TSSC4* | 10078 | 0.09 | 0.04567 |
| cg02577849 | *ADAMTS6* | 11174 | 0.09 | 0.04567 |
| cg04700925 | *KIAA0922* | 23240 | 0.10 | 0.04567 |
| cg18678121 | *SEC61A2* | 55176 | -0.28 | 0.04567 |
| cg19837824 | *NTHL1* | 4913 | 0.07 | 0.04567 |
| cg19964192 | *MAGEA10* | 4109 | -0.45 | 0.04567 |
| cg25059899 | *BCL2* | 596 | -0.24 | 0.04567 |
| cg21944757 | *ARFGAP1* | 55738 | 0.08 | 0.04567 |
| cg01318557 | *LAT2* | 7462 | 0.28 | 0.04567 |
| cg09825414 | *TCOF1* | 6949 | -0.40 | 0.04567 |
| cg11408517 | *LATS1* | 9113 | 0.10 | 0.04567 |
| cg13678049 | *PARC* | 23113 | -0.31 | 0.04567 |
| cg14707431 | *C9orf97* | 158427 | 0.11 | 0.04567 |
| cg17685111 | *BMP8A* | 353500 | -0.26 | 0.04567 |
| cg22748573 | *CITED4* | 163732 | 0.09 | 0.04567 |
| cg23146358 | *CDKN1C* | 1028 | 0.08 | 0.04567 |
| cg02613803 | *TMPRSS2* | 7113 | 0.09 | 0.04567 |
| cg06958211 | *PAK6* | 56924 | -0.14 | 0.04567 |
| cg17129388 | *NGFR* | 4804 | -0.30 | 0.04567 |
| cg07360692 | *FLJ20032* | 54790 | 0.09 | 0.04567 |
| cg02784848 | *FLJ22688* | 80199 | 0.11 | 0.04567 |
| cg08897388 | *LAMA4* | 3910 | 0.09 | 0.04567 |
| cg22967016 | *MICAL2* | 9645 | 0.09 | 0.04567 |
| cg04315771 | *IL4I1* | 259307 | -0.38 | 0.04568 |
| cg15816080 | *TPST1* | 8460 | 0.09 | 0.04568 |
| cg08132931 | *ADD2* | 119 | 0.09 | 0.04568 |
| cg05064673 | *PTPRU* | 10076 | 0.10 | 0.04568 |
| cg01743008 | *MAGEA2* | 4101 | -0.40 | 0.04568 |
| cg07408740 | *OGDH* | 4967 | -0.27 | 0.04568 |
| cg14171478 | *C14orf118* | 55668 | 0.09 | 0.04568 |
| cg20377762 | *TMEM61* | 199964 | -0.40 | 0.04568 |
| cg20469837 | *GALNT5* | 11227 | 0.33 | 0.04568 |
| cg20884362 | *C21orf70* | 85395 | -0.23 | 0.04568 |
| cg22233974 | *MACF1* | 23499 | -0.38 | 0.04568 |
| cg26727372 | *ME2* | 4200 | 0.12 | 0.04568 |
| cg26920757 | *SH2D3C* | 10044 | 0.39 | 0.04568 |
| cg27333993 | *RBM3* | 5935 | 0.09 | 0.04568 |
| cg15427567 | *FLJ20859* | 64745 | 0.08 | 0.04568 |
| cg15175266 | *CDH12* | 1010 | -0.21 | 0.04568 |
| cg09343544 | *DGCR14* | 8220 | 0.08 | 0.04569 |
| cg15195412 | *CX3CL1* | 6376 | 0.22 | 0.04569 |
| cg26368842 | *PIP5K2A* | 5305 | -0.38 | 0.04569 |
| cg01307730 | *HIST1H2AA* | 221613 | -0.37 | 0.04571 |
| cg01584473 | *MUC17* | 140453 | 0.50 | 0.04571 |
| cg06731484 | *IVD* | 3712 | 0.08 | 0.04571 |
| cg06810647 | *CRAMP1L* | 57585 | 0.10 | 0.04571 |
| cg21089667 | *SMARCA3* | 6596 | -0.47 | 0.04571 |
| cg22858288 | *SLC22A18* | 5002 | -0.48 | 0.04571 |
| cg03192737 | *OSTN* | 344901 | 0.15 | 0.04571 |
| cg04740359 | *NTF3* | 4908 | -0.23 | 0.04571 |
| cg14519036 | *TNKS2* | 80351 | 0.10 | 0.04571 |
| cg19376794 | *USP39* | 10713 | 0.19 | 0.04571 |
| cg23859078 | *C7orf30* | 115416 | 0.08 | 0.04571 |
| cg06121469 | *KIAA1840* | 80208 | 0.09 | 0.04572 |
| cg17386181 | *MT1B* | 4490 | -0.10 | 0.04572 |
| cg09640202 | *IMP4* | 92856 | -0.37 | 0.04574 |
| cg12770741 | *NXN* | 64359 | -0.30 | 0.04574 |
| cg18966791 | *C1orf61* | 10485 | 0.38 | 0.04574 |
| cg10849854 | *GREB1* | 9687 | 0.31 | 0.04574 |
| cg08361238 | *PTGFRN* | 5738 | -0.40 | 0.04574 |
| cg17043922 | *C6orf106* | 64771 | 0.11 | 0.04574 |
| cg00695416 | *CBR1* | 873 | 0.09 | 0.04575 |
| cg03064400 | *U2AF1L3* | 199746 | 0.14 | 0.04575 |
| cg03775123 | *ANKDD1A* | 348094 | 0.31 | 0.04575 |
| cg05593325 | *GALNT12* | 79695 | -0.31 | 0.04575 |
| cg05719902 | *LOC136306* | 136306 | -0.26 | 0.04575 |
| cg07271268 | *JARID1A* | 5927 | 0.09 | 0.04575 |
| cg07965839 | *ZA20D3* | 54469 | 0.08 | 0.04575 |
| cg09234474 | *DUSP14* | 11072 | 0.07 | 0.04575 |
| cg12949975 | *GDF7* | 151449 | 0.10 | 0.04575 |
| cg13743031 | *NEIL3* | 55247 | 0.07 | 0.04575 |
| cg16386080 | *CCRK* | 23552 | -0.19 | 0.04575 |
| cg17885062 | *NRCAM* | 4897 | 0.11 | 0.04575 |
| cg18110483 | *THBS4* | 7060 | 0.09 | 0.04575 |
| cg20576002 | *FAM112B* | 121355 | -0.38 | 0.04575 |
| cg21781546 | *ZNF142* | 7701 | -0.09 | 0.04575 |
| cg21802726 | *C20orf31* | 55741 | 0.12 | 0.04575 |
| cg24323031 | *PAQR8* | 85315 | 0.10 | 0.04575 |
| cg24625128 | *JAM3* | 83700 | 0.09 | 0.04575 |
| cg25042226 | *PAX8* | 7849 | -0.46 | 0.04575 |
| cg26385222 | *HCA112* | 55365 | -0.37 | 0.04575 |
| cg26583078 | *SORBS2* | 8470 | -0.37 | 0.04575 |
| cg26917999 | *LZTS1* | 11178 | 0.17 | 0.04575 |
| cg27549944 | *PLEKHA6* | 22874 | 0.31 | 0.04575 |
| cg01221637 | *C20orf112* | 140688 | 0.09 | 0.04575 |
| cg01643284 | *DIP13B* | 55198 | 0.09 | 0.04575 |
| cg02554564 | *NTF3* | 4908 | -0.39 | 0.04575 |
| cg09152089 | *IL22RA1* | 58985 | 0.51 | 0.04575 |
| cg10364513 | *RXRG* | 6258 | 0.08 | 0.04575 |
| cg11735358 | *ATP5F1* | 515 | 0.09 | 0.04575 |
| cg11930592 | *MSX1* | 4487 | 0.11 | 0.04575 |
| cg15020645 | *APC* | 324 | 0.09 | 0.04575 |
| cg16150435 | *C6orf15* | 29113 | 0.51 | 0.04575 |
| cg16511708 | *SS18* | 6760 | 0.09 | 0.04575 |
| cg18445047 | *MGC26885* | 124044 | 0.11 | 0.04575 |
| cg19194595 | *PHF16* | 9767 | 0.12 | 0.04575 |
| cg20385229 | *ALKBH* | 8846 | -0.35 | 0.04575 |
| cg23228178 | *PADI4* | 23569 | 0.12 | 0.04575 |
| cg24123124 | *MRPS17* | 51373 | 0.06 | 0.04575 |
| cg24254206 | *SNRPD2* | 6633 | -0.17 | 0.04575 |
| cg26081162 | *C6orf141* | 135398 | 0.11 | 0.04575 |
| cg26356176 | *SNAG1* | 112574 | 0.10 | 0.04575 |
| cg27383956 | *FLJ13265* | 79935 | -0.39 | 0.04575 |
| cg00367438 | *C7orf21* | 83590 | 0.16 | 0.04576 |
| cg03400060 | *BHMT2* | 23743 | -0.30 | 0.04576 |
| cg06177968 | *WDR55* | 54853 | 0.08 | 0.04576 |
| cg07476030 | *RTN4IP1* | 84816 | -0.25 | 0.04576 |
| cg14894144 | *LAMA3* | 3909 | -0.15 | 0.04576 |
| cg18346038 | *CDC91L1* | 128869 | 0.11 | 0.04576 |
| cg18876487 | *AIRE* | 326 | -0.26 | 0.04576 |
| cg21544377 | *C12orf61* | 283416 | 0.09 | 0.04576 |
| cg23159337 | *ATP13A4* | 84239 | 0.34 | 0.04576 |
| cg24496423 | *STK23* | 26576 | 0.35 | 0.04576 |
| cg24650229 | *IGSF11* | 152404 | -0.54 | 0.04576 |
| cg24947394 | *USP33* | 23032 | 0.09 | 0.04576 |
| cg25087423 | *BLR1* | 643 | -0.07 | 0.04576 |
| cg25600606 | *HIPK3* | 10114 | 0.31 | 0.04576 |
| cg26607785 | *TBX21* | 30009 | -0.39 | 0.04576 |
| cg07531356 | *INSL6* | 11172 | -0.32 | 0.04576 |
| cg12927153 | *PTGS1* | 5742 | 0.08 | 0.04576 |
| cg18746357 | *RELA* | 5970 | -0.42 | 0.04576 |
| cg09149294 | *ARRDC4* | 91947 | 0.07 | 0.04577 |
| cg17141902 | *NINJ1* | 4814 | -0.28 | 0.04577 |
| cg18708106 | *SUCLA2* | 8803 | 0.11 | 0.04577 |
| cg20837735 | *SERPINB5* | 5268 | -0.32 | 0.04578 |
| cg13812587 | *TMEM15* | 22845 | 0.08 | 0.04578 |
| cg24751129 | *GNMT* | 27232 | -0.40 | 0.04578 |
| cg26661481 | *IL10RA* | 3587 | -0.24 | 0.04578 |
| cg02184413 | *VNN1* | 8876 | 0.27 | 0.04578 |
| cg23698969 | *SLC22A18* | 5002 | -0.53 | 0.04579 |
| cg17319849 | *GBA* | 2629 | 0.09 | 0.04580 |
| cg02712165 | *C17orf76* | 388341 | -0.37 | 0.04581 |
| cg05636175 | *TNFRSF10C* | 8794 | 0.09 | 0.04581 |
| cg09427311 | *ANGPTL2* | 23452 | 0.26 | 0.04581 |
| cg10052840 | *SEMA6B* | 10501 | -0.47 | 0.04581 |
| cg17976829 | *TXNL2* | 10539 | 0.11 | 0.04581 |
| cg21321885 | *NDE1* | 54820 | 0.09 | 0.04581 |
| cg00035347 | *NT5C2* | 22978 | -0.20 | 0.04581 |
| cg16177163 | *PTDSS1* | 9791 | -0.26 | 0.04581 |
| cg17386991 | *POLR3F* | 10621 | 0.07 | 0.04581 |
| cg23493704 | *SLC10A3* | 8273 | -0.33 | 0.04581 |
| cg18856252 | *TYK2* | 7297 | 0.11 | 0.04581 |
| cg09223071 | *SUPT7L* | 9913 | 0.08 | 0.04582 |
| cg01262913 | *DSCR9* | 257203 | 0.31 | 0.04583 |
| cg02235880 | *FANCF* | 2188 | 0.11 | 0.04583 |
| cg02922563 | *TREX1* | 11277 | 0.12 | 0.04583 |
| cg05807991 | *TRAM1L1* | 133022 | 0.15 | 0.04583 |
| cg06143901 | *TCF15* | 6939 | -0.28 | 0.04583 |
| cg16133244 | *GNPTG* | 84572 | -0.23 | 0.04583 |
| cg16582174 | *ENTH* | 9685 | -0.27 | 0.04583 |
| cg16592658 | *EBI3* | 10148 | 0.42 | 0.04583 |
| cg18292711 | *KIF5B* | 3799 | -0.22 | 0.04583 |
| cg23089438 | *CSF1* | 1435 | 0.13 | 0.04583 |
| cg24907852 | *ENOSF1* | 55556 | 0.12 | 0.04583 |
| cg26718574 | *PAPD1* | 55149 | 0.09 | 0.04583 |
| cg12315311 | *UNQ3033* | 284415 | 0.38 | 0.04585 |
| cg08763433 | *SMG5* | 23381 | 0.07 | 0.04586 |
| cg04531254 | *MGC17839* | 219902 | 0.08 | 0.04586 |
| cg00828602 | *WNT5A* | 7474 | 0.10 | 0.04587 |
| cg05358404 | *RTEL1* | 51750 | -0.29 | 0.04587 |
| cg11331769 | *TAZ* | 6901 | -0.27 | 0.04587 |
| cg19157971 | *RABIF* | 5877 | 0.10 | 0.04587 |
| cg20017147 | *TEX101* | 83639 | 0.28 | 0.04587 |
| cg23181434 | *TLK2* | 11011 | -0.29 | 0.04587 |
| cg05720937 | *ACTR1B* | 10120 | 0.09 | 0.04587 |
| cg10857345 | *TEX13B* | 56156 | -0.34 | 0.04587 |
| cg13073894 | *ZNF566* | 84924 | 0.07 | 0.04587 |
| cg01929065 | *CD63* | 967 | 0.08 | 0.04590 |
| cg02060618 | *BMP15* | 9210 | 0.34 | 0.04590 |
| cg14129786 | *MGMT* | 4255 | 0.37 | 0.04590 |
| cg22136753 | *CSRP2BP* | 57325 | 0.08 | 0.04590 |
| cg24424381 | *PEX11A* | 8800 | 0.13 | 0.04590 |
| cg24511869 | *RELB* | 5971 | 0.08 | 0.04590 |
| cg27524460 | *PTGER1* | 5731 | 0.29 | 0.04590 |
| cg07636178 | *HIST1H3C* | 8352 | 0.11 | 0.04591 |
| cg07864632 | *TRIP12* | 9320 | 0.15 | 0.04592 |
| cg15555727 | *PYCRL* | 65263 | 0.11 | 0.04593 |
| cg07545232 | *MAGEA3* | 4102 | -0.43 | 0.04593 |
| cg07607921 | *ZZEF1* | 23140 | 0.07 | 0.04593 |
| cg12503243 | *PRPF39* | 55015 | 0.14 | 0.04593 |
| cg17864730 | *ITGB8* | 3696 | 0.09 | 0.04594 |
| cg23239396 | *CALCR* | 799 | 0.07 | 0.04594 |
| cg26810336 | *IL1RAPL1* | 11141 | 0.14 | 0.04594 |
| cg27322545 | *PLAA* | 9373 | 0.08 | 0.04594 |
| cg10046892 | *PAQR6* | 79957 | -0.12 | 0.04594 |
| cg02164442 | *ITGAD* | 3681 | 0.08 | 0.04595 |
| cg04464446 | *GAL* | 51083 | -0.25 | 0.04595 |
| cg21850239 | *POLR2G* | 5436 | 0.09 | 0.04595 |
| cg22497867 | *MAGEA4* | 4103 | -0.28 | 0.04595 |
| cg14918082 | *KCNAB3* | 9196 | -0.48 | 0.04595 |
| cg16883145 | *TGFB1* | 7040 | -0.38 | 0.04595 |
| cg04645342 | *NEGR1* | 257194 | 0.10 | 0.04596 |
| cg05996238 | *PAGE2* | 203569 | -0.41 | 0.04596 |
| cg12468189 | *STK23* | 26576 | 0.26 | 0.04596 |
| cg13155059 | *CAPZA2* | 830 | 0.07 | 0.04596 |
| cg20718816 | *LTBP2* | 4053 | 0.10 | 0.04596 |
| cg16900022 | *MDN1* | 23195 | 0.08 | 0.04597 |
| cg24441350 | *TPPP* | 11076 | 0.09 | 0.04597 |
| cg19762747 | *CRX* | 1406 | 0.29 | 0.04598 |
| cg04731384 | *MGC3101* | 79007 | 0.51 | 0.04600 |
| cg07423149 | *CHI3L1* | 1116 | 0.26 | 0.04600 |
| cg15818800 | *DNAJC1* | 64215 | 0.10 | 0.04600 |
| cg06899976 | *PCDHB1* | 29930 | -0.39 | 0.04600 |
| cg13915726 | *DUSP9* | 1852 | 0.17 | 0.04600 |
| cg24471894 | *KIAA0020* | 9933 | 0.32 | 0.04600 |
| cg02602411 | *PPP3R2* | 5535 | -0.30 | 0.04602 |
| cg19430430 | *COL5A3* | 50509 | 0.10 | 0.04603 |
| cg14058998 | *SEPW1* | 6415 | 0.05 | 0.04605 |
| cg09712606 | *IRF3* | 3661 | 0.09 | 0.04605 |
| cg11235871 | *QARS* | 5859 | 0.07 | 0.04605 |
| cg06598256 | *DNAJC4* | 3338 | -0.10 | 0.04606 |
| cg07922606 | *HIST1H3E* | 8353 | -0.46 | 0.04606 |
| cg08572611 | *ACTL6B* | 51412 | 0.11 | 0.04606 |
| cg10525372 | *WISP3* | 8838 | -0.33 | 0.04606 |
| cg20334738 | *MAB21L2* | 10586 | -0.32 | 0.04606 |
| cg20370277 | *NCKIPSD* | 51517 | 0.10 | 0.04606 |
| cg22764925 | *GGT1* | 2678 | 0.40 | 0.04606 |
| cg23242017 | *LMTK2* | 22853 | 0.10 | 0.04606 |
| cg23274123 | *C1orf96* | 126731 | 0.12 | 0.04606 |
| cg03020597 | *SLITRK2* | 84631 | 0.13 | 0.04606 |
| cg05308617 | *ARMC8* | 25852 | 0.10 | 0.04606 |
| cg09001953 | *FLJ11200* | 55325 | 0.15 | 0.04606 |
| cg15928132 | *CCKAR* | 886 | -0.36 | 0.04606 |
| cg25575359 | *PRPF19* | 27339 | 0.10 | 0.04606 |
| cg26461352 | *TIMM8A* | 1678 | 0.25 | 0.04606 |
| cg27485921 | *ATP6V1E2* | 90423 | 0.34 | 0.04606 |
| cg05749161 | *RASA4* | 10156 | 0.11 | 0.04607 |
| cg03712708 | *CKS2* | 1164 | 0.10 | 0.04607 |
| cg20295671 | *YPEL1* | 29799 | 0.07 | 0.04607 |
| cg25786436 | *PRKAG2* | 51422 | 0.09 | 0.04607 |
| cg17324707 | *C3orf28* | 26355 | 0.08 | 0.04607 |
| cg00003994 | *MEOX2* | 4223 | 0.08 | 0.04607 |
| cg01131735 | *SC65* | 10609 | 0.11 | 0.04607 |
| cg08918431 | *MASK-BP3* | 404734 | 0.08 | 0.04607 |
| cg12951282 | *ASGR2* | 433 | 0.30 | 0.04607 |
| cg18053505 | *PCAF* | 8850 | 0.14 | 0.04607 |
| cg23641264 | *JRK* | 8629 | -0.09 | 0.04607 |
| cg25887739 | *TBC1D8* | 11138 | -0.09 | 0.04607 |
| cg01348086 | *RGS14* | 10636 | 0.37 | 0.04607 |
| cg27465531 | *MBTPS2* | 51360 | 0.13 | 0.04608 |
| cg22797169 | *IL1RL2* | 8808 | -0.32 | 0.04611 |
| cg02358761 | *STK17B* | 9262 | 0.08 | 0.04611 |
| cg11678767 | *COL4A6* | 1288 | 0.11 | 0.04612 |
| cg15425280 | *GRIA2* | 2891 | 0.08 | 0.04612 |
| cg22168676 | *OFCC1* | 266553 | 0.12 | 0.04612 |
| cg22378065 | *PLAGL1* | 5325 | -0.62 | 0.04612 |
| cg23684521 | *SDFR1* | 27020 | 0.08 | 0.04612 |
| cg26516954 | *B3GNTL1* | 146712 | 0.07 | 0.04612 |
| cg03143333 | *CCDC11* | 220136 | -0.30 | 0.04612 |
| cg03622689 | *C21orf67* | 84536 | 0.11 | 0.04612 |
| cg08858521 | *WFIKKN1* | 117166 | 0.38 | 0.04612 |
| cg16889368 | *MRPL15* | 29088 | 0.09 | 0.04612 |
| cg26045220 | *KIAA1324* | 57535 | 0.08 | 0.04612 |
| cg08210616 | *ZNF502* | 91392 | 0.11 | 0.04613 |
| cg23772557 | *USP16* | 10600 | 0.10 | 0.04615 |
| cg23124451 | *CBX7* | 23492 | -0.29 | 0.04615 |
| cg03032025 | *CPEB4* | 80315 | -0.24 | 0.04615 |
| cg10301967 | *RPS18* | 6222 | 0.08 | 0.04615 |
| cg12748258 | *HR* | 55806 | 0.09 | 0.04615 |
| cg19676312 | *RNPEP* | 6051 | 0.09 | 0.04615 |
| cg27226618 | *ACP1* | 52 | -0.34 | 0.04615 |
| cg21533271 | *PLXNA3* | 55558 | 0.17 | 0.04616 |
| cg06434451 | *MARCKSL1* | 65108 | -0.30 | 0.04618 |
| cg07535879 | *MTRF1L* | 54516 | 0.11 | 0.04618 |
| cg23855093 | *GPR128* | 84873 | 0.16 | 0.04618 |
| cg11595794 | *ANKRD37* | 353322 | 0.10 | 0.04619 |
| cg17154022 | *HMGN2* | 3151 | 0.13 | 0.04619 |
| cg18992201 | *DPPA2* | 151871 | -0.41 | 0.04619 |
| cg23722792 | *SCN4A* | 6329 | -0.06 | 0.04619 |
| cg04171565 | *FLJ22624* | 79866 | 0.08 | 0.04619 |
| cg09607282 | *RTN2* | 6253 | 0.17 | 0.04619 |
| cg12504957 | *MDM2* | 4193 | 0.10 | 0.04619 |
| cg13467814 | *C2orf30* | 27248 | 0.09 | 0.04619 |
| cg02331561 | *ABCA3* | 21 | 0.09 | 0.04620 |
| cg14460735 | *C1orf71* | 163882 | -0.31 | 0.04620 |
| cg25092283 | *SORBS1* | 10580 | -0.29 | 0.04620 |
| cg08901867 | *TTLL6* | 284076 | -0.07 | 0.04621 |
| cg12967560 | *LYSMD4* | 145748 | -0.36 | 0.04621 |
| cg17683775 | *PTGES* | 9536 | 0.13 | 0.04621 |
| cg25756129 | *SFRS10* | 6434 | 0.11 | 0.04621 |
| cg00109274 | *DGKH* | 160851 | 0.08 | 0.04622 |
| cg06700265 | *DUSP16* | 80824 | 0.08 | 0.04622 |
| cg09143663 | *BACH1* | 571 | -0.28 | 0.04622 |
| cg11976048 | *MGC4172* | 79154 | 0.08 | 0.04622 |
| cg14958635 | *NEUROG1* | 4762 | 0.10 | 0.04622 |
| cg17910564 | *VDAC3* | 7419 | -0.34 | 0.04622 |
| cg21430666 | *HIST3H3* | 8290 | -0.60 | 0.04622 |
| cg03242880 | *BTG4* | 54766 | -0.31 | 0.04623 |
| cg14264994 | *CTCFL* | 140690 | -0.38 | 0.04623 |
| cg17188169 | *DDX43* | 55510 | -0.40 | 0.04623 |
| cg25903375 | *SET7* | 80854 | 0.09 | 0.04623 |
| cg21096966 | *BTBD5* | 54813 | -0.32 | 0.04623 |
| cg08259617 | *GADD45B* | 4616 | 0.06 | 0.04624 |
| cg19573464 | *KPNB1* | 3837 | 0.07 | 0.04625 |
| cg26872475 | *NOD9* | 79671 | 0.09 | 0.04625 |
| cg04586563 | *DIP2C* | 22982 | -0.40 | 0.04625 |
| cg07077459 | *PLAGL1* | 5325 | -0.64 | 0.04625 |
| cg14912575 | *C14orf162* | 56936 | -0.27 | 0.04625 |
| cg12870705 | *NRP1* | 8829 | 0.14 | 0.04626 |
| cg26530497 | *TEB1* | 54937 | -0.40 | 0.04626 |
| cg00002426 | *SLMAP* | 7871 | -0.26 | 0.04627 |
| cg05063104 | *SLC5A8* | 160728 | -0.24 | 0.04627 |
[truncated: 63,015 more chars]
