# Supplementary material for: Integrative DNA Methylation and Gene Expression Analyses Identify DNA Packaging and Epigenetic Regulatory Genes Associated with Low Motility Sperm
Source: PLoS One. 2011 Jun 2;6(6):e20280. doi: 10.1371/journal.pone.0020280 (PMC3107223; doi:10.1371/journal.pone.0020280)
Supplement: Table S4 — Aberrant mRNA Transcripts in Low Motility Sperm. (DOC) [file pone.0020280.s004.doc]

| **Table S4: Aberrant mRNA Transcripts in Low Motility Sperm** | | | |
| --- | --- | --- | --- |
| **Gene** | **Slope (log_2_)** | **Fold Change** | **Q-value** |
| *GLI3* | 2.5 | -5.7 | 0.016 |
| *SERPINA5* | 3.7 | -13.0 | 0.016 |
| *ACE* | 1.9 | -3.7 | 0.020 |
| *APBA1* | 1.5 | -2.8 | 0.020 |
| *HDAC1* | -3.2 | 9.2 | 0.020 |
| *CTNND2* | 2.1 | -4.3 | 0.028 |
| *FERMT2* | 2.2 | -4.6 | 0.028 |
| *CYP19A1* | 2.3 | -4.9 | 0.028 |
| *PHPT1* | 2.1 | -4.3 | 0.028 |
| *SNRPN* | 2.7 | -6.5 | 0.028 |
| *FANCC* | 2.3 | -4.9 | 0.030 |
| *SIRT3* | 1.6 | -3.0 | 0.036 |
| *DNMT3A* | -1.0 | 2.0 | 0.041 |
| *PPP1R9A* | 2.1 | -4.3 | 0.041 |
| *CDH18* | 2.1 | -4.3 | 0.042 |
| *ALDH1L1* | 1.5 | -2.8 | 0.046 |
| *LDB1* | -3.0 | 8.0 | 0.046 |
| *PCSK4* | 2.6 | -6.1 | 0.047 |
| *PEX10* | 2.0 | -4.0 | 0.047 |
| *FAS* | -2.6 | 6.1 | 0.049 |
| Note: Genes with a negative slope (-) have increased transcript presence in the low motility samples. Genes with a positive slope (+) have decreased transcript presence in the low motility samples. | | | |
